# Exploring the full catalytic cycle of rhodium(i)–BINAP-catalysed isomerisation of allylic amines: a graph theory approach for path optimisation†
†Electronic supplementary information (ESI) available. See DOI: 10.1039/c7sc00401j



**DOI:** 10.1039/c7sc00401j

**Published:** 2017-05-03

**Authors:** Takayoshi Yoshimura, Satoshi Maeda, Tetsuya Taketsugu, Masaya Sawamura, Keiji Morokuma, Seiji Mori

**Affiliations:** a Institute of Quantum Beam Science , Ibaraki University , Mito 310-8512 , Japan . Email: seiji.mori.compchem@vc.ibaraki.ac.jp; b Department of Chemistry , Faculty of Science , Hokkaido University , Kita-10, Nishi-8, Kita-ku , Sapporo 060-0810 , Japan . Email: smaeda@eis.hokudai.ac.jp; c Fukui Institute for Fundamental Chemistry , Kyoto University , 34-4 Takano Nishihiraki-cho, Sakyo , Kyoto 606-8103 , Japan . Email: morokuma.keiji.3a@kyoto-u.ac.jp

## Abstract

We explored the reaction mechanism of the cationic rhodium(i)–BINAP complex catalysed isomerisation of allylic amines using the artificial force induced reaction method with the global reaction route mapping strategy, which enabled us to search for various reaction paths without assumption of transition states. The entire reaction network was reproduced in the form of a graph, and reasonable paths were selected from the complicated network using Prim’s algorithm. As a result, a new dissociative reaction mechanism was proposed. Our comprehensive reaction path search provided rationales for the *E*/*Z* and *S*/*R* selectivities of the stereoselective reaction.

## Abstract 2

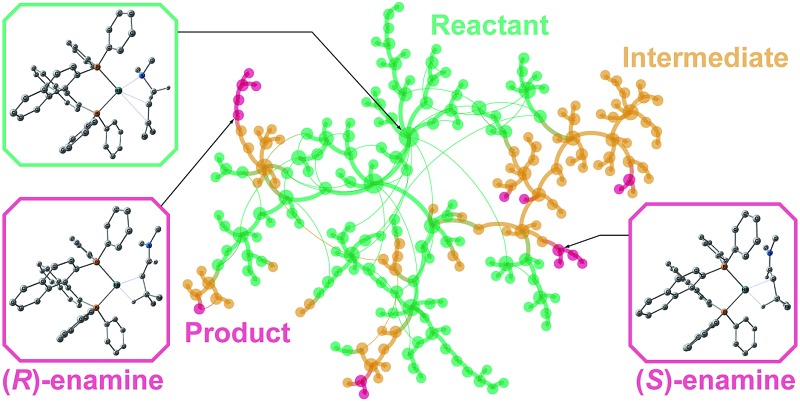
The reaction mechanism of the cationic rhodium(i)–BINAP complex catalysed isomerisation of allylic amines was explored using the artificial force induced reaction method with the global reaction route mapping strategy.

## 1 Introduction

**Fig. 1:**
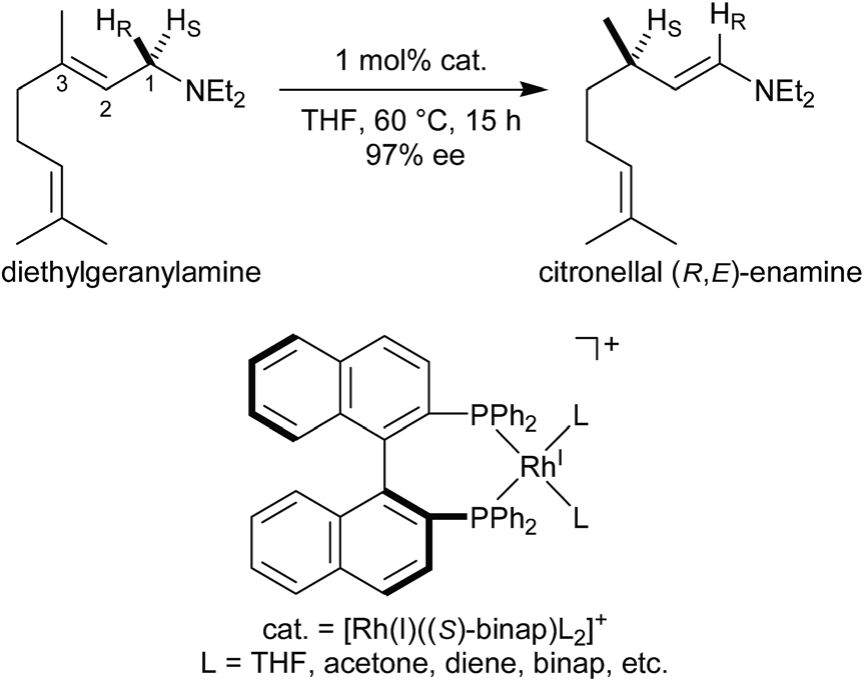
The asymmetric 1,3-hydrogen shift of diethylgeranylamine.

Noyori *et al.* reported the BINAP–metal-catalysed hydrogenation reaction of enamides with excellent enantioselectivity; we have previously examined the mechanisms of this reaction using the quantum mechanics/molecular mechanics (QM/MM) method.^1–7^ Otsuka *et al.* reported a highly regioselective and enantioselective 1,3-hydrogen shift reaction of allylic amines using a cationic rhodium(i)–BINAP catalyst (Fig. 1).^8–13^ The asymmetric 1,3-hydrogen shift of diethylgeranylamine forms a corresponding citronellal (*R*,*E*)-enamine, which generates a stereogenic carbon centre from a prochiral compound. This reaction has been used in the stereoselective synthesis of l-menthol.^14^ In addition, the isomerisation includes a C–H bond cleavage step. Thus, knowledge of the mechanism of this reaction would be highly meaningful in the context of metal-catalysed C–H functionalisation chemistry.^15^

**Fig. 2:**
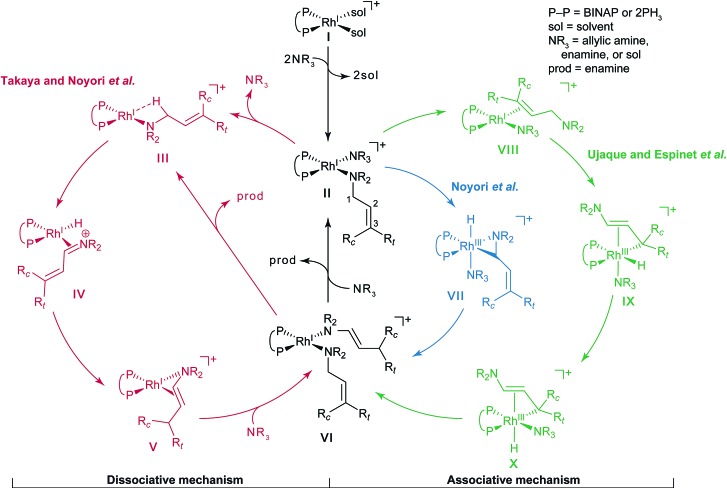
Three catalytic cycles in the literature: the routes proposed by Takaya and Noyori *et al.* (red), Noyori *et al.* (blue), and Ujaque and Espinet *et al.* (green).

Three reaction mechanisms have previously been reported based on experimental and computational studies (Fig. 2).^16–18^ All of these mechanisms start from the bis-solvent coordinate Rh(i)–BINAP complex **I**, and the bis-substrate coordinate complex **II** is formed through ligand substitution. The difference in the three mechanisms lies in the C(1)–H σ-bond cleavage step. The first mechanism, proposed by Noyori, is a dissociative mechanism and includes the formation of the transient iminium–Rh(i)-hydride complex **IV** through β-hydride elimination from the mono-amine coordinate complex **III**.^16^ Complex **IV** is converted to the η^3^-enamine coordinate complex **V** by the transfer of a hydride from the rhodium to C(3). Ligand substitution from complex **V** forms complex **II** or complex **III** through the bis-amine coordinate Rh(i) complex **VI**. However, *ab initio* molecular orbital studies showed that the associative mechanism is more advantageous than Noyori’s dissociative mechanism.^17^ A second mechanism, Takaya and Noyori’s mechanism, was then proposed. They suggested that the C(1)–H oxidative addition occurs directly from complex **II** without liberation of an NR_3_ molecule. This mechanism involves a Rh(i)/Rh(iii) two-electron-redox process through a characteristic distorted-octahedral Rh(iii) hydride complex **VII**. Complex **VI** is formed by reductive elimination from complex **VII**. Later, Ujaque and Espinet proposed a more reasonable allylmetal mechanism based on density functional theory (DFT) studies.^18^ This mechanism begins with intramolecular isomerisation of κ^1^-(*N*)-coordinate complex **II** to η^2^-(C=C)-coordinate complex **VIII**, which then undergoes C(1)–H σ-bond cleavage to form distorted-octahedral η^3^-allyl complex **IX**. After a conformational exchange from **IX** to **X**, the reaction concludes with reductive elimination to form complex **VI**.

The three mechanisms were proposed based on assumptions of the intermediates and transition states (TSs). To eliminate the need for this assumption to allow exploration of the reaction paths, we used a reaction path search method called artificial force induced reaction (AFIR), which can algorithmically predict reaction paths and TSs on reaction paths. To analyse a complicated reaction path network obtained using the AFIR method, Prim’s algorithm was employed. Although kinetic approaches such as rate constant matrix contraction^19,20^ and kinetic Monte Carlo^21–23^ have been used in evaluating quantities such as overall rate constants and branching ratios, graph theory approaches including Prim’s algorithm would be useful to visualise an overview of highly multistep reaction paths. Furthermore, our own N-layered integrated molecular orbital + molecular mechanics (ONIOM) method was employed to reduce the computational cost, which enabled us to use the structure of the Rh(i)–BINAP complex without replacing the functional groups. In the studies by Noyori *et al.* ^17^ and Ujaque and Espinet *et al.*,^18^ two PH_3_ molecules were used as a model of the BINAP ligand for the same purpose; as a result of this simplification, the associative mechanism appeared to be the most viable. However, our results, without simplification of the Rh(i)–BINAP complex, demonstrated the advantages of the dissociative mechanism over the associative mechanism.

## 2 Computational methods

### 2.1 Outline of the computational procedure

**Fig. 3:**
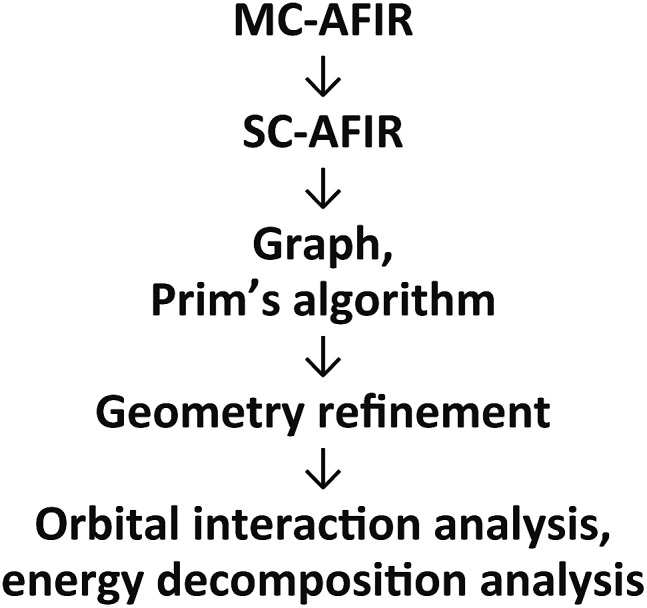
The computational procedure used in the present study.

An outline of our computational procedure is shown in Fig. 3. We employed the AFIR method coded in the global reaction route mapping (GRRM) programme to explore the reaction path network.^24–31^ There are two modes for the AFIR method. In the multi-component mode (MC-AFIR), the force is applied between two or more reactant molecules to induce a reaction between the reactants. In the single-component mode (SC-AFIR), fragments are defined automatically in an entire system (molecule or complex) and the force is applied between the fragments. In this study, the MC-AFIR and SC-AFIR were used, respectively, in the coordination state sampling and in the path sampling within the coordination complexes. The graph consists of connections between nodes and edges. In the present study, a node and an edge represent a local minimum (LM) and a TS, respectively. Prim’s algorithm was employed to construct a minimum spanning tree (MST) from the graph. Examination of the algorithm procedure demonstrates that the MST shows energetically reasonable paths. Finally, some LMs and TSs were reoptimised and analysed at higher computational levels for more detailed study.

### 2.2 Coordination state search with the MC-AFIR method

**Fig. 4:**
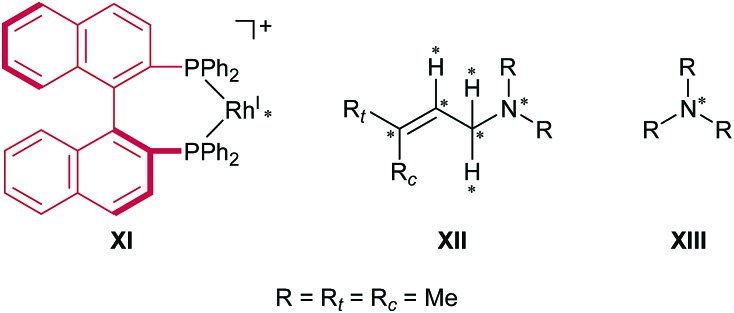
Computational models. Artificial force was applied between the atoms marked with asterisks. The MM region is depicted in red and the QM region is depicted in black.

The models that we considered are shown in Fig. 4. The full cationic Rh(i)–BINAP complex **XI** was used as a catalyst. To simplify the models of the substrates, *N*,*N*,3-trimethylbut-2-en-1-amine **XII** and trimethylamine **XIII** were used as the substrates. To examine the enantiotopic face, two methyl groups on the allylic group were indicated as R_*t*_ (*trans*) and R_*c*_ (*cis*); the priority of R_*t*_ was established to be higher than that of R_*c*_. The ONIOM-type hybrid QM/MM model was employed to reduce the computational cost.^32–39^ The QM and MM regions in the ONIOM method were defined as given in Fig. 4. The MM region was treated at the universal force field (UFF) level, while the QM region was treated at the B3LYP level in conjunction with the LANL2DZ basis set for the rhodium atom and the 6-31G basis set for other atoms (BS1).^40–43^ The initial geometries of the fragments were optimised, respectively, at the B3LYP/BS1 level. Electrostatic potential (ESP) atomic charges for the MM region were calculated for each fragment at the same level as the geometry optimisation of the fragments; the ESP atomic charges were fixed while the AFIR search was performed. We considered the dissociative mechanism and the associative mechanism separately. In the dissociative mechanism, the cationic Rh(i)–BINAP complex **X** and the allylic amine **XI** were included in the computational model, and the artificial forces were applied between the fragments (**X** and **XI**). In the associative mechanism, the trimethylamine **XII** was appended to the computational model of the dissociative mechanism, and artificial forces were applied between three fragments (**X**, **XI** and **XII**). The AFIR search was initiated from randomly generated initial orientations and directions of the fragments; this type of AFIR search is called MC-AFIR. The *γ* value for the MC-AFIR search was set to 300 kJ mol^–1^. The path sampling in the MC-AFIR search was terminated when the last ten new AFIR paths resulted in AFIR paths found earlier. All approximate LMs and TSs obtained with the MC-AFIR search were fully optimised without artificial forces at the ONIOM level.

### 2.3 Reaction path search with the SC-AFIR method

After the MC-AFIR search was performed, we applied an SC-AFIR search, which is suitable for finding stepwise paths. The computational level for the SC-AFIR search was the same as for the MC-AFIR search. The initial structures for the SC-AFIR search were selected from the results of the MC-AFIR search; each selected LM was found to be the precursor of the C(1)–H bond cleavage step. All approximate LMs and TSs obtained with SC-AFIR were fully optimised without artificial force at the ONIOM level. After the MC-AFIR and SC-AFIR searches, the connections between the LMs and TSs were verified by intrinsic reaction coordinate (IRC) analysis from each TS.^44^ During the search, the artificial force is applied, and paths are somewhat deviated from actual LMs, TSs and IRCs. We emphasise that the LMs, TSs and IRCs finally obtained and discussed below are “actual” local minima, first-order saddle points, and steepest descent paths in the mass-weighted coordinate, respectively, on the adiabatic potential energy surface. Both AFIR searches were performed with a developmental version of the GRRM programme; the energies, gradients and Hessians were computed with Gaussian 09 (Rev. D01).^31,45^ Technical details of the MC-AFIR and SC-AFIR methods and their recent applications to organic reactions can be found in recent review papers.^46,47^

### 2.4 Representation of the reaction network and extraction of the reaction path

**Fig. 5:**
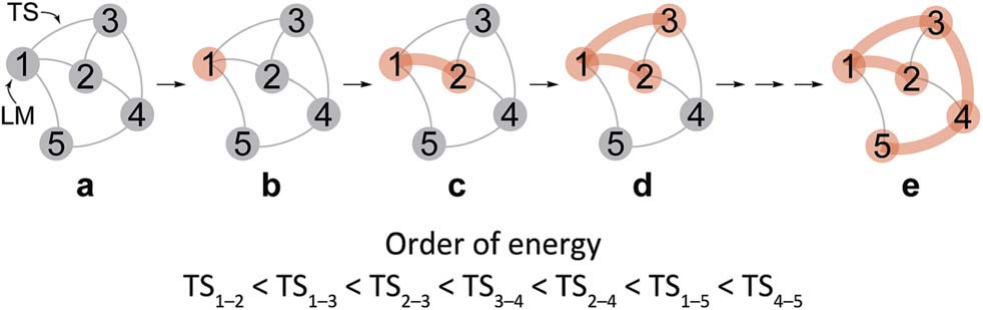
Graph G of the reaction path network and extraction procedures for determining the rational path. The TS between LM (*i*) and LM (*j*) is denoted as TS_*i*–*j*_. Unselected and selected vertices are coloured grey and orange, respectively. All edges denote the reaction path network, and the thick orange edges denote MST.

After the comprehensive search for reaction paths is complete, the remaining problems are to understand the entire reaction network and to select probable paths from the greatly extended reaction network. We showed the reaction network in the form of graph G (Fig. 5). A vertex and an edge represent a LM and a TS, respectively. A number of paths from an arbitrary vertex to another vertex are combinations of the edges. If the numbers of vertices and edges increase, finding probable paths becomes much more difficult because of the exponential growth of the number of combinations. Therefore, we applied Prim’s algorithm to solve the combination problem.^48^ The algorithm accepts a graph G and outputs a tree T by repeating a selection of an edge with a smaller weight. If the energy of the TS is used as the weight of the edge, the algorithm is applicable to obtain a tree T of reaction paths. Because there is a unique path from a starting point to another point in this tree T, the discussion of the reaction paths becomes easy to understand. The concrete procedure is as follows. **1**: select an arbitrary vertex from the graph G, which becomes a starting point of the reaction (*e.g.* vertex 1 is selected in Fig. 5(b)). **2**: select a vertex which has not been selected previously and which is connected to the selected vertex. If there is more than one possible edge, the most stable one is selected (*e.g.* three edges connect to vertex 1 in Fig. 5(c). The most stable edge is 1–2; thus, it will be selected). **3**: select a vertex which has not been selected previously and which is connected to the vertex in the set of selected vertices (Fig. 5(d)). If there is more than one possible edge, the most stable one is selected. **4**: perform step **3** as many times as possible. Finally, by this procedure, we can draw a tree T in which the edge with the highest weight is excluded (Fig. 5(e)). This algorithm is known to provide an MST which is uniquely formed from a network. Thus, any vertex can be selected as the starting point. In the present study, the first vertex was chosen from a vertex with a maximum degree (maximum number of edges connected to the vertex). The numbers of the vertices were arranged in the order of the vertex chosen by Prim’s algorithm. Using this order, the smaller number is given to a vertex which passes through energetically lower TSs from the starting vertex. The Gephi programme was employed to illustrate the graphs.^49^ The Gibbs free energies at the ONIOM(B3LYP/BS1:UFF) level (25 °C) were used for the analysis of the reaction paths.

**Fig. 6:**
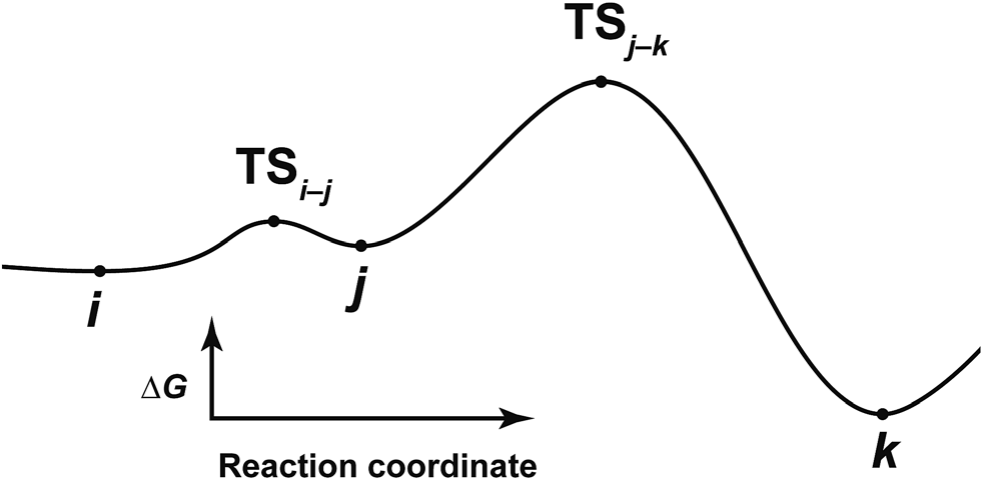
Potential energy curve along the reaction path from *i* to *k*.

Here, the notations of the LM and the TS are explained. The number of the LM is decided according to Prim’s algorithm, and the LM itself is represented by this number (*e.g. i*, *j*, *k* in Fig. 6). The TS which connects *i* and *j* is written as TS_*i*–*j*_. Some reaction steps were discussed together. For example, in Fig. 6, if a reaction path from *i* to *k* is discussed, both LMs and a TS with the highest energy (TS_*j*–*k*_) in the path are focused on. To indicate both ends clearly, notation such as TS_*j*–*k*_ (*i* → *k*) is introduced.

### 2.5 Full DFT optimisation and single point energy calculations

Some LMs and TSs which were selected from the results of the MC-AFIR and the SC-AFIR reaction path searches were reoptimised without the ONIOM model. The dispersion-corrected B3LYP + D3 functional in conjunction with the Stuttgart/Dresden effective core potentials, the associated SDD basis set for a rhodium atom, and the 6-31G(d) basis sets for the other atoms (BS2) were employed for the entire system.^50–52^ After geometry optimisation, IRC calculations were performed to confirm that the TS is connected to the correct LMs.^44^

Single point energy calculations were performed with the same functional as that used for the full DFT geometry optimisation. The BS2 basis sets were replaced with a triple zeta valence polarisation basis set, def2-TZVP (BS3).^53,54^ A polarised continuum model using the integral equation formalism variant (IEF-PCM) with a dielectric constant of *ε* = 7.4257 (THF) was employed to estimate the energetics in solution.^55–57^ Thus, the present computational level is denoted B3LYP + D3(PCM)/BS3//B3LYP + D3/BS2. To analyse the orbital interactions, natural bond orbital (NBO) analysis was performed at the same level.^58^

### 2.6 Energy decomposition analysis

**Fig. 7:**
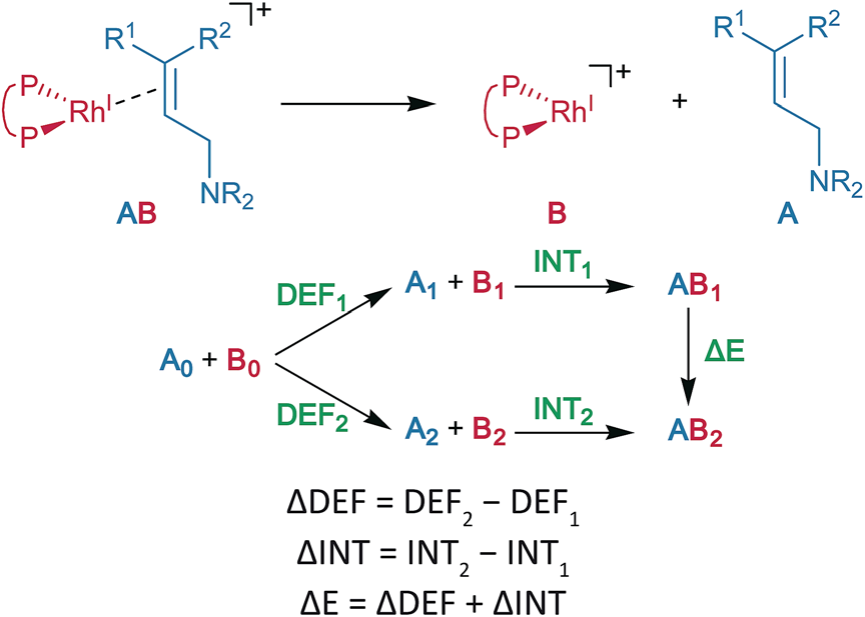
EDA for an allylic amine coordinate cationic rhodium(i)–BINAP complex.

Energy decomposition analysis (EDA) was performed for the key TSs of the stereochemistry-determining step.^59,60^ The B3LYP + D3(PCM)/BS3 level was employed for the EDA. The structures of the rhodium complexes were divided into the allylic amine region (A) and the cationic rhodium(i)–BINAP region (B), as shown in Fig. 7. The deformation energy (DEF) is the sum of the deformation energy of A (DEF_A_, defined as the energy of A at the optimised structure of the complex relative to that of the optimised isolated structure A_0_) and that of B (DEF_B_). INT indicates the interaction energy between A and B at the optimised structure of a complex AB. The energy difference (Δ*E*) between the two optimised structures, AB_1_ and AB_2_, can be written as a sum of the ΔDEF and the ΔINT. The electronic structure calculations described in the last two sections were performed with Gaussian 09 (Rev. D01).^45^

## 3 Results and discussion

### 3.1 Coordination states of the BINAP–rhodium(i)–allylamine complex (results of the MC-AFIR method)

**Fig. 8:**
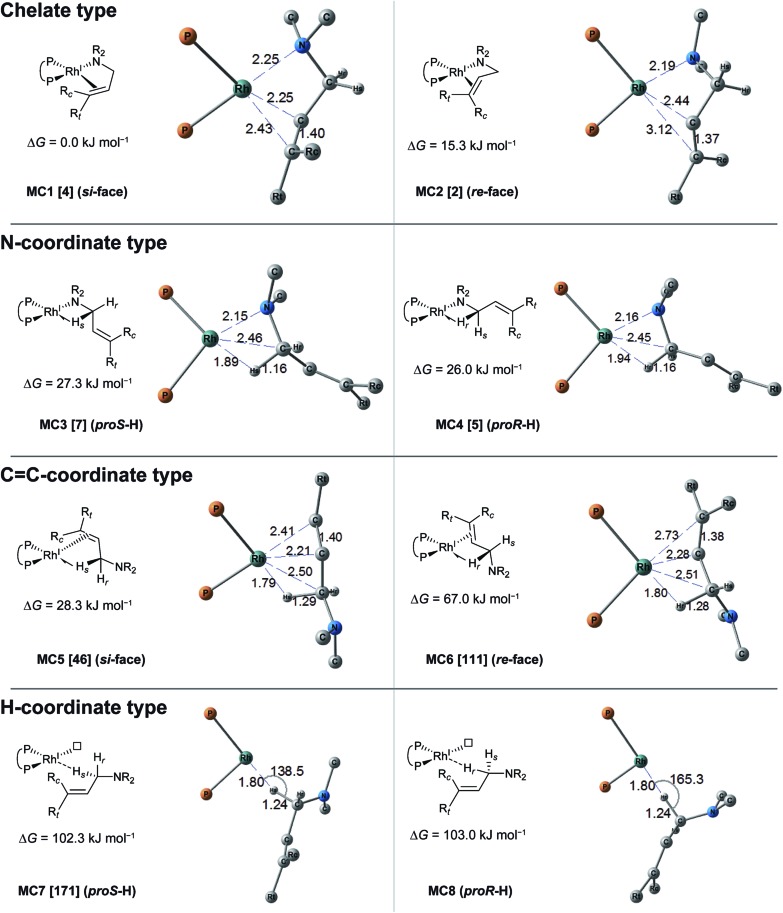
Coordination states of the BINAP–Rh(i)-substrate complex in the dissociative mechanism. The structures and Gibbs energies are calculated at the ONIOM(B3LYP/BS1:UFF) (25 °C) level. Numbers within brackets denote numbers determined by Prim’s algorithm, which are identical to those in graph G_D_.

**Fig. 9:**
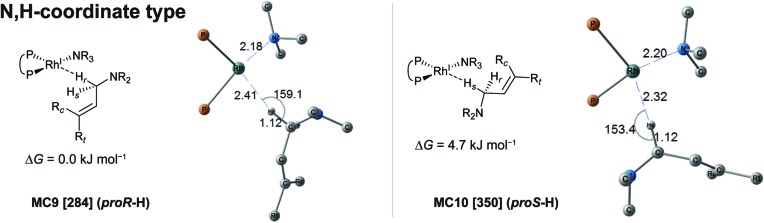
Coordination states of the BINAP–Rh(i)-substrate complex in the associative mechanism. The structures and Gibbs energies are calculated at the ONIOM(B3LYP/BS1:UFF) (25 °C) level. Numbers within brackets denote numbers determined by Prim’s algorithm, which are identical to those in graph G_A_.

The MC-AFIR coordination searches for the dissociative mechanism provided four types with two diastereomers in each type (Fig. 8); the searches for the associative mechanism provided one type with two diastereomers (Fig. 9).

In the dissociative mechanism, the chelate complex **MC1** is the most energetically stable LM. The C=C double bond of the substrate in **MC1** coordinates to the Rh(i) centre from the *si*-face. Another chelate complex, **MC2** (Δ*G* = 15.3 kJ mol^–1^), whose C=C double bond is coordinated from the *re*-face, is an energetically higher LM than **MC1** (Δ*G* = 0.0 kJ mol^–1^). Next, the stable LMs are classified into N-coordinate types (**MC3** and **MC4**); these have a Rh(i)–N coordinated bond and a Rh(i)···H–C(1) interaction. The coordination mode between the Rh(i) centre and the H–C(1) σ-bond forms a side-on geometry, and the H–C bond distance (1.16 Å) is longer than the general H–C bond distance (1.10 Å), showing a typical agostic interaction.^61^ The energy difference between the complexes **MC3** and **MC4** is very small (Δ*G* = 1.3 kJ mol^–1^). The next type is a C=C-coordinated type. C=C-coordination from the *si*-face and *re*-face directions forms the C=C-coordinate Rh(i) complexes **MC5** and **MC6**, respectively. Both complexes also have agostic interactions. The π-coordination bond of C(2)=C(3) is inclined toward the C(2) atom because the agostic interaction attracts the Rh(i) atom to the H–C(1) σ-bond. There is a large energy difference between **MC5** and **MC6** (Δ*G* = 38.7 kJ mol^–1^), which will be discussed in Section 3.3.4. The last type of complex has a Rh(i)···H–C(1) interaction and a vacant coordination position on the Rh(i) atom (**MC7** and **MC8**). The rhodium atom of **MC7** interacts with the *pro-S*-hydrogen atom, while that of **MC8** interacts with the *pro-R*-hydrogen atom. The Rh(i)···H–C(1) of both complexes is linear. Thus, this interaction is classified as an anagostic interaction.^61^ The four agostic complexes (**MC3**, **MC4**, **MC5** and **MC6**) were selected as the initial structures for the SC-AFIR reaction path searches of the dissociative mechanism.

In the associative mechanism, the results of the MC-AFIR search include only N,H-coordinate complexes **MC9** and **MC10** (Fig. 9); these have a Rh(i)–N coordination bond and a Rh(i)···H–C(1) interaction. The Rh(i)···H–C(1) interaction is classified as an anagostic interaction, similar to **MC7** and **MC8**. No bisamine coordinate Rh(i)–BINAP complex like complex **II**, which was identified using SC-AFIR, was found.

Further results for the coordination state of the bis-substrate coordinate Rh(i)–BINAP complex will be discussed in Section 3.4. Both of the N,H-coordinate complexes (**MC9** and **MC10**) were selected as initial structures for the SC-AFIR reaction path searches of the associative mechanism.

### 3.2 Reaction networks (results of the SC-AFIR method)

**Fig. 10:**
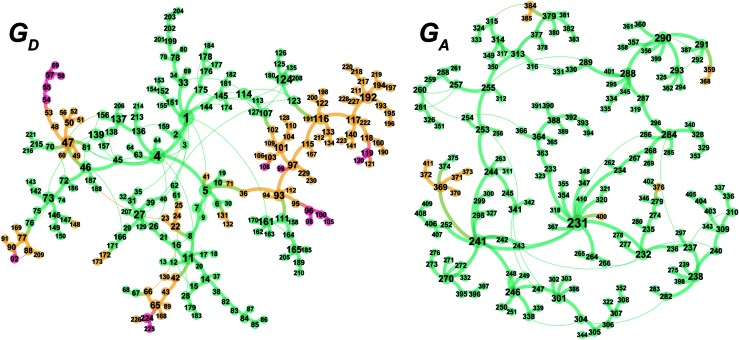
Graphs G_D_ (left) and G_A_ (right). The ‘reactant’, ‘hydride’ and ‘product’ complexes are coloured green, yellow and red, respectively. The classes of the complexes are defined as follows. The ‘reactant’ complex has a geometry with two C(1)–H bonds in the substrate. The ‘hydride’ complex is not classified as either the reactant or the product class. The ‘product’ complex has a geometry with one C(3)–H bond in the substrate. The presence or absence of the C–H bond is determined by whether the length of the bond is less than 1.3 Å. The bold line denotes the MST in the network.

The MC-AFIR and SC-AFIR reaction path searches for dissociative and associative mechanisms gave two reaction path networks, which are shown as graphs G_D_ and G_A_ in Fig. 10. The subscripts ‘D’ and ‘A’ denote dissociative and associative mechanisms, respectively. The former consists of 361 LMs and 344 TSs, while the latter consists of 257 LMs and 257 TSs.

To discuss the networks, all LMs and TSs are classified into the following three classes: **C1**: a ‘reactant’ complex, which has two C(1)–H bonds in the substrate (green in Fig. 10); **C2**: a ‘hydride’ complex, which is classified as neither a reactant nor a product complex (yellow in Fig. 10); **C3**: a ‘product’ complex, which has one C(3)–H bond in the substrate (red in Fig. 10). The longest C–H bond distance was set as 1.3 Å.

### 3.3 Dissociative mechanism (G_D_)

**Fig. 11:**
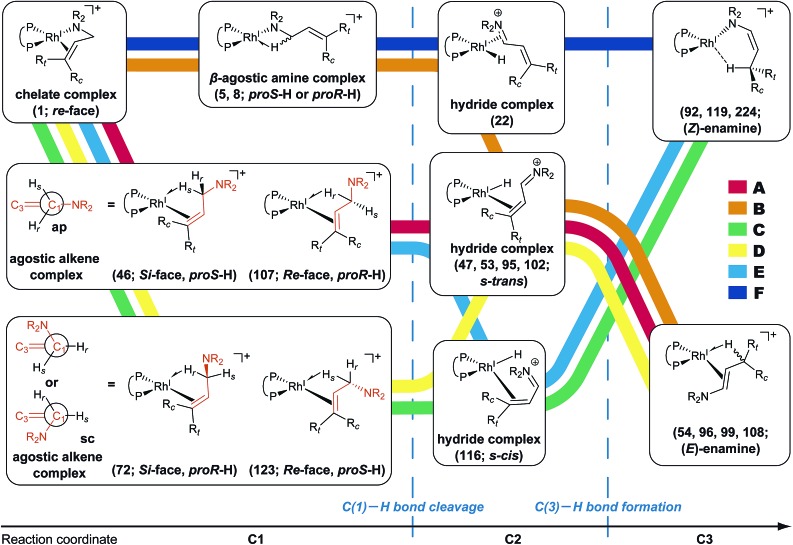
Possible paths of the cationic Rh(i)–BINAP-catalysed isomerisation of allylic amines.

According to the present rules, the chelate complex **1** is the starting point in the dissociative mechanism (Fig. 11). A nitrogen atom and a C=C double bond from the *re*-face direction are coordinated to a Rh(i) atom in complex **1**. All of the paths can be classified into six types (A–F in Fig. 11) by their combinations of intermediates. The paths from complex **1** to the product (**C3**) are summarised in Table 1.

**Table 1:** Paths to the product complex. The LM denotes the end point of the path. *E*/*Z* and *S*/*R* denote the stereochemistry of the product

LM (**C3**)	*E*/*Z*	*S*/*R*	Possible paths	Path on MST
**54**	*E*	*R*	A, B	A
**92**	*Z*	*R*	C	C
**96**	*E*	*S*	A, B, D	B
**99**	*E*	*S*	A, B, D	B
**108**	*E*	*S*	A, B, D	B
**119**	*Z*	*S*	C, E	E
**224**	*Z*	*S*	F	F

#### 3.3.1 Path to **54** (the most favourable reaction path)

**Fig. 12:**
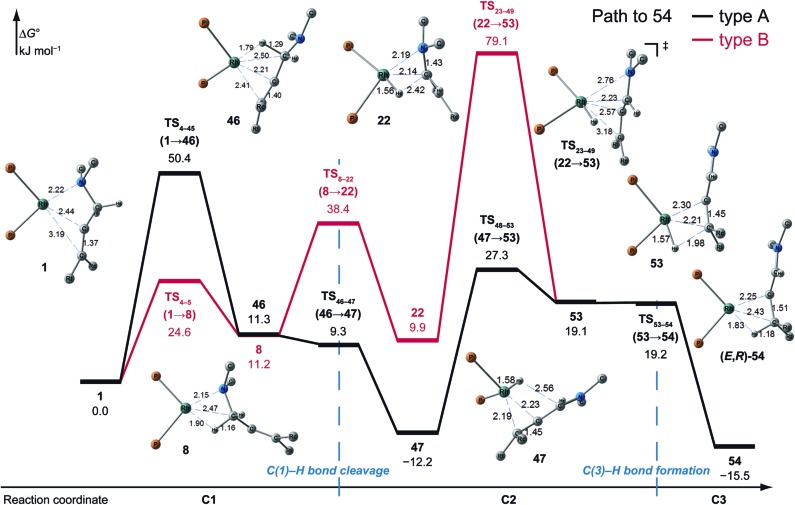
The Gibbs free energy profile along the path to **54** (type A and B) and the molecular structures on the path, calculated at the ONIOM(B3LYP/BS1:UFF) level (25 °C). The energies are given relative to **1** in kJ mol^–1^.

A path to **54** (type A) is the most favourable reaction path and forms an (*E*,*R*)-enamine; this stereochemistry is consistent with the experimental results. The path to **54** starts from the chelate complex **1**; complex **1** is converted to the C=C-coordinate complex **46**, which experiences an agostic interaction between the Rh(i) atom and the H_S_–C(1) bond. The energy profile is shown in Fig. 12. The Rh-bound H atom (H_S_) migrates to the Rh(i) atom as a hydride with the assistance of an electron supplied from the N atom; this results in the formation of an iminium ion structure (**47**). Importantly, the C(1)=N double bond of the iminium ion moiety is not bound to the Rh(i) atom, in contrast to the η^2^-coordination in the mechanism proposed by Noyori *et al.* (**IV** and **VII** in Fig. 2). Then, a conformational change through rotation around the π-coordination axis forms another C=C-coordination Rh(i) hydride complex **53**, which is the rate limiting step of path A to **54** (Δ*G* = 27.3 kJ mol^–1^). Finally, the (*E*,*R*)-enamine coordinate complex **54** is formed through hydride migration.

One path to **54** (type B) is consistent with the mechanism of Noyori *et al.* In this path, the Rh(i)-bound iminium ion **IV** in Fig. 2 is formed through hydride migration from the N-coordinate Rh(i) complex **III**. The path to **54** (type B) forms the Rh(i)-bound iminium ion **22** through hydride migration from the N-coordinate Rh(i) complex **8**. Then, intramolecular coordination switching occurs (TS_23–49_ (**22** → **53**)), when the iminium ion of the substrate is eliminated from the Rh(i) centre and the C=C double bond of the substrate coordinates to the Rh(i) centre to form the C=C-coordinate Rh(i) hydride complex **53**. Because the distance between the iminium ion and the Rh(i) centre is longer than that of the other intermediates, the energy of TS_23–49_ (**22** → **53**) is higher (Δ*G* = 79.1 kJ mol^–1^) than that of the other LMs and TSs. As a result, the type B path is disadvantageous compared with the type A path.

#### 3.3.2 Path to **92**, **119** and **224** (types C, E and F; formation of (*Z*)-enamine)

**Fig. 13:**
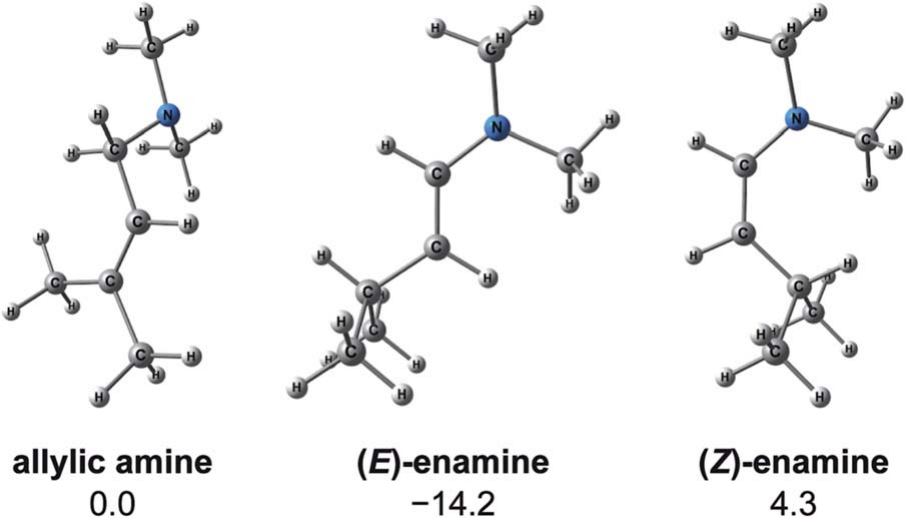
Molecular structures and energies of the allylic amine (left), (*E*)-enamine (centre) and (*Z*)-enamine (right). Energies are calculated at the B3LYP + D3(PCM)/BS3//B3LYP + D3/BS2 (60 °C) level in kJ mol^–1^. The most stable conformer is shown for each geometry.

A path to **92** (type C) forms the (*Z*,*R*)-enamine. As a result of the reaction path search, an early number was assigned to the product of the path of type C. However, the (*Z*)-enamine was not obtained in the experiment. These contradictory results are explained by the thermodynamic stabilities of the products (Fig. 13). Because the transformation from an allylic amine to a (*Z*)-enamine is an endergonic process (Δ*G* = 4.3 kJ mol^–1^), the type C path can be ruled out. Both the path to **119** (type E) and the path to **224** (type F), which form (*Z*)-enamines, can also be ruled out for the same reason.

#### 3.3.3 Path to **96** (formation of (*E*,*S*)-enamine)

**Fig. 14:**
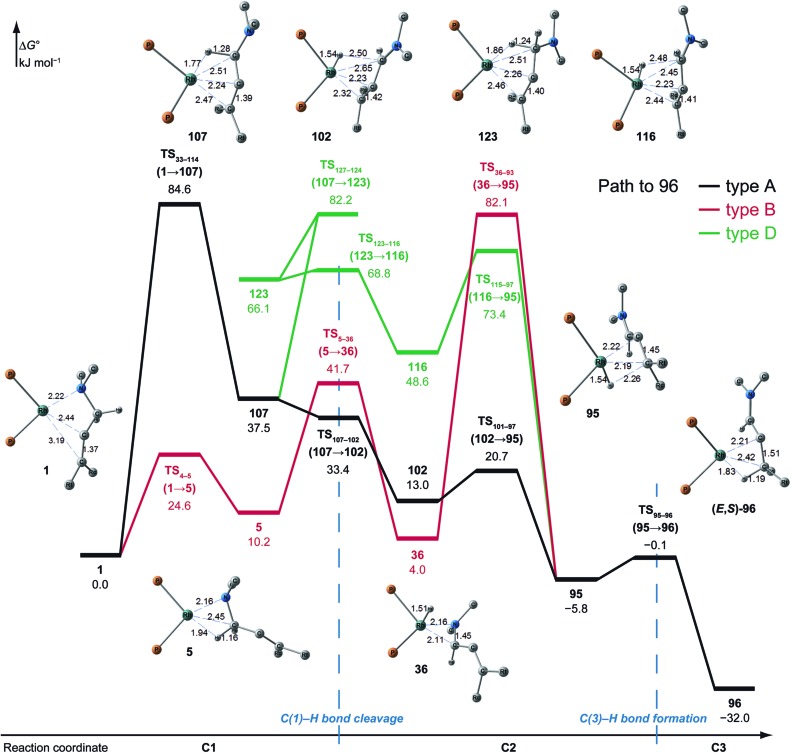
The Gibbs free energy profile along the path to **96** (types A, B and D) and molecular structures on the path, calculated at the ONIOM(B3LYP/BS1:UFF) level (25 °C). The energies are given relative to **1** in kJ mol^–1^.

Paths to **96**, **99** and **108** give the (*E*,*S*)-enamine, which is the opposite enantiomer of **54** ((*E*,*R*)-enamine). The energy profile is shown in Fig. 14. These paths share the path from **1** to hydride complex **93** and then branch off to each destination. Because the differences mainly arise from a small conformational change in the BINAP ligand and because the paths are similar, the paths to **96** are discussed as a representative. There are three paths (A, B and D) to **96**. The path to **96** (type A) is similar to the path to **54** (type A) except for the unstable TS_33–144_ for the change of coordination mode (**1** → **107**; Δ*G* = 84.6 kJ mol^–1^). The path to **96** (type B) is almost the same as the path to **54** (type B). The path of type D passes an s-*cis*-iminium ion coordinate Rh(i) complex **116** (Δ*G* = 48.6 kJ mol^–1^), which is much higher in energy compared with the s-*trans* isomer **102** (Δ*G* = 13.0 kJ mol^–1^); thus, the type D path is not preferred.

#### 3.3.4 *R*/*S* selection

**Fig. 15:**
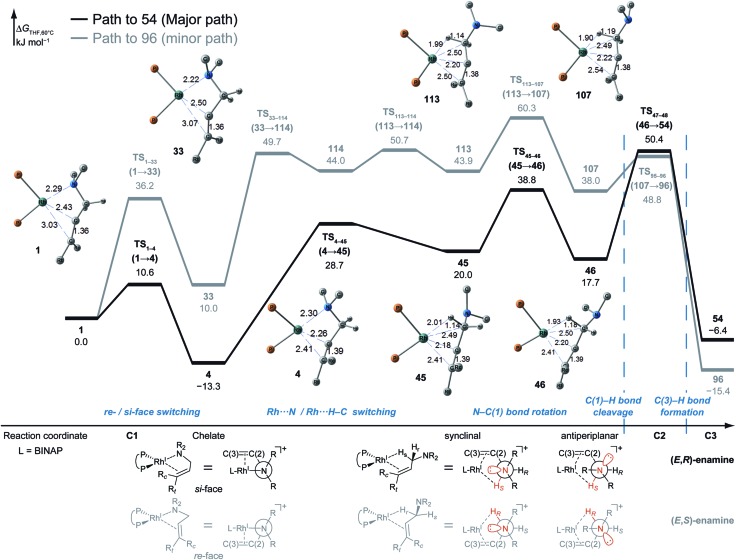
The Gibbs free energy profile along the path to **54** and the path to **96** (type A) and the molecular structures on the paths, calculated at the B3LYP + D3(PCM)/BS3//B3LYP + D3/BS2 level (60 °C). The energies are given relative to **1** in kJ mol^–1^.

The type A path is the preferred path to **54**; it affords the (*E*,*R*)-enamine (the major product in the experiment) and is the most reasonable path. The path to **96** (type A) forms the (*E*,*S*)-enamine, which is a minor product in the experiment, and passes through comparably unstable LMs and TSs. The difference between the two type A paths determines the *R*/*S* selectivity of the product; this difference between the path from **1** to **46** (Fig. 12) and the path from **1** to **107** (Fig. 14) is remarkable. Both paths (type A) were compared at higher computational levels (Fig. 15).

The difference between the paths (type A) can be observed from the chelate complex. The chelate complex **33** (Δ*G* = 10.0 kJ mol^–1^), in which the C=C double bond is coordinated to Rh(i) from the *re*-face, is higher in energy than another chelate complex, **4** (Δ*G* = –13.3 kJ mol^–1^), in which the C=C double bond is coordinated from the opposite face. The reactions on both paths A (**1** → **46** and **1** → **107**) proceed while maintaining the Rh(i)···C=C bond and the energy difference. Accordingly, the *R*/*S* selectivity of the product depends on the coordination state of the enantiotopic face of the allyl group.

**Fig. 16:**
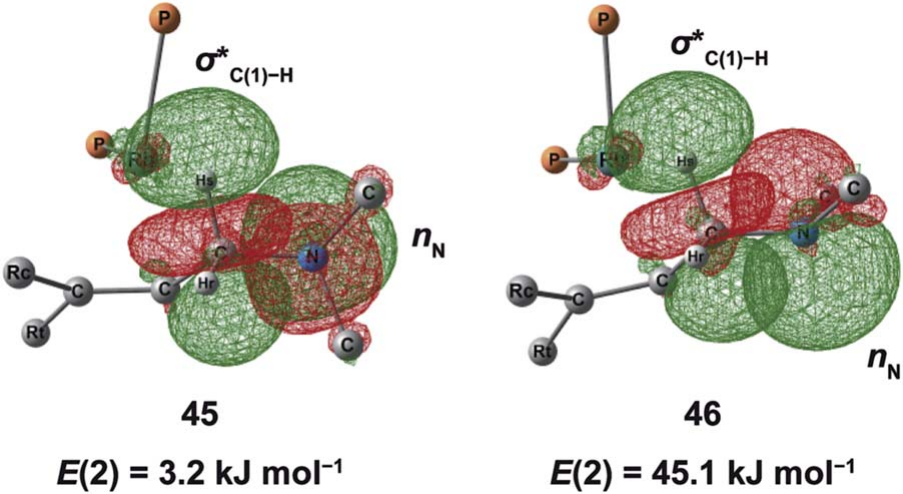
NBO analysis of the non-bonding orbital of the nitrogen atom (*n*_N_) and the antibonding orbital of the C(1)–H bond 
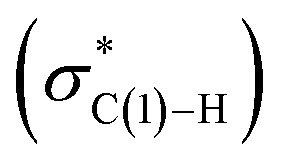
. *E*(2) refers to the second-order perturbation energy for the donor–acceptor interaction.

N–C(1) bond rotations (**45** → **46** and **113** → **107**) occur on the type A path; this is a significantly high energy process. The rotation connects a C=C-coordinate Rh(i) complex (**45** or **113**) with the synclinal (sc) conformation of the dihedral angle H–C(1)–N–lp (lone pair) to a complex with an antiperiplanar (ap) conformation (**46** or **107**). The sc conformation must form immediately after the Rh(i)···N bond cleavage. In contrast, the ap conformation is advantageous from the viewpoint of the stereoelectronic effects between the non-bonding orbital of the nitrogen (*n*_N_) and the C(1)–H antibonding orbital 
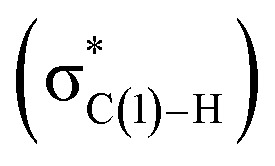
. The orbital overlap can be confirmed by NBO analysis, and the strength of the donor–acceptor interaction between the *n*_N_ and the 
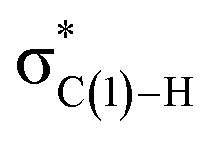
 orbitals can be confirmed by the second-order perturbation energy *E*(2) (Fig. 16). As the donor–acceptor interaction increases in strength (**45**: *E*(2) = 3.2 kJ mol^–1^ → **46**: *E*(2) = 45.1 kJ mol^–1^), the C(1)–H bond length increases slightly (**45**: 1.14 Å → **46**: 1.18 Å). In other words, the interaction weakens the C(1)–H bond. This factor is important for C(1)–H activation.

**Fig. 17:**
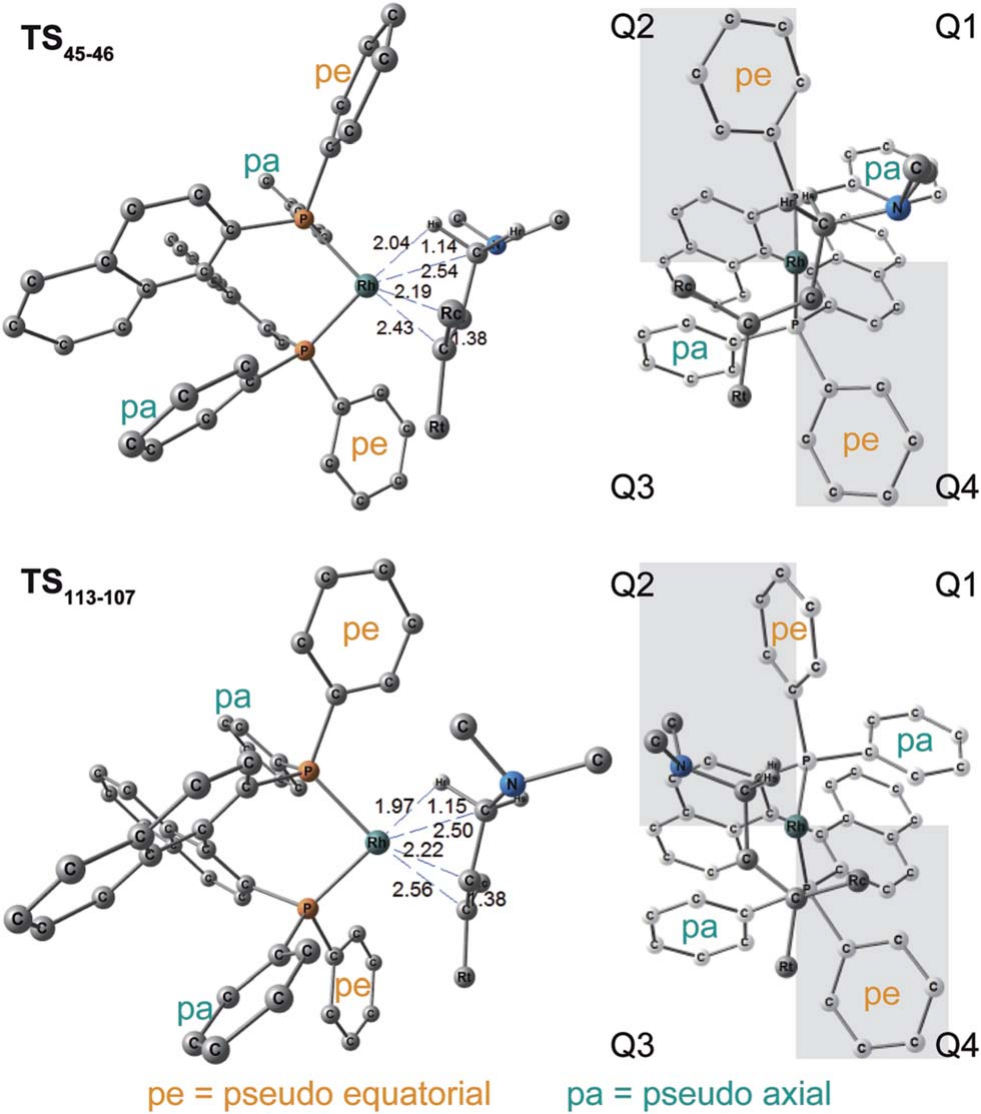
Molecular structures and quadrant models of TSs on the N–C bond rotation step. TS_45–46_ (*si*-face) is shown on the top and TS_113–107_ (*re*-face) is shown on the bottom. The structures are optimised at the B3LYP + D3(PCM)/BS3//B3LYP + D3/BS2 level.

The N–C(1) bond rotation is a more energy-demanding step in the paths from **1** to **46** and from **1** to **107** (Fig. 15). Comparing the respective TSs of the N–C(1) bond rotation, the BINAP ligand at TS_45–46_ (the *si*-face) maintains *C*_2_-symmetry very well, while that for TS_113–107_ (the *re*-face) is largely distorted, especially for two of the pseudo-equatorial phenyl groups (Fig. 17). This is because the dialkylamino group and the methyl group on the C(3) atom in TS_113–107_ (*re*-face) occupy the upper left space (second quadrant) and the lower right (fourth quadrant) (Fig. 17), which was originally occupied by the pseudo-equatorial phenyl groups of the BINAP ligand, to avoid steric repulsion between the substrate and the phenyl group; therefore, the BINAP ligand is largely distorted. We performed EDA to investigate the energy difference between TS_45–46_ and TS_113–107_ (Table 2). The energy difference (ΔΔ*E* = 19.1 kJ mol^–1^) between these TSs is consistent with the deformation energy difference (ΔDEF = 16.9 kJ mol^–1^) and the interaction energy difference (ΔINT = 2.2 kJ mol^–1^). The contributions of the ΔΔ*E* mainly originate from the DEF (88.9%), and the contribution of INT is small (11.1%). Furthermore, the difference in deformation energy in the allylamine moiety (ΔDEF_A_ = –0.1 kJ mol^–1^) is negligible, and that of the BINAP–Rh(i) moiety (ΔDEF_B_ = 17.0 kJ mol^–1^) occupies the majority of the ΔDEF.

**Table 2:** EDA for the energy difference between two TSs on the N–C bond rotation step in kJ mol^–1^. Energies are calculated at the B3LYP(PCM) + D3/BS3//B3LYP + D3/BS2 level

	DEF (DEF_A_, DEF_B_)	INT	Δ*E*	Δ*G*
TS_45–46_ (*si*-face)	52.5 (39.5, 13.0)	–132.9	51.6	17.7
TS_113–107_ (*re*-face)	69.4 (39.3, 30.0)	–130.7	70.7	38.0
*Δ*	16.9 (–0.1, 17.0)	2.2	19.1	21.5

### 3.4 Associative mechanism

**Fig. 18:**
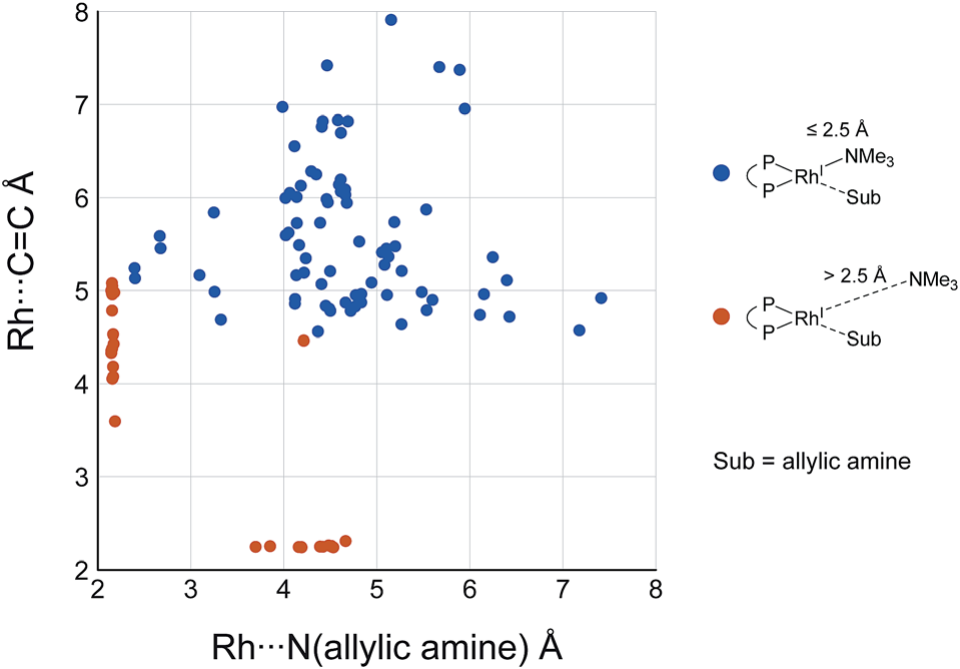
Scatter plot of Rh(i)–N distances *versus* distances between the Rh(i) atom and the centre of the C(2)=C(3) bond of each complex in the associative mechanism. The molecular structures are calculated at the ONIOM(B3LYP/BS1:UFF) level.

**Fig. 19:**
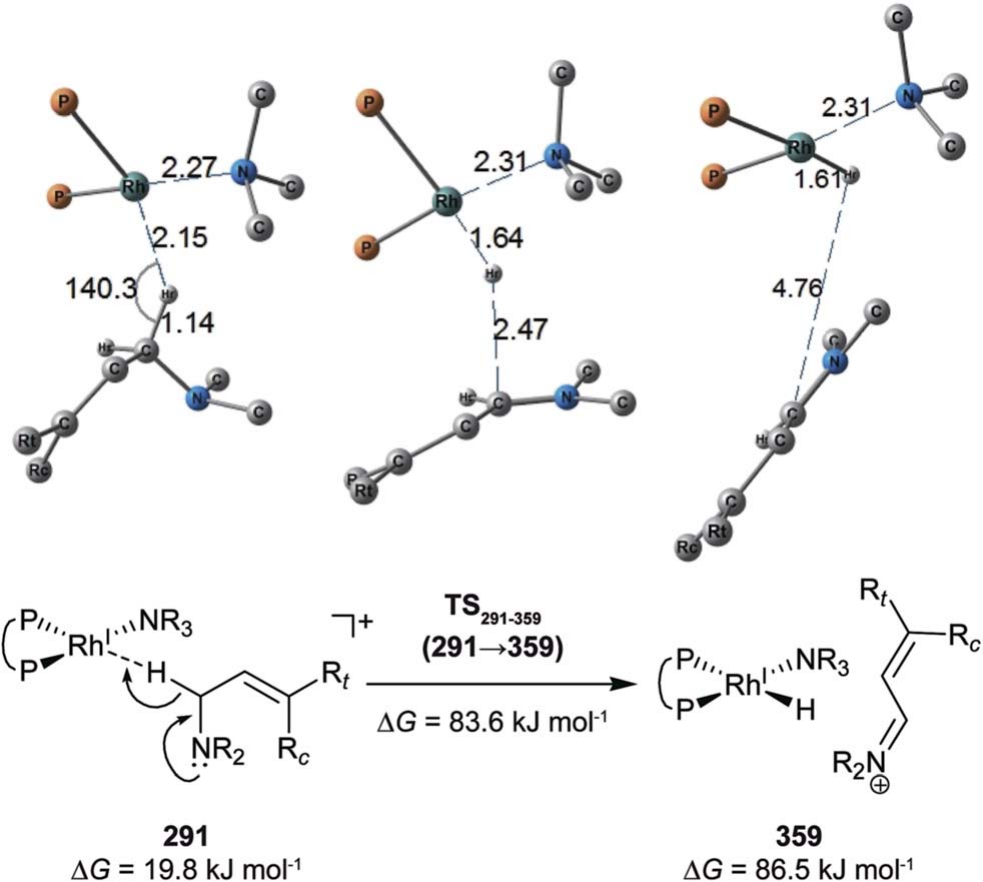
C(1)–H bond cleavage reaction in the associative mechanism. The Gibbs free energies are relative to the sum of the energies of complex **1** and the TMA at the B3LYP + D3(PCM)/BS3//B3LYP + D3/BS2 level. The temperature was set to 60 °C.

**Fig. 20:**
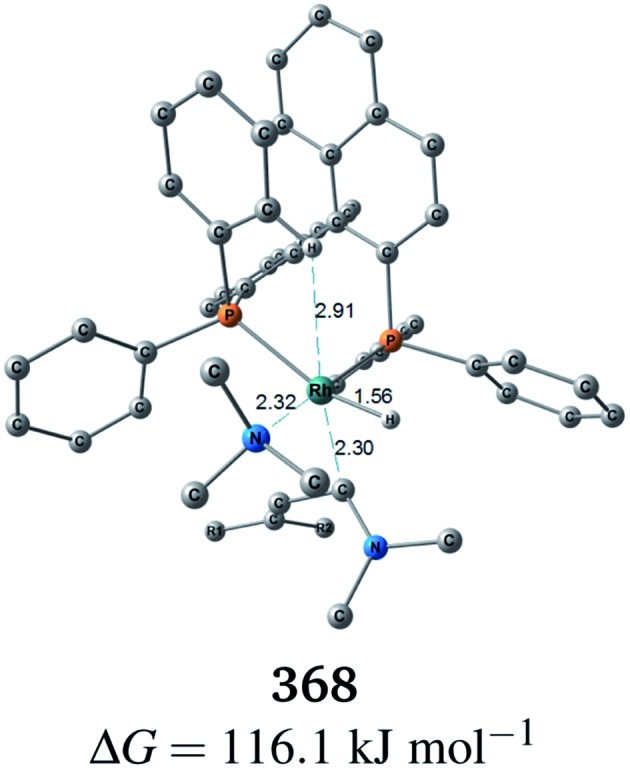
Molecular structure of **368**. The Gibbs free energy is relative to the sum of the energies of complex **1** and the TMA, and is based on the B3LYP + D3(PCM)/BS3//B3LYP + D3/BS2 level. The temperature was set to 60 °C.

While some product complexes (red vertex in Fig. 10) are present in the network of the dissociative mechanism, there is no product complex in the network of the associative mechanism. To confirm the coordination state of all of the LMs, the Rh(i)–N (allylic amine) distance and the distance between the Rh(i) atom and the centre of the C(2)=C(3) bond are summarised in the scatter plot shown in Fig. 18, in which the LMs are divided into two groups by the Rh(i)–N (TMA) distance. Some C=C-coordinate Rh(i) complexes have no TMA coordination (orange plot in Fig. 18); however, no C=C-coordinate Rh(i) complex is formed with TMA coordination (blue plot in Fig. 18). This result is different from the allylic mechanism of Ujaque and Espinet *et al.* ^18^ (to find a C=C-coordinate Rh(i) complex, 647 approximate TSs were searched with the MC-AFIR and SC-AFIR methods for the associative mechanism). In the previous study by Ujaque and Espinet *et al.*, the models that they used to conclude that the associative mechanism and allylic mechanism are favourable were too small (*e.g.* they used two PH_3_ molecules as a model of the BINAP ligand). However, BINAP is a bulky ligand; thus, the coordination of the comparably bulky C=C bond to the Rh(i) centre is impossible in the associative mechanism. There are eight paths of C(1)–H bond cleavage in the graph G_A_ (**231** → **400**, **241** → **369**, **279** → **376**, **291** → **359**, **315** → **384**, **374** → **369**, **379** → **384** and **402** → **376**). The most preferred path among the eight paths starts from the N-coordinate Rh(i) complex **291** (Fig. 19). After **291** is formed, a hydride on C(1) is abstracted by the Rh(i) centre, and the complex is separated into the N-hydride-coordinate Rh(i) and iminium ion fragments **359** (Fig. 19). The eight C(1)–H cleavage paths shown above proceed similarly. The hydride complex **368** has the largest coordination number (five-coordinate Rh(iii) complex) in the entire network, which is formed from **359** through Rh(i)–C(1) bond formation. **368** has a square pyramidal geometry, as shown in Fig. 20.

In all of the hydride complexes except for complex **368**, the substrate is located far from the Rh(i) centre (see the ESI, Section 2.1†). Because the vacant position of complex **368** is blocked by a phenyl group of the BINAP ligand, the formation of the six-coordinate distorted-octahedral complex proposed by Takaya and Noyori *et al.* ^17^ and Ujaque and Espinet *et al.* ^18^ appears to be impossible. Furthermore, the relative free energy of complex **368** (Δ*G* = 116.1 kJ mol^–1^) is energetically unstable. Accordingly, the associative mechanisms of Takaya and Noyori *et al.* and Ujaque and Espinet *et al.* are unfavorable.

## 4 Conclusions

**Fig. 21:**
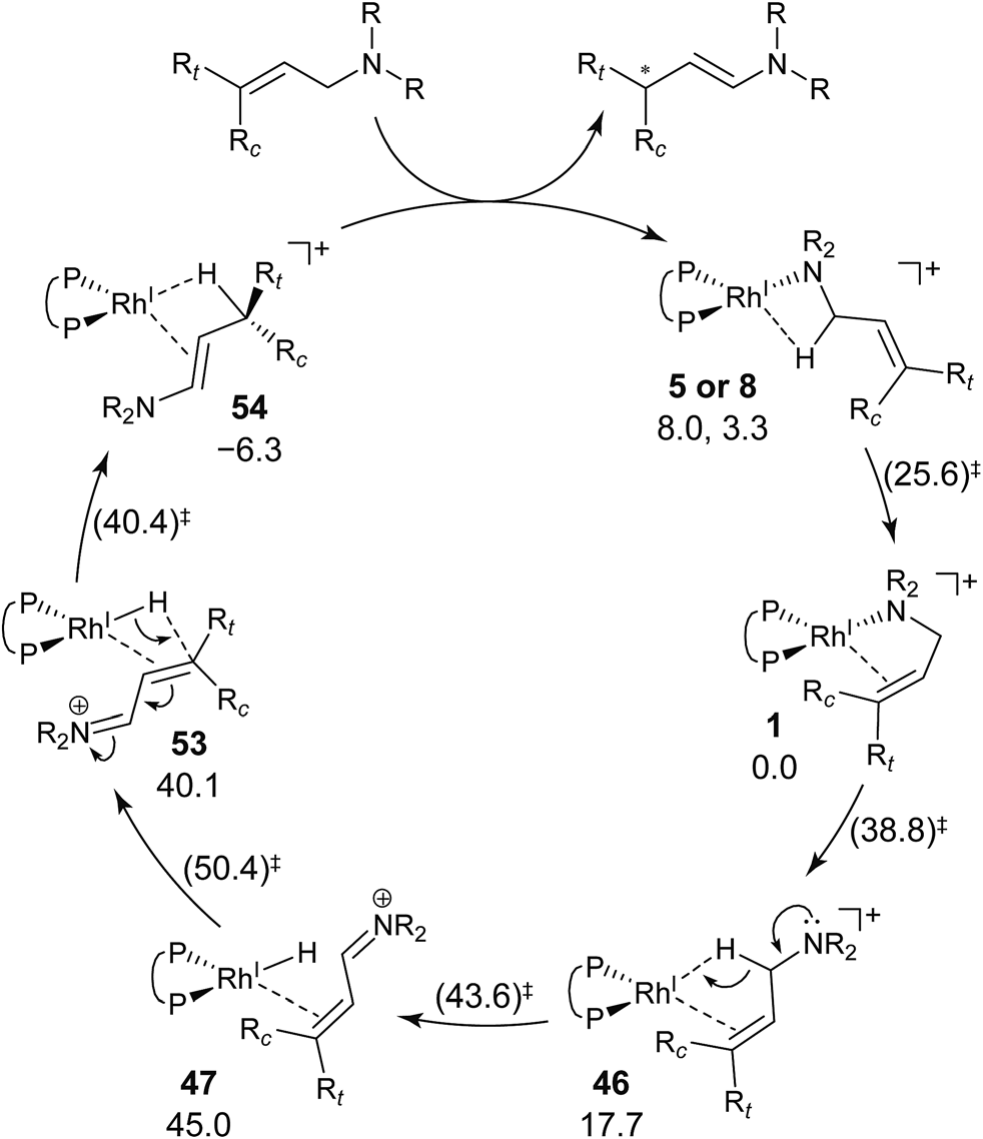
Catalytic cycle of the cationic Rh(i)–BINAP-catalysed isomerisation of allylic amines, as determined by a comprehensive reaction path search without assumption of TSs using the MC-AFIR and SC-AFIR methods and the selection of a rational reaction path using Prim’s algorithm. The Gibbs free energies are denoted with and without parentheses, calculated at the B3LYP + D3(PCM)/BS3//B3LYP + D3/BS2 level (60 °C). The energies are given relative to **1** in kJ mol^–1^.

We explored the reaction paths of the cationic Rh(i)–BINAP-catalysed isomerisation of allylic amines by employing the MC-AFIR and SC-AFIR methods combined with the ONIOM method; furthermore, we determined the most rational path from the complicated reaction network by employing Prim’s algorithm. We conclude that the associative mechanism is impossible because there is no path leading to the product complex in the associative mechanism; also, the intermediates in the associative mechanism are unstable compared with those in the dissociative mechanism. In the dissociative mechanism, a path to **54** (type A) is the most rational path; it affords the (*E*,*R*)-enamine, which is consistent with the experimental results. Some paths afford the (*Z*)-enamine. However, the path to the (*Z*)-enamine is an endergonic process; therefore, the (*Z*)-enamine is not formed. The path to **96** leads to the opposite enantiomer of **54**. The C=C-coordinate Rh(i) complexes on the path to **54** (*e.g.* **45**: Δ*G* = 20.0 kJ mol^–1^) are comparably stable intermediates. In contrast, those on the path to **96** (*e.g.* **113**: Δ*G* = 43.9 kJ mol^–1^) are higher in energy. The difference arises from the steric repulsion between a pseudo-equatorial phenyl group and a substrate; the phenyl group on the dialkylamino group side is especially distorted. The path to **96** is not preferred because of the instability of the C=C-coordinated intermediates. The catalytic cycle is summarised in Fig. 21. The cycle starts from the chelate complex **1** or from the N-coordinate complex **5** or **8**. Complex **1** is converted to a C=C-coordinate complex **46**, which has an agostic interaction between the Rh(i) atom and the H–C(1) bond. In **46**, the C=C double bond is coordinated to the Rh(i) atom from the *si*-face direction. Then, the hydride ion on the C(1) atom migrates to the Rh(i) atom and the hydride complex **47** is formed, in which the lone pair of the nitrogen atom assists hydride migration. Complex **53** is a conformational isomer of **47**. Finally, hydride migration from the Rh(i) atom to the C(3) atom forms the product complex **54** ((*E*,*R*)-enamine). After the formation of **54**, the catalytic cycle moves to the next cycle through substitution.

The aim of the present study is to demonstrate the possibility of determining a reaction mechanism without assumption of TSs. We achieved this using the MC-AFIR and SC-AFIR methods. Furthermore, the *E*/*Z* and *S*/*R* selectivity of the product could be explained. In addition, we demonstrated the usefulness of Prim’s algorithm to find a rational combination of elementary reaction steps. We hope the present study demonstrates the successful application of the AFIR method and Prim’s algorithm.
