# Supplementary material for: Exploring the full catalytic cycle of rhodium(i)–BINAP-catalysed isomerisation of allylic amines: a graph theory approach for path optimisation
Source: Chem Sci. 2017 May 3;8(6):4475–88. doi: 10.1039/c7sc00401j (PMC5618337; doi:10.1039/c7sc00401j)
Supplement: Supplementary file 1 [file SC-008-C7SC00401J-s001.pdf]

Electronic Supplementary Material (ESI) for Chemical Science.
This journal is © The Royal Society of Chemistry 2017

Exploring the Full Catalytic Cycle of Rhodium(I)-BINAP-Catalysed Isomerisation of Allylic Amines: A Graph Theory Approach for Path Optimisation

Takayoshi Yoshimura, Satoshi Maeda*, Tetsuya Taketsugu,
Masaya Sawamura, Keiji Morokuma* and Seiji Mori*

Contents

1 Absolute energies of TSs and LMs **2**

2 Molecular structures **5**
2.1 Hydride complexes in the associative mechanism (ONIOM(B3LYP/BS1:UFF) level) 5

3 Cartesian coordinates **6**
3.1 Dissociative mechanism (ONIOM(B3LYP/BS1:UFF) level) 6
3.2 Associative mechanism (ONIOM(B3LYP/BS1:UFF) level) 16
3.3 Simple molecules (B3LYP+D3/BS2 level) 20
3.4 Dissociative mechanism (B3LYP+D3/BS2 level) 20
3.5 Associative mechanism (B3LYP+D3/BS2 level) 27

1 Absolute energies of TSs and LMs

Table S1: The electronic and Gibbs free energies (60 °C) of simple molecules[a]

Name	E	G
allylic amine	-330.647486	-330.471406
(E)-enamine	-330.653916	-330.476816
(Z)-enamine	-330.647281	-330.469767
TMA	-174.552133	-174.455988
BINAP-Rh(I)	-2489.986682	-2489.438835

[a]The energies are calculated at the B3LYP+D3(PCM)/BS3//B3LYP+D3/BS2 level. The energies are given in a.u.

Table S2: The electronic and Gibbs free energies of LMs and TSs in the dissociative mechanism

No.	$E_{\text{ONIOM}}{}^{a}$	$G_{\text{ONIOM}}{}^{a}$	E^{b}	G^{b}
1	-1125.730772	-1124.913096	-2820.684453	-2819.931331
4	-1125.740405	-1124.919239	-2820.691181	-2819.936397
5	-1125.720529	-1124.909197	-2820.674481	-2819.928267
8	-1125.720719	-1124.908826	-2820.677211	-2819.930078
22	-1125.723900	-1124.909316	-	-
33	-1125.721588	-1124.902540	-2820.679019	-2819.927520
36	-1125.721164	-1124.911588	-2820.663364	-2819.921637
45	-1125.712512	-1124.900701	-2820.669576	-2819.923703
46	-1125.720351	-1124.908783	-2820.672001	-2819.924606
47	-1125.730769	-1124.917743	-2820.660056	-2819.914195
53	-1125.715590	-1124.905836	-2820.660548	-2819.916040
54	-1125.731889	-1124.919010	-2820.681749	-2819.933746
95	-1125.727547	-1124.915314	-2820.659911	-2819.914335
96	-1125.737528	-1124.925292	-2820.684304	-2819.937191
102	-1125.720668	-1124.908160	-2820.658028	-2819.914839
107	-1125.712478	-1124.898816	-2820.664969	-2819.916859
113	-1125.703807	-1124.890748	-2820.661987	-2819.914623
114	-1125.712653	-1124.898140	-2820.662071	-2819.914568
116	-1125.707573	-1124.894602	-	-
123	-1125.700956	-1124.887932	-	-
TS_{1-4}	-1125.724434	-1124.908375	-2820.676824	-2819.927279
TS_{1-33}	-1125.708539	-1124.887994	-2820.668845	-2819.917529
TS_{4-5}	-1125.716807	-1124.903738	-	-
TS_{4-45}	-1125.707529	-1124.893881	-2820.667743	-2819.920404
TS_{5-36}	-1125.705762	-1124.897195	-	-
TS_{8-22}	-1125.707998	-1124.898483	-	-
TS_{23-49}	-1125.694792	-1124.882959	-	-
TS_{33-114}	-1125.697630	-1124.880858	-2820.661644	-2819.912385
TS_{36-93}	-1125.694127	-1124.881821	-	-
TS_{45-46}	-1125.712512	-1124.900721	-2820.664784	-2819.916537
TS_{46-47}	-1125.719707	-1124.909562	-2820.660513	-2819.914719
TS_{47-48}	-1125.713859	-1124.902802	-2820.659168	-2819.912130
TS_{48-53}	-1125.715378	-1124.902716	-2820.660078	-2819.913601
TS_{53-54}	-1125.715545	-1124.905765	-2820.660374	-2819.915930
TS_{95-96}	-1125.723451	-1124.913136	-2820.659426	-2819.912741
TS_{97-101}	-1125.720543	-1124.905600	-2820.665494	-2819.919089
TS_{97-115}	-1125.694400	-1124.885121	-	-
$TS_{102-107}$	-1125.711682	-1124.900373	-2820.655958	-2819.912780
$TS_{107-113}$	-1125.702519	-1124.888569	-2820.657531	-2819.908350
$TS_{113-114}$	-1125.703635	-1124.888731	-2820.661372	-2819.912018
$TS_{116-123}$	-1125.698679	-1124.886908	-	-
$TS_{124-127}$	-1125.694309	-1124.881770	-	-

[a]The energies are calculated at the ONIOM(B3LYP/BS1) (25 °C) level. [b]The energies are calculated at the B3LYP+D3(PCM)/BS3//B3LYP+D3/BS2 (60 °C) level. The energies are given in a.u.

Table S3: The electronic and Gibbs free energies of LMs and TSs in the associative mechanism

No.	$E_{\mathrm{ONIOM}}{}^{a}$	$G_{\mathrm{ONIOM}}{}^{a}$	E^{b}	G^{b}
291	-1300.155345	-1299.226716	-2995.241576	-2994.379772
359	-1300.154670	-1299.227120	-2995.212374	-2994.354363
368	-1300.140555	-1299.205971	-2995.209142	-2994.343077
369	-1300.157997	-1299.230089	-	-
370	-1300.154705	-1299.227178	-	-
371	-1300.151196	-1299.227567	-	-
372	-1300.150897	-1299.222780	-	-
373	-1300.144374	-1299.216164	-	-
376	-1300.144018	-1299.215448	-	-
384	-1300.143256	-1299.214948	-	-
385	-1300.144023	-1299.216400	-	-
400	-1300.138067	-1299.209300	-	-
411	-1300.109017	-1299.184911	-	-
$TS_{291-359}$	-1300.135983	-1299.209685	-2995.215337	-2994.355446

[a]The energies are calculated at the ONIOM(B3LYP/BS1) (25 °C) level. [b]The energies are calculated at the B3LYP+D3(PCM)/BS3//B3LYP+D3/BS2 (60 °C) level. The energies are given in a.u.

2 Molecular structures

2.1 Hydride complexes in the associative mechanism (ONIOM(B3LYP/BS1:UFF) level)

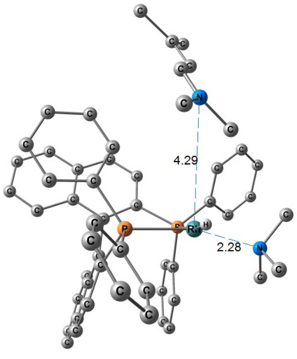

359

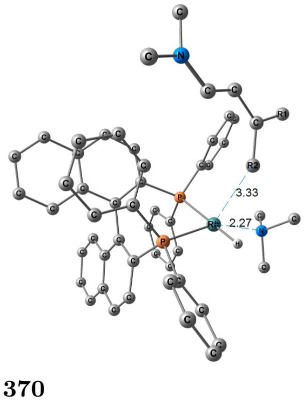

370

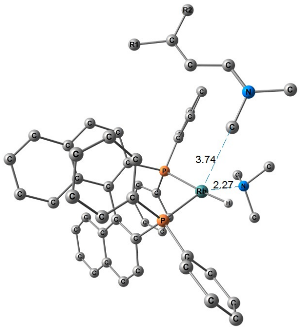

372

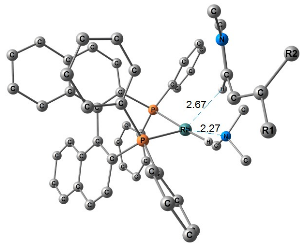

384

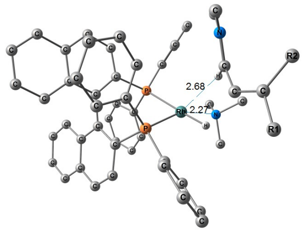

385

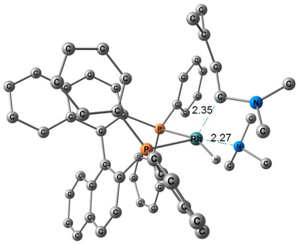

368

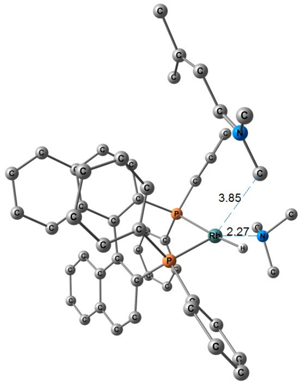

371

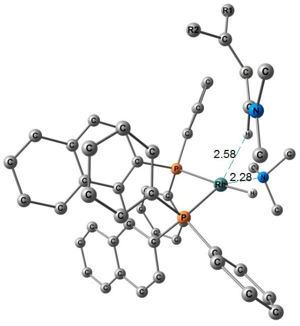

373

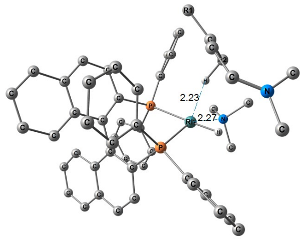

400

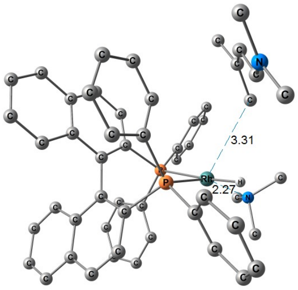

369

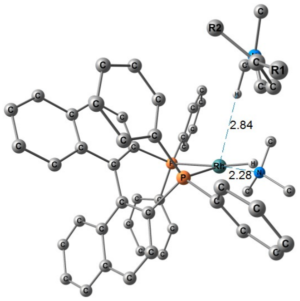

376

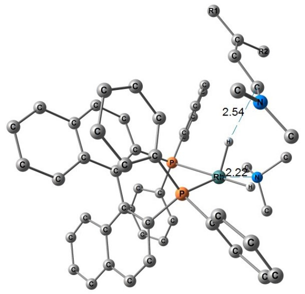

411

3 Cartesian coordinates

3.1 Dissociative mechanism (ONIOM(B3LYP/BS1:UFF) level)

1
Rh 1.969972 -0.127877 -0.369654
P 0.494486 -1.814103 0.491525
P 0.317517 1.609213 -0.369799
C 1.097704 -3.419748 -0.096172
C 0.297682 -1.872559 2.329038
C 1.238496 3.165421 -0.262384
C -0.851799 1.673289 -1.799087
C -1.206244 -1.598527 -0.175986
C -0.709742 1.493709 1.145676
N 3.955237 -1.079972 -0.078643
C 4.660411 -0.009559 -0.889065
C 3.713245 0.401633 -1.993150
C 3.551712 1.665409 -2.483802
C 2.663747 1.915273 -3.681528
C 4.350280 2.860265 -2.022171
H 5.617734 -0.398519 -1.266607
H 4.881412 0.827040 -0.223289
H 3.258754 -0.416982 -2.552279
H 3.751180 3.775123 -2.066355
H 4.745013 2.758139 -1.009060
H 5.202285 3.010125 -2.698318
H 1.899300 2.667305 -3.445207
H 3.254132 2.327293 -4.509628
H 2.168320 1.005316 -4.025573
C 4.305123 -2.427922 -0.621410
H 5.389673 -2.588261 -0.571537
H 3.816828 -3.201623 -0.028358
H 3.976966 -2.509678 -1.657927
C 4.321186 -1.008888 1.370990
H 4.043345 -0.029372 1.761789
H 3.768166 -1.780906 1.909093
H 5.397731 -1.175282 1.510063
C 1.361640 -3.571819 -1.459272
C 1.849123 -4.786367 -1.957072
C 2.092889 -5.844678 -1.076605
C 1.860162 -5.689239 0.293662
C 1.369093 -4.471195 0.781235
H 1.185911 -2.740400 -2.125214
H 2.046484 -4.906577 -3.018439
H 2.475615 -6.785859 -1.455584
H 2.070515 -6.508097 0.975986
H 1.228334 -4.343489 1.839532
C 0.999427 -0.964194 3.134582
C 0.814664 -0.955677 4.521063
C -0.075121 -1.849415 5.117757
C -0.783519 -2.751391 4.323547
C -0.609252 -2.757278 2.935819
H 1.672059 -0.249302 2.679863
H 1.357489 -0.245568 5.129754
H -0.221841 -1.837565 6.193129
H -1.485428 -3.437054 4.778529
H -1.183870 -3.443294 2.326735
C 2.286594 3.247436 0.655996
C 3.003631 4.440690 0.808483
C 2.679168 5.546815 0.016684
C 1.652166 5.459552 -0.929101
C 0.937860 4.262616 -1.070315
H 2.525598 2.386343 1.261457
H 3.810579 4.509363 1.532341
H 3.233913 6.472031 0.127136
H 1.415869 6.316248 -1.554119
H 0.163509 4.192827 -1.812381
C -0.715970 0.752334 -2.847785
C -1.628133 0.745165 -3.908068
C -2.688370 1.651797 -3.930058
C -2.836188 2.565933 -2.886664
C -1.931103 2.571827 -1.820396
H 0.090905 0.030936 -2.829531
H -1.513781 0.027200 -4.708648
H -3.398966 1.641013 -4.750460
H -3.665000 3.260835 -2.890024
H -2.072016 3.266360 -1.002138
C -1.719900 -2.534666 -1.098141
C -2.908638 -2.366975 -1.703252
C -3.697266 -1.249382 -1.418046
C -4.931965 -1.068833 -2.062671
C -5.702224 -0.009578 -1.763555
C -5.290068 0.925979 -0.804760
C -4.114190 0.787126 -0.167072
C -3.263659 -0.306696 -0.452488
C -2.010006 -0.487533 0.196498
H -1.167270 -3.423062 -1.338159
H -3.257523 -3.108407 -2.415821
H -5.273460 -1.784346 -2.801363
H -6.656113 0.123009 -2.263461
H -5.929155 1.772239 -0.577162
H -3.829879 1.538283 0.557168
C -0.540104 2.437456 2.179839
C -1.173595 2.325559 3.360570
C -2.043937 1.257874 3.599119
C -2.693517 1.134629 4.838214
C -3.548981 0.121647 5.055181
C -3.808590 -0.822334 4.052060
C -3.205205 -0.737935 2.853126
C -2.291338 0.305728 2.578126
C -1.629483 0.428349 1.324665
H 0.110938 3.281680 2.044758
H -1.005447 3.070348 4.132684
H -2.512640 1.857192 5.625236
H -4.049299 0.033126 6.013940
H -4.504077 -1.631013 4.248715
H -3.424860 -1.493810 2.111418

4
Rh 2.004639 0.289252 -0.042967
P 0.497158 -1.580335 -0.656250
P 0.180923 1.708698 0.672144
C 1.326183 -2.476824 -1.995982
C 0.027148 -2.766930 0.682825
C 0.876994 2.943625 1.804632
C -0.822334 2.551460 -0.635300
C -1.099442 -0.966058 -1.320414
C -1.015559 0.705069 1.641165
N 3.936096 -0.857725 0.102339
C 4.782020 0.399968 0.110707
C 3.836486 1.582038 0.123784
C 3.281299 2.105114 -1.046344
C 2.778134 3.535273 -1.049538
C 3.575307 1.578345 -2.438449
H 5.438893 0.400219 0.985971
H 5.417415 0.396952 -0.778796
H 3.836947 2.211358 1.009074
H 4.415568 2.143261 -2.869555
H 2.716501 1.728748 -3.098107
H 3.832335 0.519431 -2.458431
H 1.845524 3.667262 -1.600585
H 3.527905 4.160100 -1.553532
H 2.663421 3.934126 -0.038961
C 4.453482 -1.858649 -0.876465
H 4.390582 -1.460479 -1.889224
H 3.854751 -2.768342 -0.817017
H 5.496728 -2.115545 -0.653223
C 3.893225 -1.476037 1.467493
H 3.474357 -0.760244 2.176209
H 4.903638 -1.763691 1.787556
H 3.268557 -2.369080 1.432706
C 1.809454 -1.741381 -3.079612
C 2.488449 -2.378496 -4.124941
C 2.698501 -3.759611 -4.070091
C 2.240691 -4.499035 -2.974762
C 1.560439 -3.851872 -1.934994
H 1.646376 -0.675197 -3.104702
H 2.856348 -1.806050 -4.971651
H 3.227968 -4.258241 -4.874423
H 2.424788 -5.568701 -2.927222
H 1.251755 -4.416442 -1.073976
C 0.455924 -2.542483 1.999196
C 0.057625 -3.397629 3.031925
C -0.782485 -4.478844 2.765375
C -1.231241 -4.698784 1.463010
C -0.843033 -3.840638 0.429052
H 1.072086 -1.683322 2.225888
H 0.391692 -3.208551 4.042875
H -1.096161 -5.137433 3.569054
H -1.897720 -5.524307 1.252960
H -1.221609 -4.003882 -0.571861
C 1.791997 2.512843 2.767073
C 2.385060 3.428248 3.644608
C 2.067520 4.785804 3.543382
C 1.170181 5.228222 2.566516
C 0.581480 4.303758 1.694155
H 2.035717 1.462851 2.829187
H 3.091407 3.090580 4.397765
H 2.526555 5.500286 4.217841
H 0.942037 6.286905 2.479091
H -0.071953 4.655935 0.917729
C -0.513456 2.351571 -1.988963
C -1.296474 2.934193 -2.990660
C -2.404997 3.711123 -2.653978
C -2.732956 3.899166 -1.311388
C -1.957100 3.312098 -0.306579
H 0.326875 1.727417 -2.262214
H -1.046741 2.769715 -4.029872
H -3.016571 4.156330 -3.432338
H -3.603573 4.482694 -1.044212
H -2.241359 3.437840 0.730317
C -1.406384 -1.147555 -2.684933
C -2.526433 -0.651333 -3.238480
C -3.446695 0.057608 -2.461328
C -4.611515 0.582874 -3.044322
C -5.507268 1.241322 -2.290114
C -5.296389 1.411768 -0.915010
C -4.192723 0.924125 -0.321105
C -3.216941 0.228198 -1.072592
C -2.034499 -0.301011 -0.482591
H -0.744349 -1.703867 -3.322290
H -2.717456 -0.808189 -4.295827
H -4.797683 0.456776 -4.104345
H -6.406451 1.642485 -2.746119
H -6.033717 1.945530 -0.325200
H -4.063947 1.089180 0.739893
C -1.054294 0.834578 3.045124
C -1.821822 0.045495 3.816745
C -2.627648 -0.940569 3.241407
C -3.414580 -1.775380 4.051673
C -4.203432 -2.709433 3.494959
C -4.255773 -2.863521 2.102931
C -3.516955 -2.082933 1.294646
C -2.666991 -1.088133 1.831995
C -1.867313 -0.243612 1.011132
H -0.460624 1.580230 3.539620
H -1.811957 0.175885 4.894785
H -3.393259 -1.669917 5.129935
H -4.810012 -3.350428 4.126249
H -4.899204 -3.624549 1.675024
H -3.577617 -2.244846 0.227130

5
Rh -0.151864 -3.291370 0.244181
P 0.940633 -1.727690 1.636800
P -0.987775 -1.712529 -1.325705
C 0.885780 -2.389019 3.322612
C 2.695182 -1.400485 1.164678
C -1.399007 -2.684774 -2.795923
C -2.440422 -0.687555 -0.826773
C 0.123968 -0.085311 1.631171
C 0.366637 -0.560833 -1.746843
N 0.418312 -5.238827 0.972009
C -0.291439 -5.679488 -0.285601
C -1.121094 -6.928913 -0.236489
C -1.021411 -7.975500 -1.084382
C -1.957551 -9.155253 -0.940824
C -0.038143 -8.094276 -2.226377
H -1.030645 -4.831128 -0.548465
H 0.447900 -5.713529 -1.082067
H -1.903765 -6.943906 0.516503
H 0.541798 -9.020448 -2.133881
H -0.578099 -8.155560 -3.179961
H 0.666061 -7.262110 -2.298354
H -2.545890 -9.294497 -1.857035
H -1.392042 -10.082636 -0.784896
H -2.651617 -9.030565 -0.105416
C 1.874410 -5.571665 0.967273
H 2.325403 -5.210282 0.043567
H 2.343981 -5.068663 1.815548
H 2.025459 -6.654187 1.059831
C -0.226473 -5.691066 2.239610
H -1.247047 -5.309965 2.290021
H -0.228324 -6.783684 2.312159
H 0.344925 -5.282282 3.074594
C -0.356135 -2.722819 3.865882
C -0.444020 -3.270302 5.151661
C 0.724469 -3.504858 5.882876
C 1.973674 -3.202832 5.331030
C 2.050104 -2.651403 4.045549
H -1.248879 -2.555512 3.282415
H -1.410999 -3.522264 5.577641
H 0.663131 -3.934286 6.876761
H 2.879564 -3.405766 5.895435
H 3.013540 -2.453772 3.609411
C 3.249605 -2.044428 0.049152
C 4.556775 -1.757871 -0.357724
C 5.322860 -0.824841 0.341917
C 4.777649 -0.176207 1.450346
C 3.467834 -0.452427 1.855495
H 2.656138 -2.752640 -0.514092
H 4.970490 -2.256194 -1.223803
H 6.335354 -0.599893 0.021841
H 5.362630 0.556160 1.990174
H 3.046264 0.074210 2.702113
C -0.483294 -3.645974 -3.227324
C -0.785966 -4.476985 -4.312673
C -2.020318 -4.347999 -4.956796
C -2.950717 -3.401700 -4.514481
C -2.639106 -2.577698 -3.425090
H 0.464828 -3.738390 -2.720161
H -0.071775 -5.221827 -4.652471
H -2.261892 -4.990558 -5.796335
H -3.915368 -3.317633 -5.007355
H -3.370424 -1.876259 -3.063639
C -3.054166 -0.903270 0.415772
C -4.111183 -0.088556 0.835018
C -4.565383 0.949508 0.020772
C -3.960021 1.171443 -1.216352
C -2.898680 0.363860 -1.637473
H -2.697563 -1.694557 1.062684
H -4.571687 -0.261260 1.798177
H -5.381692 1.584569 0.350164
H -4.299119 1.982492 -1.846562
H -2.419738 0.560765 -2.588076
C -0.478978 0.392258 2.812170
C -1.181309 1.537948 2.843576
C -1.323761 2.313149 1.689201
C -2.074946 3.499426 1.719901
C -2.190888 4.255068 0.615245
C -1.563983 3.875730 -0.579546
C -0.835951 2.747074 -0.648072
C -0.688517 1.913994 0.485780
C 0.066852 0.708210 0.452472
H -0.382106 -0.157291 3.730660
H -1.639192 1.861437 3.773525
H -2.562813 3.814697 2.634697
H -2.771231 5.171411 0.646159
H -1.669740 4.502267 -1.458616
H -0.378677 2.486983 -1.592934
C 1.014345 -0.673293 -2.991994
C 2.125257 0.027124 -3.281771
C 2.673620 0.906732 -2.341898
C 3.844510 1.623304 -2.638321
C 4.353457 2.486769 -1.743438
C 3.724761 2.689066 -0.506914
C 2.601965 2.021605 -0.186915
C 2.032493 1.095873 -1.090714
C 0.849614 0.367341 -0.787978
H 0.626670 -1.337263 -3.744545
H 2.600438 -0.096216 -4.250404
H 4.342499 1.488179 -3.591231
H 5.257381 3.038802 -1.979229
H 4.152209 3.392569 0.199262
H 2.153925 2.199073 0.781474

8
Rh -3.581897 2.371956 1.293186
P -4.320510 3.688787 -0.551711
P -4.395176 3.822908 2.979672
C -3.369096 3.106351 -1.977526
C -6.122945 3.660951 -0.953301
C -4.840656 2.789467 4.399752
C -3.181473 5.107265 3.515490
C -3.869930 5.429651 -0.223384
C -5.884325 4.756850 2.454809
N -2.430458 0.873472 2.311200
C -2.254693 0.326301 0.912551
C -0.863280 0.220580 0.368828
C -0.272799 -0.901240 -0.095883
C 1.119685 -0.831736 -0.683565
C -0.884944 -2.282624 -0.098686
H -2.804527 -0.611638 0.847192
H -2.840308 1.018751 0.190904
H -0.322916 1.159161 0.287842
H -0.958619 -2.661723 -1.125792
H -0.240619 -2.982505 0.447757
H -1.881683 -2.330865 0.346303
H 1.804896 -1.494904 -0.140628
H 1.113038 -1.171445 -1.727261
H 1.527356 0.182297 -0.656630
C -1.194177 1.408060 2.959828
H -0.729106 2.152524 2.317480
H -1.494934 1.882102 3.896252
H -0.481059 0.602360 3.167415
C -3.114410 -0.106033 3.210602
H -4.084689 -0.370785 2.788880
H -2.501046 -1.005215 3.341804
H -3.267623 0.358508 4.186350
C -2.000233 2.890788 -1.812237
C -1.231807 2.372031 -2.861203
C -1.846737 2.059379 -4.077386
C -3.222272 2.253145 -4.241871
C -3.982694 2.767873 -3.183673
H -1.538343 3.128466 -0.866095
H -0.166088 2.204772 -2.732615
H -1.257419 1.654635 -4.892774
H -3.698269 1.991954 -5.182984
H -5.045899 2.881379 -3.300823
C -6.986698 2.823966 -0.231821
C -8.363355 2.843389 -0.479436
C -8.892335 3.698177 -1.447103
C -8.040477 4.534695 -2.168689
C -6.664147 4.523868 -1.920482
H -6.588574 2.170095 0.533492
H -9.019396 2.197802 0.088364
H -9.961454 3.716605 -1.633709
H -8.445835 5.209131 -2.910850
H -6.015520 5.195480 -2.468252
C -5.736060 1.736908 4.200773
C -6.080280 0.888292 5.260034
C -5.502216 1.085535 6.517789
C -4.579326 2.117768 6.715102
C -4.244834 2.963448 5.649598
H -6.159990 1.584068 3.219834
H -6.783976 0.074981 5.107428
H -5.760533 0.428083 7.340593
H -4.117353 2.253812 7.688998
H -3.506429 3.732259 5.794529
C -1.921161 5.175045 2.904330
C -1.015904 6.181672 3.255787
C -1.359389 7.132434 4.217464
C -2.613434 7.076240 4.826372
C -3.525675 6.077287 4.471397
H -1.654432 4.458221 2.139165
H -0.049724 6.226772 2.772031
H -0.657597 7.915934 4.485366

H -2.890019 7.817338 5.564074
H -4.505013 6.057533 4.932172
C -2.785123 6.008310 -0.909910
C -2.301882 7.220430 -0.584745
C -2.876274 7.955912 0.457563
C -2.358241 9.213057 0.809099
C -2.928120 9.927712 1.793900
C -4.046381 9.434680 2.480549
C -4.571101 8.235377 2.172471
C -4.002465 7.441847 1.150046
C -4.524772 6.166987 0.797751
H -2.312131 5.478289 -1.717669
H -1.456728 7.626157 -1.132777
H -1.498982 9.616583 0.286487
H -2.524162 10.899108 2.059615
H -4.491123 10.029528 3.270899
H -5.427600 7.887076 2.733755
C -7.115487 4.515211 3.097048
C -8.258909 5.094675 2.692594
C -8.259368 5.983742 1.614072
C -9.458456 6.573666 1.182012
C -9.453055 7.446315 0.160672
C -8.255802 7.783490 -0.485314
C -7.087656 7.239761 -0.100117
C -7.041108 6.311252 0.966290
C -5.826705 5.708514 1.399661
H -7.164938 3.855558 3.943940
H -9.186693 4.870138 3.210181
H -10.394625 6.332030 1.671361
H -10.382671 7.900130 -0.166912
H -8.274972 8.491921 -1.306455
H -6.189671 7.519924 -0.633646

22

Rh 0.437993 -3.727096 -1.697938
P 2.220559 -2.138624 -1.499724
P -1.233214 -2.375901 -0.199562
C 3.754538 -3.026494 -1.866248
C 2.067502 -0.690183 -2.635125
C -2.912288 -2.792219 -0.744578
C -1.103290 -2.530612 1.637828
C 2.298726 -1.468749 0.203149
C -0.961776 -0.593289 -0.534660
N -0.573810 -5.642644 -2.009731
C 0.619955 -5.810635 -1.238469
C 0.559666 -6.148606 0.173370
C 1.525198 -6.776510 0.897837
C 1.330454 -6.990135 2.379856
C 2.818850 -7.316344 0.344295
H 1.446591 -6.214365 -1.810217
H 1.388465 -4.177755 -2.851167
H -0.338706 -5.847687 0.702334
H 3.674448 -6.854659 0.853552
H 2.888242 -8.394719 0.535707
H 2.938991 -7.159432 -0.730178
H 1.400014 -8.055710 2.634156
H 2.121736 -6.482136 2.947329
H 0.363657 -6.615861 2.727381
C -0.528843 -6.086775 -3.438121
H -0.815872 -7.142255 -3.505815
H -1.226254 -5.479263 -4.016465
H 0.475506 -5.942910 -3.832075
C -1.898767 -5.950455 -1.405013
H -2.675023 -5.560932 -2.065117
H -2.024717 -7.032990 -1.297754
H -2.002923 -5.476419 -0.432214
C 3.984559 -4.239321 -1.217728
C 5.127727 -4.994437 -1.507632
C 6.028611 -4.538645 -2.474455
C 5.785422 -3.341168 -3.154288
C 4.639785 -2.592762 -2.854281
H 3.278042 -4.580445 -0.476490
H 5.316907 -5.930454 -0.990016
H 6.914265 -5.120472 -2.705489
H 6.478795 -3.000831 -3.918572
H 4.440013 -1.691689 -3.405725
C 0.980636 -0.606425 -3.518166
C 0.822727 0.510924 -4.344581
C 1.745514 1.556377 -4.298484
C 2.828042 1.482777 -3.421806
C 2.985029 0.372549 -2.585893
H 0.249690 -1.404223 -3.550437
H -0.023494 0.566467 -5.015869
H 1.618400 2.425506 -4.936219
H 3.538982 2.296448 -3.371253
H 3.814235 0.338123 -1.890819
C -3.191533 -2.757768 -2.112367
C -4.458980 -3.116102 -2.587443
C -5.440507 -3.536554 -1.684879
C -5.155043 -3.610996 -0.317842
C -3.884851 -3.247047 0.147259
H -2.417559 -2.453181 -2.800053
H -4.678984 -3.079497 -3.650478
H -6.421841 -3.821920 -2.047401
H -5.912479 -3.964382 0.376206
H -3.653178 -3.354279 1.191685
C -0.086869 -3.313156 2.204758
C 0.051582 -3.402475 3.593538
C -0.812930 -2.699829 4.432795
C -1.811092 -1.897655 3.879048
C -1.947942 -1.799887 2.490353
H 0.623519 -3.816748 1.566246
H 0.843969 -4.006179 4.014256
H -0.699932 -2.764623 5.510287
H -2.472559 -1.335262 4.523999
H -2.708894 -1.153446 2.072249
C 3.350564 -1.872082 1.048718
C 3.399139 -1.522945 2.345743
C 2.395141 -0.725585 2.902814
C 2.442669 -0.372510 4.261355
C 1.490704 0.414926 4.788901
C 0.441362 0.897659 3.995141
C 0.361466 0.580918 2.690630
C 1.334524 -0.251011 2.089375
C 1.283127 -0.611703 0.713051
H 4.149667 -2.479911 0.664985
H 4.224609 -1.864834 2.962921
H 3.248765 -0.728947 4.891742
H 1.533952 0.685443 5.838790
H -0.316093 1.532323 4.442002
H -0.471760 0.965015 2.118124
C -1.963437 0.151748 -1.189167
C -1.781293 1.434014 -1.548360
C -0.580393 2.087456 -1.258253
C -0.394020 3.427532 -1.635289
C 0.745364 4.066150 -1.321421
C 1.760448 3.410048 -2.012038
C 1.620083 2.126405 -0.236352
C 0.443665 1.406421 -0.552084
C 0.258575 0.045737 -0.175825
H -2.915120 -0.295751 -1.410944
H -2.578137 1.966344 -2.059123
H -1.170213 3.957233 -2.174947
H 0.881315 5.102693 -1.612166
H 2.670069 3.946776 -0.365112
H 2.432869 1.656339 0.300302

33

Rh 0.093010 -4.899516 -0.703553
P 0.401233 -3.043280 0.782352
P -2.196789 -4.148027 -1.241136
C 1.888665 -2.939047 1.824216
C 0.384427 -1.362744 0.011704
C -2.446209 -4.448804 -3.012056
C -3.508704 -5.037894 -0.288449
C -1.060860 -3.238861 1.865732
C -2.553595 -2.364182 -0.951791
N 1.984452 -5.984649 -0.584103
C 1.515768 -7.093700 -1.497557
C 0.046360 -7.351028 -1.281694
C -0.863792 -7.556336 -2.280097
C -2.224047 -8.135390 -1.962502
C -0.563775 -7.425384 -3.755718
H 2.118631 -7.996359 -1.307280
H 1.714277 -6.773703 -2.521322
H -0.242940 -7.639525 -0.273927
H -1.453482 -7.091174 -4.299757
H -0.293179 -8.404964 -4.171042
H 0.244932 -6.726420 -3.978319
H -2.352554 -9.079630 -2.507433
H -3.033122 -7.478853 -2.304207
H -2.354573 -8.338400 -0.898331
C 2.253936 -6.506773 0.794462
H 1.351226 -6.962033 1.200328
H 2.541052 -5.675181 1.439691
H 3.066756 -7.244636 0.775837
C 3.233080 -5.390988 -1.169314
H 2.966940 -4.701506 -1.970526
H 3.877033 -6.188150 -1.561769
H 3.793921 -4.871531 -0.395918
C 3.116661 -2.816076 1.180068
C 4.300974 -2.679675 1.913662
C 4.243637 -2.617577 3.308312
C 3.008633 -2.652452 3.961945
C 1.830903 -2.787453 3.214833
H 3.135264 -2.786061 0.102500
H 5.256747 -2.598831 1.404050
H 5.156521 -2.503547 3.882246
H 2.964994 -2.547913 5.042424
H 0.886839 -2.718245 3.722554
C 0.453793 -1.224310 -1.381352
C 0.473447 0.045272 -1.968740
C 0.442111 1.189622 -1.171074
C 0.403064 1.061428 0.217361
C 0.383205 -0.206060 0.808644
H 0.492826 -2.104931 -2.005863
H 0.514856 0.137386 -3.045296
H 0.453560 2.174242 -1.627519
H 0.380516 1.943869 0.842431
H 0.351067 -0.293289 1.887026
C -1.434997 -4.060678 -3.890974
C -1.586299 -4.238455 -5.271644
C -2.758190 -4.816658 -5.768628
C -3.770904 -5.219484 -4.892452
C -3.610369 -5.034057 -3.513239
H -0.534539 -3.620501 -3.496256
H -0.798727 -3.934453 -5.955167
H -2.879502 -4.960222 -6.836641
H -4.675542 -5.677456 -5.282735
H -4.385955 -5.358531 -2.845016
C -3.149305 -6.054179 0.607828
C -4.122139 -6.702042 1.375954
C -5.462308 -6.329284 1.273321
C -5.827429 -5.301855 0.403477
C -4.857253 -4.648992 -0.363517
H -2.109814 -6.331307 0.717763
H -3.829604 -7.485541 2.061390
H -6.215138 -6.827117 1.876175
H -6.862136 -4.994684 0.333683
H -5.150024 -3.834689 -1.013812
C -0.975286 -4.237289 2.853741
C -2.011193 -4.549995 3.649913
C -3.252476 -3.933367 3.465067
C -4.339586 -4.271917 4.287167
C -5.546880 -3.724621 4.070840
C -5.743020 -2.818928 3.019706
C -4.727109 -2.470579 2.210240
C -3.430984 -3.002220 2.408220
C -2.327404 -2.664649 1.572233
H -0.046783 -4.779358 2.969105
H -1.889592 -5.308404 4.417049
H -4.213640 -4.981242 5.096548
H -6.382887 -3.990623 4.709322
H -6.729541 -2.398816 2.856182
H -4.930787 -1.781846 1.402950
C -2.862255 -1.535949 -2.050339
C -3.111289 -0.222273 -1.906913
C -3.070759 0.373662 -0.642300
C -3.307094 1.750605 -0.498689
C -3.264665 2.320840 0.717216
C -2.985015 1.554666 1.857454
C -2.755038 0.233177 1.760696
C -2.791322 -0.413631 0.503795
C -2.546881 -1.806830 0.358425
H -2.902068 -1.946689 -3.044065
H -3.336176 0.379350 -2.782184
H -3.524157 2.361794 -1.366756
H -3.448636 3.385221 0.821171
H -2.953551 2.036890 2.828638
H -2.531926 -0.319912 2.663128

36

Rh 1.138691 -3.338257 -0.511381
P 1.574863 -2.519106 1.734437
P -0.947942 -2.045865 -0.962783
C 2.273274 -3.944704 2.604197
C 2.703242 -1.074458 1.975763
C -1.238349 -1.998764 -2.752353
C -2.484591 -2.598613 -0.101202
C -0.004587 -2.098407 2.562134
C -0.603668 -0.347669 -0.369995
N 1.884207 -4.658307 -2.047502
C 2.823186 -4.531037 -0.944682
C 4.125358 -3.876288 -1.113527
C 5.250673 -4.140746 -0.405081
C 6.501486 -3.322115 -0.634770
C 5.392462 -5.224253 0.636701
H 1.778449 -2.108042 -1.106891
H 2.821063 -5.416548 -0.310825
H 4.165988 -3.060141 -1.828398
H 6.145711 -5.959518 0.327720
H 5.742261 -4.798869 1.586138
H 4.463200 -5.762819 0.844086
H 6.800115 -2.801909 0.285610
H 7.344019 -3.967358 -0.915880
H 6.365177 -2.574455 -1.420941
C 1.117973 -5.935981 -2.103619
H 0.840185 -6.240232 -1.091225
H 0.211683 -5.780012 -2.691976
H 1.723083 -6.727478 -2.561040
C 2.244139 -4.164118 -3.403242
H 2.666264 -3.164140 -3.342189
H 2.963538 -4.841577 -3.876315
H 1.332599 -4.122174 -4.002926
C 1.653858 -5.183711 2.437407
C 2.173125 -6.324578 3.061226
C 3.324872 -6.217766 3.847111
C 3.958853 -4.980959 4.004233
C 3.433484 -3.846205 3.372775
H 0.767220 -5.252928 1.825824
H 1.689435 -7.289177 2.935724
H 3.733616 -7.098655 4.329786
H 4.862249 -4.906499 4.603088
H 3.940784 -2.902489 3.469538
C 3.472199 -0.572959 0.913782
C 4.306239 0.534230 1.101354
C 4.389192 1.150660 2.349604
C 3.641308 0.651535 3.415308
C 2.801756 -0.451526 3.231777
H 3.438840 -1.045508 -0.055119
H 4.892670 0.910017 0.273660
H 5.036424 2.010408 2.492148
H 3.702560 1.123309 4.386748
H 2.220947 -0.823866 4.065903
C -0.178778 -1.669224 -3.599692
C -0.353748 -1.666922 -4.988974
C -1.594566 -2.017087 -5.529000
C -2.652119 -2.376285 -4.687875
C -2.466480 -2.374241 -3.299403
H 0.776934 -1.407242 -3.176729
H 0.468915 -1.401739 -5.647049
H -1.734663 -2.021503 -6.604346
H -3.608905 -2.666734 -5.112858
H -3.271486 -2.687087 -2.658190
C -2.474306 -3.779318 0.655599
C -3.615893 -4.181537 1.356900
C -4.778613 -3.411646 1.309392
C -4.797915 -2.236612 0.557631
C -3.656904 -1.825893 -0.139149
H -1.573199 -4.376638 0.709695
H -3.593023 -5.090735 1.942005
H -5.662108 -3.722554 1.857935
H -5.692661 -1.629713 0.525696
H -3.675916 -0.901117 -0.701496
C -0.426183 -2.861927 3.669399
C -1.611205 -2.656326 4.268865
C -2.470294 -1.651740 3.814222
C -3.709468 -1.442352 4.441234
C -4.522702 -0.459468 4.020533
C -4.148398 0.371553 2.955949
C -2.970995 0.200947 2.328891
C -2.082272 -0.824986 2.728773
C -0.830181 -1.042609 2.086465
H 0.206465 -3.636592 4.066823
H -1.898915 -3.273603 5.114685
H -4.015706 -2.068410 5.271015
H -5.477474 -0.301204 4.511255
H -4.819658 1.160904 2.635223
H -2.728024 0.859127 1.506012
C -0.432202 0.689940 -1.306401
C 0.009915 1.908002 -0.948442
C 0.298985 2.192060 0.389947
C 0.765029 3.464177 0.760262
C 0.993149 3.750734 2.052844
C 0.761806 2.792444 3.049097
C 0.317100 1.563229 2.733240
C 0.082388 1.205535 1.385771
C -0.384513 -0.085030 1.011408
H -0.655895 0.523721 -2.344783
H 0.139528 2.675841 -1.705554
H 0.936088 4.225069 0.007762
H 1.350290 4.736434 2.332676
H 0.943807 3.048708 4.087085
H 0.153387 0.853295 3.532412

45

Rh 1.774548 0.322958 -0.281545
P 0.381286 -1.575944 -0.764588
P 0.056386 1.665350 0.626096
C 1.260874 -2.494942 -2.049475
C 0.036505 -2.686431 0.666498
C 0.837129 2.803770 1.796913
C -0.936239 2.614667 -0.607838
C -1.223697 -1.010555 -1.426814
C -1.139098 0.632832 1.558224
N 4.671921 -1.016894 0.800553
C 4.175636 -0.244760 -0.327972
C 3.777283 1.164293 0.013569
C 3.325550 2.073980 -0.945963
C 3.109140 3.525103 -0.575589
C 3.420735 1.826006 -2.440411
H 4.816390 -0.292560 -1.232324
H 3.241768 -0.828017 -0.715921
H 3.941700 1.492041 1.034163
H 4.330885 2.308450 -2.825914
H 2.573482 2.273202 -2.966768
H 3.468630 0.767479 -2.704407
H 2.144803 3.900594 -0.933996
H 3.884394 4.141031 -1.047750
H 3.172351 3.688317 0.504204
C 4.641527 -2.469883 0.564893
H 3.620046 -2.776513 0.309052
H 4.932999 -2.989064 1.479392
H 5.313472 -2.793232 -0.251183
C 5.970243 -0.541840 1.320995
H 5.910695 0.519497 1.572959
H 6.794793 -0.684042 0.599723
H 6.206399 -1.093538 2.233099
C 1.759355 -1.785913 -3.142912
C 2.514185 -2.437734 -4.125556
C 2.784720 -3.803460 -3.994746
C 2.310958 -4.512790 -2.886184
C 1.555930 -3.850886 -1.909436
H 1.547117 -0.731262 -3.229731
H 2.892397 -1.889460 -4.983912
H 3.371196 -4.313476 -4.751166
H 2.538078 -5.570120 -2.780755
H 1.220892 -4.390736 -1.040981
C 0.636921 -2.429753 1.907933
C 0.339443 -3.228678 3.016712
C -0.559634 -4.289258 2.899200
C -1.160300 -4.551680 1.667534
C -0.870786 -3.752586 0.556830
H 1.325792 -1.600885 2.012773
H 0.805644 -3.017270 3.969447
H -0.793635 -4.904568 3.762206
H -1.866398 -5.365533 1.572793
H -1.358976 -3.950955 -0.388737
C 1.762763 2.283582 2.702607
C 2.427426 3.126876 3.600789
C 2.168829 4.500756 3.577569
C 1.257940 5.030959 2.658129
C 0.598273 4.178375 1.763458
H 1.960112 1.221606 2.705178
H 3.143876 2.720621 4.309315
H 2.683362 5.159308 4.268664
H 1.073776 6.101403 2.632684
H -0.072368 4.594568 1.033894
C -0.622090 2.524701 -1.971432
C -1.401118 3.188411 -2.924607
C -2.504757 3.944363 -2.528183
C -2.830089 4.032676 -1.174495
C -2.059516 3.363014 -0.218535
H 0.216764 1.921337 -2.291196
H -1.150975 3.106389 -3.973584
H -3.112045 4.454282 -3.269403

H -3.693313 4.604747 -0.862122
H -2.338152 3.415293 0.826206
C -1.510517 -1.184813 -2.794172
C -2.610744 -0.660999 -3.362929
C -3.522381 0.074187 -2.597508
C -4.664918 0.630690 -3.195190
C -5.553112 1.311708 -2.451814
C -5.357127 1.473947 -1.073286
C -4.274777 0.956895 -0.465154
C -3.306919 0.239617 -1.205147
C -2.147034 -0.318634 -0.600409
H -0.840315 -1.748988 -3.418642
H -2.790101 -0.810716 -4.423402
H -4.840445 0.510346 -4.257731
H -6.435271 1.736898 -2.919210
H -6.088848 2.024983 -0.492466
H -4.156201 1.113904 0.598449
C -1.171110 0.716931 2.964953
C -1.944930 -0.088865 3.712335
C -2.764109 -1.045743 3.106706
C -3.556880 -1.899139 3.891411
C -4.357334 -2.805870 3.306846
C -4.416199 -2.912931 1.910662
C -3.672084 -2.113596 1.125863
C -2.809909 -1.145735 1.692904
C -2.001329 -0.285560 0.897627
H -0.563884 1.439413 3.479427
H -1.928682 0.003967 4.794135
H -3.530400 -1.830222 4.972535
H -4.968554 -3.461041 3.918882
H -5.070341 -3.651725 1.460372
H -3.740025 -2.238663 0.053882
46
Rh 1.822396 0.314490 -0.315442
P 0.425069 -1.597453 -0.746881
P 0.100120 1.689053 0.620469
C 1.293171 -2.537521 -2.024932
C 0.050493 -2.696590 0.687536
C 0.910429 2.807134 1.790654
C -0.920676 2.656072 -0.577238
C -1.179441 -1.025848 -1.408838
C -1.085202 0.658515 1.564873
N 5.015853 -0.873201 0.488510
C 4.280273 -0.133700 -0.454216
C 3.819711 1.243924 -0.127044
C 3.258024 2.081329 -1.097793
C 3.003993 3.536916 -0.766486
C 3.307576 1.794061 -2.587862
H 4.729520 -0.218734 -1.446561
H 3.192087 -0.766722 -0.720686
H 4.028760 1.649902 0.855439
H 4.182227 2.303706 -3.018486
H 2.424833 2.191689 -3.094328
H 3.385348 0.732333 -2.827328
H 2.016291 3.868249 -1.102282
H 3.741985 4.162720 -1.283452
H 3.097512 3.736503 0.305102
C 5.464011 -2.193683 0.022288
H 4.628023 -2.915331 -0.029496
H 6.222079 -2.585301 0.701737
H 5.904790 -2.105105 -0.974117
C 4.635465 -0.812402 1.904894
H 3.650298 -1.274730 2.082156
H 4.602222 0.219334 2.257231
H 5.385484 -1.343762 2.492316
C 1.763461 -1.850169 -3.144590
C 2.513878 -2.515351 -4.121616
C 2.810816 -3.871934 -3.958392
C 2.368746 -4.558299 -2.822747
C 1.617977 -3.883017 -1.851811
H 1.531163 -0.802383 -3.257121
H 2.868768 -1.984191 -5.000480
H 3.394511 -4.392076 -4.710073
H 2.618778 -5.607505 -2.691166
H 1.313549 -4.403246 -0.960516
C 0.587431 -2.406372 1.949996
C 0.263896 -3.197379 3.057135
C -0.601300 -4.283053 2.917317
C -1.144299 -4.575921 1.666001
C -0.829058 -3.783939 0.557219
H 1.237563 -1.550905 2.074093
H 0.679404 -2.958437 4.026618
H -0.856542 -4.892215 3.778689
H -1.825998 -5.408215 1.553828
H -1.274298 -4.005881 -0.404195
C 1.850172 2.266105 2.669195
C 2.543439 3.091454 3.562366
C 2.298783 4.468113 3.561244
C 1.373831 5.018953 2.668286
C 0.685905 4.184303 1.778029
H 2.036347 1.202686 2.653831
H 3.271504 2.669901 4.249801
H 2.835811 5.113100 4.248092
H 1.201699 6.091704 2.658683
H 0.007190 4.616670 1.065566
C -0.659454 2.560118 -1.951453
C -1.461189 3.237241 -2.875838
C -2.536560 4.011738 -2.439752
C -2.811919 4.103572 -1.075235
C -2.018734 3.420112 -0.147981
H 0.153787 1.939497 -2.301365
H -1.251364 3.150531 -3.933264
H -3.161485 4.532318 -3.158572
H -3.653758 4.689631 -0.731916
H -2.259902 3.474812 0.905910
C -1.479754 -1.221610 -2.770396
C -2.577897 -0.694588 -3.340096
C -3.475566 0.063767 -2.580969
C -4.615996 0.623044 -3.180088
C -5.492518 1.324293 -2.441726
C -5.286630 1.504596 -1.066925
C -4.205975 0.985389 -0.457628
C -3.249553 0.248096 -1.192714
C -2.091810 -0.313037 -0.586456
H -0.823779 -1.807695 -3.389403
H -2.767493 -0.861379 -4.396295
H -4.799524 0.488398 -4.239573
H -6.373104 1.751629 -2.910138
H -6.009123 2.071649 -0.490021
H -4.079652 1.156483 0.602883
C -1.115416 0.754476 2.970804
C -1.888729 -0.045304 3.725175
C -2.708261 -1.007361 3.127715
C -3.501298 -1.853647 3.919777
C -4.300755 -2.766295 3.343088
C -4.358228 -2.886734 1.947918
C -3.613941 -2.094462 1.156156
C -2.753058 -1.120744 1.714705
C -1.944493 -0.267709 0.911851
H -0.508702 1.482967 3.478279
H -1.873224 0.057391 4.806131
H -3.475953 -1.774516 5.000228
H -4.912273 -3.415949 3.960699
H -5.011423 -3.630272 1.504107
H -3.680905 -2.229662 0.085326
47
Rh 1.885345 -0.030052 -0.285342
P 0.254267 -1.679948 -0.982366
P 0.170292 1.642942 0.632960
C 0.968503 -2.503714 -2.425039
C -0.116959 -2.931947 0.321840
C 0.990682 2.818970 1.748508
C -0.874075 2.577446 -0.574451
C -1.357021 -0.948448 -1.450444
C -1.026297 0.651712 1.610349
N 5.204711 -0.077667 1.656220
C 4.618788 0.070563 0.469762
C 3.770559 1.120126 0.034486
C 3.334282 1.235524 -1.341494
C 2.814651 2.599173 -1.766042
C 4.037125 0.519338 -2.486407
H 4.851656 -0.710517 -0.242240
H 2.787136 -1.241163 -0.740864
H 3.603750 1.972002 0.684516
H 4.914780 1.101518 -2.802867
H 3.368672 0.448147 -3.348219
H 4.358150 -0.494429 -2.242850
H 2.188774 2.525187 -2.657790
H 3.664121 3.248704 -2.023345
H 2.251649 3.093661 -0.968963
C 5.977197 -1.297772 1.970492
H 5.503623 -1.833266 2.799327
H 6.997646 -1.027579 2.255673
H 6.007679 -1.950933 1.097683
C 5.073738 0.884015 2.764062
H 4.812325 1.870044 2.381845
H 6.030305 0.955267 3.285354
H 4.307068 0.549837 3.470798
C 1.375188 -1.713089 -3.499722
C 1.996820 -2.293342 -4.611981
C 2.228526 -3.672121 -4.631268
C 1.850274 -4.465425 -3.543169
C 1.229062 -3.874064 -2.435418
H 1.190360 -0.649895 -3.471978
H 2.301862 -1.680001 -5.455411
H 2.711981 -4.127344 -5.488881
H 2.049466 -5.533638 -3.555348
H 0.974634 -4.481304 -1.584270
C 0.488744 -2.827481 1.583079
C 0.172248 -3.735733 2.598379
C -0.750770 -4.756274 2.367505
C -1.359335 -4.866507 1.116829
C -1.054947 -3.954275 0.101422
H 1.194607 -2.030055 1.778227
H 0.641474 -3.640436 3.568376
H -0.998108 -5.457533 3.158311
H -2.083309 -5.649458 0.935289
H -1.551168 -4.034206 -0.857354
C 1.822720 2.322765 2.752716
C 2.496172 3.197007 3.614845
C 2.361783 4.577209 3.441316
C 1.567860 5.082132 2.407319
C 0.891585 4.198470 1.556549
H 1.941557 1.254740 2.857571
H 3.131557 2.809317 4.406049
H 2.888759 5.258801 4.099877
H 1.488089 6.155500 2.259336
H 0.319151 4.591889 0.735641
C -0.702390 2.381234 -1.952376
C -1.518161 3.047502 -2.872595
C -2.521000 3.909460 -2.428377
C -2.711468 4.098206 -1.059570
C -1.902855 3.428359 -0.135770
H 0.041148 1.681509 -2.307187
H -1.378635 2.881139 -3.931932
H -3.157919 4.420745 -3.143262
H -3.498678 4.751571 -0.708248
H -2.076135 3.563978 0.924131
C -1.792633 -1.045956 -2.786763
C -2.920608 -0.454297 -3.215367
C -3.718744 0.274833 -2.329085
C -4.891957 0.899796 -2.782358
C -5.671893 1.577572 -1.923990
C -5.331257 1.668662 -0.567606
C -4.215593 1.084380 -0.096004
C -3.356740 0.365634 -0.960546
C -2.163993 -0.262580 -0.501192
H -1.224021 -1.613988 -3.501381
H -3.214500 -0.549064 -4.256514
H -5.178387 0.835916 -3.825503
H -6.578299 2.056229 -2.280067
H -5.977119 2.219296 0.107795
H -3.986038 1.189821 0.955529
C -1.037102 0.758729 3.015181
C -1.762511 -0.066470 3.789637
C -2.559564 -1.060149 3.214468
C -3.309075 -1.926655 4.026843
C -4.111051 -2.849956 3.470896
C -4.217748 -2.958360 2.077658
C -3.514481 -2.147659 1.267266
C -2.644428 -1.170023 1.803353
C -1.882007 -0.295362 0.979105
H -0.458511 1.518854 3.505859
H -1.729813 0.044399 4.869541
H -3.251598 -1.851522 5.106417
H -4.689979 -3.514278 4.104108
H -4.875966 -3.707115 1.650607
H -3.620017 -2.270912 0.197995
53
Rh -1.120749 0.592109 -2.006738
P -0.580254 0.960105 -4.298614
P -3.490641 1.353750 -2.197918
C 1.224249 1.072405 -4.398195
C -1.175438 -0.311035 -5.500938
C -4.421222 0.367067 -0.998033
C -3.851084 3.148865 -1.953691
C -1.299640 2.561640 -4.831888
C -4.146997 0.928317 -3.854619
N -2.298614 1.294342 1.882306
C -1.638017 0.492638 1.036214
C -0.572812 0.876463 0.204324
C 0.308700 -0.068969 -0.460554
C 1.766847 0.357820 -0.566883
C 0.148038 -1.568381 -0.193705
H -1.950159 -0.546240 1.053723
H 0.001018 -0.436481 -2.385642
H -0.221399 1.900858 0.263499
H 0.506694 -1.812402 0.815496
H 0.747442 -2.139560 -0.906826
H -0.886941 -1.909176 -0.292753
H 2.265495 -0.144617 -1.401496
H 2.298219 0.082986 0.354602
H 1.867656 1.436991 -0.705381
C -3.340260 0.765129 2.783928
H -3.547606 -0.275501 2.528673
H -4.260534 1.344528 2.672637
H -3.006928 0.819423 3.824318
C -1.959193 2.723640 2.016779
H -2.044468 3.226343 1.049597
H -0.938318 2.845833 2.392291
H -2.652955 3.186788 2.716983
C 1.886419 1.926184 -3.516009
C 3.284759 1.998661 -3.512349
C 4.019634 1.188968 -4.383226
C 3.363145 0.306602 -5.246875
C 1.963623 0.243954 -5.243314
H 1.309148 2.541135 -2.841805
H 3.799804 2.673571 -2.834150
H 5.103170 1.238176 -4.382748
H 3.939648 -0.333222 -5.909572
H 1.465170 -0.461378 -5.884875
C -1.863556 -1.443684 -5.040879
C -2.366635 -2.383823 -5.945897
C -2.191177 -2.203553 -7.318307
C -1.509396 -1.079516 -7.784812
C -1.010690 -0.132536 -6.884740
H -2.020455 -1.584798 -3.979283
H -2.902277 -3.248929 -5.578997
H -2.588509 -2.930401 -8.019850
H -1.381852 -0.927375 -8.848222
H -0.503888 0.747780 -7.258983
C -4.160058 -1.003049 -0.932738
C -4.868699 -1.817769 -0.041292
C -5.840130 -1.250298 0.789078
C -6.093021 0.124420 0.740287
C -5.372177 0.932383 -0.148636
H -3.415004 -1.429355 -1.585524
H -4.669403 -2.884298 0.007765
H -6.390575 -1.879389 1.483278
H -6.832083 0.564333 1.403708
H -5.538042 1.994100 -0.153253
C -2.806603 4.027666 -1.633766
C -3.048165 5.399228 -1.503413
C -4.332990 5.908412 -1.694552
C -5.376903 5.042473 -2.021566
C -5.138001 3.671492 -2.162120
H -1.802913 3.644720 -1.499865
H -2.233297 6.066033 -1.256922
H -4.518339 6.973224 -1.595188
H -6.372735 5.432509 -2.182374
H -5.949754 3.012638 -2.442693
C -0.453316 3.669252 -5.038413
C -0.934688 4.902756 -5.271923
C -2.313532 5.124911 -5.336489
C -2.814461 6.416570 -5.567096
C -4.138657 6.620003 -5.667756
C -5.037194 5.550582 -5.551220
C -4.595294 4.299901 -5.329651
C -3.211944 4.036210 -5.202868
C -2.704759 2.728407 -4.965397
H 0.613796 3.548696 -5.015792
H -0.245813 5.730553 -5.412313
H -2.137137 7.256111 -5.670666
H -4.519425 7.620042 -5.847597
H -6.101902 5.737396 -5.639310
H -5.322869 3.505106 -5.236154
C -5.144409 -0.061187 -3.975295
C -5.600916 -0.473253 -5.170594
C -5.102442 0.086377 -6.350563
C -5.575016 -0.352258 -7.598242
C -5.111166 0.202935 -8.730194
C -4.154357 1.226121 -8.684843
C -3.676765 1.671977 -7.509361
C -4.129039 1.115978 -6.289638
C -3.644000 1.554909 -5.025503
H -5.572307 -0.511973 -3.097399
H -6.365110 -1.243385 -5.216619
H -6.319808 -1.137262 -7.656187
H -5.482892 -0.138268 -9.690868
H -3.796239 1.661698 -9.611439
H -2.931984 2.455992 -7.521397
54
Rh -1.720969 0.515717 -0.134839
P -0.505520 -1.341837 0.660616
P 0.169671 1.875837 -0.643763
C -1.511322 -2.115888 1.953351
C -0.069185 -2.597141 -0.622094
C -0.424669 3.149254 -1.785324
C 1.054108 2.678161 0.766193
C 1.080796 -0.840666 1.434968
C 1.396730 0.836666 -1.517802
N -4.752329 -2.012825 0.356218
C -4.425750 -0.710735 0.449300
C -3.879966 0.070982 -0.573412
C -3.867589 1.582154 -0.533034
C -4.466632 2.197845 -1.819113
C -4.444704 2.243097 0.734000
H -4.642885 -0.258552 1.410880
H -2.751393 1.965438 -0.570097
H -3.840594 -0.359553 -1.570143
H -5.525636 2.077234 0.793272
H -4.272127 3.322860 0.698375
H -3.969884 1.862175 1.643104
H -4.261226 3.272473 -1.864084
H -5.551222 2.051831 -1.825626
H -4.046582 1.730818 -2.714565
C -5.427786 -2.714673 1.462367
H -5.409736 -2.088807 2.356157
H -4.911377 -3.652518 1.682299
H -6.467495 -2.935384 1.201418
C -4.512774 -2.768883 -0.882786
H -5.144825 -2.403828 -1.699311
H -4.734164 -3.820779 -0.705892
H -3.462627 -2.671890 -1.179464
C -2.072574 -1.292101 2.930592
C -2.814371 -1.844278 3.981952
C -2.996455 -3.229317 4.045288
C -2.455042 -4.057218 3.056271
C -1.720546 -3.494257 2.004653
H -1.917028 -0.226199 2.874510
H -3.248050 -1.204652 4.744952
H -3.572699 -3.662374 4.855129
H -2.618134 -5.130289 3.097635
H -1.333100 -4.129855 1.229775
C -0.512884 -2.425540 -1.941228
C -0.140962 -3.333031 -2.938472
C 0.679121 -4.419137 -2.631003
C 1.129581 -4.594929 -1.322426
C 0.767924 -3.684985 -0.323761
H -1.138140 -1.578518 -2.193305
H -0.488636 -3.188446 -3.952292
H 0.969216 -5.120929 -3.406578
H 1.774636 -5.428580 -1.080010
H 1.144993 -3.816577 0.682382
C -1.218843 2.754617 -2.863041
C -1.754388 3.709171 -3.736353
C -1.510149 5.066693 -3.508440
C -0.741418 5.469742 -2.411854
C -0.208131 4.506245 -1.545962
H -1.407880 1.703936 -3.025204
H -2.361406 3.402108 -4.583702
H -1.924841 5.811006 -4.179660
H -0.567943 6.526985 -2.230043
H 0.355831 4.821515 -0.685394
C 0.559169 2.541656 2.071528
C 1.253275 3.092360 3.153787
C 2.448206 3.782184 2.946001
C 2.947885 3.922140 1.651049
C 2.261056 3.368421 0.565986
H -0.357921 1.993507 2.245493
H 0.865046 2.973743 4.156286
H 2.989365 4.202805 3.787723

H 3.880289 4.444791 1.484906
H 2.669955 3.462710 -0.431872
C 1.251622 -1.001545 2.825343
C 2.352417 -0.571020 3.465218
C 3.384661 0.051781 2.758212
C 4.525176 0.517571 3.432543
C 5.522963 1.102404 2.749215
C 5.443580 1.254732 1.358263
C 4.367798 0.821471 0.677406
C 3.288964 0.201904 1.351274
C 2.129536 -0.263334 0.668668
H 0.490981 -1.482249 3.414195
H 2.436767 -0.708111 4.539116
H 4.609281 0.405959 4.507146
H 6.402370 1.458200 3.276031
H 6.261725 1.729087 0.827053
H 4.344145 0.969716 -0.393480
C 1.585516 0.995769 -2.904452
C 2.343660 0.147898 -3.621975
C 2.988355 -0.926399 -2.999728
C 3.768843 -1.818883 -3.752511
C 4.415675 -2.828445 -3.146114
C 4.327200 -3.003354 -1.758098
C 3.589056 -2.169768 -1.003861
C 2.880115 -1.099795 -1.596275
C 2.086728 -0.198637 -0.834243
H 1.117721 1.812112 -3.424664
H 2.453886 0.299630 -4.691671
H 3.859118 -1.696451 -4.825450
H 5.018859 -3.513379 -3.733053
H 4.860911 -3.822995 -1.289387
H 3.539674 -2.345343 0.062321

95

Rh 2.005957 0.664177 0.149487
P 0.071303 2.019888 0.671026
P 0.665712 -1.322535 -0.859006
C 0.560159 3.402220 1.744001
C -0.830098 2.682727 -0.798284
C 1.582045 -2.032444 -2.255520
C 0.355144 -2.652963 0.380153
C -1.112870 0.940444 1.565942
C -0.996912 -0.825300 -1.447060
N 4.425122 -1.847047 1.415549
C 4.270171 -0.583063 0.997792
C 4.027167 -0.123613 -0.317742
C 3.956501 1.291401 -0.634244
C 3.848346 1.680653 -2.096562
C 4.728912 2.337930 0.164987
H 2.601279 1.686762 1.135662
H 4.378192 0.158373 1.779837
H 3.987953 -0.830018 -1.140214
H 4.234897 3.310934 0.084564
H 5.739665 2.439303 -0.254152
H 4.821125 2.105603 1.227573
H 4.846068 1.908814 -2.498155
H 3.239037 2.582838 -2.209172
H 3.418095 0.887755 -2.707964
C 4.561032 -2.156132 2.852095
H 5.494637 -2.698210 3.029226
H 3.722281 -2.776352 3.184664
H 4.568388 -1.231275 3.430149
C 4.392080 -3.016613 0.525873
H 4.513860 -2.715341 -0.514003
H 3.445428 -3.557336 0.636281
H 5.210458 -3.691518 0.788232
C 1.297590 3.140602 2.903078
C 1.738916 4.190369 3.717778
C 1.458855 5.510565 3.355013
C 0.750551 5.782553 2.181102
C 0.312369 4.725349 1.373442
H 1.513003 2.119598 3.180076
H 2.298440 3.985212 4.626412
H 1.800382 6.327756 3.981098
H 0.551855 6.811505 1.893679
H -0.198597 4.944331 0.452485
C -0.319059 2.467956 -2.086676
C -1.018221 2.918640 -3.211064
C -2.233354 3.588171 -3.062305
C -2.747897 3.808219 -1.784456
C -2.055659 3.354665 -0.657082
H 0.615230 1.937044 -2.213496
H -0.617135 2.740056 -4.199447
H -2.778151 3.931819 -3.936019
H -3.695682 4.315369 -1.662994
H -2.474491 3.512123 0.328627
C 1.859031 -1.222614 -3.357066
C 2.611987 -1.714426 -4.430105
C 3.100686 -3.023550 -4.387049
C 2.841756 -3.835576 -3.278499
C 2.084736 -3.333668 -2.212399
H 1.470499 -0.216572 -3.379786
H 2.820532 -1.085294 -5.290734
H 3.689276 -3.408699 -5.212396
H 3.237565 -4.846686 -3.241621
H 1.919394 -3.948069 -1.344903
C 0.840708 -2.512027 1.688212
C 0.581365 -3.494432 2.649412
C -0.162873 -4.626448 2.315679
C -0.649916 -4.774320 1.016366
C -0.400656 -3.790698 0.053758
H 1.412526 -1.633842 1.959376
H 0.960079 -3.372676 3.654906
H -0.363659 -5.387115 3.063456
H -1.231638 -5.647316 0.753023
H -0.796191 -3.907181 -0.947103
C -1.288118 1.112450 2.952930
C -1.949510 0.217159 3.706650
C -2.509694 -0.923184 3.123790
C -3.185362 -1.865963 3.915765
C -3.767008 -2.934596 3.346023
C -3.717472 -3.119923 1.957668
C -3.080170 -2.238474 1.166911
C -2.435015 -1.108436 1.720101
C -1.747986 -0.155516 0.916949
H -0.889868 1.979275 3.445664
H -2.046960 0.382380 4.775692
H -3.248119 -1.734754 4.989652
H -4.289026 -3.658634 3.963013
H -4.199384 -3.986483 1.518330
H -3.060111 -2.422571 0.101369
C -1.346421 -1.043796 -2.794798
C -2.508255 -0.609805 -3.311852
C -3.433784 0.060812 -2.508019
C -4.644433 0.519063 -3.052836
C -5.550107 1.129028 -2.270475
C -5.305295 1.312589 -0.902844
C -4.157512 0.888639 -0.344794
C -3.166321 0.250724 -1.128195
C -1.934427 -0.204271 -0.576819
H -0.683792 -1.585964 -3.445003
H -2.730751 -0.791346 -4.359089
H -4.859027 0.377755 -4.105604
H -6.485196 1.477176 -2.697039
H -6.053673 1.802367 -0.289077
H -4.007828 1.058826 0.712606

96

Rh 1.439586 1.101524 -0.182236
P -0.802672 1.760443 0.321192
P 0.968542 -1.155071 -0.647139
C -0.647110 3.282354 1.285946
C -1.961960 2.030585 -1.090863
C 2.181350 -1.686213 -1.881277
C 1.027467 -2.287170 0.810713
C -1.543296 0.467955 1.380329
C -0.698885 -1.347635 -1.381367
N 4.640919 -0.380347 1.006755
C 3.915638 0.745815 0.861773
C 3.616052 1.444075 -0.324747
C 3.097681 2.865800 -0.283108
C 3.239028 3.560741 -1.651591
C 3.615115 3.762472 0.865199
H 1.927830 2.861069 -0.084856
H 3.566779 1.164356 1.799461
H 4.066780 1.129676 -1.260241
H 3.093324 4.724954 0.849966
H 4.686149 3.946762 0.737498
H 3.450248 3.316043 1.849622
H 4.294789 3.771362 -1.853847
H 2.693372 4.508943 -1.662162
H 2.846992 2.926356 -2.451827
C 4.842749 -0.991056 2.332493
H 5.910673 -1.025478 2.571695
H 4.449145 -2.013293 2.342830
H 4.328499 -0.403532 3.094139
C 5.335620 -1.042191 -0.107602
H 4.886018 -0.765133 -1.061442
H 5.254561 -2.126368 0.005837
H 6.395691 -0.767335 -0.123697
C 0.316276 3.325063 2.294942
C 0.530387 4.503186 3.020493
C -0.213747 5.646738 2.714425
C -1.160145 5.617156 1.684863
C -1.366063 4.433253 0.964728
H 0.889822 2.437781 2.518400
H 1.271586 4.534612 3.814465
H -0.051345 6.562848 3.271952
H -1.724357 6.513173 1.440544
H -2.070716 4.422433 0.151635
C -1.495053 1.923003 -2.409149
C -2.376195 2.061187 -3.486589
C -3.731363 2.304845 -3.260646
C -4.204506 2.411525 -1.952616
C -3.328989 2.267771 -0.871598
H -0.448839 1.716073 -2.595055
H -2.005118 1.968980 -4.498298
H -4.415186 2.404707 -4.097712
H -5.256282 2.587111 -1.770810
H -3.711303 2.327376 0.139394
C 2.430222 -0.838948 -2.962105
C 3.379940 -1.186323 -3.930562
C 4.088308 -2.385311 -3.803711
C 3.855378 -3.229575 -2.713243
C 2.903492 -2.872516 -1.749568
H 1.879960 0.085410 -3.045436
H 3.571673 -0.529822 -4.774448
H 4.828755 -2.657319 -4.547781
H 4.422230 -4.150404 -2.608713
H 2.756402 -3.503020 -0.891694
C 1.388010 -1.782374 2.068171
C 1.382833 -2.612319 3.194083
C 1.015035 -3.953024 3.077893
C 0.648153 -4.462360 1.831973
C 0.643481 -3.633966 0.704963
H 1.658268 -0.739954 2.172980
H 1.663022 -2.209507 4.157643
H 1.008914 -4.595090 3.952964
H 0.350428 -5.497788 1.737465
H 0.332617 -4.033359 -0.251908
C -1.724258 0.715289 2.754534
C -2.111929 -0.250497 3.606172
C -2.359579 -1.547101 3.142322
C -2.750912 -2.557832 4.035430
C -3.016575 -3.794589 3.582529
C -2.912560 -4.094016 2.217060
C -2.539899 -3.153178 1.331316
C -2.239967 -1.839661 1.759385
C -1.838629 -0.818099 0.855213
H -1.547279 1.698122 3.154730
H -2.229499 -0.020883 4.661030
H -2.845556 -2.346367 5.094018
H -3.320254 -4.570593 4.277521
H -3.134420 -5.098592 1.873645
H -2.461949 -3.428467 0.288226
C -0.815114 -1.722313 -2.734830
C -2.004368 -1.788268 -3.357814
C -3.183116 -1.489626 -2.667853
C -4.422560 -1.543082 -3.326163
C -5.555473 -1.269948 -2.657917
C -5.519677 -0.930614 -1.298525
C -4.352285 -0.868644 -0.633739
C -3.131371 -1.144030 -1.293087
C -1.874889 -1.082399 -0.627404
H 0.063030 -1.965966 -3.306629
H -2.046537 -2.073794 -4.404628
H -4.473774 -1.807717 -4.375748
H -6.510032 -1.315317 -3.171939
H -6.447094 -0.715127 -0.778927
H -4.370385 -0.592272 0.411586

102

Rh 2.015924 0.094623 -0.803671
P 0.200799 -1.844360 -0.285772
P 0.307426 1.802432 -0.693498
C 0.576488 -3.258945 -1.357915
C 0.082229 -2.435089 1.461050
C 0.906215 3.514047 -0.652347
C -1.027502 1.818165 -1.969348
C -1.517412 -1.307330 -0.677359
C -0.408371 1.435546 0.949232
N 4.166851 1.115354 1.933114
C 4.135850 0.674708 0.668430
C 3.852848 -0.648642 0.210398
C 3.998761 -1.061398 -1.141056
C 3.895592 -2.542624 -1.435462
C 4.800688 -0.311735 -2.190807
H 4.475245 1.400575 -0.056449
H 2.850847 1.247843 -1.398452
H 3.615009 -1.418198 0.934774
H 4.282714 -0.344926 -3.155014
H 4.981297 0.736803 -1.950830
H 5.773204 -0.806265 -2.326779
H 3.423882 -2.720354 -2.407707
H 4.897976 -2.988791 -1.485248
H 3.326170 -3.080543 -0.672230
C 3.870796 0.296638 3.119760
H 4.584379 0.552632 3.906797
H 2.858220 0.503849 3.484153
H 3.973439 -0.763358 2.894708
C 4.405449 2.545340 2.211425
H 3.539119 2.976166 2.724779
H 5.288486 2.664100 2.845265
H 4.557540 3.084581 1.275055
C 0.673142 -3.045343 -2.734054
C 0.990044 -4.102339 -3.596098
C 1.232083 -5.374185 -3.068382
C 1.165208 -5.588650 -1.688117
C 0.842698 -4.525573 -0.835013
H 0.496613 -2.057813 -3.127302
H 1.055706 -3.938313 -4.667910
H 1.483953 -6.195471 -3.730277
H 1.374110 -6.574198 -1.281242
H 0.831394 -4.685155 0.228972
C 0.907175 -1.872874 2.442205
C 0.801565 -2.275108 3.777293
C -0.139845 -3.235144 4.149172
C -0.980250 -3.788047 3.181950
C -0.879791 -3.384002 1.846513
H 1.607294 -1.100861 2.166456
H 1.443364 -1.827185 4.523615
H -0.225621 -3.541844 5.186747
H -1.721262 -4.523086 3.465738
H -1.548824 -3.805852 1.107499
C 1.897708 3.911347 -1.549333
C 2.357768 5.234350 -1.557793
C 1.795543 6.167883 -0.682095
C 0.760856 5.790617 0.178870
C 0.307210 4.465785 0.177941
H 2.279530 3.210119 -2.270649
H 3.132602 5.542957 -2.254070
H 2.144524 7.194665 -0.689933
H 0.303427 6.527821 0.832866
H -0.521589 4.187063 0.808215
C -0.964483 0.942557 -3.062426
C -1.968887 0.947922 -4.035982
C -3.039749 1.836646 -3.936305
C -3.099895 2.726061 -2.863521
C -2.099133 2.722446 -1.886256
H -0.135410 0.254748 -3.151028
H -1.913692 0.259538 -4.868118
H -3.821046 1.837614 -4.689755
H -3.928972 3.415560 -2.778182
H -2.165140 3.409551 -1.053473
C -2.264703 -2.016206 -1.640499
C -3.511245 -1.651767 -1.989094
C -4.124205 -0.550773 -1.383050
C -5.423506 -0.168080 -1.754330
C -6.018410 0.876343 -1.153798
C -5.356594 1.596025 -0.149121
C -4.111837 1.260043 0.233508
C -3.441186 0.172554 -0.372782
C -2.122634 -0.209272 -0.002298
H -1.850804 -2.888169 -2.114208
H -4.046395 -2.222922 -2.741638
H -5.955805 -0.714263 -2.524116
H -7.023436 1.165242 -1.443651
H -5.857650 2.433762 0.323963
H -3.633272 1.846207 1.006441
C 0.295895 1.923127 2.066202
C -0.061350 1.623781 3.326207
C -1.130068 0.755479 3.568529
C -1.496635 0.434148 4.885503
C -2.496509 -0.432614 5.116378
C -3.182697 -1.032631 4.051798
C -2.860000 -0.751480 2.776872
C -1.822820 0.163331 2.480204
C -1.443603 0.482598 1.145149
H 1.167756 2.541512 1.909947
H 0.503682 2.036506 4.156417
H -0.973253 0.879276 5.723427
H -2.774772 -0.677080 6.136210
H -3.980634 -1.736480 4.261955
H -3.407121 -1.248704 1.988229

107

Rh 1.902011 -0.036222 -0.500015
P 0.195018 -1.776414 -0.286159
P 0.296807 1.753182 -0.482737
C 0.613150 -3.170525 -1.367107
C 0.048930 -2.385700 1.451532
C 1.021233 3.409555 -0.422845
C -0.932505 1.849356 -1.856642
C -1.514616 -1.243336 -0.718939
C -0.566410 1.439077 1.094490
N 4.530034 1.373690 1.372697
C 4.204142 0.722784 0.170185
C 3.913497 -0.734310 0.183515
C 3.832694 -1.496133 -0.982174
C 3.865115 -3.004191 -0.844372
C 4.162387 -0.979428 -2.368144
H 4.876241 1.030344 -0.634189
H 3.130214 1.228789 -0.303449
H 3.909399 -1.249807 1.135543
H 3.481010 -1.418074 -3.104475
H 4.098133 0.106310 -2.460307
H 5.179267 -1.293091 -2.645927
H 3.399633 -3.505329 -1.697138
H 4.911431 -3.336100 -0.822503
H 3.385839 -3.351681 0.075176
C 4.851819 2.803087 1.254267
H 3.941683 3.422677 1.158608
H 5.402444 3.130788 2.137203
H 5.476831 2.971611 0.374124
C 3.836932 0.988495 2.607759
H 4.035020 -0.053307 2.864190
H 4.205594 1.610456 3.423909
H 2.746143 1.127561 2.519119
C 0.842032 -2.914333 -2.719973
C 1.166436 -3.957909 -3.595379
C 1.275817 -5.262157 -3.103576
C 1.069539 -5.522540 -1.745035
C 0.742462 -4.471789 -0.878169
H 0.759934 -1.904469 -3.086287
H 1.337567 -3.759308 -4.649612
H 1.531224 -6.073943 -3.775813
H 1.172546 -6.535176 -1.364933
H 0.616530 -4.672330 0.170596
C 0.895848 -1.871222 2.443404
C 0.758730 -2.276246 3.774743
C -0.228570 -3.194798 4.132276
C -1.082473 -3.705574 3.154216
C -0.955096 -3.296079 1.822999
H 1.648967 -1.142114 2.181301
H 1.416270 -1.867281 4.529914
H -0.336932 -3.504428 5.166953
H -1.857847 -4.408623 3.427186
H -1.638934 -3.680507 1.077100
C 2.164320 3.674311 -1.174996
C 2.725720 4.957424 -1.182063
C 2.115161 5.982929 -0.453181
C 0.936687 5.736054 0.258387
C 0.382963 4.449608 0.256374
H 2.595522 2.893713 -1.779970
H 3.620571 5.162926 -1.762753
H 2.543211 6.979318 -0.460255
H 0.448668 6.543788 0.796680
H -0.545563 4.264005 0.772134
C -0.783389 1.038719 -2.990537
C -1.713563 1.097843 -4.033633
C -2.795585 1.975627 -3.961696
C -2.940436 2.801382 -2.846919
C -2.012603 2.745593 -1.801716
H 0.055187 0.360449 -3.057499
H -1.591578 0.459927 -4.898221
H -3.519404 2.018033 -4.769400

H -3.778668 3.482072 -2.782988
H -2.145623 3.381680 -0.937282
C -2.191999 -1.897235 -1.769255
C -3.425307 -1.534561 -2.162097
C -4.092727 -0.485384 -1.524193
C -5.372530 -0.095238 -1.951242
C -6.015766 0.907899 -1.330935
C -5.423824 1.577413 -0.251118
C -4.200437 1.231726 0.187537
C -3.481811 0.183079 -0.433180
C -2.181593 -0.206057 -0.005880
H -1.730054 -2.719819 -2.284578
H -3.903396 -2.061111 -2.982545
H -5.849754 -0.600763 -2.782497
H -7.004956 1.203555 -1.665137
H -5.962096 2.384177 0.234837
H -3.777285 1.780993 1.017514
C -0.000216 1.980345 2.263781
C -0.443803 1.655539 3.490154
C -1.472533 0.720448 3.644540
C -1.928078 0.372745 4.926602
C -2.903062 -0.539784 5.073114
C -3.476473 -1.159607 3.954370
C -3.065140 -0.855258 2.710653
C -2.045516 0.102137 2.502908
C -1.576059 0.447506 1.204673
H 0.839149 2.654390 2.183909
H 0.019031 2.103081 4.364626
H -1.496612 0.837155 5.805511
H -3.251633 -0.803051 6.066391
H -4.259098 -1.896861 4.096859
H -3.525939 -1.367291 1.877202

113

Rh 1.848286 0.008328 -0.553808
P 0.213340 -1.728918 -0.300734
P 0.206709 1.767668 -0.514551
C 0.662010 -3.119925 -1.372067
C 0.114617 -2.309129 1.450493
C 0.929330 3.423356 -0.462801
C -1.047600 1.855447 -1.865349
C -1.517659 -1.251559 -0.716518
C -0.618229 1.442032 1.078997
N 4.299797 1.285610 1.615264
C 4.102660 0.813871 0.250147
C 3.839420 -0.660816 0.158082
C 3.840705 -1.377946 -1.037048
C 3.916450 -2.888140 -0.970929
C 4.194663 -0.778968 -2.383047
H 4.887677 1.144417 -0.458693
H 3.174586 1.382843 -0.141484
H 3.799458 -1.202244 1.096430
H 3.565843 -1.214317 -3.166675
H 4.082600 0.307314 -2.421225
H 5.237275 -1.022242 -2.634292
H 3.466275 -3.365687 -1.845186
H 4.973070 -3.186891 -0.964549
H 3.452921 -3.292238 -0.066476
C 5.538018 0.773364 2.240023
H 6.450375 1.159022 1.750537
H 5.552179 1.076519 3.288931
H 5.560258 -0.317934 2.202268
C 4.159782 2.744589 1.752880
H 3.168810 3.054096 1.403154
H 4.242954 3.016291 2.806481
H 4.918118 3.312248 1.183802
C 0.864010 -2.870324 -2.730633
C 1.201894 -3.913527 -3.601473
C 1.354557 -5.209454 -3.099085
C 1.177694 -5.461756 -1.734796
C 0.835040 -4.411911 -0.872826
H 0.748869 -1.866282 -3.104885
H 1.350572 -3.720784 -4.660179
H 1.620982 -6.020700 -3.767659
H 1.314447 -6.467354 -1.346784
H 0.730544 -4.604908 0.179668
C 0.936162 -1.726153 2.427017
C 0.828902 -2.111445 3.767007
C -0.105517 -3.073729 4.150074
C -0.938369 -3.647516 3.189112
C -0.840466 -3.259871 1.848950
H 1.639996 -0.953687 2.149416
H 1.465605 -1.649084 4.509154
H -0.191733 -3.366215 5.191747
H -1.675572 -4.382690 3.482275
H -1.509842 -3.692903 1.116674
C 2.082291 3.678830 -1.202257
C 2.653980 4.957340 -1.201173
C 2.044502 5.986190 -0.475559
C 0.859318 5.746347 0.227727
C 0.296272 4.463998 0.219030
H 2.515323 2.891628 -1.798194
H 3.556089 5.156961 -1.772579
H 2.480151 6.979298 -0.476684
H 0.374822 6.555714 0.766763
H -0.632721 4.280012 0.734872
C -0.910912 1.049961 -3.004601
C -1.862176 1.100920 -4.028985
C -2.953688 1.964475 -3.932434
C -3.087306 2.784081 -2.811726
C -2.138459 2.736462 -1.785121
H -0.066504 0.380752 -3.089797
H -1.749551 0.467023 -4.897761
H -3.693939 2.000174 -4.725419
H -3.933094 3.453185 -2.728455
H -2.263204 3.366720 -0.915216
C -2.184474 -1.929756 -1.758224
C -3.432211 -1.607011 -2.140412
C -4.125878 -0.576745 -1.499628
C -5.420313 -0.226530 -1.917119
C -6.088083 0.759077 -1.294606
C -5.507385 1.449789 -0.222035
C -4.270656 1.142182 0.207475
C -3.526496 0.112996 -0.415567
C -2.212097 -0.236223 0.002325
H -1.701981 -2.738949 -2.275649
H -3.901212 -2.150687 -2.954877
H -5.888964 -0.748786 -2.742866
H -7.088337 1.024014 -1.621493
H -6.065240 2.242040 0.265658
H -3.856807 1.706863 1.031795
C -0.037083 1.986589 2.239339
C -0.464825 1.663608 3.472016
C -1.485070 0.721661 3.641608
C -1.921273 0.372921 4.930110
C -2.881346 -0.552894 5.091273
C -3.457336 -1.186610 3.981607
C -3.064797 -0.881252 2.732067
C -2.063651 0.092017 2.508967
C -1.614332 0.439495 1.204323
H 0.800987 2.659632 2.147665
H 0.008424 2.113780 4.339406
H -1.485317 0.845969 5.802122
H -3.214433 -0.817396 6.089483
H -4.226387 -1.935644 4.135959
H -3.526069 -1.404596 1.906004

114

Rh 1.676031 0.300109 -1.004768
P 0.406843 -1.502121 -0.127580
P -0.044190 1.987637 -0.692297
C 1.038178 -3.047072 -0.835294
C 0.457953 -1.608725 1.715068
C 0.826012 3.572882 -0.626234
C -1.353856 2.042790 -1.990147
C -1.373497 -1.341555 -0.553202
C -0.888673 1.721400 0.904998
N 4.123190 -1.062209 1.659054
C 4.385012 -0.306648 0.422238
C 3.661421 -0.861884 -0.788602
C 3.531494 -0.240929 -2.041166
C 3.463339 -1.008368 -3.340767
C 3.663716 1.258242 -2.144943
H 5.473664 -0.292738 0.183931
H 4.091779 0.732595 0.600617
H 3.563009 -1.944382 -0.804513
H 2.699814 1.783808 -1.793480
H 4.444247 1.693184 -1.518300
H 3.764213 1.597847 -3.176328
H 2.627413 -0.678956 -3.966719
H 4.388855 -0.841158 -3.906814
H 3.378527 -2.082964 -3.167553
C 4.673938 -2.430196 1.653884
H 5.776784 -2.447147 1.574359
H 4.385647 -2.933621 2.579482
H 4.264673 -3.011106 0.822143
C 4.547683 -0.313550 2.859172
H 4.058982 0.665048 2.876465
H 4.246826 -0.866625 3.752169
H 5.641488 -0.158101 2.901543
C 1.089170 -3.177510 -2.223641
C 1.607107 -4.338933 -2.810177
C 2.106865 -5.359258 -1.995803
C 2.097632 -5.217689 -0.604840
C 1.569396 -4.055362 -0.029169
H 0.721493 -2.372789 -2.842400
H 1.633229 -4.445057 -3.891019
H 2.516144 -6.257820 -2.444233
H 2.509840 -6.002128 0.023731
H 1.608332 -3.929250 1.038645
C 1.142769 -0.634452 2.454184
C 1.127034 -0.662480 3.852929
C 0.429246 -1.663342 4.528649
C -0.263116 -2.632068 3.802043
C -0.261443 -2.599525 2.403729
H 1.661352 0.163550 1.939676
H 1.654961 0.099499 4.408721
H 0.416238 -1.682285 5.613642
H -0.819640 -3.401340 4.319746
H -0.825781 -3.339237 1.850527
C 1.954479 3.671996 0.189585
C 2.692034 4.861127 0.237163
C 2.302800 5.946604 -0.553850
C 1.189796 5.842797 -1.394656
C 0.458655 4.648476 -1.434673
H 2.249418 2.823702 0.789099
H 3.564278 4.943056 0.879553
H 2.871780 6.869369 -0.523511
H 0.902301 6.683116 -2.020593
H -0.376974 4.560306 -2.106248
C -1.288635 1.167829 -3.084462
C -2.310131 1.150590 -4.039942
C -3.406703 2.004034 -3.913233
C -3.480183 2.876528 -2.827148
C -2.465720 2.891756 -1.864498
H -0.451069 0.488810 -3.182411
H -2.251456 0.466454 -4.875471
H -4.201381 1.985313 -4.652369
H -4.333910 3.531388 -2.716489
H -2.546087 3.553565 -1.011627
C -1.958262 -2.261252 -1.447204
C -3.218342 -2.123605 -1.894518
C -4.010476 -1.056365 -1.463086
C -5.321109 -0.905750 -1.944944
C -6.092188 0.101773 -1.503344
C -5.605227 1.012096 -0.555486
C -4.355735 0.900467 -0.070890
C -3.501421 -0.137966 -0.510598
C -2.171686 -0.288031 -0.025201
H -1.402514 -3.113319 -1.789799
H -3.622474 -2.850543 -2.592705
H -5.720686 -1.603406 -2.671498
H -7.104752 0.211178 -1.877720
H -6.246376 1.816163 -0.210791
H -4.017030 1.631132 0.650930
C -0.662947 2.617437 1.968107
C -1.150093 2.396409 3.201647
C -1.914778 1.255731 3.467215
C -2.408576 1.017630 4.760208
C -3.161888 -0.067120 5.007028
C -3.468819 -0.973034 3.982320
C -3.014306 -0.779220 2.731532
C -2.212321 0.343565 2.422366
C -1.708752 0.581867 1.113192
H -0.084951 3.510493 1.809296
H -0.944467 3.107980 3.995633
H -2.188537 1.708092 5.565822
H -3.541625 -0.244643 6.007848
H -4.079220 -1.841605 4.204400
H -3.264042 -1.509035 1.973379

116

Rh 1.963999 0.032602 -0.517442
P 0.109489 -1.879865 -0.123455
P 0.303177 1.767465 -0.479551
C 0.559065 -3.287516 -1.175399
C -0.163231 -2.493414 1.600712
C 0.928216 3.466779 -0.371187
C -0.918850 1.822760 -1.862702
C -1.561927 -1.319038 -0.654513
C -0.557833 1.394740 1.091297
N 4.556009 1.378258 1.287968
C 3.645397 0.384323 1.226831
C 3.693444 -0.892796 0.540267
C 4.023580 -1.239221 -0.782536
C 3.957695 -2.714577 -1.130845
C 4.796285 -0.450846 -1.824065
H 2.895134 1.164222 -1.003189
H 2.912308 0.449698 2.024759
H 3.319883 -1.715752 1.142616
H 4.341439 -0.609872 -2.807487
H 4.822413 0.620008 -1.649221
H 5.825507 -0.834859 -1.884527
H 3.542317 -2.867333 -2.131934
H 4.970063 -3.139575 -1.140957
H 3.361142 -3.288566 -0.416882
C 5.858948 1.373269 0.612071
H 5.844177 1.989742 -0.294192
H 6.604145 1.784866 1.297973
H 6.139533 0.353547 0.355277
C 4.182099 2.641426 1.945471
H 3.397581 2.458843 2.682589
H 5.051040 3.069825 2.447537
H 3.806581 3.361223 1.204888
C 0.771001 -3.061928 -2.536532
C 1.144430 -4.114814 -3.380866
C 1.328537 -5.394291 -2.848182
C 1.147022 -5.620736 -1.480173
C 0.767573 -4.561901 -0.645370
H 0.638663 -2.068659 -2.932769
H 1.299338 -3.941583 -4.441999
H 1.624395 -6.212334 -3.495742
H 1.310734 -6.612542 -1.067834
H 0.665521 -4.731659 0.412297
C 0.596011 -1.982913 2.662853
C 0.361464 -2.409263 3.974056
C -0.639063 -3.343409 4.241945
C -1.409088 -3.848432 3.194142
C -1.181718 -3.420319 1.882342
H 1.352418 -1.238081 2.473948
H 0.952886 -2.002786 4.783240
H -0.822935 -3.669018 5.260890
H -2.193715 -4.565022 3.396237
H -1.797472 -3.806214 1.080051
C 2.003660 3.852096 -1.171296
C 2.489233 5.164960 -1.123356
C 1.868251 6.102247 -0.292711
C 0.751456 5.739262 0.465615
C 0.273881 4.424032 0.409645
H 2.438864 3.149225 -1.859662
H 3.331970 5.462314 -1.741237
H 2.237444 7.121344 -0.257467
H 0.250514 6.480370 1.082237
H -0.614693 4.157107 0.958299
C -0.779120 0.958312 -2.957631
C -1.698576 0.991716 -4.011230
C -2.759332 1.897697 -3.990079
C -2.894417 2.775994 -2.914969
C -1.979009 2.744244 -1.857809
H 0.042913 0.256972 -2.985398
H -1.585205 0.311873 -4.844512
H -3.474759 1.920618 -4.806022
H -3.716356 3.478763 -2.890514
H -2.103670 3.422729 -1.024751
C -2.234508 -2.008851 -1.684057
C -3.440970 -1.621631 -2.134431
C -4.086464 -0.515521 -1.572781
C -5.343529 -0.109332 -2.049455
C -5.971824 0.939109 -1.491393
C -5.387475 1.639605 -0.426859
C -4.184931 1.280982 0.056887
C -3.481206 0.188699 -0.501199
C -2.204940 -0.216892 -0.023682
H -1.794630 -2.883700 -2.128351
H -3.918674 -2.178263 -2.935013
H -5.816457 -0.640586 -2.866986
H -6.944108 1.246172 -1.862809
H -5.914637 2.480869 0.010237
H -3.765322 1.852744 0.873619
C 0.044773 1.871410 2.270740
C -0.422773 1.560922 3.491367
C -1.513202 0.696712 3.630261
C -1.996230 0.365597 4.906676
C -3.020719 -0.492870 5.039948
C -3.617855 -1.073837 3.913018
C -3.182553 -0.782924 2.674289
C -2.113674 0.122769 2.479363
C -1.616552 0.451801 1.186092
H 0.923768 2.494322 2.198453
H 0.065725 1.965423 4.372773
H -1.544188 0.796933 5.792035
H -3.389240 -0.744800 6.028905
H -4.438401 -1.770684 4.044993
H -3.664458 -1.265383 1.835489

123

Rh 1.796796 -0.000020 -0.320080
P 0.136305 -1.763778 -0.176415
P 0.162167 1.751765 -0.361147
C 0.665494 -3.143398 -1.225777
C -0.101413 -2.372596 1.551802
C 0.851477 3.418368 -0.222646
C -0.995049 1.853072 -1.796335
C -1.555773 -1.277998 -0.719140
C -0.774576 1.394233 1.163686
N 5.165140 1.447163 0.692002
C 3.902294 0.809457 0.789626
C 3.747471 -0.672141 0.599656
C 3.826061 -1.364452 -0.609086
C 3.902476 -2.877913 -0.544631
C 4.205555 -0.781747 -1.957191
H 3.130144 1.267433 -0.059104
H 3.420635 1.126290 1.717081
H 3.650980 -1.259116 1.506964
H 3.554092 -1.195351 -2.734629
H 4.135847 0.305394 -2.000591
H 5.233557 -1.073060 -2.215884
H 3.502501 -3.347228 -1.447600
H 4.955256 -3.181592 -0.476865
H 3.381836 -3.287338 0.325699
C 6.251993 0.951383 -0.152009
H 6.177097 1.283297 -1.198781
H 7.197178 1.323115 0.252970
H 6.274573 -0.139160 -0.126714
C 5.188210 2.881306 0.993006
H 4.480124 3.102508 1.797664
H 6.188936 3.171885 1.320913
H 4.917720 3.495589 0.118661
C 0.964636 -2.880414 -2.563556
C 1.379764 -3.912288 -3.414186
C 1.510819 -5.210336 -2.911260
C 1.236010 -5.475878 -1.565874
C 0.817709 -4.437116 -0.724149
H 0.865756 -1.874658 -2.938058
H 1.604950 -3.709097 -4.457338
H 1.836723 -6.012784 -3.563942
H 1.356570 -6.482841 -1.175998
H 0.639302 -4.639793 0.316641
C 0.649408 -1.818128 2.598719
C 0.436736 -2.227135 3.919060
C -0.533079 -3.186468 4.211037
C -1.294725 -3.733851 3.178373
C -1.091101 -3.322477 1.857386
H 1.381796 -1.050884 2.389326
H 1.019842 -1.787745 4.717041
H -0.700890 -3.498161 5.237133
H -2.057813 -4.467777 3.399944
H -1.704785 -3.736550 1.067605
C 2.035239 3.718645 -0.893043
C 2.564175 5.014925 -0.848860
C 1.886051 6.015037 -0.144933
C 0.673280 5.730092 0.490533
C 0.150592 4.431972 0.434691
H 2.521929 2.957088 -1.480393
H 3.486576 5.250394 -1.372318
H 2.289718 7.021050 -0.112470
H 0.134923 6.517772 1.010235
H -0.802768 4.217192 0.890375
C -0.778145 1.057119 -2.929972
C -1.653799 1.118129 -4.019117
C -2.748286 1.982873 -3.993635
C -2.960799 2.793207 -2.878405
C -2.088032 2.735213 -1.786886
H 0.069500 0.387107 -2.960467
H -1.479894 0.491582 -4.883146
H -3.429732 2.026700 -4.837322

H -3.809460 3.463284 -2.850408
H -2.274357 3.358395 -0.923039
C -2.146020 -1.946140 -1.811926
C -3.359970 -1.613345 -2.284032
C -4.094038 -0.583004 -1.689568
C -5.352665 -0.223617 -2.198615
C -6.060391 0.760588 -1.619457
C -5.557600 1.440586 -0.501582
C -4.357089 1.124196 0.015435
C -3.573161 0.096666 -0.559447
C -2.294791 -0.261748 -0.048479
H -1.630337 -2.755557 -2.296281
H -3.770152 -2.149386 -3.134549
H -5.761505 -0.737832 -3.060422
H -7.032766 1.032542 -2.017096
H -6.146812 2.231641 -0.050156
H -4.002703 1.680504 0.872519
C -0.271086 1.913243 2.371389
C -0.788199 1.575452 3.565191
C -1.827370 0.642412 3.646508
C -2.357958 0.279293 4.895012
C -3.335269 -0.639144 4.972889
C -3.835629 -1.250949 3.815354
C -3.351990 -0.931167 2.601898
C -2.329233 0.035746 2.465666
C -1.784758 0.398272 1.201763
H 0.575705 2.582661 2.349061
H -0.376256 2.009347 4.471399
H -1.981840 0.735235 5.803276
H -3.741641 -0.914755 5.940527
H -4.620371 -1.994535 3.902870
H -3.757903 -1.437891 1.737329

TS$_{1-4}$

Rh 0.652778 -3.083177 -2.921898
P 1.709014 -0.934099 -3.036791
P -1.381151 -2.280162 -1.995894
C 3.480043 -1.261828 -3.234140
C 1.148263 0.225836 -4.360532
C -2.717392 -3.330250 -2.628090
C -1.419219 -2.274746 -0.149025
C 1.453644 -0.092981 -1.433529
C -1.758450 -0.548749 -2.481708
N 0.392115 -5.252318 -2.749156
C 1.878743 -5.565327 -2.828359
C 2.504197 -4.768738 -3.938043
C 3.461907 -5.130286 -4.812341
C 4.001508 -4.154253 -5.832168
C 4.092842 -6.504634 -4.848428
H 2.303967 -5.283241 -1.858085
H 2.016294 -6.646779 -2.955319
H 2.172172 -3.709106 -4.044828
H 4.046575 -6.916139 -5.863645
H 5.155317 -6.435789 -4.581758
H 3.625154 -7.219106 -4.168051
H 5.076087 -3.991332 -5.681859
H 3.873451 -4.538468 -6.850416
H 3.504067 -3.180189 -5.766921
C -0.166797 -5.789554 -1.470279
H 0.378774 -5.366729 -0.627726
H -1.220865 -5.517427 -1.397728
H -0.086050 -6.884135 -1.444573
C -0.320388 -5.871914 -3.914190
H 0.008560 -5.399992 -4.838841
H -0.113938 -6.949524 -3.954445
H -1.394122 -5.729730 -3.794223
C 4.066023 -2.243679 -2.433836
C 5.415989 -2.577970 -2.594481
C 6.175456 -1.930409 -3.573462
C 5.588188 -0.962660 -4.394667
C 4.235816 -0.637709 -4.227308
H 3.465646 -2.749466 -1.693048
H 5.871588 -3.341619 -1.970674
H 7.220509 -2.187980 -3.705111
H 6.177659 -0.475931 -5.166643
H 3.779217 0.081206 -4.884033
C 0.208988 -0.200678 -5.310629
C -0.271014 0.686010 -6.280259
C 0.179433 2.006239 -6.311226
C 1.113670 2.438759 -5.369820
C 1.592495 1.557871 -4.394755
H -0.158726 -1.218443 -5.285210
H -0.999832 0.346222 -7.003384
H -0.198652 2.694721 -7.060348
H 1.457166 3.464357 -5.380425
H 2.299276 1.910392 -3.654495
C -2.839104 -3.488066 -4.009903
C -3.846667 -4.299428 -4.545809
C -4.721407 -4.971166 -3.686514
C -4.587686 -4.839090 -2.300629
C -3.579939 -4.020365 -1.774921
H -2.146224 -2.977111 -4.661284
H -3.945143 -4.416303 -5.621293
H -5.499819 -5.605767 -4.095570
H -5.258744 -5.377419 -1.637117
H -3.458354 -3.948518 -0.708807
C -0.284600 -2.673131 0.572517
C -0.274738 -2.611864 1.969820
C -1.393503 -2.146591 2.661529
C -2.522731 -1.737421 1.951694
C -2.533549 -1.787362 0.554010
H 0.599293 -3.007261 0.045066
H 0.609135 -2.915956 2.513739
H -1.382070 -2.094176 3.745632
H -3.387838 -1.362184 2.481526
H -3.404623 -1.441214 0.012499
C 2.517029 -0.007048 -0.513993
C 2.338262 0.439589 0.741921
C 1.071685 0.844865 1.176159
C 0.882270 1.293861 2.493299
C -0.329988 1.708211 2.898453
C -1.419382 1.703359 2.016186
C -1.277197 1.280595 0.747461
C -0.024302 0.826591 0.276101
C 0.166253 0.368893 -1.056596
H 3.508171 -0.307092 -0.803747
H 3.183336 0.480195 1.422687
H 1.712389 1.313497 3.189578
H -0.469439 2.055841 3.916832
H -2.388397 2.044059 2.364308
H -2.145074 1.282727 0.101869
C -2.884543 -0.285075 -3.289081
C -3.165797 0.947057 -3.746455
C -2.346248 2.029267 -3.415940
C -2.631836 3.312093 -3.911309
C -1.855212 4.355208 -3.574730
C -0.754154 4.181824 -2.725150
C -0.449211 2.969023 -2.230494
C -1.233245 1.837725 -2.558848
C -0.939723 0.535973 -2.062611
H -3.560413 -1.076315 -3.555225
H -4.037643 1.099944 -4.375421
H -3.481473 3.469574 -4.565192
H -2.082370 5.344071 -3.959430
H -0.141628 5.038580 -2.465670
H 0.416061 2.879404 -1.588104

TS$_{1-33}$

Rh 0.539278 -0.075014 2.533176
P 2.380482 1.535475 2.928246
P 0.863968 -1.057060 4.666147
C 3.143766 1.838316 1.311873
C 1.860467 3.161849 3.637226
C -0.346915 -2.312961 5.279005
C 2.474659 -1.972535 4.759877
C 3.732650 0.929253 4.033117
C 0.993573 0.403753 5.794520
N -0.878112 -1.448781 1.645840
C -1.000371 -0.661953 0.358360
C -0.884175 0.803798 0.706482
C -0.181930 1.735389 -0.002825
C -0.321337 3.205661 0.320935
C 0.616087 1.433192 -1.249247
H -1.954570 -0.902665 -0.134359
H -0.193557 -0.978749 -0.304277
H -1.574748 1.167034 1.465823
H 1.465793 2.117158 -1.340341
H 0.999172 0.411125 -1.286087
H -0.010607 1.591190 -2.136733
H 0.654795 3.654635 0.544219
H -0.709419 3.741968 -0.554472
H -0.995489 3.385254 1.160191
C -2.218174 -1.582692 2.299455
H -2.940976 -2.019966 1.598941
H -2.132420 -2.245644 3.158851
H -2.570438 -0.608336 2.635574
C -0.284377 -2.800435 1.400829
H 0.706933 -2.682009 0.962817
H -0.191529 -3.321884 2.354512
H -0.923507 -3.390746 0.731371
C 3.451398 0.737258 0.511750
C 4.086648 0.913128 -0.723847
C 4.398645 2.203908 -1.161897
C 4.073804 3.312961 -0.374032
C 3.444638 3.125472 0.863467
H 3.205249 -0.252453 0.860251
H 4.332250 0.055481 -1.343505
H 4.886332 2.346615 -2.119911
H 4.308656 4.314484 -0.723666
H 3.189553 3.981384 1.461992
C 0.497427 3.455968 3.780591
C 0.088243 4.664099 4.354741
C 1.034582 5.584656 4.805673
C 2.393247 5.295094 4.679297
C 2.807112 4.089125 4.104896
H -0.244526 2.738248 3.456005
H -0.966643 4.878131 4.458222
H 0.715765 6.517844 5.259103
H 3.131906 5.998087 5.039981
H 3.864030 3.868806 4.027811
C -0.006309 -3.672477 5.182261
C -0.875184 -4.675079 5.626870
C -2.117064 -4.328124 6.154309
C -2.499619 -2.989192 6.213339
C -1.624104 -1.991647 5.763961
H 0.924398 -3.969416 4.732070
H -0.590938 -5.720886 5.551759
H -2.795284 -5.101592 6.497199
H -3.483774 -2.726468 6.591528
H -1.995382 -0.999754 5.759749
C 3.211021 -2.218559 3.592252
C 4.398391 -2.956280 3.643796
C 4.853991 -3.472084 4.857515
C 4.113862 -3.256193 6.019874
C 2.929325 -2.513467 5.974888
H 2.856238 -1.838462 2.644581
H 4.962559 -3.128415 2.737492
H 5.777030 -4.041717 4.897068
H 4.460564 -3.653250 6.964457
H 2.361572 -2.353207 6.882438
C 5.015712 0.702964 3.490971
C 6.029855 0.215685 4.227056
C 5.844755 -0.085326 5.579288
C 6.901358 -0.611739 6.340365
C 6.723083 -0.891725 7.642348
C 5.485552 -0.663788 8.260064
C 4.450601 -0.160289 7.564542
C 4.588356 0.151263 6.191558
C 3.517276 0.685470 5.421001
H 5.215533 0.920168 2.457378
H 6.997717 0.050758 3.763467
H 7.866934 -0.793968 5.883520
H 7.543970 -1.296962 8.225026
H 5.363716 -0.898575 9.312117
H 3.508476 -0.010647 8.075035
C -0.198819 1.074587 6.121192
C -0.235118 2.152457 6.920552
C 0.949747 2.733935 7.368492
C 0.909875 3.879221 8.180564
C 2.046824 4.507709 8.518560
C 3.283344 4.043910 8.052504
C 3.360999 2.951409 7.272055
C 2.194877 2.228987 6.916232
C 2.226130 1.064231 6.088235
H -1.107478 0.794620 5.641424
H -1.188168 2.615847 7.156261
H -0.038618 4.270140 8.529418
H 2.009613 5.392668 9.145317
H 4.187852 4.578983 8.321283
H 4.336925 2.651702 6.921663

TS$_{4-5}$

Rh -1.614564 -0.188381 -0.230143
P -0.193242 1.701702 -0.554311
P 0.088627 -1.680951 0.412883
C -1.155642 2.840573 -1.580980
C 0.412516 2.582051 0.951204
C -0.635667 -2.874336 1.568237
C 0.871109 -2.586269 -0.994505
C 1.275748 1.163511 -1.496749
C 1.477347 -0.833855 1.265628
N -3.363205 -1.456832 -0.440969
C -4.223633 -0.241965 -0.766084
C -4.509636 0.616668 0.434117
C -5.703095 1.104070 0.828061
C -5.797234 2.007225 2.038433
C -7.021935 0.836990 0.138726
H -5.134620 -0.576196 -1.276014
H -3.643444 0.322657 -1.526567
H -3.623834 0.894016 1.014380
H -7.752169 0.445086 0.857227
H -7.439420 1.772551 -0.255397
H -6.952830 0.130415 -0.691634
H -6.206779 2.986915 1.760560
H -6.475939 1.582266 2.788536
H -4.821522 2.166241 2.507023
C -3.936754 -2.243953 0.693075
H -4.965740 -2.548754 0.466673
H -3.333219 -3.141368 0.839386
H -3.927565 -1.645376 1.601578
C -3.231926 -2.333214 -1.650030
H -4.200320 -2.780175 -1.909994
H -2.864076 -1.740813 -2.488412
H -2.518626 -3.129158 -1.429031
C -1.832716 2.322594 -2.686026
C -2.632323 3.153399 -3.480433
C -2.764759 4.505549 -3.149417
C -2.109600 5.023907 -2.027600
C -1.311566 4.184162 -1.239901
H -1.724801 1.276590 -2.929402
H -3.151392 2.753861 -4.347073
H -3.385380 5.153212 -3.758846
H -2.229631 6.071258 -1.764733
H -0.834621 4.577736 -0.359630
C -0.024568 2.175969 2.220477
C 0.477353 2.790092 3.372735
C 1.420630 3.813225 3.270827
C 1.861104 4.222852 2.012072
C 1.366937 3.608078 0.857122
H -0.743234 1.371410 2.310948
H 0.137075 2.463340 4.345860
H 1.813779 4.284509 4.166075
H 2.601429 5.006804 1.926858
H 1.733709 3.916861 -0.113407
C -1.307059 -2.387822 2.691619
C -1.909244 -3.270789 3.596179
C -1.853566 -4.647325 3.357436
C -1.209262 -5.139396 2.217724
C -0.607797 -4.247585 1.320268
H -1.359948 -1.321693 2.853013
H -2.426102 -2.892121 4.473321
H -2.323016 -5.335615 4.051542
H -1.186799 -6.208587 2.026298
H -0.146948 -4.626728 0.425512
C 0.452657 -2.330876 -2.308361
C 1.079971 -2.961296 -3.387892
C 2.133705 -3.849123 -3.168659
C 2.561930 -4.103414 -1.865466
C 1.944261 -3.467921 -0.783472
H -0.345623 -1.624146 -2.491512
H 0.751962 -2.750411 -4.396596
H 2.623123 -4.333293 -4.007835
H 3.386776 -4.780697 -1.689790
H 2.301714 -3.652603 0.221493
C 1.395952 1.506166 -2.857136
C 2.365014 0.994161 -3.636630
C 3.300655 0.098374 -3.107464
C 4.301807 -0.449118 -3.926161
C 5.215152 -1.287553 -3.408200
C 5.186698 -1.624638 -2.047757
C 4.243514 -1.120827 -1.232170
C 3.255332 -0.241851 -1.731058
C 2.239421 0.308617 -0.902521
H 0.699138 2.192874 -3.304650
H 2.416945 1.276459 -4.683914
H 4.347301 -0.195815 -4.978795
H 5.987422 -1.705571 -4.045586
H 5.935346 -2.301624 -1.650882
H 4.250428 -1.414235 -0.191067
C 1.721167 -1.104864 2.627623
C 2.656448 -0.445556 3.332704
C 3.442273 0.535817 2.722673
C 4.404290 1.237794 3.467198
C 5.173911 2.165213 2.873774
C 5.032484 2.443800 1.507526
C 4.122930 1.792094 0.761501
C 3.284682 0.810271 1.340537
C 2.304988 0.103604 0.587390
H 1.158180 -1.863123 3.139638
H 2.800627 -0.679539 4.383216
H 4.534277 1.034172 4.523555
H 5.916457 2.702844 3.454295
H 5.665654 3.195722 1.049042
H 4.040214 2.047050 -0.286157

TS$_{4-45}$

Rh 0.250838 -3.684156 -0.409692
P 1.880261 -2.030527 -0.078311
P -1.629753 -2.300872 0.179884
C 3.430530 -2.873219 0.328173
C 2.140059 -0.889921 -1.507240
C -3.042877 -3.085403 -0.630341
C -1.952377 -2.151294 1.988441
C 1.431471 -0.964177 1.347835
C -1.413298 -0.619264 -0.494043
N -0.361097 -6.192062 1.148918
C -0.141083 -6.273947 -0.310942
C 1.172719 -5.647643 -0.736411
C 1.388637 -5.065503 -1.990714
C 2.799994 -4.747821 -2.443013
C 0.389178 -5.120366 -3.133484
H -0.237944 -7.305627 -0.714777
H -0.986247 -5.721757 -0.780552
H 2.037078 -5.846980 -0.113042
H 0.431430 -4.208515 -3.735373
H 0.650291 -5.959101 -3.795244
H -0.643643 -5.271609 -2.811696
H 3.098447 -5.456190 -3.225841
H 2.880011 -3.745414 -2.875252
H 3.523550 -4.843292 -1.628106
C 0.566999 -7.016303 1.947450
H 1.601349 -6.725758 1.751549
H 0.368134 -6.846480 3.007594
H 0.458965 -8.095252 1.736392
C -1.771117 -6.430017 1.515682
H -2.415917 -5.736728 0.962435
H -2.106918 -7.458792 1.296717
H -1.902757 -6.242011 2.583146
C 3.396279 -3.892322 1.281052
C 4.558311 -4.603997 1.601348
C 5.756855 -4.296612 0.950261
C 5.793724 -3.289910 -0.020030
C 4.624964 -2.583918 -0.333219
H 2.461711 -4.124770 1.771024
H 4.532060 -5.393068 2.347571
H 6.659797 -4.846415 1.192052
H 6.723994 -3.066475 -0.534920
H 4.649350 -1.837388 -1.106187
C 1.406067 -1.074074 -2.687836
C 1.545043 -0.182518 -3.756380
C 2.412859 0.905375 -3.656379
C 3.140703 1.101556 -2.482585
C 2.998317 0.217692 -1.408063
H 0.713265 -1.901157 -2.766976
H 0.969245 -0.333248 -4.659336
H 2.515091 1.600234 -4.483974
H 3.803382 1.951734 -2.393019
H 3.546223 0.396123 -0.491652
C -2.916304 -3.446428 -1.971869
C -3.956188 -4.118012 -2.625984
C -5.118991 -4.443038 -1.920300
C -5.240449 -4.105676 -0.568184
C -4.194170 -3.432953 0.076047
H -2.012573 -3.191074 -2.503764
H -3.863573 -4.389135 -3.673959
H -5.927125 -4.965180 -2.420759
H -6.139555 -4.373962 -0.020290
H -4.275415 -3.202169 1.123954
C -1.162718 -2.870830 2.897411
C -1.357304 -2.723942 4.274690
C -2.338383 -1.857603 4.758400
C -3.127977 -1.138473 3.860477
C -2.934283 -1.277719 2.482306
H -0.388762 -3.534356 2.532563
H -0.738544 -3.281042 4.964747
H -2.483962 -1.740483 5.827577

H -3.882654 -0.457180 4.229372
H -3.537380 -0.699092 1.794399
C 2.166890 -1.072216 2.545846
C 1.819259 -0.408642 3.661853
C 0.703153 0.432302 3.667383
C 0.329170 1.106276 4.841486
C -0.733468 1.928090 4.840098
C -1.481158 2.127781 3.671644
C -1.153830 1.500491 2.528010
C -0.046395 0.621318 2.478737
C 0.328433 -0.067100 1.290568
H 3.036163 -1.701840 2.594468
H 2.408570 -0.532745 4.565399
H 0.895960 0.969863 5.754926
H -1.016374 2.446713 5.750250
H -2.334981 2.796319 3.693510
H -1.765621 1.674523 1.653473
C -2.201226 -0.199444 -1.582532
C -1.985601 0.972329 -2.206327
C -0.962820 1.825520 -1.777335
C -0.731377 3.043957 -2.436233
C 0.233058 3.873732 -2.004099
C 1.018102 3.540431 -0.891517
C 0.827548 2.382122 -0.235269
C -0.168864 1.471792 -0.656061
C -0.398825 0.235580 0.007949
H -3.002104 -0.822629 -1.940541
H -2.610243 1.255310 -3.048225
H -1.331825 3.325262 -3.293307
H 0.404864 4.815028 -2.515832
H 1.788923 4.226993 -0.558552
H 1.461661 2.158338 0.611979

TS_{5-36}

Rh -1.698576 0.406110 -0.076552
P -0.498651 -1.630215 0.187063
P 0.294015 1.834482 -0.371027
C -1.626599 -2.776812 1.017770
C 0.141016 -2.405617 -1.362027
C -0.211354 3.220993 -1.420917
C 1.049302 2.482037 1.183251
C 0.946905 -1.337496 1.272263
C 1.628523 0.916989 -1.225673
N -3.339812 1.806728 0.118314
C -3.859602 0.477993 0.375359
C -5.033425 -0.020013 -0.374246
C -6.004670 -0.817600 0.128782
C -7.140240 -1.274293 -0.758263
C -6.070460 -1.331105 1.547301
H -3.872969 0.257711 1.437533
H -2.882044 -0.622222 -0.364140
H -5.081922 0.246816 -1.424710
H -7.046760 -1.105282 1.991121
H -5.965921 -2.424500 1.553142
H -5.297894 -0.924355 2.204077
H -7.171087 -2.370720 -0.809653
H -8.106281 -0.951220 -0.350217
H -7.048904 -0.887713 -1.776676
C -3.826212 2.534617 -1.086382
H -4.884474 2.796203 -0.980987
H -3.237994 3.448614 -1.186109
H -3.676275 1.925719 -1.976805
C -3.233885 2.702921 1.309259
H -4.209398 3.148218 1.537525
H -2.877112 2.129128 2.163651
H -2.517058 3.495038 1.082807
C -2.327881 -2.320495 2.135090
C -3.251279 -3.151467 2.780879
C -3.491529 -4.435049 2.281856
C -2.817821 -4.885018 1.141941
C -1.891227 -4.047821 0.506984
H -2.142016 -1.323937 2.505810
H -3.787320 -2.801895 3.658825
H -4.210649 -5.080653 2.773950
H -3.021900 -5.876479 0.747053
H -1.400662 -4.385067 -0.388814
C -0.134634 -1.814889 -2.604028
C 0.395931 -2.356035 -3.779702
C 1.207077 -3.490168 -3.728921
C 1.487284 -4.083430 -2.497824
C 0.965997 -3.541679 -1.318534
H -0.748876 -0.924827 -2.652927
H 0.181270 -1.887211 -4.730389
H 1.622822 -3.905435 -4.641563
H 2.126160 -4.954985 -2.450601
H 1.210675 -3.995059 -0.366476
C -0.839767 2.940332 -2.635387
C -1.284081 3.980137 -3.461259
C -1.116178 5.306366 -3.051421
C -0.516626 5.593031 -1.820736
C -0.072494 4.545306 -1.003801
H -0.981057 1.911623 -2.930192
H -1.765831 3.761926 -4.410045
H -1.463622 6.115774 -3.683941
H -0.407245 6.624504 -1.497565
H 0.354289 4.767072 -0.041483
C 0.484877 2.151089 2.423497
C 1.081623 2.584416 3.612046
C 2.247281 3.350469 3.575537
C 2.817284 3.681699 2.345889
C 2.229687 3.242715 1.154999
H -0.407176 1.539780 2.463366
H 0.640196 2.316854 4.562348
H 2.711619 3.682027 4.498905
H 3.726652 4.266294 2.311332
H 2.693215 3.485111 0.207264
C 0.927096 -1.820982 2.595161
C 1.884293 -1.504917 3.484831
C 2.952323 -0.681877 3.114087
C 3.942531 -0.339880 4.049497
C 4.982931 0.426980 3.682776
C 5.099107 0.892316 2.365729
C 4.170935 0.586254 1.441949
C 3.052365 -0.209839 1.780432
C 2.047190 -0.552107 0.833531
H 0.129897 -2.462469 2.924987
H 1.824180 -1.891023 4.498061
H 3.876811 -0.695518 5.071011
H 5.745698 0.686096 4.409715
H 5.948624 1.508244 2.091403
H 4.293354 0.974880 0.440059
C 2.018567 1.305818 -2.522671
C 2.915076 0.605550 -3.238902
C 3.512053 -0.538968 -2.702065
C 4.440041 -1.275101 -3.456669
C 5.035331 -2.357018 -2.927624
C 4.744556 -2.764764 -1.618538
C 3.860836 -2.086335 -0.865284
C 3.201056 -0.945508 -1.379393
C 2.255233 -0.204253 -0.616887
H 1.603757 2.190698 -2.970568
H 3.178492 0.935784 -4.239247
H 4.685836 -0.972745 -4.467828
H 5.753516 -2.919715 -3.515017
H 5.239751 -3.639423 -1.210816
H 3.658425 -2.441905 0.135831

TS_{8-22}

Rh -3.510914 2.039327 1.471439
P -4.602207 3.311645 3.275930
P -4.437495 3.342737 -0.306001
C -4.888310 2.171414 4.655080
C -3.613162 4.751272 3.870903
C -3.471202 3.023438 -1.806135
C -6.219769 3.009780 -0.660866
C -6.216066 4.007898 2.757792
C -4.311366 5.120477 0.122156
N -2.266611 0.623449 2.519945
C -1.770323 0.701549 1.154717
C -0.532548 1.437171 0.860333
C 0.376055 1.102658 -0.089254
C 1.610376 1.953278 -0.282382
C 0.279805 -0.087470 -1.012478
H -1.847510 -0.264278 0.660430
H -3.030128 1.076696 0.260204
H -0.369062 2.348249 1.424529
H 0.221092 0.250813 -2.055369
H 1.180165 -0.708376 -0.933994
H -0.587178 -0.726116 -0.828367
H 2.514773 1.370033 -0.064092
H 1.700047 2.287090 -1.323238
H 1.607548 2.835551 0.363053
C -1.405116 1.114489 3.634481
H -0.561385 0.434695 3.796422
H -1.037721 2.114082 3.421118
H -2.019988 1.147735 4.536276
C -2.914315 -0.681053 2.856157
H -3.636874 -0.934077 2.080278
H -2.158093 -1.469615 2.946354
H -3.435938 -0.580086 3.807681
C -5.591602 0.991673 4.407069
C -5.812598 0.067371 5.435521
C -5.302111 0.320736 6.712233
C -4.569160 1.485890 6.959398
C -4.358629 2.406450 5.924776
H -5.963433 0.799781 3.412117
H -6.367778 -0.846489 5.244339
H -5.464405 -0.394312 7.511179
H -4.157477 1.668590 7.948081
H -3.763571 3.283378 6.108860
C -2.367265 5.026988 3.289116
C -1.632460 6.148945 3.686222
C -2.133083 7.007249 4.665840
C -3.373182 6.742046 5.247710
C -4.116380 5.627034 4.846871
H -1.978687 4.378166 2.514604
H -0.675902 6.354023 3.225287
H -1.563579 7.879906 4.969605
H -3.771355 7.409527 5.999895
H -5.088189 5.443926 5.287172
C -2.080599 3.110117 -1.736395
C -1.293665 2.831415 -2.860255
C -1.906910 2.432326 -4.050932
C -3.297712 2.307243 -4.118124
C -4.076050 2.595758 -2.989671
H -1.616321 3.410059 -0.809653
H -0.212894 2.919342 -2.810577
H -1.301877 2.210069 -4.923026
H -3.769011 1.979086 -5.040476
H -5.142333 2.465650 -3.038520
C -6.894546 1.998086 0.038103
C -8.257897 1.773707 -0.179994
C -8.962083 2.555722 -1.096166
C -8.298960 3.563984 -1.795861
C -6.937560 3.797242 -1.576444
H -6.361235 1.396048 0.762528
H -8.768120 0.995193 0.370644
H -10.021400 2.384577 -1.259747
H -8.842153 4.182409 -2.497682
H -6.438419 4.597553 -2.107664
C -7.398119 3.546274 3.371091
C -8.616226 3.946205 2.967708
C -8.749968 4.862433 1.920838
C -10.024486 5.268941 1.493184
C -10.150591 6.170845 0.505627
C -9.017584 6.721188 -0.108941
C -7.780622 6.356504 0.272504
C -7.594449 5.404871 1.302950
C -6.302238 4.987477 1.730923
H -7.348224 2.857445 4.194507
H -9.500384 3.555963 3.462743
H -10.913859 4.861891 1.959668
H -11.138210 6.482708 0.181847
H -9.143273 7.450034 -0.902386
H -6.934897 6.798291 -0.236553
C -3.353341 5.921168 -0.529901
C -3.089801 7.180489 -0.139936
C -3.779150 7.747814 0.935957
C -3.490635 9.057835 1.352041
C -4.178390 9.612638 2.364027
C -5.193839 8.898426 3.014636
C -5.498501 7.641849 2.644888
C -4.796342 7.009647 1.592712
C -5.087328 5.681290 1.173515
H -2.802575 5.532671 -1.366395
H -2.333973 7.757717 -0.664183
H -2.715929 9.631358 0.856860
H -3.952147 10.625932 2.679371
H -5.738129 9.367148 3.827240
H -6.281763 7.122721 3.180434

TS_{23-49}

Rh 1.660295 1.192467 -0.036078
P -0.627346 1.887834 -0.040585
P 1.043606 -1.311369 -0.534211
C -0.968882 3.562682 0.570472
C -1.567187 1.796358 -1.627985
C 2.258545 -1.946712 -1.723163
C 0.951431 -2.468622 0.903669
C -1.335120 0.701254 1.159541
C -0.613190 -1.461381 -1.322731
N 4.358632 1.152142 -0.602887
C 3.760160 1.720720 0.492680
C 3.519068 1.020830 1.730393
C 2.949458 1.600520 2.844410
C 2.758307 0.788381 4.101303
C 2.594888 3.059184 2.976426
H 1.784832 2.741974 0.023448
H 3.805155 2.800593 0.505254
H 3.801126 -0.023400 1.793767
H 1.611123 3.170153 3.447045
H 3.315856 3.558807 3.637844
H 2.560553 3.591789 2.025646
H 3.252375 1.281681 4.948303
H 1.692612 0.726602 4.359096
H 3.157763 -0.224335 4.012352
C 4.797349 2.020420 -1.717015
H 5.875271 2.212672 -1.654603
H 4.577068 1.524783 -2.665034
H 4.251317 2.962812 -1.679762
C 4.962622 -0.193564 -0.581224
H 5.038963 -0.562272 -1.604999
H 5.966397 -0.159705 -0.141834
H 4.343682 -0.888961 -0.015995
C -0.207924 4.630472 0.092893
C -0.473625 5.938257 0.518130
C -1.532534 6.177615 1.398536
C -2.341433 5.122095 1.828802
C -2.067710 3.818509 1.396511
H 0.557008 4.456851 -0.644916
H 0.122727 6.768357 0.149527
H -1.746595 7.189879 1.723686
H -3.188755 5.319032 2.479805
H -2.728476 3.016623 1.682125
C -0.901268 1.484465 -2.821841
C -1.603299 1.416822 -4.029800
C -2.974434 1.672642 -4.061509
C -3.641360 2.004309 -2.882128
C -2.944007 2.070950 -1.671435
H 0.161956 1.289914 -2.807932
H -1.079212 1.164008 -4.941333
H -3.519293 1.615595 -4.998509
H -4.704879 2.200890 -2.899068
H -3.475851 2.318396 -0.762457
C 2.492270 -1.215551 -2.888818
C 3.430855 -1.658213 -3.828858
C 4.152432 -2.830505 -3.583992
C 3.945484 -3.551458 -2.403597
C 3.001005 -3.102039 -1.471744
H 1.943575 -0.303563 -3.056469
H 3.608125 -1.091414 -4.738220
H 4.885936 -3.174415 -4.304765
H 4.524668 -4.449445 -2.207600
H 2.872475 -3.637831 -0.548160
C 1.221863 -1.997083 2.193518
C 1.111458 -2.845485 3.299698
C 0.718155 -4.173190 3.129852
C 0.429152 -4.648636 1.850128
C 0.533234 -3.800438 0.742665
H 1.488216 -0.961844 2.334204
H 1.319072 -2.464191 4.290098
H 0.626960 -4.829532 3.989440
H 0.111000 -5.673256 1.712838
H 0.287989 -4.174287 -0.243144
C -1.164512 1.004951 2.523932
C -1.526984 0.154375 3.498723
C -2.041562 -1.106098 3.179454
C -2.412448 -1.998288 4.198583
C -2.867036 -3.224320 3.891133
C -2.971755 -3.633715 2.554888
C -2.625045 -2.808541 1.551169
C -2.152424 -1.503068 1.821162
C -1.767364 -0.600534 0.789195
H -0.710431 1.944629 2.802300
H -1.394241 0.439488 4.537958
H -2.330756 -1.706028 5.238807
H -3.151827 -3.909251 4.683016
H -3.332683 -4.631573 2.330470
H -2.709147 -3.172274 0.536671
C -0.719503 -2.014412 -2.615404
C -1.898783 -2.121748 -3.252552
C -3.079498 -1.695418 -2.636518
C -4.308480 -1.800438 -3.308082
C -5.443714 -1.407666 -2.706172
C -5.420932 -0.891549 -1.402937
C -4.263713 -0.775340 -0.727598
C -3.041773 -1.170246 -1.319901
C -1.796939 -1.056387 -0.642318
H 0.155816 -2.378672 -3.123052
H -1.931962 -2.547230 -4.250929
H -4.349698 -2.201443 -4.313941
H -6.390200 -1.493650 -3.229929
H -6.350201 -0.583109 -0.936068
H -4.288615 -0.364413 0.272613

TS_{33-114}

Rh 1.885008 -0.005747 -0.656771
P 0.241677 -1.760051 -0.367689
P 0.219830 1.748878 -0.612224
C 0.646125 -3.137277 -1.476836
C 0.144625 -2.380612 1.368970
C 0.982805 3.383286 -0.603543
C -1.077302 1.832002 -1.922604
C -1.499647 -1.265444 -0.726483
C -0.548965 1.459057 1.011931
N 3.694272 1.303073 1.516387
C 4.502986 0.524021 0.545878
C 3.844157 -0.774870 0.115849
C 3.830679 -1.238800 -1.205938
C 3.881134 -2.728996 -1.462283
C 4.265672 -0.387152 -2.386819
H 5.510215 0.284449 0.946732
H 4.668762 1.170439 -0.320937
H 3.711116 -1.522383 0.893678
H 3.812814 -0.764028 -3.307955
H 3.991554 0.670494 -2.286789
H 5.357605 -0.424632 -2.512203
H 3.397476 -3.013654 -2.399741
H 4.934036 -3.028773 -1.552016
H 3.449359 -3.308947 -0.641563
C 3.712701 0.725212 2.881232
H 4.668535 0.938866 3.390487
H 2.898839 1.152488 3.471907
H 3.592873 -0.357306 2.846537
C 4.096442 2.726730 1.573131
H 4.127733 3.148562 0.566883
H 3.365128 3.288783 2.159460
H 5.089682 2.863793 2.033387
C 0.736296 -2.884037 -2.847128
C 1.030007 -3.919315 -3.743193
C 1.257926 -5.209647 -3.255595
C 1.198225 -5.464497 -1.881823
C 0.894961 -4.423686 -0.994629
H 0.568032 -1.883596 -3.209983
H 1.089154 -3.723759 -4.810123
H 1.492681 -6.014112 -3.943898
H 1.394888 -6.465000 -1.506671
H 0.885647 -4.616806 0.063185
C 0.908035 -1.762857 2.365126
C 0.806010 -2.171472 3.698462
C -0.074776 -3.193399 4.053723
C -0.861353 -3.798033 3.072205
C -0.765987 -3.385110 1.739013
H 1.546232 -0.935931 2.100280
H 1.397249 -1.676960 4.456972
H -0.159939 -3.504694 5.089999
H -1.560394 -4.577559 3.343663
H -1.400554 -3.841919 0.990392
C 2.188014 3.564117 -1.275284
C 2.776414 4.833901 -1.340896
C 2.140430 5.922380 -0.734143
C 0.916063 5.748521 -0.079643
C 0.333401 4.475928 -0.027745
H 2.653907 2.719908 -1.755986
H 3.716746 4.977785 -1.865167
H 2.590890 6.907488 -0.783807
H 0.417506 6.600015 0.374772
H -0.618538 4.335705 0.458567
C -0.965313 1.033148 -3.068918
C -1.949699 1.072579 -4.062102
C -3.050913 1.918285 -3.926937
C -3.161405 2.731068 -2.798859
C -2.179192 2.694739 -1.803515
H -0.112460 0.379797 -3.184838
H -1.854260 0.444668 -4.937194
H -3.816620 1.945388 -4.695679

H -4.015189 3.385221 -2.684728
H -2.290035 3.315731 -0.925798
C -2.216248 -1.951596 -1.730067
C -3.478347 -1.628098 -2.060068
C -4.141453 -0.592995 -1.396383
C -5.452353 -0.243243 -1.759545
C -6.092226 0.745239 -1.112960
C -5.465833 1.439146 -0.068704
C -4.212188 1.131869 0.308806
C -3.495449 0.099741 -0.341716
C -2.163803 -0.248461 0.020074
H -1.765715 -2.772836 -2.255710
H -3.984574 -2.178084 -2.847547
H -5.956256 -0.768291 -2.562425
H -7.105283 1.009887 -1.397897
H -6.001672 2.233754 0.439392
H -3.763731 1.699883 1.112365
C 0.045793 2.055765 2.138681
C -0.334731 1.751112 3.391679
C -1.317955 0.780803 3.615096
C -1.702746 0.448967 4.924262
C -2.628210 -0.501695 5.136341
C -3.219171 -1.177741 4.059822
C -2.876318 -0.888897 2.791901
C -1.912790 0.107846 2.515965
C -1.516776 0.438372 1.190572
H 0.858936 2.751764 2.002786
H 0.148131 2.239422 4.232468
H -1.255365 0.955681 5.771178
H -2.921789 -0.753008 6.150217
H -3.959221 -1.946160 4.254969
H -3.346412 -1.443938 1.991693

TS$_{36-93}$

Rh -0.684426 1.361697 2.585679
P -0.283310 -0.543748 4.036760
P -0.148003 3.285867 4.098538
C -1.656588 -1.710058 3.816029
C 1.304701 -1.494265 3.929461
C 0.025214 4.797063 3.117003
C -1.222571 3.588886 5.567638
C -0.339463 0.009513 5.791535
C 1.496865 2.812645 4.732169
N -2.913162 0.726401 1.095755
C -1.539712 0.553227 0.817368
C -0.813259 1.645353 0.162586
C 0.434387 1.549361 -0.408425
C 1.034552 2.732695 -1.120373
C 1.241191 0.280733 -0.470425
H -1.282148 -0.472297 0.575496
H 0.834431 1.388926 2.481424
H -1.323795 2.595047 0.042939
H 1.325656 -0.050058 -1.514450
H 2.263052 0.461900 -0.118537
H 0.810933 -0.531071 0.116735
H 2.026830 2.968282 -0.715688
H 1.179706 2.503610 -2.184290
H 0.404634 3.622519 -1.042841
C -3.638321 1.996083 0.869444
H -3.964126 2.092605 -0.176291
H -4.521795 2.003868 1.510992
H -3.026883 2.855818 1.141475
C -3.785335 -0.459023 0.989458
H -4.200873 -0.548131 -0.023735
H -3.213317 -1.362184 1.212626
H -4.609811 -0.380769 1.700828
C -2.959279 -1.273296 4.059555
C -4.038170 -2.158090 3.937683
C -3.807892 -3.475304 3.529216
C -2.508958 -3.906659 3.241602
C -1.435181 -3.018273 3.383266
H -3.126258 -0.247514 4.349987
H -5.050712 -1.821189 4.140301
H -4.640952 -4.161016 3.420938
H -2.337441 -4.925497 2.905522
H -0.438503 -3.344684 3.138916
C 2.279785 -1.184414 2.968181
C 3.487259 -1.889045 2.925894
C 3.738371 -2.912806 3.838665
C 2.776567 -3.232773 4.796000
C 1.570041 -2.527971 4.845734
H 2.109928 -0.399380 2.251718
H 4.229813 -1.636590 2.180742
H 4.676712 -3.457593 3.803674
H 2.964519 -4.025936 5.507445
H 0.837186 -2.780605 5.601306
C 0.716565 4.731534 1.907220
C 0.843904 5.869117 1.100640
C 0.254189 7.070868 1.504571
C -0.464777 7.134987 2.702598
C -0.583175 5.991415 3.503093
H 1.161858 3.798233 1.604186
H 1.392214 5.821999 0.164003
H 0.344883 7.954022 0.881763
H -0.937355 8.066084 3.002949
H -1.164269 6.033686 4.407680
C -2.424976 2.881132 5.708902
C -3.219591 3.054101 6.847051
C -2.824848 3.936343 7.853377
C -1.632173 4.647541 7.718755
C -0.830820 4.473327 6.585801
H -2.734419 2.186858 4.938613
H -4.141820 2.498001 6.946110
H -3.440607 4.066562 8.737697
H -1.315587 5.325230 8.500059
H 0.103817 5.012984 6.502083
C -1.237711 -0.617820 6.680498
C -1.386520 -0.215682 7.953403
C -0.616453 0.833814 8.458845
C -0.778124 1.252908 9.789583
C -0.006241 2.232167 10.288654
C 0.973275 2.844171 9.495358
C 1.156702 2.471470 8.216284
C 0.359111 1.454455 7.638164
C 0.514530 1.038471 6.284029
H -1.820994 -1.462065 6.358638
H -2.101281 -0.721501 8.595801
H -1.521436 0.785054 10.424357
H -0.134114 2.548950 11.318598
H 1.590062 3.628054 9.921618
H 1.920200 2.976907 7.641371
C 2.637028 3.512543 4.295351
C 3.880460 3.044387 4.502924
C 4.078997 1.834973 5.178490
C 5.377387 1.340133 5.382454
C 5.566589 0.202991 6.072883
C 4.477224 -0.498665 6.607753
C 3.219534 -0.055525 6.434203
C 2.966682 1.121742 5.695239
C 1.651406 1.616588 5.482823
H 2.528807 4.446738 3.771070
H 4.734313 3.608692 4.139132
H 6.235086 1.875081 4.991584
H 6.572413 -0.173044 6.229204
H 4.653752 -1.409672 7.169129
H 2.406906 -0.625460 6.863333

TS$_{45-46}$

Rh 1.778119 0.322566 -0.290212
P 0.383120 -1.575177 -0.772763
P 0.062397 1.664428 0.622787
C 1.259644 -2.492767 -2.060745
C 0.040982 -2.687448 0.657570
C 0.846055 2.801059 1.793394
C -0.932547 2.615695 -0.607778
C -1.223065 -1.008335 -1.430817
C -1.131453 0.631136 1.556153
N 4.677732 -1.019681 0.783718
C 4.178881 -0.246031 -0.342631
C 3.781853 1.162723 0.001643
C 3.328369 2.073878 -0.955664
C 3.113396 3.524597 -0.582859
C 3.420195 1.827888 -2.450652
H 4.817366 -0.292921 -1.248625
H 3.243866 -0.828564 -0.728899
H 3.948609 1.489033 1.022324
H 4.329617 2.310635 -2.837499
H 2.571902 2.275997 -2.974553
H 3.467265 0.769706 -2.716196
H 2.148481 3.901021 -0.938720
H 3.887934 4.140807 -1.055826
H 3.178966 3.686330 0.497017
C 4.645941 -2.472393 0.546562
H 3.623573 -2.778234 0.293362
H 4.939798 -2.992714 1.459642
H 5.315350 -2.795191 -0.271806
C 5.977682 -0.545836 1.301191
H 5.919362 0.515304 1.554277
H 6.800249 -0.687805 0.577613
H 6.215939 -1.098507 2.212156
C 1.756032 -1.782454 -3.154299
C 2.508544 -2.433222 -4.139415
C 2.778899 -3.799210 -4.010993
C 2.307266 -4.509851 -2.902363
C 1.554545 -3.848993 -1.923131
H 1.543969 -0.727617 -3.239265
H 2.885110 -1.883937 -4.997847
H 3.363589 -4.308418 -4.769337
H 2.534256 -5.567396 -2.798819
H 1.221182 -4.389881 -1.054677
C 0.644104 -2.432628 1.898076
C 0.348697 -3.232932 3.006414
C -0.550990 -4.293049 2.889388
C -1.154346 -4.553623 1.658645
C -0.866906 -3.753146 0.548399
H 1.333477 -1.604134 2.002565
H 0.816980 -3.022952 3.958443
H -0.783377 -4.909430 3.752065
H -1.860921 -5.367109 1.564311
H -1.357161 -3.950088 -0.396398
C 1.773448 2.279374 2.696425
C 2.440337 3.121272 3.594267
C 2.182179 4.495271 3.573377
C 1.269508 5.026984 2.656581
C 0.607619 4.175790 1.762228
H 1.970425 1.217327 2.697201
H 3.158163 2.713848 4.300726
H 2.698429 5.152747 4.264217
H 1.085672 6.097525 2.632914
H -0.064437 4.593163 1.034643
C -0.621368 2.527385 -1.972160
C -1.402213 3.192603 -2.922795
C -2.504729 3.948427 -2.523015
C -2.827113 4.035101 -1.168515
C -2.054718 3.363931 -0.215086
H 0.216578 1.924138 -2.294512
H -1.154360 3.111851 -3.972413
H -3.113432 4.459519 -3.262264
H -3.689459 4.607068 -0.853543
H -2.331085 3.414954 0.830319
C -1.512879 -1.180704 -2.797783
C -2.614139 -0.655762 -3.363493
C -3.523873 0.078739 -2.595156
C -4.667496 0.636420 -3.189655
C -5.553852 1.316775 -2.443485
C -5.354850 1.477145 -1.065170
C -4.271379 0.958923 -0.460040
C -3.305365 0.242275 -1.203046
C -2.144381 -0.317170 -0.601531
H -0.844218 -1.744299 -3.424425
H -2.795825 -0.804031 -4.423772
H -4.845346 0.517526 -4.251973
H -6.436863 1.742883 -2.908430
H -6.085129 2.027677 -0.482062
H -4.150465 1.114501 0.603509
C -1.160403 0.713422 2.963056
C -1.932897 -0.093069 3.711060
C -2.753720 -1.048873 3.105959
C -3.545096 -1.903009 3.891265
C -4.347128 -2.808700 3.307253
C -4.409043 -2.913929 1.911061
C -3.666341 -2.113835 1.125697
C -2.802604 -1.147013 1.692131
C -1.995437 -0.286090 0.896228
H -0.551813 1.435021 3.477156
H -1.914283 -0.001646 4.792942
H -3.516260 -1.835505 4.972418
H -4.957256 -3.464452 3.919755
H -5.064414 -3.651909 1.461226
H -3.736638 -2.237487 0.053703

TS$_{46-47}$

Rh 1.854531 0.280871 -0.288986
P 0.110255 1.732019 0.570882
P 0.422081 -1.614756 -0.715888
C 0.899337 2.905244 1.702671
C -0.906491 2.644254 -0.673138
C 1.274414 -2.579796 -1.985948
C 0.035421 -2.695970 0.728878
C -1.077432 0.732354 1.546822
C -1.178892 -1.036253 -1.383871
N 4.962981 -0.844050 0.736761
C 4.344530 -0.143413 -0.270796
C 3.833589 1.220309 -0.071622
C 3.278991 1.964051 -1.129579
C 2.993477 3.436992 -0.920602
C 3.404500 1.575274 -2.592133
H 4.760656 -0.359796 -1.253335
H 3.083678 -0.843904 -0.622190
H 3.986514 1.705449 0.884641
H 4.266186 2.099830 -3.030709
H 2.519661 1.887232 -3.153182
H 3.541511 0.504523 -2.751911
H 2.017469 3.725863 -1.321630
H 3.745782 4.033336 -1.451952
H 3.038938 3.720009 0.135207
C 4.614406 -0.588624 2.141658
H 3.556056 -0.818350 2.333975
H 4.799561 0.454636 2.405481
H 5.239679 -1.214149 2.778299
C 5.429108 -2.211059 0.413706
H 4.600454 -2.927093 0.452929
H 6.201715 -2.504902 1.120886
H 5.854246 -2.215688 -0.592878
C 1.828202 2.409685 2.618847
C 2.507295 3.278331 3.481256
C 2.260346 4.652700 3.410906
C 1.347650 5.157966 2.479329
C 0.673941 4.280042 1.620385
H 2.017109 1.347569 2.656515
H 3.226591 2.891772 4.197865
H 2.786592 5.331078 4.073488
H 1.174415 6.227682 2.415410
H 0.006244 4.676721 0.877393
C -0.630630 2.498376 -2.040252
C -1.427587 3.134462 -2.997368
C -2.511932 3.917948 -2.601403
C -2.800585 4.060708 -1.244060
C -2.012277 3.418427 -0.283792
H 0.192337 1.873177 -2.358681
H -1.206335 3.009715 -4.048629
H -3.133030 4.406678 -3.345449
H -3.649033 4.654316 -0.931375
H -2.263202 3.512841 0.764995
C 1.751064 -1.910051 -3.113596
C 2.485436 -2.596169 -4.088320
C 2.759922 -3.956210 -3.914919
C 2.311812 -4.625237 -2.771310
C 1.577290 -3.929063 -1.802670
H 1.535401 -0.859501 -3.234586
H 2.845023 -2.078594 -4.973382
H 3.331033 -4.492614 -4.664853
H 2.544604 -5.677417 -2.631945
H 1.268130 -4.436810 -0.905878
C 0.579437 -2.401465 1.987298
C 0.248450 -3.178097 3.102355
C -0.630134 -4.254414 2.974297
C -1.178970 -4.552437 1.726724
C -0.857230 -3.773968 0.610241
H 1.243285 -1.555293 2.101264
H 0.670109 -2.936208 4.068478
H -0.890456 -4.852785 3.841696
H -1.870291 -5.377912 1.623512
H -1.307097 -3.999391 -0.348204
C -1.109543 0.872249 2.949092
C -1.876242 0.090067 3.728241
C -2.688566 -0.896153 3.161412
C -3.477035 -1.721612 3.979598
C -4.273628 -2.654225 3.431623
C -4.332996 -2.816033 2.040739
C -3.592501 -2.045161 1.224655
C -2.733443 -1.052979 1.752680
C -1.929539 -0.220891 0.923474
H -0.510088 1.621404 3.434007
H -1.861974 0.226985 4.805448
H -3.451154 -1.609587 5.057136
H -4.881880 -3.287380 4.069265
H -4.984385 -3.574520 1.620230
H -3.660763 -2.212000 0.158366
C -1.486219 -1.257999 -2.740192
C -2.586794 -0.742114 -3.315007
C -3.481780 0.029413 -2.566576
C -4.626525 0.574100 -3.170926
C -5.501260 1.287437 -2.442193
C -5.289064 1.495236 -1.072314
C -4.203885 0.991123 -0.458409
C -3.248929 0.241687 -1.183530
C -2.086518 -0.304897 -0.572007
H -0.834833 -1.857147 -3.351264
H -2.781437 -0.929894 -4.366759
H -4.815003 0.418077 -4.226600
H -6.385329 1.703132 -2.914507
H -6.010316 2.071535 -0.503040
H -4.073166 1.183817 0.597818

TS$_{47-48}$

Rh 1.852031 0.276628 -0.447491
P 0.037705 1.885320 0.503460
P 0.399881 -1.666916 -0.615170
C 0.872512 3.037263 1.623574
C -0.930911 2.820763 -0.754740
C 1.163599 -2.923053 -1.689628
C 0.013949 -2.445926 1.014534
C -1.186294 0.938386 1.480582
C -1.206830 -1.131937 -1.323207
N 4.636007 -1.503420 0.204796
C 4.138496 -0.531916 -0.616272
C 3.986426 0.839790 -0.234883
C 3.533311 1.868923 -1.084330
C 3.475040 3.285036 -0.549231
C 3.602342 1.809588 -2.601263
H 4.160647 -0.793420 -1.665343
H 2.027375 -0.096194 -1.907057
H 4.170149 1.112682 0.800373
H 4.457793 2.410544 -2.940413
H 2.703898 2.244273 -3.049495
H 3.723776 0.800226 -2.998018
H 2.570085 3.802826 -0.884826
H 4.329852 3.861923 -0.924882
H 3.507443 3.320364 0.543800
C 4.824104 -1.298646 1.646668
H 5.351425 -0.361997 1.840621
H 5.433381 -2.114453 2.037783
H 3.865933 -1.290068 2.184210
C 4.689433 -2.892677 -0.268012
H 4.550262 -2.919680 -1.351440
H 3.895689 -3.496717 0.193094
H 5.655899 -3.341005 -0.025397
C 1.730324 2.508955 2.589669
C 2.435281 3.357681 3.451542
C 2.289821 4.742877 3.327116
C 1.452463 5.278261 2.342845
C 0.750098 4.420336 1.486661
H 1.844884 1.438100 2.664813
H 3.097938 2.947069 4.208182
H 2.837419 5.405386 3.988414
H 1.358816 6.355574 2.237627
H 0.137704 4.835140 0.706030
C -0.649811 2.651084 -2.117995
C -1.411172 3.313025 -3.086721
C -2.460785 4.149838 -2.705846
C -2.749751 4.321875 -1.351766
C -1.998070 3.653759 -0.379826
H 0.152275 1.992385 -2.424528
H -1.187802 3.170105 -4.135281
H -3.052744 4.660325 -3.459038
H -3.568255 4.961771 -1.050627
H -2.246325 3.775499 0.666818
C 1.564473 -2.579112 -2.985708
C 2.219168 -3.511148 -3.800318
C 2.490940 -4.789686 -3.307570
C 2.115333 -5.136232 -2.007440
C 1.459321 -4.198194 -1.200774
H 1.343509 -1.600541 -3.377120
H 2.517094 -3.246361 -4.811086
H 3.001951 -5.513535 -3.932954
H 2.346232 -6.125298 -1.621505
H 1.218168 -4.459626 -0.185947
C 0.619164 -1.956779 2.181159
C 0.306102 -2.509340 3.427324
C -0.611687 -3.556006 3.522307
C -1.216700 -4.050266 2.366469
C -0.912401 -3.496673 1.118657
H 1.321713 -1.136103 2.119311
H 0.775351 -2.118844 4.319973
H -0.857071 -3.980843 4.490521

H -1.937217 -4.854126 2.434639
H -1.401167 -3.875305 0.230115
C -1.299139 1.179666 2.864372
C -2.109360 0.452637 3.652195
C -2.892293 -0.570625 3.110487
C -3.736909 -1.329277 3.937275
C -4.513808 -2.290246 3.410643
C -4.497469 -2.547854 2.033182
C -3.700429 -1.844417 1.209476
C -2.855587 -0.828328 1.716018
C -1.991727 -0.066664 0.878063
H -0.731742 1.969089 3.325017
H -2.158177 0.667506 4.715507
H -3.772202 -1.140751 5.003825
H -5.166674 -2.870325 4.054677
H -5.136682 -3.325663 1.629435
H -3.713161 -2.084120 0.155069
C -1.539030 -1.514086 -2.637347
C -2.597543 -0.999500 -3.286540
C -3.430793 -0.069905 -2.658049
C -4.530806 0.472788 -3.342219
C -5.360886 1.325505 -2.718890
C -5.148505 1.679052 -1.379493
C -4.105471 1.180977 -0.692147
C -3.192338 0.294394 -1.308662
C -2.075942 -0.252420 -0.615260
H -0.945393 -2.243717 -3.154733
H -2.808139 -1.314216 -4.304534
H -4.722405 0.201395 -4.373755
H -6.210867 1.738908 -3.251892
H -5.835347 2.363696 -0.893925
H -3.974721 1.485472 0.337382

TS$_{48-53}$

Rh -1.089394 0.639227 -1.982600
P -0.561722 0.962081 -4.293469
P -3.487104 1.410855 -2.188557
C 1.243266 1.030472 -4.450128
C -1.205893 -0.332858 -5.444680
C -4.439785 0.454570 -0.980433
C -3.821034 3.213139 -1.961451
C -1.272381 2.556611 -4.867194
C -4.148968 0.977859 -3.841461
N -2.452717 0.793665 1.934438
C -1.608406 0.223650 1.066598
C -0.686684 0.898524 0.248028
C 0.408553 0.222861 -0.436753
C 1.709842 1.005892 -0.510301
C 0.643397 -1.269285 -0.179992
H -1.634714 -0.860351 1.051168
H -0.050728 -0.457353 -2.363064
H -0.594183 1.973250 0.366472
H 1.061923 -1.419403 0.824901
H 1.366541 -1.658946 -0.901454
H -0.266053 -1.868340 -0.278010
H 2.361971 0.603261 -1.291016
H 2.248301 0.912455 0.443744
H 1.541513 2.068298 -0.701697
C -3.266221 -0.022328 2.858465
H -3.239476 -1.066305 2.542895
H -4.302027 0.323072 2.844549
H -2.878176 0.056654 3.878459
C -2.533062 2.259314 2.080024
H -2.734911 2.718667 1.107484
H -1.599006 2.665978 2.479813
H -3.348670 2.502491 2.759413
C 1.951753 1.941010 -3.666618
C 3.350140 1.989924 -3.720334
C 4.040094 1.098164 -4.546251
C 3.338853 0.158883 -5.308379
C 1.939632 0.122127 -5.249465
H 1.409945 2.621104 -3.027567
H 3.899577 2.710127 -3.120153
H 5.123469 1.128385 -4.589278
H 3.880622 -0.543911 -5.935632
H 1.408187 -0.625610 -5.811819
C -1.913319 -1.433778 -4.938937
C -2.448073 -2.392021 -5.805996
C -2.284921 -2.262063 -7.185575
C -1.583364 -1.170428 -7.697398
C -1.052623 -0.205524 -6.835539
H -2.060552 -1.535973 -3.871639
H -2.998768 -3.231744 -5.404060
H -2.706839 -3.002762 -7.857654
H -1.464672 -1.057264 -8.766690
H -0.529011 0.648923 -7.244943
C -4.213345 -0.921116 -0.905428
C -4.965694 -1.716393 -0.032445
C -5.946185 -1.123904 0.769368
C -6.162371 0.256968 0.713602
C -5.398492 1.045063 -0.156608
H -3.462127 -1.366614 -1.537641
H -4.792508 -2.786869 0.024725
H -6.529581 -1.733936 1.449680
H -6.907968 0.716075 1.356212
H -5.542727 2.109556 -0.170871
C -2.768820 4.075637 -1.622251
C -2.991088 5.451064 -1.498461
C -4.264018 5.980234 -1.713641
C -5.315780 5.130193 -2.056605
C -5.096228 3.755408 -2.190926
H -1.774943 3.676648 -1.464579
H -2.170682 6.105442 -1.237128
H -4.434306 7.048025 -1.618983
H -6.302615 5.535490 -2.234467
H -5.913956 3.108633 -2.482138
C -0.417121 3.644390 -5.134928
C -0.886501 4.878623 -5.387726
C -2.262748 5.120693 -5.418502
C -2.750777 6.413113 -5.671550
C -4.073876 6.632584 -5.750171
C -4.984012 5.578825 -5.589819
C -4.554378 4.328125 -5.345418
C -3.172468 4.048893 -5.236463
C -2.678066 2.740071 -4.976949
H 0.647265 3.507736 -5.150628
H -0.189769 5.690742 -5.573798
H -2.064401 7.239910 -5.811756
H -4.444734 7.632965 -5.947991
H -6.047645 5.777815 -5.662974
H -5.290402 3.545628 -5.219755
C -5.166609 0.007455 -3.951093
C -5.628903 -0.412367 -5.141322
C -5.116750 0.119858 -6.327743
C -5.595617 -0.327045 -7.570091
C -5.119143 0.203003 -8.708787
C -4.142862 1.208031 -8.675869
C -3.658776 1.661037 -7.505805
C -4.123543 1.130896 -6.279031
C -3.632038 1.578185 -5.019878
H -5.606811 -0.421686 -3.068614
H -6.408568 -1.167344 -5.178020
H -6.355541 -1.098074 -7.618477
H -5.495818 -0.144381 -9.665315
H -3.774889 1.623629 -9.607780
H -2.898938 2.430216 -7.527808

TS$_{53-54}$

Rh 1.411235 1.254484 -0.141509
P -0.883214 1.846715 0.106677
P 1.001444 -1.197103 -0.362627
C -0.917628 3.485638 0.875545
C -1.901706 1.878119 -1.435140
C 2.295464 -1.810278 -1.471063
C 0.954097 -2.216113 1.177180
C -1.685822 0.648717 1.240975
C -0.598668 -1.485469 -1.206899
N 5.058675 -0.490353 0.609103
C 4.389349 0.495584 -0.004501
C 3.569543 1.448706 0.622414
C 3.108497 2.665014 -0.024749
C 3.005168 3.887625 0.877878
C 3.599565 3.007461 -1.433920
H 4.554417 0.560876 -1.074812
H 1.248932 2.710935 -0.702005
H 3.485088 1.419521 1.703199
H 4.649124 3.329815 -1.399427
H 3.011364 3.832530 -1.842787
H 3.511245 2.168715 -2.130925
H 2.286421 4.611969 0.482529
H 3.983346 4.384277 0.936698
H 2.700525 3.621083 1.892748
C 5.986731 -1.363134 -0.135722
H 5.894808 -1.163363 -1.204747
H 5.742731 -2.412643 0.048745
H 7.018555 -1.175778 0.175720
C 4.992407 -0.681250 2.070285
H 3.951863 -0.718578 2.400247
H 5.502576 0.134939 2.592568
H 5.475845 -1.623769 2.324723
C -0.142773 3.700513 2.015342
C -0.086488 4.968102 2.607502
C -0.793427 6.029131 2.034312
C -1.545531 5.828057 0.872680
C -1.596121 4.555105 0.289886
H 0.405881 2.875686 2.445054
H 0.506671 5.132148 3.503038
H -0.751142 7.014110 2.486530
H -2.079185 6.660125 0.421271
H -2.147904 4.413895 -0.622865
C -1.304089 1.611433 -2.675996
C -2.075666 1.578098 -3.842009
C -3.450647 1.807751 -3.782488
C -4.053584 2.072315 -2.553101
C -3.288108 2.099644 -1.383106
H -0.241141 1.414972 -2.731201
H -1.604321 1.363705 -4.791637
H -4.049362 1.774443 -4.687746
H -5.121250 2.237899 -2.498998
H -3.770256 2.282597 -0.431304
C 2.576962 -1.071554 -2.622033
C 3.553542 -1.510350 -3.524448
C 4.251284 -2.693926 -3.264623
C 3.985754 -3.428173 -2.104212
C 3.012852 -2.976015 -1.203315
H 2.025175 -0.165254 -2.814816
H 3.770595 -0.938980 -4.422262
H 5.010486 -3.037743 -3.958217
H 4.546727 -4.334480 -1.894945
H 2.842345 -3.518138 -0.291223
C 1.177935 -1.605357 2.419058
C 1.087804 -2.347512 3.601275
C 0.769785 -3.705257 3.556853
C 0.536541 -4.319510 2.326053
C 0.616414 -3.579006 1.142118
H 1.407177 -0.548401 2.465413
H 1.262065 -1.863688 4.552541
H 0.698156 -4.279247 4.475246
H 0.278168 -5.368929 2.285782
H 0.407096 -4.059514 0.194990
C -2.018987 1.047774 2.550703
C -2.457334 0.174262 3.474106
C -2.615703 -1.177158 3.152443
C -3.060279 -2.090436 4.122373
C -3.248252 -3.381022 3.799741
C -3.010980 -3.833600 2.494551
C -2.584185 -2.989056 1.538957
C -2.358892 -1.624238 1.831651
C -1.904717 -0.700024 0.850154
H -1.929971 2.077459 2.843557
H -2.690084 0.523189 4.475893
H -3.259520 -1.760007 5.135012
H -3.593337 -4.081465 4.553196
H -3.172535 -4.879106 2.255653
H -2.403594 -3.381369 0.547348
C -0.611738 -2.034506 -2.505690
C -1.749660 -2.189043 -3.204432
C -2.977683 -1.817220 -2.649658
C -4.164000 -1.971687 -3.385343
C -5.345438 -1.634948 -2.841704
C -5.413616 -1.128781 -1.536482
C -4.299471 -0.965251 -0.801245
C -3.030740 -1.299515 -1.330388
C -1.827679 -1.134928 -0.587828
H 0.304612 -2.351659 -2.971253
H -1.712063 -2.609784 -4.204816
H -4.135038 -2.367670 -4.393638
H -6.258684 -1.759862 -3.414306
H -6.378808 -0.866224 -1.116923
H -4.397388 -0.562110 0.197463

TS$_{95-96}$

Rh 1.454144 1.207406 -0.224898
P -0.795199 1.807844 0.338294
P 0.984454 -1.157353 -0.699815
C -0.679902 3.316528 1.329750
C -1.974851 2.067314 -1.059190
C 2.174725 -1.653581 -1.971450
C 1.056088 -2.340842 0.716482
C -1.493016 0.477458 1.385236
C -0.693842 -1.330165 -1.412733
N 4.501418 -0.369693 1.443653
C 3.899863 0.753460 1.032768
C 3.677776 1.201295 -0.292553
C 3.223054 2.560045 -0.550480
C 3.280293 3.013439 -2.004293
C 3.559077 3.685247 0.433674
H 1.525739 2.799312 -0.178743
H 3.587382 1.406511 1.838711
H 4.035422 0.620173 -1.135780
H 2.967249 4.575848 0.203514
H 4.620711 3.945696 0.339398
H 3.354554 3.424082 1.474288
H 4.285470 3.394352 -2.231160
H 2.567019 3.821387 -2.186869
H 3.057372 2.194157 -2.691865
C 4.645663 -0.671661 2.881652
H 5.705065 -0.672281 3.157660
H 4.226063 -1.658130 3.099753
H 4.120772 0.079066 3.473229
C 5.079433 -1.376542 0.540834
H 4.978649 -1.069387 -0.499014
H 4.571677 -2.336641 0.679090
H 6.141936 -1.507464 0.765438
C 0.299009 3.375944 2.323467
C 0.481397 4.546465 3.069568
C -0.309825 5.667108 2.799326
C -1.270688 5.623101 1.783921
C -1.444535 4.447109 1.042647
H 0.909291 2.506730 2.519739
H 1.234086 4.589711 3.852111
H -0.171992 6.577350 3.372853
H -1.870607 6.502671 1.566373
H -2.159911 4.428776 0.239649
C -1.519394 1.978864 -2.382998
C -2.412922 2.112841 -3.450654
C -3.769347 2.333806 -3.209587
C -4.231398 2.420835 -1.896212
C -3.343601 2.279597 -0.824917
H -0.471830 1.790773 -2.580982
H -2.050420 2.035934 -4.466773
H -4.462706 2.430979 -4.039110
H -5.284035 2.578011 -1.702804
H -3.717406 2.322737 0.190105
C 2.352255 -0.810506 -3.069546
C 3.297624 -1.119232 -4.055061
C 4.075942 -2.273782 -3.926801
C 3.915228 -3.112539 -2.819251
C 2.965268 -2.795193 -1.840160
H 1.746562 0.078116 -3.154375
H 3.432267 -0.466975 -4.913218
H 4.813765 -2.515627 -4.683859
H 4.535264 -3.998498 -2.714451
H 2.871876 -3.420181 -0.970351
C 1.389226 -1.874012 1.995879
C 1.387403 -2.744425 3.091002
C 1.055194 -4.088578 2.920908
C 0.717838 -4.560803 1.652137
C 0.706292 -3.691544 0.556407
H 1.629678 -0.829255 2.142384
H 1.642638 -2.369473 4.072497
H 1.053016 -4.761953 3.772082
H 0.448097 -5.599200 1.515782
H 0.417900 -4.062607 -0.418711
C -1.648877 0.697092 2.767865
C -2.009412 -0.286895 3.610139
C -2.254315 -1.577153 3.129342
C -2.617415 -2.606554 4.013059
C -2.880417 -3.838056 3.544864
C -2.801588 -4.113354 2.172778
C -2.456349 -3.154049 1.295790
C -2.160413 -1.844574 1.739633
C -1.787606 -0.803515 0.844625
H -1.474499 1.673145 3.184179
H -2.107243 -0.076642 4.671062
H -2.691980 -2.413910 5.076824
H -3.161893 -4.628680 4.232650
H -3.019998 -5.114327 1.816968
H -2.396735 -3.411835 0.247129
C -0.838709 -1.712243 -2.761555
C -2.039515 -1.765401 -3.363453
C -3.202602 -1.453284 -2.653129
C -4.454984 -1.502111 -3.286870
C -5.573692 -1.227002 -2.595801
C -5.510118 -0.891297 -1.236484
C -4.329452 -0.832498 -0.595236
C -3.122454 -1.106637 -1.280129
C -1.852911 -1.048950 -0.639626
H 0.024436 -1.978288 -3.345542
H -2.104042 -2.057483 -4.407351
H -4.527923 -1.766824 -4.335154
H -6.538468 -1.269764 -3.090629
H -6.426602 -0.676226 -0.697670
H -4.325726 -0.559646 0.451246

TS$_{97-101}$

Rh 2.027263 0.156237 -0.816994
P 0.226352 -1.804759 -0.298399
P 0.289788 1.836952 -0.767104
C 0.610385 -3.237548 -1.344805
C 0.113823 -2.373051 1.455728
C 0.863097 3.556002 -0.777845
C -1.043734 1.793597 -2.043250
C -1.501098 -1.290917 -0.683156
C -0.414777 1.500591 0.885777
N 4.233319 0.744344 2.084096
C 4.151076 0.517759 0.768131
C 3.863488 -0.711620 0.101124
C 4.012929 -0.883766 -1.304559
C 3.888293 -2.284166 -1.863177
C 4.853036 0.018926 -2.193082
H 4.420856 1.369067 0.159194
H 2.859050 1.374125 -1.276658
H 3.624474 -1.592454 0.686155
H 4.376765 0.130325 -3.172222
H 5.014244 1.019333 -1.789470
H 5.835013 -0.447594 -2.357525
H 3.363462 -2.277079 -2.824773
H 4.883600 -2.709470 -2.048430
H 3.357930 -2.957794 -1.184771
C 4.107546 -0.277567 3.135927
H 4.998257 -0.240862 3.770683
H 3.229828 -0.070760 3.755495
H 4.031060 -1.273931 2.705583
C 4.426123 2.119015 2.586497
H 3.545448 2.433089 3.157139
H 5.305388 2.162106 3.234991
H 4.562578 2.803933 1.748173
C 0.669949 -3.059824 -2.728086
C 0.996861 -4.131001 -3.568572
C 1.286675 -5.380608 -3.012786
C 1.258607 -5.558327 -1.626071
C 0.926737 -4.481101 -0.794583
H 0.458625 -2.088553 -3.143602
H 1.034096 -3.994579 -4.645569
H 1.546509 -6.212484 -3.658142
H 1.505588 -6.525820 -1.197764
H 0.947700 -4.610806 0.273467
C 0.924411 -1.782255 2.432001
C 0.821101 -2.171673 3.771819
C -0.101861 -3.146178 4.151825
C -0.927584 -3.726988 3.188778
C -0.830398 -3.336522 1.849368
H 1.609891 -0.998340 2.147445
H 1.449030 -1.703357 4.516643
H -0.185014 -3.442389 5.192650
H -1.654339 -4.473798 3.478632
H -1.487682 -3.781052 1.113110
C 1.838997 3.940019 -1.697187
C 2.276177 5.269610 -1.752983
C 1.706970 6.221166 -0.901260
C 0.688413 5.854178 -0.016640
C 0.257389 4.522539 0.029601
H 2.226164 3.219794 -2.398194
H 3.038411 5.568694 -2.467138
H 2.037900 7.253000 -0.945886
H 0.225318 6.604242 0.618526
H -0.560560 4.249923 0.676627
C -0.960508 0.894930 -3.115917
C -1.963844 0.856229 -4.089838
C -3.053552 1.723982 -4.011319
C -3.133328 2.637119 -2.959907
C -2.133437 2.677772 -1.982603
H -0.115896 0.224496 -3.188634
H -1.892887 0.150390 -4.905998
H -3.833936 1.690811 -4.764986

H -3.976704 3.310979 -2.891250
H -2.213787 3.383379 -1.166597
C -2.251715 -2.027482 -1.622617
C -3.505606 -1.683274 -1.965082
C -4.123763 -0.576689 -1.375022
C -5.430652 -0.214887 -1.740593
C -6.030552 0.835005 -1.154799
C -5.366475 1.581173 -0.171197
C -4.114598 1.265597 0.205509
C -3.438447 0.173284 -0.385998
C -2.112122 -0.187227 -0.021540
H -1.834894 -2.905970 -2.081040
H -4.042733 -2.275579 -2.699649
H -5.964780 -0.781706 -2.494027
H -7.041410 1.107666 -1.440089
H -5.871684 2.422815 0.290419
H -3.634867 1.871817 0.962058
C 0.289784 2.021647 1.987686
C -0.051472 1.738297 3.255825
C -1.103430 0.856688 3.522837
C -1.454053 0.553584 4.848475
C -2.437724 -0.324903 5.103434
C -3.122623 -0.955323 4.055724
C -2.814857 -0.692413 2.773246
C -1.795106 0.233358 2.451327
C -1.431496 0.533482 1.107566
H 1.149653 2.652578 1.813778
H 0.513846 2.174195 4.073883
H -0.931282 1.022388 5.673767
H -2.703628 -0.555226 6.129830
H -3.907063 -1.668211 4.285294
H -3.359924 -1.212981 1.998341

TS$_{97-115}$

Rh 1.878271 0.370701 -0.283871
P 0.169943 -1.659022 0.082432
P 0.069017 1.946496 -0.511223
C 0.784049 -3.141432 -0.767594
C -0.034832 -2.071006 1.870058
C 0.560169 3.685818 -0.538713
C -1.048193 1.733200 -1.966323
C -1.543594 -1.316267 -0.492609
C -0.869047 1.664804 1.033761
N 5.651437 -0.782750 1.414017
C 4.382128 -0.546934 1.189096
C 3.668549 -0.793201 -0.071041
C 3.779161 0.086859 -1.227189
C 3.576041 -0.513461 -2.610196
C 4.767118 1.250771 -1.246608
H 2.628465 1.654570 0.157631
H 3.841292 -0.130184 2.037163
H 3.475380 -1.852824 -0.261214
H 4.426507 2.022001 -1.942354
H 4.888797 1.724067 -0.268921
H 5.751644 0.910026 -1.604399
H 3.097037 0.210383 -3.276492
H 4.543026 -0.785424 -3.060510
H 2.959755 -1.414095 -2.579701
C 6.562342 -1.361480 0.397426
H 7.355774 -0.642974 0.178024
H 7.004371 -2.276182 0.800079
H 6.002208 -1.584471 -0.507772
C 6.281233 -0.486897 2.725951
H 5.540324 -0.059354 3.401185
H 6.680607 -1.410993 3.150740
H 7.097494 0.224606 2.578795
C 0.928710 -3.110770 -2.155026
C 1.444841 -4.216986 -2.840825
C 1.834760 -5.352363 -2.124407
C 1.718014 -5.380200 -0.731226
C 1.198653 -4.268412 -0.055801
H 0.630728 -2.226810 -2.695961
H 1.549052 -4.195199 -3.921866
H 2.240196 -6.210408 -2.649146
H 2.041844 -6.256538 -0.176599
H 1.148689 -4.277585 1.019556
C 0.693205 -1.364357 2.837860
C 0.511309 -1.633780 4.198224
C -0.398051 -2.609996 4.606733
C -1.127568 -3.317295 3.650470
C -0.954716 -3.046496 2.289292
H 1.388544 -0.593965 2.532266
H 1.075947 -1.078712 4.934821
H -0.539946 -2.815602 5.662986
H -1.838579 -4.070847 3.961168
H -1.537659 -3.589568 1.556520
C 1.775777 4.036945 -1.120121
C 2.173764 5.378962 -1.170522
C 1.335383 6.368750 -0.646820
C 0.099730 6.023435 -0.089408
C -0.288705 4.678505 -0.046053
H 2.390295 3.273721 -1.565512
H 3.120259 5.655631 -1.626830
H 1.637179 7.409652 -0.688313
H -0.555712 6.797960 0.299220
H -1.246910 4.407802 0.368308
C -0.740753 0.774794 -2.943018
C -1.580563 0.589259 -4.045989
C -2.729862 1.366221 -4.192877
C -3.034087 2.334828 -3.236444
C -2.198267 2.522822 -2.130673
H 0.149241 0.168902 -2.838702
H -1.336760 -0.160503 -4.786133
H -3.383552 1.218857 -5.046764
H -3.926663 2.936850 -3.341438
H -2.458373 3.265322 -1.389781
C -2.135048 -2.165555 -1.448829
C -3.353942 -1.924748 -1.962545
C -4.094270 -0.815985 -1.541361
C -5.362198 -0.559830 -2.088263
C -6.083189 0.490451 -1.661319
C -5.586193 1.341548 -0.664447
C -4.376591 1.128863 -0.116435
C -3.576049 0.041688 -0.537957
C -2.288031 -0.211069 0.009834
H -1.617993 -3.047303 -1.781743
H -3.766113 -2.600982 -2.705375
H -5.768970 -1.209388 -2.854378
H -7.063331 0.681086 -2.086289
H -6.187870 2.180857 -0.332405
H -4.027253 1.813875 0.644232
C -0.426134 2.353407 2.179036
C -0.928027 2.108443 3.401089
C -1.893369 1.113176 3.581941
C -2.408598 0.850707 4.861785
C -3.320568 -0.119731 5.037457
C -3.768418 -0.884825 3.952122
C -3.297393 -0.664809 2.711878
C -2.339514 0.348370 2.472921
C -1.809415 0.608095 1.176907
H 0.360448 3.087335 2.085725
H -0.562684 2.671397 4.254865
H -2.073647 1.427448 5.715871
H -3.715212 -0.317257 6.028735
H -4.501429 -1.666774 4.118358
H -3.661916 -1.288277 1.907261

TS$_{102-107}$

Rh 1.948273 0.018678 -0.553817
P 0.242330 -1.792126 -0.312328
P 0.292721 1.767409 -0.464949
C 0.652636 -3.160889 -1.428416
C 0.126834 -2.440629 1.414015
C 0.952002 3.451442 -0.380499
C -0.972399 1.853686 -1.807048
C -1.480045 -1.270858 -0.708887
C -0.520511 1.390261 1.126334
N 4.520881 1.538235 1.228189
C 4.278248 0.812458 0.092311
C 3.956005 -0.616649 0.145016
C 3.842895 -1.408518 -1.007001
C 3.867176 -2.912525 -0.820991
C 4.200593 -0.945358 -2.405504
H 4.857195 1.149057 -0.765424
H 3.019145 1.295819 -0.548026
H 3.943983 -1.106097 1.110687
H 3.512593 -1.390388 -3.132139
H 4.163163 0.137372 -2.536209
H 5.210375 -1.295712 -2.665229
H 3.419879 -3.435921 -1.670341
H 4.910604 -3.249702 -0.763694
H 3.362357 -3.230800 0.095598
C 3.935240 1.125953 2.512261
H 4.143863 1.892949 3.257459
H 2.846537 1.003994 2.416266
H 4.367380 0.184418 2.859268
C 4.862856 2.961430 1.084346
H 3.956784 3.588170 1.080497
H 5.504985 3.274807 1.908843
H 5.397137 3.121157 0.145621
C 0.869298 -2.872449 -2.776728
C 1.197259 -3.893065 -3.677420
C 1.320751 -5.207103 -3.216059
C 1.125027 -5.500030 -1.862644
C 0.795452 -4.471932 -0.970019
H 0.777301 -1.855076 -3.119240
H 1.360091 -3.669247 -4.727906
H 1.578793 -6.001227 -3.908100
H 1.239152 -6.520074 -1.506239
H 0.680173 -4.697140 0.074875
C 0.986916 -1.945008 2.404144
C 0.873899 -2.382607 3.727464
C -0.102316 -3.315138 4.078974
C -0.969516 -3.807162 3.103091
C -0.866209 -3.365225 1.780211
H 1.731784 -1.205793 2.147797
H 1.541638 -1.988098 4.481357
H -0.191859 -3.650066 5.107545
H -1.736463 -4.521027 3.371721
H -1.560007 -3.736054 1.036641
C 2.032738 3.796270 -1.190990
C 2.539203 5.101990 -1.179148
C 1.934040 6.070799 -0.372712
C 0.812979 5.745656 0.397339
C 0.313744 4.437304 0.376125
H 2.456567 3.061411 -1.855182
H 3.386312 5.368835 -1.804752
H 2.319361 7.084461 -0.364800
H 0.325483 6.510434 0.995508
H -0.576148 4.195142 0.934445
C -0.835705 1.059133 -2.953863
C -1.790115 1.115186 -3.975003
C -2.884180 1.974331 -3.868279
C -3.016991 2.784620 -2.740717
C -2.065107 2.731665 -1.717215
H 0.012029 0.395552 -3.047932
H -1.677583 0.489410 -4.849693
H -3.626667 2.014347 -4.658981
H -3.864133 3.451094 -2.650027
H -2.187540 3.356618 -0.842990
C -2.169369 -1.916070 -1.756902
C -3.414651 -1.562842 -2.119672
C -4.082666 -0.532886 -1.451552
C -5.376296 -0.153813 -1.845626
C -6.019975 0.830268 -1.195886
C -5.414712 1.490668 -0.117787
C -4.177735 1.155232 0.289630
C -3.458118 0.126974 -0.363026
C -2.144374 -0.250998 0.031098
H -1.707242 -2.724104 -2.294809
H -3.902299 -2.082338 -2.938991
H -5.863979 -0.652754 -2.674783
H -7.019920 1.117331 -1.504602
H -5.953670 2.281878 0.392371
H -3.744555 1.696701 1.119606
C 0.083264 1.895413 2.293136
C -0.324238 1.537731 3.522706
C -1.350490 0.600939 3.681769
C -1.770134 0.220718 4.966847
C -2.740594 -0.695887 5.117426
C -3.344366 -1.288260 3.999849
C -2.967638 -0.952622 2.753217
C -1.955104 0.011511 2.541102
C -1.521702 0.389984 1.239229
H 0.921701 2.570502 2.205844
H 0.165572 1.960384 4.394841
H -1.314112 0.662899 5.844815
H -3.061323 -0.984367 6.113067
H -4.122241 -2.029911 4.145479
H -3.451126 -1.444669 1.920686

TS$_{107-113}$

Rh 1.856669 -0.012090 -0.614132
P 0.211572 -1.753969 -0.350910
P 0.231138 1.761986 -0.561696
C 0.634345 -3.139178 -1.440879
C 0.133805 -2.350026 1.395947
C 0.962209 3.413868 -0.519602
C -1.056319 1.857181 -1.880053
C -1.522309 -1.256401 -0.725585
C -0.553131 1.433617 1.050056
N 4.078029 1.323610 1.625873
C 4.039889 0.789149 0.296556
C 3.818942 -0.682073 0.136918
C 3.835309 -1.312903 -1.111950
C 3.916887 -2.823924 -1.149429
C 4.226353 -0.629999 -2.407883
H 4.864743 1.150135 -0.346356
H 3.131249 1.324862 -0.262688
H 3.798375 -1.302473 1.022512
H 3.598527 -0.993833 -3.228039
H 4.142302 0.458920 -2.375168
H 5.266164 -0.883813 -2.660498
H 3.449805 -3.238980 -2.046872
H 4.972557 -3.124106 -1.180278
H 3.464240 -3.289572 -0.269196
C 4.595322 0.507395 2.734349
H 5.656908 0.235072 2.614089
H 4.493451 1.085594 3.654645
H 4.010683 -0.405741 2.861605
C 4.393595 2.755881 1.679722
H 3.714896 3.307120 1.017352
H 4.237966 3.126706 2.694116
H 5.428778 2.984680 1.375057
C 0.793102 -2.884737 -2.804204
C 1.121339 -3.921505 -3.686336
C 1.310345 -5.215158 -3.190630
C 1.179081 -5.471494 -1.822016
C 0.845068 -4.428277 -0.948770
H 0.653614 -1.881818 -3.173005
H 1.235738 -3.725195 -4.748646
H 1.570088 -6.021228 -3.868035
H 1.344949 -6.474851 -1.439601
H 0.778320 -4.623216 0.106568
C 0.958095 -1.766625 2.369347
C 0.869484 -2.165162 3.706741
C -0.051045 -3.140551 4.090508
C -0.888745 -3.713414 3.133042
C -0.808579 -3.312847 1.795435
H 1.646474 -0.981167 2.091443
H 1.507026 -1.701064 4.446945
H -0.123863 -3.443006 5.130359
H -1.615639 -4.458491 3.427070
H -1.480805 -3.746079 1.065892
C 2.087287 3.671331 -1.300285
C 2.656727 4.950826 -1.319532
C 2.074227 5.977729 -0.569381
C 0.917007 5.735294 0.178242
C 0.354520 4.452680 0.187657
H 2.499294 2.884740 -1.911911
H 3.536359 5.152398 -1.924336
H 2.508461 6.971318 -0.585264
H 0.452496 6.543152 0.736731
H -0.555608 4.267598 0.735663
C -0.954529 1.049819 -3.021526
C -1.930864 1.107344 -4.021710
C -3.012619 1.979808 -3.898778
C -3.111273 2.801772 -2.776156
C -2.137317 2.747459 -1.773653
H -0.117500 0.374401 -3.127317
H -1.845090 0.471864 -4.892379
H -3.772232 2.020754 -4.672967
H -3.949025 3.478099 -2.672615
H -2.234404 3.380141 -0.901904
C -2.221425 -1.928006 -1.749696
C -3.474603 -1.591821 -2.101273
C -4.141306 -0.554060 -1.443822
C -5.442330 -0.191015 -1.828596
C -6.084628 0.800758 -1.189281
C -5.470590 1.485160 -0.131320
C -4.226491 1.165428 0.266903
C -3.508062 0.129750 -0.375194
C -2.187102 -0.232179 0.009590
H -1.759974 -2.743510 -2.276641
H -3.969565 -2.131017 -2.903243
H -5.936714 -0.708412 -2.642304
H -7.090155 1.075541 -1.490868
H -6.008384 2.282407 0.370551
H -3.786654 1.725406 1.080932
C 0.073683 1.958212 2.195808
C -0.321381 1.632098 3.438461
C -1.349877 0.703676 3.631910
C -1.752269 0.351768 4.930502
C -2.716898 -0.565071 5.114164
C -3.330867 -1.186717 4.018110
C -2.971666 -0.877886 2.759436
C -1.968218 0.087236 2.512802
C -1.553110 0.437387 1.197571
H 0.923155 2.615643 2.082179
H 0.185736 2.067659 4.294011
H -1.286125 0.814874 5.792160
H -3.023754 -0.832083 6.120077
H -4.102436 -1.929203 4.190460
H -3.461830 -1.391887 1.944229

TS$_{113-114}$

Rh 1.822666 0.022184 -0.706399
P 0.233470 -1.720118 -0.343778
P 0.170690 1.787449 -0.610973
C 0.668542 -3.137579 -1.386442
C 0.195224 -2.245129 1.428157
C 0.927176 3.427542 -0.571634
C -1.116687 1.864298 -1.931292
C -1.516392 -1.274799 -0.717066
C -0.616160 1.481451 1.005306
N 3.987643 0.902869 1.955498
C 4.050212 0.678091 0.515523
C 3.737728 -0.733969 0.090841
C 3.825311 -1.171308 -1.236387
C 3.942782 -2.650457 -1.522968
C 4.272038 -0.273352 -2.374420
H 4.994933 1.031742 0.049095
H 3.271476 1.369645 0.060348
H 3.646920 -1.480108 0.871765
H 3.773298 -0.561817 -3.304693
H 4.079632 0.789666 -2.196605
H 5.354384 -0.384059 -2.535987
H 3.484586 -2.926861 -2.476114
H 5.006715 -2.911413 -1.599215
H 3.512023 -3.265808 -0.728147
C 4.993593 0.152665 2.733480
H 6.028004 0.467351 2.504534
H 4.809207 0.319210 3.796852
H 4.908274 -0.919031 2.541499
C 3.967046 2.335114 2.299764
H 3.165402 2.834455 1.743579
H 3.765504 2.449806 3.366469
H 4.915693 2.850768 2.064285
C 0.767190 -2.941879 -2.765364
C 1.089279 -4.009556 -3.612392
C 1.337943 -5.272469 -3.066353
C 1.269111 -5.468287 -1.683259
C 0.936707 -4.395929 -0.845460
H 0.582223 -1.960918 -3.173179
H 1.154857 -3.860042 -4.686371
H 1.595075 -6.101516 -3.716439
H 1.480417 -6.447582 -1.262793
H 0.916328 -4.543091 0.219675
C 1.001439 -1.588673 2.370816
C 0.939337 -1.936689 3.723548
C 0.063131 -2.932730 4.154473
C -0.757666 -3.577246 3.228474
C -0.704067 -3.228042 1.875185
H 1.653351 -0.785419 2.058513
H 1.564206 -1.417139 4.437401
H 0.011125 -3.195824 5.206163
H -1.451212 -4.338636 3.558816
H -1.363879 -3.717103 1.170015
C 2.165370 3.616541 -1.180182
C 2.774377 4.877725 -1.166105
C 2.123624 5.952620 -0.551151
C 0.865451 5.773856 0.033805
C 0.265671 4.508430 0.012717
H 2.633672 2.793727 -1.693381
H 3.738985 5.028376 -1.642605
H 2.588762 6.932241 -0.542417
H 0.356899 6.615497 0.495744
H -0.705593 4.363428 0.457768
C -1.009338 1.037558 -3.058865
C -1.988996 1.065816 -4.056963
C -3.081939 1.925430 -3.945009
C -3.190050 2.761863 -2.834161
C -2.213206 2.736574 -1.833251
H -0.166638 0.366619 -3.154085
H -1.897793 0.416474 -4.916792
H -3.844361 1.943296 -4.717323

H -4.038927 3.425170 -2.737205
H -2.324829 3.374068 -0.967908
C -2.210728 -1.991013 -1.714713
C -3.469595 -1.684844 -2.073176
C -4.150021 -0.637278 -1.446989
C -5.457156 -0.305598 -1.839337
C -6.114412 0.692744 -1.226033
C -5.510256 1.414694 -0.187572
C -4.260708 1.125590 0.216968
C -3.526054 0.085226 -0.399040
C -2.198800 -0.245478 -0.007051
H -1.743480 -2.819590 -2.213896
H -3.959172 -2.257693 -2.854913
H -5.944462 -0.853121 -2.637450
H -7.124640 0.943090 -1.533101
H -6.060251 2.216125 0.293966
H -3.829170 1.714145 1.015090
C -0.053134 2.090242 2.142507
C -0.455581 1.786590 3.388623
C -1.434506 0.809219 3.596725
C -1.842961 0.480312 4.899464
C -2.767909 -0.473794 5.097252
C -3.335233 -1.155968 4.012053
C -2.968740 -0.870481 2.749972
C -2.003452 0.129059 2.488849
C -1.582445 0.456512 1.169891
H 0.749606 2.799234 2.024429
H 0.005468 2.282647 4.237261
H -1.414113 0.992009 5.752947
H -3.079646 -0.722865 6.106251
H -4.076156 -1.926378 4.195823
H -3.421568 -1.430548 1.943424
TS$_{116-123}$
Rh -2.652511 -2.666394 1.495124
P -1.687211 -0.850337 2.739279
P -1.584346 -1.922213 -0.654003
C -2.312205 -0.632824 4.426190
C 0.148564 -0.806981 2.937740
C -1.027779 -3.390307 -1.559701
C -2.702070 -0.931921 -1.742857
C -2.235987 0.585543 1.750857
C -0.109942 -0.842205 -0.420332
N -5.322454 -4.236818 3.529436
C -4.844552 -3.381398 2.577339
C -4.588109 -3.709395 1.160559
C -3.694378 -4.666840 0.655002
C -3.819876 -5.011097 -0.818590
C -2.912817 -5.685768 1.462600
H -3.353191 -3.104122 2.933793
H -5.179662 -2.359400 2.738809
H -5.208055 -3.173059 0.449194
H -3.386672 -6.675944 1.393509
H -2.806492 -5.417901 2.513493
H -1.907321 -5.790179 1.041189
H -4.591742 -5.780940 -0.949619
H -2.891912 -5.433266 -1.214565
H -4.102406 -4.148431 -1.428802
C -5.451197 -5.689593 3.363761
H -6.363663 -6.011132 3.873111
H -4.603081 -6.233662 3.795835
H -5.535271 -5.935051 2.305785
C -5.525695 -3.724390 4.892664
H -4.679923 -3.990147 5.540551
H -6.440645 -4.145493 5.315392
H -5.612276 -2.634877 4.869091
C -2.489615 -1.754592 5.234399
C -2.928193 -1.613377 6.557098
C -3.159328 -0.336146 7.076494
C -2.927856 0.795950 6.289393
C -2.486606 0.643129 4.969056
H -2.249291 -2.732345 4.851275
H -3.070456 -2.488153 7.185393
H -3.492948 -0.222342 8.102080
H -3.074646 1.787360 6.708874
H -2.262212 1.515894 4.377434
C 0.924491 -1.902559 2.533083
C 2.315682 -1.879558 2.677152
C 2.946179 -0.768606 3.238124
C 2.180622 0.317496 3.662266
C 0.789324 0.299437 3.519320
H 0.444582 -2.769459 2.100621
H 2.902941 -2.727164 2.351463
H 4.026058 -0.749743 3.345853
H 2.663215 1.183388 4.095122
H 0.209686 1.154022 3.841014
C -0.239707 -4.322927 -0.883923
C 0.199838 -5.483497 -1.532145
C -0.166176 -5.711731 -2.862082
C -0.972074 -4.790846 -3.539095
C -1.406047 -3.632485 -2.881636
H 0.031247 -4.138331 0.143010
H 0.817783 -6.206817 -1.007615
H 0.167805 -6.610890 -3.368163
H -1.268284 -4.981680 -4.566902
H -2.055935 -2.946775 -3.394649
C -4.026581 -0.689935 -1.351051
C -4.866834 0.103040 -2.139147
C -4.392624 0.671342 -3.321593
C -3.072263 0.448571 -3.712798
C -2.226155 -0.337480 -2.924175
H -4.396323 -1.099058 -0.421620
H -5.884973 0.284478 -1.822499
H -5.044437 1.291436 -3.928867
H -2.694472 0.897900 -4.621302
H -1.197039 -0.484060 -3.226211
C -3.532794 1.074944 1.996799
C -4.091407 2.034330 1.239460
C -3.408336 2.546435 0.131572
C -3.996794 3.541506 -0.665747
C -3.353676 4.007842 -1.748996
C -2.093440 3.507116 -2.103063
C -1.498334 2.552529 -1.365999
C -2.128977 2.035775 -0.210354
C -1.537471 1.020972 0.594176
H -4.112166 0.654399 2.805561
H -5.088886 2.392681 1.475583
H -4.973647 3.937693 -0.414621
H -3.813412 4.777273 -2.360569
H -1.595996 3.893378 -2.986106
H -0.532455 2.186182 -1.684719
C 1.147481 -1.280865 -0.884817
C 2.267063 -0.556852 -0.711689
C 2.218394 0.680644 -0.063216
C 3.392468 1.426270 0.131730
C 3.341090 2.620321 0.745482
C 2.120314 3.140202 1.197648
C 0.974761 2.456261 1.028533
C 0.976974 1.194078 0.390273
C -0.211282 0.435947 0.199344
H 1.238471 -2.218902 -1.402265
H 3.214966 -0.938969 -1.078372
H 4.346492 1.044668 -0.212509
H 4.251812 3.192002 0.891632
H 2.102834 4.106693 1.689892
H 0.056152 2.889141 1.400765
TS$_{124-127}$
Rh 1.710280 0.100773 -0.581483
P 0.171021 -1.684476 -0.229505
P -0.002710 1.842587 -0.467482
C 0.710159 -3.087972 -1.241578
C 0.030012 -2.224310 1.533734
C 0.717406 3.492735 -0.307502
C -1.218616 1.924660 -1.855810
C -1.564826 -1.289722 -0.713011
C -0.864229 1.469887 1.095527
N 5.346982 0.791808 1.252470
C 3.926576 0.475575 1.237399
C 3.523335 -0.738429 0.379153
C 3.749558 -0.889977 -1.001555
C 3.947367 -2.260679 -1.607135
C 4.275949 0.237845 -1.866017
H 3.388602 1.381360 0.905607
H 3.568648 0.282981 2.258554
H 3.352660 -1.661183 0.930889
H 3.853726 0.183465 -2.874993
H 4.091530 1.231922 -1.450294
H 5.368184 0.156469 -1.963700
H 3.543068 -2.325213 -2.621327
H 5.024069 -2.462627 -1.681436
H 3.512195 -3.056502 -0.996579
C 6.298161 -0.271158 1.553761
H 6.620425 -0.848540 0.669021
H 7.194812 0.160991 2.011105
H 5.860355 -0.971954 2.273672
C 5.864395 2.019938 0.663246
H 5.081169 2.786002 0.654313
H 6.692422 2.402996 1.271682
H 6.241224 1.902382 -0.368217
C 0.860105 -2.898482 -2.616640
C 1.275033 -3.954643 -3.437323
C 1.565432 -5.198004 -2.867761
C 1.447353 -5.385473 -1.486934
C 1.023952 -4.324865 -0.675660
H 0.644851 -1.930585 -3.042333
H 1.381193 -3.810492 -4.508799
H 1.894457 -6.017428 -3.497345
H 1.692558 -6.348370 -1.047337
H 0.969075 -4.463269 0.389589
C 0.739970 -1.548926 2.536713
C 0.595836 -1.913924 3.878989
C -0.268364 -2.948389 4.237734
C -0.994676 -3.613751 3.249981
C -0.858848 -3.247066 1.907255
H 1.378287 -0.718243 2.276642
H 1.145816 -1.379747 4.641826
H -0.384677 -3.225529 5.280722
H -1.678798 -4.405857 3.523116
H -1.446111 -3.754358 1.152521
C 2.052023 3.684883 -0.647871
C 2.642176 4.948300 -0.517490
C 1.879909 6.020429 -0.041815
C 0.535833 5.833258 0.298214
C -0.042713 4.564841 0.161916
H 2.615616 2.860476 -1.039286
H 3.682118 5.101095 -0.792244
H 2.330737 7.001674 0.058100
H -0.052426 6.669035 0.666684
H -1.070589 4.409073 0.442782
C -1.017065 1.134121 -2.997090
C -1.938875 1.154334 -4.048910
C -3.069551 1.967740 -3.976987
C -3.275600 2.763010 -2.850142
C -2.357511 2.744962 -1.794836
H -0.146603 0.494604 -3.060962
H -1.773369 0.534555 -4.919445
H -3.786982 1.979427 -4.791398
H -4.157143 3.386125 -2.782304
H -2.554270 3.340893 -0.916609
C -2.175748 -2.019706 -1.754558
C -3.418112 -1.747264 -2.189562
C -4.167688 -0.726238 -1.600000
C -5.458264 -0.431919 -2.069418
C -6.184618 0.537081 -1.487855
C -5.670016 1.264783 -0.406113
C -4.438847 1.011244 0.071967
C -3.633120 0.003625 -0.508862
C -2.322268 -0.289328 -0.038709
H -1.658069 -2.834139 -2.225344
H -3.841531 -2.329114 -3.002753
H -5.877400 -0.985407 -2.901434
H -7.181705 0.758462 -1.854407
H -6.274948 2.041756 0.048910
H -4.077116 1.603568 0.901353
C -0.403283 2.088906 2.273255
C -0.879923 1.758645 3.486223
C -1.836651 0.746437 3.618570
C -2.323356 0.391857 4.887254
C -3.226948 -0.594363 5.013210
C -3.694031 -1.284770 3.886261
C -3.250829 -0.975140 2.654867
C -2.304246 0.058458 2.468815
C -1.805560 0.413609 1.184504
H 0.377354 2.831321 2.216714
H -0.500544 2.265475 4.368465
H -1.973300 0.909986 5.772286
H -3.599924 -0.862879 5.996157
H -4.419660 -2.080951 4.012397
H -3.627619 -1.542108 1.814685

3.2 Associative mechanism (ONIOM(B3LYP/BS1:UFF) level)

291
Rh -0.738339 0.677285 0.084699
P 0.050475 -1.452071 0.829321
P 1.359730 1.405347 -0.795857
C -1.197731 -2.028853 2.008532
C 0.400453 -2.777847 -0.411936
C 0.969351 2.389937 -2.267365
C 2.450056 2.369623 0.344343
C 1.604189 -1.163769 1.747879
C 2.409635 0.003876 -1.350727
N -4.616392 -1.146646 -0.591457
C -3.210359 -1.166571 -0.938225
C -2.905585 -0.972605 -2.405939
C -2.933821 -1.935562 -3.348208
C -2.576987 -1.621007 -4.784253
C -3.308016 -3.373819 -3.076941
H -2.606465 -0.363507 -0.357999
H -2.799712 -2.112795 -0.564942
H -2.625485 0.036197 -2.701273
H -3.557989 -3.558640 -2.029754
H -2.494021 -4.049963 -3.369132
H -4.182789 -3.658083 -3.675980
H -3.422818 -1.830082 -5.452376
H -1.743649 -2.246911 -5.127855
H -2.292800 -0.571676 -4.911916
C -5.449906 -0.078417 -1.145184
H -5.282560 0.902669 -0.659827
H -6.503139 -0.339346 -1.012842
H -5.251856 0.023250 -2.213882
C -4.913937 -1.524042 0.787702
H -4.543784 -0.785070 1.527742
H -4.442007 -2.484938 1.016127
H -5.992019 -1.630093 0.923050
C -1.725282 -1.105495 2.911890
C -2.707754 -1.494082 3.830785
C -3.174095 -2.812275 3.826185
C -2.670787 -3.734390 2.902856
C -1.690082 -3.334132 1.986059
H -1.354426 -0.092555 2.907976
H -3.109817 -0.778934 4.542878
H -3.937361 -3.118421 4.533145
H -3.051359 -4.752028 2.889614
H -1.333272 -4.035894 1.254157
C 0.169027 -2.538729 -1.773796
C 0.483101 -3.510165 -2.729544
C 1.038679 -4.728092 -2.337891
C 1.287340 -4.969665 -0.986718
C 0.981308 -3.998056 -0.028324
H -0.227366 -1.584909 -2.091123
H 0.303167 -3.308586 -3.776251
H 1.287192 -5.479019 -3.081086
H 1.735760 -5.904314 -0.678134
H 1.203980 -4.185819 1.014230
C 0.136486 1.823676 -3.234716
C -0.218023 2.548649 -4.378572
C 0.252354 3.855527 -4.539611
C 1.066933 4.437198 -3.562408
C 1.419583 3.701361 -2.423691
H -0.226366 0.817109 -3.092868
H -0.858998 2.104605 -5.134961
H -0.022643 4.424138 -5.421176
H 1.415495 5.458985 -3.684663
H 2.015544 4.163438 -1.656803
C 2.079199 2.540919 1.686012
C 2.926537 3.206643 2.577649
C 4.158287 3.697477 2.144025
C 4.545580 3.513318 0.816437
C 3.705240 2.840358 -0.076312
H 1.145619 2.127269 2.043674
H 2.630406 3.326269 3.610865
H 4.817553 4.207978 2.838944
H 5.506992 3.875928 0.478437
H 4.029447 2.676763 -1.096081
C 1.602868 -1.206796 3.155683
C 2.672068 -0.830934 3.880076
C 3.836029 -0.383140 3.245641
C 4.944322 0.029714 4.002970
C 6.067708 0.438632 3.389481
C 6.153047 0.455969 1.990262
C 5.113237 0.067109 1.231226
C 3.907491 -0.364153 1.829187
C 2.784590 -0.777874 1.062144
H 0.729011 -1.541968 3.685924
H 2.626685 -0.870223 4.964361
H 4.902454 0.017942 5.085743
H 6.921127 0.755082 3.980169
H 7.069885 0.789318 1.516448
H 5.215728 0.105588 0.155100
C 2.720926 -0.132907 -2.719860
C 3.421315 -1.177578 -3.193916
C 3.886190 -2.170426 -2.327316
C 4.599607 -3.271594 -2.828292
C 5.061315 -4.215505 -1.991360
C 4.842342 -4.115780 -0.610628
C 4.164470 -3.075849 -0.093328
C 3.654155 -2.057943 -0.933070
C 2.924147 -0.946051 -0.426383
H 2.407063 0.613039 -3.426841
H 3.627413 -1.247225 -4.257736
H 4.784317 -3.366067 -3.891845
H 5.612973 -5.063111 -2.384618
H 5.224987 -4.889290 0.046465
H 4.008917 -3.045199 0.976367
N -1.830728 2.602583 0.086865
C -2.830768 2.312091 1.172535
H -2.296349 2.096388 2.099534
H -3.433312 1.447981 0.890995
H -3.493053 3.174471 1.326718
C -1.043233 3.813188 0.488745
H -0.268137 4.006562 -0.252422
H -0.589613 3.643159 1.462969
H -1.700097 4.691040 0.545016
C -2.547640 2.890409 -1.197950
H -3.055511 1.991684 -1.543392
H -1.822199 3.203714 -1.949523
H -3.278786 3.697722 -1.058499
359
Rh -1.043283 0.733261 0.731134
P -0.183476 -1.348110 1.418001
P 1.019071 1.470257 -0.532928
C -1.236766 -1.889449 2.791375
C -0.009367 -2.742476 0.206277
C 0.441507 2.420599 -1.967771
C 2.327882 2.432888 0.354816
C 1.509857 -1.065558 2.066114
C 1.925825 0.012171 -1.180175
N -3.587191 -1.581239 -1.835224
C -3.185988 -2.183660 -2.949182
C -2.625670 -1.543975 -4.088975
C -2.197147 -2.186140 -5.222845
C -1.590520 -1.388829 -6.344590
C -2.270446 -3.670989 -5.457006
H -2.374807 0.149707 1.379095
H -3.296576 -3.261670 -2.943302
H -2.507911 -0.466671 -4.057107
H -2.680214 -4.242223 -4.622236
H -1.273254 -4.068682 -5.684692
H -2.891544 -3.878482 -6.338980
H -2.156050 -1.533322 -7.275127
H -0.566519 -1.728604 -6.551125

H -1.558432 -0.318671 -6.124507
C -3.473881 -0.151215 -1.562652
H -2.579839 0.048869 -0.896949
H -4.355890 0.167951 -1.003824
H -3.395932 0.430377 -2.478145
C -4.038916 -2.369512 -0.667869
H -3.418009 -2.096221 0.194227
H -3.946350 -3.436330 -0.870079
H -5.079537 -2.126161 -0.437043
C -1.542617 -0.966736 3.794698
C -2.413142 -1.312376 4.835528
C -3.002979 -2.579772 4.849515
C -2.736415 -3.491925 3.823787
C -1.862154 -3.136940 2.788657
H -1.092537 0.014371 3.768007
H -2.636828 -0.601741 5.626439
H -3.681431 -2.852096 5.650736
H -3.218422 -4.465871 3.824722
H -1.700564 -3.823822 1.976571
C -0.370034 -2.557122 -1.135832
C -0.172111 -3.578670 -2.070391
C 0.378975 -4.797428 -1.677649
C 0.745779 -4.989995 -0.345918
C 0.566685 -3.965800 0.589330
H -0.769929 -1.606022 -1.458037
H -0.443517 -3.413922 -3.103232
H 0.532049 -5.586821 -2.406462
H 1.190164 -5.926574 -0.037809
H 0.882768 -4.115809 1.613651
C -0.502261 1.832876 -2.812202
C -1.023232 2.544079 -3.899398
C -0.610427 3.861159 -4.122687
C 0.309626 4.467405 -3.260720
C 0.826760 3.745290 -2.177266
H -0.818166 0.818172 -2.622170
H -1.748533 2.081685 -4.562936
H -1.013860 4.419063 -4.960570
H 0.610265 5.498230 -3.426342
H 1.499155 4.227759 -1.489560
C 2.217838 2.657738 1.734720
C 3.229210 3.331042 2.427541
C 4.366161 3.777796 1.753805
C 4.494136 3.541600 0.384739
C 3.489148 2.861253 -0.310331
H 1.361845 2.277924 2.275319
H 3.134606 3.491044 3.492898
H 5.152726 4.294965 2.294212
H 5.380201 3.872013 -0.140529
H 3.610804 2.661566 -1.367272
C 1.738868 -1.094949 3.455801
C 2.915730 -0.727787 3.992759
C 3.969199 -0.309931 3.173297
C 5.190706 0.092984 3.737276
C 6.210148 0.466391 2.945728
C 6.073737 0.454813 1.550701
C 4.920185 0.073975 0.973943
C 3.815116 -0.318156 1.763597
C 2.577126 -0.721876 1.191671
H 0.963990 -1.419470 4.126092
H 3.046229 -0.755543 5.070599
H 5.320935 0.101217 4.813059
H 7.151932 0.775291 3.387333
H 6.910118 0.759033 0.930911
H 4.853277 0.089366 -0.105514
C 2.057794 -0.170237 -2.572168
C 2.639068 -1.261989 -3.097824
C 3.160033 -2.256966 -2.265964
C 3.757396 -3.401599 -2.818571
C 4.283512 -4.343154 -2.017822
C 4.248559 -4.197556 -0.624295
C 3.684201 -3.116207 -0.057850
C 3.109421 -2.100320 -0.857257
C 2.496130 -0.944489 -0.296336
H 1.699248 0.577814 -3.255575
H 2.706507 -1.367058 -4.176448
H 3.801265 -3.531001 -3.893511
H 4.745263 -5.224217 -2.451232
H 4.681468 -4.969475 0.002660
H 3.669081 -3.050516 1.021369
N -2.104534 2.744416 0.911638
C -3.097976 2.614213 2.032465
H -2.582794 2.249571 2.920547
H -3.855828 1.883363 1.754960
H -3.569322 3.587036 2.238853
C -1.122020 3.810184 1.287396
H -0.375370 3.907472 0.498343
H -0.636623 3.530791 2.220935
H -1.630402 4.776896 1.418202
C -2.824929 3.169088 -0.325121
H -3.523657 2.388051 -0.621552
H -2.104802 3.332233 -1.128702
H -3.379051 4.104535 -0.156169
368
Rh -1.131056 0.442437 0.144789
P -0.082660 -1.605991 0.952386
P 1.253994 1.302441 -0.946858
C -1.229089 -2.327661 2.169700
C 0.356420 -2.918446 -0.281383
C 0.902754 2.277357 -2.439359
C 2.324205 2.301019 0.182820
C 1.508176 -1.207666 1.784333
C 2.331633 -0.103909 -1.413785
N -4.160468 -0.554583 -0.425561
C -2.852601 -0.763792 -0.868740
C -2.572973 -0.516459 -2.311406
C -2.562768 -1.460216 -3.282178
C -2.265838 -1.093191 -4.718224
C -2.883388 -2.919438 -3.051632
H -2.142813 0.138463 1.264459
H -2.486680 -1.716366 -0.486094
H -2.397138 0.516196 -2.601100
H -2.902436 -3.194334 -1.993215
H -2.166151 -3.568285 -3.566481
H -3.871901 -3.156237 -3.468946
H -3.055557 -1.456614 -5.387581
H -1.328903 -1.557812 -5.052888
H -2.177644 -0.011241 -4.851458
C -5.195794 0.135206 -1.205469
H -5.444890 1.125327 -0.801484
H -6.110782 -0.466609 -1.206197
H -4.861112 0.247440 -2.236855
C -4.534743 -1.086291 0.883894
H -4.067157 -0.511404 1.698103
H -4.194668 -2.125040 0.986840
H -5.617816 -1.061045 1.004799
C -1.674489 -1.540501 3.236751
C -2.618085 -2.036946 4.144194
C -3.142166 -3.319303 3.962537
C -2.737853 -4.096394 2.873730
C -1.792510 -3.590824 1.972504
H -1.280428 -0.543558 3.363763
H -2.950353 -1.429483 4.981483
H -3.877775 -3.706990 4.658838
H -3.171354 -5.080723 2.719045
H -1.536243 -4.170483 1.103687
C 0.222843 -2.668235 -1.653724
C 0.606508 -3.630011 -2.594116
C 1.131336 -4.852356 -2.175923
C 1.286835 -5.104940 -0.813138
C 0.918409 -4.139588 0.129550
H -0.133146 -1.706533 -1.989063
H 0.508349 -3.414557 -3.649048
H 1.433228 -5.595742 -2.906790
H 1.714296 -6.041800 -0.482406
H 1.071470 -4.335693 1.183012
C 0.193345 1.675607 -3.479785
C -0.144472 2.405626 -4.625873
C 0.207544 3.755659 -4.712450
C 0.885015 4.375660 -3.658097
C 1.223067 3.634298 -2.518846
H -0.080516 0.635541 -3.392959
H -0.680450 1.932265 -5.443022
H -0.056502 4.327698 -5.595034
H 1.135015 5.431110 -3.719728
H 1.699956 4.126739 -1.689796
C 1.968303 2.457697 1.530296
C 2.795843 3.167834 2.406246
C 3.993334 3.719544 1.950647
C 4.367480 3.550436 0.617313
C 3.547446 2.832823 -0.259544
H 1.066670 1.993936 1.905725
H 2.512111 3.274098 3.444301
H 4.637301 4.265114 2.633115
H 5.302688 3.961510 0.261760
H 3.860295 2.686592 -1.285464
C 1.586868 -1.258072 3.190682
C 2.666119 -0.825660 3.864909
C 3.775883 -0.325118 3.179726
C 4.896210 0.139430 3.888154
C 5.979286 0.579822 3.226669
C 6.011149 0.576591 1.825636
C 4.957956 0.138930 1.113217
C 3.788940 -0.322607 1.762263
C 2.652859 -0.791653 1.043013
H 0.777208 -1.662483 3.765821
H 2.675929 -0.872939 4.949936
H 4.897467 0.139802 4.971817
H 6.842545 0.935544 3.779489
H 6.896855 0.934093 1.311916
H 5.020721 0.164243 0.033817
C 2.713495 -0.270736 -2.760267
C 3.434940 -1.326404 -3.173587
C 3.854969 -2.297704 -2.260900
C 4.601962 -3.403966 -2.697664
C 5.033661 -4.319514 -1.814612
C 4.750759 -4.184235 -0.448658
C 4.037790 -3.139257 0.007648
C 3.552551 -2.152419 -0.882785
C 2.791599 -1.033004 -0.439010
H 2.443885 0.465219 -3.495168
H 3.698466 -1.418398 -4.222904
H 4.839538 -3.523561 -3.748124
H 5.612578 -5.170479 -2.158270
H 5.111919 -4.934160 0.246686
H 3.833156 -3.080983 1.067883
N -2.159499 2.467159 0.107161
C -3.343583 2.428933 1.033002
H -2.996958 2.209026 2.042187
H -4.017036 1.639668 0.711640
H -3.864569 3.396376 1.022125
C -1.209118 3.503431 0.630500
H -0.344590 3.566289 -0.030033
H -0.896758 3.227641 1.636285
H -1.699414 4.486002 0.662707
C -2.620659 2.896087 -1.250817
H -3.349934 2.186274 -1.632725
H -1.767924 2.935825 -1.929882
H -3.078993 3.893458 -1.204702
369
Rh 1.307364 1.406098 -0.449717
P 0.819534 -0.772409 -1.243324
P -0.954142 1.803204 0.630370
C 1.983337 -1.022395 -2.611465
C 0.877587 -2.249564 -0.121838
C -0.747868 2.924186 2.043717
C -2.310346 2.445097 -0.452015
C -0.879215 -0.782229 -1.940796
C -1.598861 0.212012 1.276589
N 5.139293 -2.807415 0.699294
C 4.641117 -1.650103 1.153312
C 4.107015 -1.441827 2.433201
C 3.542064 -0.237207 2.851786
C 2.866565 -0.193722 4.197345
C 3.469427 0.969280 2.018080
H 4.650728 -0.833055 0.437438
H 2.746529 1.063311 -1.048301
H 4.100462 -2.268626 3.134417
H 4.331177 1.112583 1.363161
H 3.324873 1.872078 2.616116
H 2.576993 0.944535 1.267711
H 1.774989 -0.126480 4.069792
H 3.167519 0.696010 4.762353
H 3.076737 -1.080883 4.798987
C 5.151433 -4.025101 1.532217
H 5.801276 -3.893339 2.403450
H 5.525966 -4.856891 0.936699
H 4.138245 -4.260745 1.871932
C 5.738278 -2.905994 -0.643645
H 6.813414 -3.097910 -0.569109
H 5.582613 -1.969183 -1.182948
H 5.269902 -3.720061 -1.203541
C 2.102379 -0.012556 -3.569679
C 3.036917 -0.123851 -4.606347
C 3.876623 -1.240145 -4.660900
C 3.794595 -2.231331 -3.678246
C 2.856434 -2.109494 -2.645219
H 1.454217 0.849000 -3.514314
H 3.115718 0.651222 -5.363682
H 4.605176 -1.331164 -5.459192
H 4.469840 -3.082106 -3.707693
H 2.840870 -2.842457 -1.856933
C 1.100599 -2.079326 1.250289
C 1.095407 -3.178126 2.115291
C 0.863282 -4.460844 1.620305
C 0.617493 -4.640085 0.258858
C 0.610511 -3.541312 -0.606642
H 1.237584 -1.085558 1.647835
H 1.274129 -3.027220 3.170728
H 0.862587 -5.312972 2.292184
H 0.420544 -5.630089 -0.129026
H 0.395617 -3.689516 -1.657068
C 0.258731 2.642438 2.966632
C 0.500709 3.504000 4.042592
C -0.261679 4.668041 4.177693
C -1.252626 4.973793 3.239155
C -1.486486 4.102680 2.167038
H 0.839094 1.744099 2.846563
H 1.277128 3.274701 4.767135
H -0.077304 5.341862 5.007197
H -1.828058 5.890204 3.336897
H -2.217181 4.365091 1.423171
C -2.052571 2.725097 -1.801457
C -3.080107 3.159000 -2.645351
C -4.376224 3.314696 -2.153009
C -4.644058 3.031356 -0.813513
C -3.622073 2.586530 0.031285
H -1.056935 2.584361 -2.198751
H -2.868979 3.365241 -3.685762
H -5.173689 3.646670 -2.810318
H -5.649482 3.137651 -0.428984
H -3.846667 2.345131 1.062350
C -1.062471 -0.844030 -3.336828
C -2.275371 -0.723537 -3.903954
C -3.414559 -0.553160 -3.111610
C -4.679546 -0.420763 -3.707288
C -5.777158 -0.296611 -2.942650
C -5.678890 -0.302685 -1.544536
C -4.485023 -0.425748 -0.937823
C -3.298924 -0.547202 -1.698289
C -2.016808 -0.675106 -1.094382
H -0.222850 -1.000745 -3.988773
H -2.369417 -0.767379 -4.985030
H -4.779789 -0.424732 -4.786290
H -6.752006 -0.197897 -3.408856
H -6.578706 -0.205443 -0.946885
H -4.453368 -0.415900 0.143214
C -1.728457 0.025680 2.667851
C -2.086251 -1.155549 3.200251
C -2.359653 -2.250782 2.375679
C -2.718538 -3.487751 2.935604
C -3.002991 -4.532604 2.140590
C -2.950261 -4.404966 0.745854
C -2.610871 -3.236732 0.172665
C -2.295619 -2.109047 0.966023
C -1.925197 -0.857534 0.398105
H -1.545308 0.840153 3.344639
H -2.163585 -1.256922 4.278687
H -2.770859 -3.607164 4.011335
H -3.280137 -5.485498 2.579346
H -3.183891 -5.261914 0.123497
H -2.570579 -3.186137 -0.906792
N 2.049002 3.540551 -0.674022
C 2.120869 3.690745 -2.167515
H 1.128272 3.544689 -2.592740
H 2.789346 2.928431 -2.565451
H 2.490023 4.693133 -2.431977
C 1.165882 4.613449 -0.125193
H 1.175350 4.563729 0.965555
H 0.146384 4.467507 -0.482708
H 1.517894 5.608135 -0.432556
C 3.419841 3.718347 -0.101047
H 4.074839 2.950329 -0.509991
H 3.367618 3.611397 0.983178
H 3.815145 4.715508 -0.345414
370
Rh -1.193129 1.537610 0.784051
P -0.509678 -0.480126 1.805977
P 0.639082 1.549498 -0.989845
C -1.341261 -0.550839 3.417800
C -0.741152 -2.106715 0.940862
C -0.056524 2.210927 -2.530829
C 2.196827 2.464609 -0.589480
C 1.299808 -0.351334 2.094468
C 1.221326 -0.153512 -1.347325
N -4.668497 -3.662486 -2.058312
C -4.591248 -2.454297 -1.485240
C -4.673028 -1.223015 -2.147693
C -4.555652 0.019667 -1.520296
C -4.854299 1.256411 -2.325489
C -4.118979 0.239470 -0.139048
H -2.335823 1.469587 1.879407
H -4.468109 -2.470336 -0.407118
H -4.904859 -1.220627 -3.207482
H -3.030655 0.653002 -0.076055
H -4.107016 -0.642503 0.504620
H -4.690220 1.030356 0.355177
H -4.028953 1.977090 -2.248131
H -5.742035 1.764321 -1.923275
H -5.034660 1.041578 -3.380712
C -4.805956 -3.822341 -3.517598
H -4.642146 -4.868045 -3.777333
H -4.065048 -3.208803 -4.037168
H -5.808874 -3.530289 -3.849313
C -4.676936 -4.893524 -1.246800
H -4.506577 -4.643139 -0.198211
H -3.886604 -5.569922 -1.583837
H -5.642523 -5.402307 -1.336719
C -1.319830 0.585925 4.230644
C -2.006337 0.599778 5.450941
C -2.742982 -0.522371 5.840933
C -2.805076 -1.646690 5.012157
C -2.112786 -1.651120 3.794500
H -0.756486 1.452006 3.917353
H -1.973950 1.476247 6.092336
H -3.279981 -0.516605 6.783310
H -3.400209 -2.506595 5.307775
H -2.204224 -2.500225 3.140160
C -1.326034 -2.155350 -0.333505
C -1.427870 -3.368750 -1.020160
C -0.965395 -4.549100 -0.441278
C -0.380668 -4.512139 0.824095
C -0.255308 -3.298302 1.506851
H -1.671948 -1.246442 -0.804755
H -1.879155 -3.389148 -2.003722
H -1.053379 -5.489005 -0.976527
H -0.003554 -5.421589 1.271565
H 0.227776 -3.277540 2.475280
C -1.231667 1.641845 -3.022907
C -1.820776 2.132162 -4.194516
C -1.243710 3.221401 -4.853871
C -0.087616 3.821127 -4.344571
C 0.499891 3.316282 -3.177476
H -1.671770 0.805017 -2.501016
H -2.722591 1.673393 -4.589994
H -1.699358 3.608600 -5.758588
H 0.345366 4.680209 -4.849484
H 1.364769 3.805786 -2.766540
C 2.341504 3.084844 0.659331
C 3.534453 3.732102 0.997213
C 4.598524 3.761694 0.095375
C 4.469504 3.135317 -1.144584
C 3.282087 2.478018 -1.482253
H 1.535076 3.040218 1.377151
H 3.634713 4.201017 1.966582
H 5.525330 4.260452 0.360923
H 5.295548 3.143150 -1.843002
H 3.202373 1.974036 -2.436977
C 1.783232 -0.107024 3.395130
C 3.074698 0.179968 3.634861
C 3.996651 0.229218 2.584840
C 5.342118 0.544340 2.835795
C 6.233398 0.551893 1.830490
C 5.838200 0.240301 0.522323
C 4.558996 -0.066535 0.243262
C 3.580691 -0.074723 1.263808
C 2.217416 -0.390874 1.008194
H 1.118906 -0.149821 4.238051
H 3.402217 0.372689 4.652330
H 5.671773 0.776937 3.841581
H 7.272010 0.793775 2.031016
H 6.575187 0.248670 -0.273304
H 4.294422 -0.292528 -0.780864

C 1.056333 -0.688952 -2.641324
C 1.402006 -1.952274 -2.941734
C 1.961845 -2.784045 -1.968376
C 2.311314 -4.107347 -2.283236
C 2.871647 -4.898256 -1.353297
C 3.118578 -4.417139 -0.060378
C 2.796876 -3.156300 0.279394
C 2.198738 -2.285425 -0.661965
C 1.836980 -0.946138 -0.339772
H 0.651014 -0.085185 -3.432857
H 1.245288 -2.326303 -3.949077
H 2.133691 -4.498105 -3.278244
H 3.140398 -5.919265 -1.603660
H 3.573264 -5.073281 0.673876
H 2.994389 -2.831566 1.291627
N -1.883205 3.693487 0.680162
C -1.466957 4.254270 2.010393
H -0.383016 4.203262 2.103532
H -1.916418 3.653410 2.799878
H -1.792449 5.301825 2.100443
C -1.287141 4.523972 -0.407945
H -1.637419 4.150567 -1.372641
H -0.199999 4.453192 -0.370178
H -1.583807 5.577810 -0.308797
C -3.373994 3.771819 0.572051
H -3.816741 3.187309 1.377377
H -3.682048 3.354760 -0.387623
H -3.711909 4.816932 0.637999

371

Rh -1.277758 1.167642 1.282902
P -0.348969 -0.919146 1.839562
P 0.442754 1.788364 -0.421188
C -0.987053 -1.285747 3.496088
C -0.631273 -2.391235 0.746558
C -0.351319 2.837190 -1.669044
C 2.000971 2.597313 0.163530
C 1.475504 -0.748781 1.954837
C 0.990340 0.264124 -1.283500
N -4.797249 -1.460846 -0.734797
C -4.280613 -1.097373 -1.902111
C -4.455901 -1.793210 -3.129179
C -3.801820 -1.486096 -4.297934
C -4.100580 -2.263026 -5.548902
C -2.763844 -0.406909 -4.437805
H -2.453935 0.649447 2.235207
H -3.685010 -0.192515 -1.871351
H -5.164300 -2.614765 -3.150918
H -2.352122 -0.063541 -3.486714
H -3.181934 0.464400 -4.961313
H -1.928217 -0.761339 -5.051215
H -3.204603 -2.796695 -5.894538
H -4.384440 -1.583701 -6.364533
H -4.904466 -2.989589 -5.410318
C -5.595775 -2.698190 -0.586385
H -5.049668 -3.552969 -0.993813
H -6.557277 -2.600965 -1.100043
H -5.780852 -2.867781 0.472943
C -4.584611 -0.625301 0.466555
H -5.409604 0.083354 0.590707
H -3.632433 -0.070281 0.410500
H -4.529976 -1.265435 1.348906
C -0.930828 -0.277675 4.461228
C -1.477836 -0.482278 5.733777
C -2.110963 -1.694084 6.025778
C -2.209246 -2.690549 5.049579
C -1.656981 -2.476200 3.780240
H -0.448208 0.658185 4.222416
H -1.417367 0.294138 6.491494
H -2.539653 -1.857642 7.008594
H -2.724804 -3.620662 5.273325
H -1.778144 -3.226610 3.018829
C -1.372677 -2.253880 -0.434606
C -1.551139 -3.343692 -1.292003
C -0.988390 -4.581575 -0.984002
C -0.235739 -4.725517 0.181903
C -0.046654 -3.635512 1.037717
H -1.779842 -1.289317 -0.698712
H -2.129069 -3.220017 -2.197861
H -1.127851 -5.425069 -1.652436
H 0.216720 -5.678828 0.418568
H 0.558009 -3.752406 1.927865
C -1.625442 2.474511 -2.104539
C -2.305720 3.257946 -3.043556
C -1.708190 4.425475 -3.528259
C -0.444723 4.813551 -3.069384
C 0.228041 4.019496 -2.131133
H -2.080908 1.584392 -1.702986
H -3.293868 2.967246 -3.389841
H -2.231442 5.039379 -4.253090
H 0.005780 5.732693 -3.433632
H 1.182074 4.339713 -1.753944
C 2.148789 2.919573 1.520372
C 3.342625 3.475546 1.991620
C 4.402310 3.712632 1.115562
C 4.266852 3.389013 -0.234670
C 3.079134 2.822577 -0.708537
H 1.339454 2.723174 2.211621
H 3.445238 3.715449 3.041143
H 5.329765 4.139914 1.483617
H 5.089609 3.557864 -0.916231
H 2.996023 2.548652 -1.752503
C 2.097494 -0.754683 3.218993
C 3.409359 -0.502262 3.369982
C 4.213671 -0.240311 2.256479
C 5.582615 0.028904 2.417925
C 6.360710 0.243343 1.343917
C 5.822666 0.197144 0.050523
C 4.516203 -0.055544 -0.144105
C 3.653309 -0.276541 0.954165
C 2.265517 -0.543820 0.790433
H 1.526114 -0.969034 4.103732
H 3.846242 -0.507560 4.364231
H 6.021634 0.058937 3.408212
H 7.418283 0.447647 1.475165
H 6.470349 0.369072 -0.802282
H 4.140358 -0.072709 -1.158121
C 0.637295 0.065545 -2.633950
C 0.915376 -1.077244 -3.284193
C 1.585059 -2.117614 -2.633978
C 1.862119 -3.315396 -3.312808
C 2.524605 -4.306085 -2.692998
C 2.950677 -4.160241 -1.365771
C 2.702550 -3.028959 -0.682190
C 2.003196 -1.958987 -1.287950
C 1.713638 -0.748942 -0.596276
H 0.125865 0.836614 -3.181884
H 0.614067 -1.191668 -4.320934
H 1.545630 -3.447604 -4.340695
H 2.736380 -5.228599 -3.223492
H 3.484414 -4.973166 -0.885606
H 3.038291 -2.964315 0.343644
N -2.230883 3.182583 1.694423
C -2.037827 3.318889 3.178510
H -0.969620 3.304282 3.398469
H -2.506410 2.470960 3.675618
H -2.477224 4.262669 3.536438
C -1.624040 4.375741 1.030950
H -1.867387 4.363654 -0.032751
H -0.541302 4.350560 1.153468
H -2.017373 5.307032 1.462809
C -3.691999 3.148960 1.378046
H -4.143592 2.308764 1.903638
H -3.813671 3.011230 0.301134
H -4.181576 4.086932 1.682518

372

Rh -1.255470 0.877953 1.286770
P -0.221330 -1.147898 1.907004
P 0.379798 1.468809 -0.517460
C -0.806968 -1.484922 3.589126
C -0.440005 -2.669507 0.871208
C -0.517635 2.388504 -1.797103
C 1.907074 2.401857 -0.046552
C 1.593012 -0.873810 1.976780
C 0.993648 -0.071175 -1.302040
N -5.295247 -0.215509 -0.866360
C -5.084959 -0.208657 -2.178818
C -3.985610 -0.820205 -2.835907
C -3.729730 -0.756691 -4.183730
C -2.496028 -1.418276 -4.733513
C -4.587514 -0.048654 -5.195887
H -2.375754 0.373335 2.309594
H -5.834999 0.314951 -2.760246
H -3.268835 -1.348437 -2.221175
H -3.995148 0.710206 -5.724598
H -5.467570 0.443584 -4.778840
H -4.929897 -0.757672 -5.961114
H -1.844485 -0.670676 -5.208348
H -2.753931 -2.144814 -5.514754
H -1.921303 -1.927889 -3.957168
C -4.365822 -0.817092 0.103824
H -4.860918 -0.881920 1.072275
H -3.443213 -0.202246 0.245666
H -4.082900 -1.822309 -0.211083
C -6.470528 0.474663 -0.291566
H -6.134006 1.234947 0.418634
H -7.100068 -0.248326 0.234310
H -7.049744 0.951811 -1.082360
C -0.790551 -0.438630 4.514355
C -1.297504 -0.625216 5.806081
C -1.850259 -1.860000 6.158440
C -1.908478 -2.898152 5.223379
C -1.396989 -2.701376 3.934159
H -0.368824 0.513714 4.229919
H -1.267848 0.182517 6.532272
H -2.247748 -2.009898 7.156446
H -2.361929 -3.847784 5.494596
H -1.487611 -3.487555 3.205765
C -1.194809 -2.606338 -0.306677
C -1.338220 -3.733401 -1.121924
C -0.714201 -4.932708 -0.778150
C 0.058966 -5.000134 0.381393
C 0.206872 -3.873593 1.197136
H -1.640911 -1.668399 -0.599783
H -1.926973 -3.670382 -2.026998
H -0.820399 -5.805267 -1.414981
H 0.559625 -5.922259 0.644100
H 0.826844 -3.930920 2.082615
C -1.774547 1.917158 -2.174434
C -2.540391 2.610953 -3.117518
C -2.043320 3.793108 -3.673993
C -0.795096 4.286860 -3.279710
C -0.038415 3.585741 -2.331248
H -2.155860 1.013256 -1.725713
H -3.519417 2.239517 -3.407744
H -2.634290 4.336684 -4.402641
H -0.423397 5.217766 -3.698581
H 0.899763 3.991014 -1.999019
C 2.091688 2.807374 1.283105
C 3.266833 3.460310 1.669272
C 4.271487 3.712127 0.734479
C 4.100258 3.305228 -0.588918
C 2.931744 2.642021 -0.977269
H 1.327286 2.599528 2.020193
H 3.398317 3.764009 2.698851
H 5.184603 4.215226 1.036636
H 4.880975 3.485862 -1.315426
H 2.822033 2.305740 -2.000353
C 2.235562 -0.780979 3.227214
C 3.532053 -0.443757 3.340257
C 4.300049 -0.187696 2.200180
C 5.652143 0.172247 2.322012
C 6.397458 0.381983 1.224115
C 5.841879 0.240697 -0.054860
C 4.550694 -0.100993 -0.211765
C 3.721561 -0.321463 0.912282
C 2.350634 -0.681675 0.788356
H 1.693380 -0.984789 4.132420
H 3.984904 -0.374134 4.324929
H 6.104799 0.277102 3.301010
H 7.442241 0.657134 1.324873
H 6.463136 0.411416 -0.927331
H 4.159778 -0.189421 -1.216310
C 0.631860 -0.365619 -2.632645
C 0.983041 -1.517408 -3.229735
C 1.732246 -2.474066 -2.539099
C 2.082067 -3.683774 -3.160736
C 2.815762 -4.595408 -2.501078
C 3.244228 -4.354380 -1.188571
C 2.928524 -3.208396 -0.559547
C 2.153942 -2.218909 -1.209082
C 1.793279 -0.995993 -0.575915
H 0.056657 0.337751 -3.208470
H 0.679319 -1.704313 -4.255439
H 1.765038 -3.888808 -4.176518
H 3.084068 -5.527416 -2.987696
H 3.836338 -5.104499 -0.675606
H 3.270182 -3.069243 0.456938
N -2.234118 2.890830 1.652398
C -1.940374 3.116354 3.108593
H -0.859690 3.142859 3.251385
H -2.346920 2.285363 3.683043
H -2.381902 4.066909 3.445885
C -1.713255 4.056144 0.876721
H -2.015022 3.960291 -0.168096
H -0.625520 4.077481 0.935714
H -2.116554 5.001009 1.268488
C -3.711546 2.804902 1.448313
H -4.095436 1.978133 2.044947
H -3.903325 2.613476 0.389462
H -4.205201 3.744039 1.744052

373

Rh -1.320478 1.020351 0.909726
P -0.329979 -0.989380 1.629183
P 0.512353 1.636155 -0.679532
C -1.081303 -1.339128 3.242918
C -0.432787 -2.515784 0.578040
C -0.201101 2.605981 -2.037019
C 1.970796 2.544057 0.009684
C 1.465716 -0.709662 1.895967
C 1.216849 0.109173 -1.414695
N -4.633749 -1.612698 -0.268360
C -4.164987 -0.487552 -0.782714
C -4.445903 0.068780 -2.086393
C -4.454211 -0.513848 -3.318236
C -4.791876 0.304977 -4.538297
C -4.042176 -1.936051 -3.597990
H -2.547241 0.511439 1.817383
H -3.503058 0.092284 -0.117925
H -4.553991 1.152933 -2.050177
H -3.685739 -2.457378 -2.711202
H -3.235089 -1.938501 -4.341128
H -4.869078 -2.509524 -4.037163
H -5.585881 -0.185371 -5.116358
H -3.924681 0.382675 -5.207230
H -5.129000 1.312092 -4.282296
C -5.634334 -2.490287 -0.910370
H -6.397379 -2.732630 -0.166535
H -5.171218 -3.419642 -1.252747
H -6.096774 -1.975263 -1.750133
C -4.242656 -2.019333 1.102707
H -5.125126 -2.028310 1.748380
H -3.512359 -1.306436 1.494208
H -3.811564 -3.023982 1.074467
C -1.189494 -0.290016 4.158510
C -1.831903 -0.483463 5.387377
C -2.392428 -1.729282 5.684105
C -2.321378 -2.772614 4.755872
C -1.673696 -2.569333 3.530761
H -0.760194 0.670453 3.916385
H -1.901526 0.327551 6.107111
H -2.895613 -1.884247 6.632378
H -2.780343 -3.731536 4.981043
H -1.665087 -3.360568 2.802505
C -1.088725 -2.476498 -0.661986
C -1.102846 -3.603764 -1.490881
C -0.475373 -4.782576 -1.089669
C 0.179541 -4.830680 0.140371
C 0.215199 -3.701535 0.963925
H -1.557560 -1.560728 -0.995243
H -1.592060 -3.555778 -2.452907
H -0.486676 -5.654010 -1.736517
H 0.682033 -5.736950 0.449992
H 0.753482 -3.741284 1.902210
C -1.382492 2.147835 -2.621447
C -2.006344 2.887363 -3.632956
C -1.450050 4.101972 -4.044262
C -0.283103 4.581819 -3.441752
C 0.333638 3.834546 -2.430239
H -1.807543 1.213207 -2.287031
H -2.919717 2.526886 -4.093808
H -1.931548 4.680014 -4.825276
H 0.133502 5.536669 -3.750096
H 1.204685 4.229410 -1.939997
C 1.985273 2.912537 1.362594
C 3.102689 3.548794 1.912974
C 4.218478 3.820948 1.120805
C 4.216094 3.451744 -0.224511
C 3.105543 2.805177 -0.776536
H 1.133284 2.688134 1.990369
H 3.102787 3.823141 2.959043
H 5.086749 4.310515 1.550464
H 5.083196 3.648045 -0.840590
H 3.125392 2.499168 -1.814697
C 1.967958 -0.613626 3.209228
C 3.246448 -0.284350 3.462731
C 4.136849 -0.039141 2.413109
C 5.469945 0.312133 2.681514
C 6.332234 0.514495 1.671418
C 5.918646 0.374880 0.339572
C 4.649978 0.041647 0.042934
C 3.701711 -0.172571 1.070023
C 2.349794 -0.523975 0.797730
H 1.328977 -0.808317 4.050865
H 3.588761 -0.212821 4.490912
H 5.813454 0.416435 3.703975
H 7.361612 0.782846 1.885414
H 6.632582 0.540094 -0.460015
H 4.370472 -0.044868 -0.998269
C 1.002404 -0.168223 -2.780143
C 1.412232 -1.314661 -3.349604
C 2.085957 -2.279360 -2.595254
C 2.503458 -3.480592 -3.191307
C 3.170047 -4.397264 -2.470257
C 3.460607 -4.170024 -1.118173
C 3.075916 -3.032918 -0.511873
C 2.366776 -2.038753 -1.226079
C 1.938264 -0.823923 -0.619933
H 0.496744 0.543848 -3.406582
H 1.216005 -1.490275 -4.403048
H 2.293882 -3.674491 -4.236637
H 3.491393 -5.322440 -2.937315
H 4.000555 -4.923886 -0.555602
H 3.310167 -2.904862 0.535960
N -2.340091 3.034774 1.190079
C -2.192676 3.249944 2.670112
H -1.132562 3.266330 2.922799
H -2.662638 2.420130 3.195823
H -2.659789 4.201914 2.965877
C -1.738591 4.202677 0.478420
H -1.938862 4.115964 -0.591196
H -0.661393 4.216293 0.642460
H -2.172054 5.147292 0.836488
C -3.793389 2.967508 0.843786
H -4.246288 2.138345 1.385994
H -3.890521 2.800297 -0.231905
H -4.304599 3.906364 1.106893

376

Rh -1.146625 1.292380 0.710652
P -0.516751 -0.939632 1.069850
P 0.909945 1.925341 -0.607019
C -1.499302 -1.509878 2.483900
C -0.679737 -2.165549 -0.314975
C 0.435925 3.182894 -1.830867
C 2.390944 2.520682 0.331336
C 1.251436 -0.981539 1.561125
C 1.539115 0.472811 -1.539539
N -4.510297 -0.398305 -1.606931
C -4.300330 -0.793723 -0.365109
C -4.817186 -1.950179 0.324317
C -4.911205 -3.258095 -0.050878
C -5.455967 -4.273470 0.921919
C -4.410431 -3.828381 -1.352237
H -2.517179 0.803882 1.386892
H -3.668783 -0.119418 0.231027
H -5.023868 -1.725276 1.371167
H -3.893685 -3.098032 -1.972759
H -5.232560 -4.266222 -1.933174
H -3.710062 -4.646553 -1.143631
H -4.688786 -5.016272 1.175401
H -6.288831 -4.826947 0.469451
H -5.810790 -3.810466 1.845531
C -3.860716 0.845422 -2.089246
H -3.056048 1.121641 -1.392072
H -4.601299 1.648925 -2.143667
H -3.445836 0.670608 -3.084805
C -5.445878 -1.035535 -2.555507
H -6.094101 -1.730004 -2.023720
H -4.899126 -1.563611 -3.341091
H -6.051642 -0.250509 -3.014907
C -1.603652 -0.668817 3.594340
C -2.401182 -1.031047 4.686750
C -3.115947 -2.231959 4.651083

C -3.043728 -3.061050 3.527724
C -2.242219 -2.689803 2.440920
H -1.052621 0.259368 3.609461
H -2.470909 -0.384518 5.557128
H -3.739047 -2.516176 5.492020
H -3.621003 -3.980697 3.493708
H -2.235849 -3.303783 1.558151
C -1.162478 -1.747681 -1.563502
C -1.217664 -2.639467 -2.639189
C -0.805477 -3.962015 -2.478141
C -0.322780 -4.387932 -1.240578
C -0.246092 -3.493673 -0.167819
H -1.463998 -0.720225 -1.705648
H -1.590493 -2.299743 -3.595533
H -0.854350 -4.654047 -3.312625
H 0.012782 -5.408056 -1.113490
H 0.159698 -3.826921 0.778662
C -0.643604 2.913173 -2.673285
C -1.079420 3.868290 -3.599175
C -0.438329 5.109021 -3.663112
C 0.626410 5.396996 -2.803186
C 1.055997 4.432582 -1.882412
H -1.130897 1.953424 -2.610872
H -1.913181 3.652251 -4.261412
H -0.774015 5.854852 -4.375262
H 1.108428 6.369882 -2.843618
H 1.846003 4.673590 -1.193214
C 2.358580 2.565403 1.732688
C 3.489910 2.955215 2.456864
C 4.667833 3.296546 1.791779
C 4.714496 3.240673 0.398549
C 3.588767 2.841969 -0.329257
H 1.462586 2.267291 2.260411
H 3.453369 2.978249 3.537456
H 5.546660 3.593074 2.355620
H 5.629304 3.489714 -0.122208
H 3.644601 2.776853 -1.408344
C 1.593798 -1.216831 2.907680
C 2.857692 -1.106709 3.352826
C 3.890314 -0.760563 2.475942
C 5.207263 -0.630732 2.946376
C 6.201793 -0.325753 2.096135
C 5.943282 -0.137481 0.731467
C 4.695435 -0.253450 0.243732
C 3.613255 -0.564064 1.099152
C 2.278700 -0.691420 0.622733
H 0.837842 -1.495098 3.619148
H 3.075428 -1.288064 4.401242
H 5.430971 -0.780252 3.996095
H 7.217532 -0.228903 2.465316
H 6.761687 0.106397 0.062883
H 4.538215 -0.089721 -0.813749
C 1.525786 0.485928 -2.949591
C 1.882305 -0.585786 -3.678228
C 2.305429 -1.760473 -3.050348
C 2.666545 -2.882255 -3.814353
C 3.103496 -3.999611 -3.210049
C 3.209864 -4.062431 -1.813967
C 2.872543 -3.009247 -1.048688
C 2.398606 -1.812843 -1.636437
C 2.026098 -0.678287 -0.861494
H 1.235083 1.371940 -3.483168
H 1.845050 -0.534324 -4.762282
H 2.599341 -2.852735 -4.895485
H 3.382675 -4.862190 -3.806199
H 3.567391 -4.974090 -1.347684
H 2.959000 -3.105420 0.024945
N -1.941049 3.367301 1.210567
C -2.751876 3.221104 2.467681
H -2.137673 2.742095 3.229678
H -3.608326 2.579532 2.268199
H -3.091914 4.207496 2.818698
C -0.836110 4.336301 1.492324
H -0.217152 4.451527 0.603069
H -0.228892 3.955863 2.311683
H -1.244178 5.319633 1.769371
C -2.811584 3.918846 0.130887
H -3.600084 3.199615 -0.089068
H -2.212627 4.084367 -0.766156
H -3.262195 4.875696 0.436297

384

Rh -1.223075 1.005259 0.778368
P -0.406073 -1.185393 1.033784
P 0.805382 1.860450 -0.507158
C -1.357135 -1.948304 2.377847
C -0.434003 -2.310793 -0.439441
C 0.233251 3.101688 -1.702837
C 2.205709 2.566650 0.475136
C 1.348631 -1.094491 1.568943
C 1.586132 0.503173 -1.468145
N -4.190803 -0.574984 -2.192055
C -4.088911 -0.439918 -0.873650
C -4.558618 -1.337802 0.143293
C -5.739005 -2.021850 0.241095
C -6.023171 -2.837043 1.476352
C -6.868731 -1.956641 -0.756389
H -2.507738 0.390060 1.478905
H -3.486625 0.408210 -0.511195
H -3.917313 -1.318362 1.021837
H -6.774576 -1.122794 -1.454617
H -7.820824 -1.843464 -0.225584
H -6.947324 -2.888544 -1.333544
H -6.250346 -3.877211 1.208243
H -6.904390 -2.450337 2.004146
H -5.179576 -2.834348 2.170482
C -3.793694 0.532970 -3.086094
H -2.925452 0.240314 -3.683930
H -3.550284 1.410203 -2.487051
H -4.623597 0.776483 -3.755070
C -4.555175 -1.831624 -2.878580
H -4.505943 -2.658122 -2.170811
H -3.842083 -2.000380 -3.689002
H -5.559769 -1.771505 -3.304695
C -1.579107 -1.201211 3.537258
C -2.351488 -1.723807 4.581734
C -2.920224 -2.994025 4.450742
C -2.730372 -3.732935 3.279196
C -1.961306 -3.198441 2.237986
H -1.136928 -0.220555 3.628691
H -2.512357 -1.149058 5.489649
H -3.521683 -3.403346 5.255126
H -3.196460 -4.708633 3.171052
H -1.865353 -3.749084 1.319546
C -0.961680 -1.853858 -1.655939
C -0.927338 -2.665990 -2.793973
C -0.374621 -3.944812 -2.729971
C 0.155711 -4.407208 -1.525539
C 0.138075 -3.592963 -0.388546
H -1.368893 -0.854535 -1.723352
H -1.329181 -2.295092 -3.726235
H -0.348796 -4.572996 -3.614575
H 0.600806 -5.391462 -1.473448
H 0.578668 -3.951047 0.533041
C -0.790215 2.745672 -2.580583
C -1.303696 3.677073 -3.490537
C -0.797826 4.980536 -3.500860
C 0.208730 5.352979 -2.603552
C 0.716958 4.410893 -1.699602
H -1.171697 1.737505 -2.553064
H -2.093774 3.394210 -4.180640
H -1.194316 5.708615 -4.199979
H 0.584357 6.372461 -2.602376
H 1.460225 4.711201 -0.982027
C 2.149152 2.561238 1.876376
C 3.225343 3.037615 2.632131
C 4.372350 3.516789 1.998779
C 4.444084 3.512266 0.605461
C 3.374285 3.027903 -0.154089
H 1.280050 2.158156 2.378586
H 3.171359 3.020978 3.712106
H 5.208820 3.880830 2.586973
H 5.336313 3.869137 0.108810
H 3.451191 3.005439 -1.233612
C 1.676877 -1.354275 2.914690
C 2.911258 -1.141670 3.403231
C 3.927614 -0.657070 2.574597
C 5.211606 -0.418805 3.091457
C 6.193547 0.020168 2.286437
C 5.954971 0.241908 0.923191
C 4.738847 0.024696 0.391871
C 3.669997 -0.428416 1.199145
C 2.368327 -0.665871 0.675831
H 0.933462 -1.733794 3.591218
H 3.117237 -1.346574 4.449704
H 5.420290 -0.591607 4.140704
H 7.183960 0.200649 2.691287
H 6.762768 0.594912 0.291449
H 4.595047 0.218607 -0.662412
C 1.613107 0.567140 -2.876766
C 2.091179 -0.438418 -3.629567
C 2.608044 -1.589305 -3.028641
C 3.099717 -2.641563 -3.818288
C 3.628229 -3.732079 -3.238608
C 3.701398 -3.835267 -1.842804
C 3.239800 -2.849154 -1.053416
C 2.665615 -1.684325 -1.615080
C 2.160389 -0.621113 -0.814327
H 1.256913 1.441668 -3.389139
H 2.080871 -0.350472 -4.711830
H 3.060749 -2.578849 -4.899391
H 4.008509 -4.540462 -3.854490
H 4.135153 -4.723623 -1.396570
H 3.305938 -2.975650 0.018517
N -2.209407 2.988286 1.297272
C -3.085411 2.747467 2.495121
H -2.488811 2.287505 3.282309
H -3.882146 2.057189 2.222678
H -3.514489 3.696974 2.849483
C -1.181517 4.007706 1.680261
H -0.518113 4.190126 0.834934
H -0.603763 3.629605 2.522006
H -1.661860 4.955107 1.966090
C -3.047272 3.533234 0.188115
H -3.776860 2.780529 -0.113482
H -2.408076 3.779766 -0.660836
H -3.577194 4.444481 0.504640

385

Rh -1.275563 1.061812 0.642949
P -0.526934 -1.133715 1.032616
P 0.872964 1.846545 -0.444143
C -1.612555 -1.840302 2.303629
C -0.437907 -2.304885 -0.402331
C 0.460140 3.080523 -1.707672
C 2.172658 2.540355 0.674410
C 1.172345 -1.055200 1.728172
C 1.703515 0.453551 -1.305014
N -3.978604 -0.551417 -2.572932
C -3.991949 -0.376914 -1.255193
C -4.563012 -1.239831 -0.259542
C -5.756913 -1.907331 -0.250011
C -6.160951 -2.683357 0.977069
C -6.791246 -1.858928 -1.346761
H -2.640063 0.496486 1.244013
H -3.416426 0.476946 -0.865936
H -4.003143 -1.202762 0.672341
H -7.784620 -1.711015 -0.908114
H -6.836497 -2.809575 -1.896311
H -6.618285 -1.052573 -2.061792
H -6.364303 -3.731595 0.721245
H -7.087214 -2.280643 1.406502
H -5.386836 -2.658837 1.747509
C -3.492287 0.523835 -3.463133
H -2.551796 0.225041 -3.936121
H -3.341571 1.433454 -2.882661
H -4.238138 0.716822 -4.238832
C -4.292477 -1.825989 -3.251301
H -4.313424 -2.631274 -2.518206
H -3.512427 -2.023566 -3.990434
H -5.255022 -1.772139 -3.766092
C -1.927164 -1.054868 3.415320
C -2.801925 -1.533455 4.398320
C -3.378773 -2.798339 4.252049
C -3.094690 -3.575607 3.125225
C -2.223583 -3.084786 2.144629
H -1.476062 -0.079663 3.519854
H -3.035077 -0.929151 5.270670
H -4.058960 -3.173866 5.008801
H -3.565982 -4.547120 3.002815
H -2.052818 -3.664816 1.255579
C -0.843652 -1.880048 -1.675944
C -0.719122 -2.729185 -2.780177
C -0.196552 -4.012952 -2.625939
C 0.213482 -4.443398 -1.364086
C 0.105695 -3.592510 -0.259379
H -1.225004 -0.877471 -1.811876
H -1.026706 -2.382727 -3.756641
H -0.100297 -4.669679 -3.484562
H 0.635277 -5.431556 -1.240785
H 0.453834 -3.926712 0.709523
C -0.550460 2.769066 -2.615923
C -0.952974 3.700894 -3.579551
C -0.346717 4.960467 -3.614932
C 0.648623 5.289902 -2.688671
C 1.043919 4.348358 -1.729316
H -1.007678 1.793941 -2.569459
H -1.733243 3.452073 -4.293476
H -0.656273 5.688572 -4.356612
H 1.102159 6.277002 -2.707807
H 1.777128 4.620309 -0.991369
C 1.927696 2.634511 2.051776
C 2.920216 3.103410 2.918595
C 4.168243 3.479777 2.421178
C 4.423627 3.382708 1.053028
C 3.437982 2.903989 0.183879
H 0.970757 2.325289 2.450152
H 2.720178 3.165795 3.979571
H 4.938913 3.839176 3.095784
H 5.393200 3.661465 0.662879
H 3.655716 2.808574 -0.872193
C 1.367753 -1.278125 3.106075
C 2.556405 -1.077972 3.701345
C 3.658353 -0.647335 2.956785
C 4.895756 -0.427146 3.583641
C 5.961555 -0.045540 2.860268
C 5.857713 0.133357 1.474062
C 4.689498 -0.066882 0.838697
C 3.535991 -0.458661 1.556829
C 2.281335 -0.677696 0.922028
H 0.555895 -1.618610 3.721649
H 2.658861 -1.253042 4.768278
H 5.000837 -0.568886 4.652844
H 6.915476 0.120550 3.350020
H 6.731840 0.438819 0.909382
H 4.650848 0.092201 -0.230382
C 1.823929 0.474714 -2.709913
C 2.345994 -0.554692 -3.398498
C 2.809027 -1.691610 -2.730572
C 3.346199 -2.768979 -3.453934
C 3.815393 -3.849739 -2.808681
C 3.779588 -3.918369 -1.409312
C 3.271523 -2.907550 -0.682181
C 2.758717 -1.750388 -1.314610
C 2.208015 -0.661256 -0.581404
H 1.499921 1.332028 -3.271156
H 2.409465 -0.498951 -4.481066
H 3.389505 -2.734102 -4.536123
H 4.231114 -4.677746 -3.373462
H 4.165793 -4.800066 -0.909410
H 3.252919 -3.008018 0.394336
N -2.274163 3.051054 1.092026
C -2.368796 3.030803 2.591595
H -1.364825 2.968747 3.011529
H -2.929990 2.149236 2.897920
H -2.865532 3.943345 2.955191
C -1.541769 4.284566 0.676264
H -1.559037 4.362352 -0.412536
H -0.507770 4.229239 1.016647
H -2.013676 5.183914 1.096521
C -3.651696 3.101444 0.510639
H -4.206060 2.222770 0.838182
H -3.573215 3.100261 -0.579496
H -4.178963 4.013563 0.829842

No.: 400

Rh -1.225668 1.196780 0.434141
P -0.441805 -0.891856 1.224886
P 0.802864 1.703342 -1.000892
C -1.397756 -1.219143 2.731882
C -0.477189 -2.401849 0.148551
C 0.262311 2.668203 -2.439875
C 2.225410 2.567246 -0.192534
C 1.321078 -0.695881 1.701648
C 1.522481 0.138281 -1.630032
N -5.558579 -1.781056 0.479099
C -4.687052 -2.431741 -0.300526
C -4.215907 -2.146373 -1.602672
C -3.893334 -0.949750 -2.236670
C -3.408815 -1.029522 -3.666126
C -3.765029 0.364557 -1.587047
H -2.524842 0.785985 1.263798
H -4.346272 -3.370775 0.132465
H -3.940288 -3.050702 -2.141894
H -2.748239 0.463035 -1.032849
H -4.470633 0.565980 -0.786051
H -3.788272 1.185501 -2.308783
H -2.359627 -0.711570 -3.744078
H -3.985326 -0.343765 -4.300059
H -3.494577 -2.035743 -4.081259
C -5.726739 -2.172803 1.892337
H -5.279500 -1.409943 2.541572
H -5.224957 -3.123740 2.075577
H -6.788442 -2.268645 2.130346
C -6.418364 -0.679991 0.024742
H -6.415179 -0.633739 -1.063149
H -6.081513 0.278335 0.435926
H -7.436004 -0.868312 0.377805
C -1.525226 -0.192100 3.670712
C -2.306827 -0.371867 4.818446
C -2.985618 -1.578980 5.008715
C -2.897264 -2.594628 4.052026
C -2.115345 -2.403201 2.905793
H -1.003216 0.739198 3.511098
H -2.392118 0.420127 5.557280
H -3.596333 -1.723035 5.893302
H -3.452111 -3.518737 4.188833
H -2.103578 -3.162117 2.143652
C -0.905363 -2.304508 -1.181468
C -0.883549 -3.423218 -2.020604
C -0.429205 -4.651868 -1.542268
C 0.016977 -4.755997 -0.224702
C 0.007518 -3.636293 0.613411
H -1.217466 -1.348122 -1.572024
H -1.213636 -3.328964 -3.045800
H -0.410510 -5.518690 -2.195079
H 0.387784 -5.701453 0.147259
H 0.380720 -3.722804 1.625780
C -0.840704 2.210371 -3.159850
C -1.336861 2.942516 -4.244458
C -0.730801 4.153213 -4.593206
C 0.357188 4.634072 -3.857289
C 0.845538 3.891896 -2.773942
H -1.299774 1.279076 -2.874855
H -2.189436 2.577720 -4.810523
H -1.112354 4.727320 -5.430414
H 0.810995 5.585890 -4.119161
H 1.651682 4.287772 -2.182880
C 2.145037 2.928671 1.159796
C 3.232862 3.531718 1.799491
C 4.414047 3.776229 1.098435
C 4.507179 3.411226 -0.244856
C 3.425834 2.797817 -0.885501
H 1.243528 2.720294 1.718919
H 3.159381 3.800063 2.844566
H 5.259045 4.239946 1.597585
H 5.424802 3.586052 -0.790246
H 3.518587 2.495055 -1.920658
C 1.674798 -0.646851 3.064821
C 2.924767 -0.360647 3.468574
C 3.934178 -0.118232 2.532131
C 5.236922 0.189702 2.957097
C 6.213757 0.384411 2.055576
C 5.951232 0.278390 0.682897
C 4.716297 -0.013580 0.237689
C 3.651484 -0.215517 1.145965
C 2.330696 -0.522530 0.714684
H 0.941254 -0.846930 3.823841
H 3.150783 -0.323384 4.530209
H 5.464534 0.265871 4.013854
H 7.218864 0.619239 2.390293
H 6.755869 0.435848 -0.027061
H 4.555119 -0.076259 -0.829937
C 1.488928 -0.137587 -3.012090
C 1.918324 -1.306521 -3.517843
C 2.432985 -2.296591 -2.675800
C 2.868996 -3.521757 -3.206201
C 3.382604 -4.463878 -2.397986
C 3.494108 -4.239555 -1.019031
C 3.087129 -3.079465 -0.473970
C 2.534012 -2.058068 -1.281329
C 2.086178 -0.818915 -0.742233
H 1.117783 0.596734 -3.703944
H 1.865763 -1.479690 -4.588588
H 2.796986 -3.714262 -4.270148

H 3.718933 -5.407821 -2.814217
H 3.912394 -5.014304 -0.385643
H 3.180530 -2.953805 0.596011
N -2.103532 3.282365 0.647243
C -1.985618 3.526253 2.125019
H -0.936100 3.482473 2.414639
H -2.527786 2.744203 2.654824
H -2.397216 4.514018 2.382419
C -1.399966 4.382352 -0.079210
H -1.562419 4.265247 -1.152558
H -0.331613 4.335447 0.130451
H -1.785554 5.365146 0.226414
C -3.547797 3.312650 0.260221
H -4.072905 2.531487 0.808347
H -3.631349 3.123564 -0.811219
H -3.991870 4.293057 0.489726
411
Rh -1.280696 1.106889 0.918191
P -0.445415 -1.039077 1.585528
P 0.605380 1.531301 -0.822664
C -1.283316 -1.416640 3.147891
C -0.602642 -2.479329 0.432232
C -0.093224 2.427435 -2.235894
C 2.083939 2.444446 -0.194147
C 1.348294 -0.842729 1.913379
C 1.281688 -0.052381 -1.448637
N -4.469754 -1.764405 0.287835
C -4.449421 -0.823871 -0.713414
C -4.245307 -1.152366 -2.092863
C -4.464282 -0.313999 -3.144026
C -4.179603 -0.752389 -4.559201
C -5.000967 1.087321 -2.950369
H -2.397749 0.783861 1.958136
H -4.827875 0.143994 -0.420477
H -3.884310 -2.150920 -2.321805
H -4.279926 1.731167 -2.417849
H -5.924028 1.090668 -2.355369
H -5.223974 1.569839 -3.905739
H -3.399041 -0.133638 -5.026310
H -5.071909 -0.655068 -5.193151
H -3.846775 -1.794019 -4.602110
C -4.214053 -3.080753 0.195571
H -4.275939 -3.683625 1.085943
H -2.389127 0.445145 0.050618
H -4.020908 -3.533043 -0.761668
C -4.817169 -1.240273 1.641133
H -4.034646 -0.550516 1.967972
H -4.896959 -2.066541 2.344687
H -5.772463 -0.716608 1.586946
C -1.335872 -0.427129 4.133847
C -2.040889 -0.647071 5.323602
C -2.721510 -1.853889 5.509374
C -2.708281 -2.830654 4.509277
C -1.996558 -2.602099 3.325207
H -0.817607 0.506806 3.977385
H -2.066921 0.114004 6.098534
H -3.274538 -2.028348 6.425890
H -3.264047 -3.754622 4.644151
H -2.040802 -3.330786 2.534717
C -1.154005 -2.312747 -0.847264
C -1.194576 -3.381663 -1.749344
C -0.699979 -4.632186 -1.381656
C -0.145867 -4.808342 -0.114367
C -0.084973 -3.737849 0.783330
H -1.515658 -1.344221 -1.157593
H -1.611292 -3.233296 -2.735498
H -0.735804 -5.460679 -2.081616
H 0.254805 -5.771285 0.171221
H 0.375614 -3.879863 1.752519
C -1.232596 1.911644 -2.856680
C -1.819062 2.588319 -3.933837
C -1.276085 3.800915 -4.367471
C -0.156438 4.339876 -3.727462
C 0.428481 3.652471 -2.656868
H -1.648493 0.978049 -2.507753
H -2.692798 2.181118 -4.432096
H -1.730251 4.330407 -5.197869
H 0.250420 5.292255 -4.056093
H 1.265644 4.090486 -2.143434
C 2.127476 2.864867 1.142685
C 3.261360 3.509887 1.647193
C 4.364722 3.739290 0.824562
C 4.333659 3.317685 -0.505047
C 3.206504 2.662117 -1.010759
H 1.286392 2.671887 1.794574
H 3.284823 3.823409 2.682002
H 5.245601 4.236149 1.218849
H 5.190378 3.482307 -1.144646
H 3.202872 2.317520 -2.036969
C 1.810933 -0.793474 3.243120
C 3.081720 -0.475756 3.545831
C 4.004009 -0.199714 2.531912
C 5.328696 0.140669 2.851123
C 6.224001 0.361603 1.874101
C 5.854133 0.249628 0.526834
C 4.594972 -0.073173 0.182162
C 3.612143 -0.300335 1.172811
C 2.269274 -0.640718 0.848526
H 1.146688 -1.018406 4.057005
H 3.393100 -0.439399 4.585706
H 5.639561 0.219766 3.886251
H 7.246804 0.621009 2.127112
H 6.594638 0.426951 -0.245462
H 4.349602 -0.141496 -0.869010
C 1.158008 -0.365197 -2.817879
C 1.571523 -1.539729 -3.323209
C 2.160691 -2.498993 -2.495022
C 2.580143 -3.730420 -3.024284
C 3.166065 -4.642742 -2.231341
C 3.370470 -4.380682 -0.869847
C 2.982466 -3.213337 -0.326413
C 2.355431 -2.222035 -1.117733
C 1.925420 -0.975728 -0.579316
H 0.730585 0.345808 -3.501476
H 1.447432 -1.741632 -4.382888
H 2.436370 -3.951903 -4.075212
H 3.488657 -5.591851 -2.646686
H 3.846123 -5.131893 -0.248660
H 3.149105 -3.058945 0.730770
N -2.231216 3.112525 1.026314
C -1.920864 3.563841 2.429156
H -0.838997 3.634909 2.557399
H -2.321693 2.837653 3.136016
H -2.366395 4.549518 2.617500
C -1.726258 4.144607 0.066519
H -2.030071 3.870786 -0.944602
H -0.638150 4.198640 0.119414
H -2.146977 5.129636 0.303591
C -3.719793 2.998676 0.853596
H -4.112563 2.301196 1.591680
H -3.927140 2.616641 -0.146501
H -4.192180 3.981673 0.979757

3.3 Simple molecules (B3LYP+D3/BS2 level)

***N*,*N*,3-trimethylbut-2-en-1-amine (Reactant)**
N -1.784101 -0.372984 0.116110
C -0.688743 -0.085139 -0.818263
C 0.611315 -0.681709 -0.350531
C 1.776705 -0.062004 -0.110648
C 2.988354 -0.842688 0.342669
C 2.019621 1.420634 -0.260522
H -0.597287 1.000972 -1.010685
H -0.957600 -0.546037 -1.779678
H 0.557611 -1.758459 -0.187117
H 2.413318 1.838637 0.676359
H 2.781254 1.611756 -1.029509
H 1.122966 1.983305 -0.529312
H 3.819856 -0.725023 -0.366785
H 3.352913 -0.473080 1.311597
H 2.776383 -1.911898 0.443271
C -3.077514 -0.052548 -0.469499
H -3.221273 -0.624741 -1.393117
H -3.877047 -0.327527 0.227710
H -3.193839 1.023962 -0.711615
C -1.601478 0.312181 1.389305
H -0.633631 0.040757 1.820337
H -1.635293 1.417218 1.292779
H -2.389174 0.008686 2.087929
BINAP-Rh(I)
Rh -0.000031 -2.460104 -0.000022
P -1.546401 -0.952108 -0.440608
P 1.546374 -0.952154 0.440593
C -2.760540 -1.852166 -1.478764
C -2.466758 -0.262137 0.978029
C 2.760486 -1.852254 1.478744
C 2.466758 -0.262185 -0.978028
C -0.912245 0.496969 -1.380088
C 0.912255 0.496929 1.380091
C -2.276307 -2.630612 -2.550935
C -3.154636 -3.385269 -3.327354
C -4.521401 -3.383361 -3.035850
C -5.006469 -2.625900 -1.967279
C -4.134194 -1.865692 -1.187059
H -1.212970 -2.635404 -2.780205
H -2.772133 -3.976561 -4.154070
H -5.205511 -3.974805 -3.637179
H -6.067784 -2.626942 -1.736971
H -4.523364 -1.287858 -0.356073
C -2.273941 -0.781825 2.265604
C -2.956892 -0.231040 3.349733
C -3.839810 0.833127 3.153341
C -4.044054 1.346559 1.870000
C -3.356693 0.806666 0.784462
H -1.571574 -1.597340 2.413956
H -2.793416 -0.627206 4.347640
H -4.365077 1.266258 3.999354
H -4.721546 2.180923 1.717334
H -3.493720 1.230397 -0.206632
C 2.276232 -2.630690 2.550911
C 3.154541 -3.385376 3.327325
C 4.521305 -3.383504 3.035819
C 5.006395 -2.626050 1.967253
C 4.134140 -1.865814 1.187038
H 1.212896 -2.635453 2.780182
H 2.772022 -3.976661 4.154038
H 5.205400 -3.974971 3.637144
H 6.067710 -2.627120 1.736944
H 4.523325 -1.287983 0.356057
C 2.273929 -0.781839 -2.265615
C 2.956894 -0.231046 -3.349731
C 3.839842 0.833092 -3.153314
C 4.044101 1.346486 -1.869961
C 3.356726 0.806586 -0.784435
H 1.571541 -1.597333 -2.413987
H 2.793406 -0.627184 -4.347647
H 4.365120 1.266229 -3.999317
H 4.721619 2.180826 -1.717274
H 3.493766 1.230290 0.206669
C -1.098200 0.540590 -2.788131
C -0.470770 1.496195 -3.549020
C 0.372479 2.465329 -2.950995
C 1.044525 3.443964 -3.730573
C 1.851436 4.386226 -3.135190
C 2.019143 4.386335 -1.729462
C 1.386322 3.446992 -0.947118
C 0.544901 2.456178 -1.527914
C -0.104747 1.444946 -0.744562
H -1.743956 -0.184920 -3.269056
H -0.619932 1.519335 -4.625409
H 0.905599 3.436562 -4.808480
H 2.358280 5.132805 -3.739462
H 2.655637 5.133830 -1.264786
H 1.532027 3.456510 0.126551
C 1.098208 0.540526 2.788134
C 0.470801 1.496137 3.549034
C -0.372419 2.465302 2.951019
C -1.044442 3.443943 3.730610
C -1.851327 4.386235 3.135237
C -2.019027 4.386370 1.729509
C -1.386228 3.447021 0.947153
C -0.544835 2.456177 1.527937
C 0.104785 1.444934 0.744574
H 1.743943 -0.185009 3.269051
H 0.619960 1.519259 4.625424
H -0.905521 3.436521 4.808517
H -2.358155 5.132817 3.739518
H -2.655499 5.133889 1.264841
H -1.531927 3.456562 -0.126517
(*E*)-*N*,*N*,3-trimethylbut-1-en-1-amine (Product)
N -1.944311 0.134999 -0.265488
C -0.628603 -0.292086 -0.450723
C 0.485484 0.177647 0.136357
C 1.876969 -0.282307 -0.223997
C 2.699972 0.866970 -0.837951
C 2.599147 -0.864965 1.005500
H 1.794753 -1.078526 -0.978859
H -0.542283 -1.078497 -1.200978
H 0.427680 0.946378 0.905692
H 3.610008 -1.206288 0.748997
H 2.693939 -0.106090 1.793479
H 2.043583 -1.711851 1.423199
H 2.798649 1.695338 -0.124014
H 3.711423 0.534753 -1.104907
H 2.215047 1.258829 -1.738715
C -2.968186 -0.896770 -0.348741
H -3.019456 -1.529243 0.555417
H -3.949395 -0.432651 -0.498371
H -2.766503 -1.547381 -1.206223
C -2.177814 1.137215 0.756440
H -1.948197 0.772858 1.774092
H -1.553966 2.015684 0.560999
H -3.226925 1.447472 0.727301
(*Z*)-*N*,*N*,3-trimethylbut-1-en-1-amine (Product)
N -1.765644 0.115569 -0.200753
C -0.792370 -0.783951 -0.635326
C 0.551315 -0.803458 -0.528787
C 1.535157 0.083872 0.201525
C 2.556461 -0.785664 0.959353
C 2.260047 1.031751 -0.775036
H 1.018192 0.694934 0.946928
H -1.244859 -1.592889 -1.209283
H 1.029368 -1.603135 -1.096096
H 3.001002 1.651614 -0.253551
H 2.786050 0.458046 -1.548868
H 1.550724 1.695119 -1.282953
H 3.117963 -1.422266 0.262841
H 3.280674 -0.163676 1.499938
H 2.057044 -1.440769 1.681804
C -3.043011 -0.456008 0.195245
H -3.036047 -0.853743 1.225548
H -3.827328 0.307457 0.130946
H -3.309606 -1.275363 -0.480439
C -1.413187 1.334011 0.499711
H -1.126566 1.170031 1.552920
H -0.587616 1.831853 -0.014500
H -2.275955 2.010491 0.489924
tetrahydrofuran (THF)
O -0.000001 1.251576 -0.000003
C -1.164210 0.429942 -0.134350
C -0.732301 -0.996037 0.231788
C 0.732304 -0.996034 -0.231790
C 1.164208 0.429945 0.134354
H -1.528401 0.475643 -1.172940
H -1.951928 0.824119 0.518757
H -1.345724 -1.764120 -0.249425
H -0.786488 -1.145404 1.317062
H 1.345730 -1.764117 0.249421
H 0.786492 -1.145396 -1.317064
H 1.528390 0.475645 1.172947
H 1.951930 0.824125 -0.518746
trimetylamine (TMA)
N -0.000005 0.000000 -0.375237
C -1.384366 0.106386 0.061403
H -1.962631 -0.738630 -0.329195
H -1.826696 1.029865 -0.329158
H -1.494662 0.114850 1.165477
C 0.600049 -1.252081 0.061408
H 1.620952 -1.330400 -0.329279
H 0.021433 -2.096909 -0.329082
H 0.647950 -1.351764 1.165480
C 0.784315 1.145692 0.061406
H 0.341629 2.069014 -0.329132
H 1.805214 1.067061 -0.329233
H 0.846857 1.236923 1.165480

3.4 Dissociative mechanism (B3LYP+D3/BS2 level)

1
Rh -1.894600 0.329882 -0.015482
P -0.556215 -1.456880 0.332145
P -0.115965 1.658326 -0.360307
C -1.472170 -2.646138 1.405328
C 0.056224 -2.430215 -1.093925
C -0.651718 3.073950 -1.411162
C 0.724374 2.413122 1.083589
C 0.970812 -1.067475 1.324852
C 1.254844 0.855702 -1.331231
N -3.544830 1.877133 0.328525
C -4.613402 0.828824 0.307387
C -4.167161 -0.218132 -0.684775
C -4.202643 -1.567189 -0.515442
C -3.794469 -2.492719 -1.631033
C -4.790812 -2.257477 0.684406
H -4.704807 0.407029 1.310268
H -5.587544 1.273749 0.051112
H -3.929889 0.157806 -1.681188
H -5.813969 -2.571487 0.433212
H -4.231246 -3.161308 0.934791
H -4.844350 -1.629817 1.574933
H -2.997772 -3.167155 -1.296751
H -4.643902 -3.124956 -1.922079
H -3.440796 -1.954059 -2.513577
C -3.400314 2.487763 1.667171
H -3.181330 1.710919 2.403354
H -2.565976 3.192495 1.648540
H -4.317235 3.019548 1.963743
C -3.870349 2.934165 -0.652732
H -3.947509 2.505213 -1.652148
H -4.826852 3.411733 -0.391760
H -3.084972 3.687975 -0.660510
C -2.112337 -2.135303 2.550598
C -2.793887 -2.980832 3.424025
C -2.872651 -4.349855 3.151999

C -2.268675 -4.862329 2.002780
C -1.568092 -4.018515 1.135027
H -2.065750 -1.068276 2.756778
H -3.270413 -2.572100 4.310707
H -3.407270 -5.010361 3.828326
H -2.334541 -5.923417 1.779749
H -1.097036 -4.436427 0.252235
C -0.362130 -2.075049 -2.383897
C 0.127309 -2.760158 -3.495561
C 1.035822 -3.807298 -3.326690
C 1.455830 -4.168260 -2.043872
C 0.974649 -3.479348 -0.931671
H -1.047335 -1.241184 -2.506334
H -0.189193 -2.468341 -4.492938
H 1.425373 -4.334444 -4.192586
H 2.176594 -4.969414 -1.911686
H 1.332906 -3.738860 0.060843
C -1.340452 2.790448 -2.605985
C -1.822660 3.819175 -3.411978
C -1.652878 5.151006 -3.021564
C -0.990767 5.443042 -1.828308
C -0.486504 4.412940 -1.028534
H -1.514380 1.755820 -2.891168
H -2.343743 3.583079 -4.335596
H -2.038251 5.954449 -3.642465
H -0.859768 6.475323 -1.516502
H 0.027225 4.659453 -0.106022
C 0.220602 2.183293 2.371086
C 0.872587 2.711115 3.485724
C 2.029358 3.475651 3.321676
C 2.534574 3.712588 2.040469
C 1.889814 3.179842 0.925613
H -0.660406 1.560201 2.491435
H 0.486677 2.512669 4.481581
H 2.543480 3.877714 4.189632
H 3.443111 4.292916 1.911656
H 2.303562 3.340558 -0.066224
C 1.003912 -1.337850 2.720977
C 2.035979 -0.889653 3.508970
C 3.108781 -0.149185 2.956345
C 4.170911 0.340184 3.762265
C 5.212705 1.043220 3.202196
C 5.231413 1.285733 1.807628
C 4.212933 0.828866 1.001876
C 3.118695 0.098565 1.546378
C 2.027961 -0.370653 0.739402
H 0.211756 -1.914073 3.180314
H 2.039654 -1.108554 4.573917
H 4.146511 0.144554 4.831266
H 6.022950 1.410509 3.825334
H 6.056458 1.840611 1.370091
H 4.238784 1.031536 -0.062222
C 1.385673 1.109911 -2.725004
C 2.264264 0.394040 -3.500908
C 3.080068 -0.616710 -2.937684
C 3.975542 -1.381638 -3.731359
C 4.774993 -2.344652 -3.159702
C 4.708498 -2.580728 -1.765482
C 3.845869 -1.859029 -0.971670
C 3.002452 -0.856058 -1.528480
C 2.072741 -0.104471 -0.733605
H 0.798574 1.889191 -3.191663
H 2.346175 0.606922 -4.563897
H 4.020017 -1.188064 -4.800067
H 5.459824 -2.922603 -3.773441
H 5.342491 -3.341338 -1.318799
H 3.800917 -2.059369 0.092172

4

Rh 1.984026 0.126728 0.171132
P 0.469915 -1.548995 -0.345134
P 0.286565 1.615822 0.417188
C 1.351777 -2.780014 -1.395351
C -0.286814 -2.505602 1.023348
C 0.825595 3.010770 1.502631
C -0.465596 2.391560 -1.065416
C -0.982478 -1.012435 -1.367029
C -1.163859 0.881135 1.325600
N 3.803824 -1.178019 0.691436
C 4.731843 -0.002514 0.720446
C 3.907722 1.255719 0.526666
C 3.546925 1.721678 -0.728609
C 3.144439 3.167685 -0.906132
C 3.955513 1.051590 -2.024269
H 5.285200 0.022640 1.666422
H 5.470880 -0.119837 -0.077021
H 3.820841 1.938254 1.368184
H 4.879701 1.520152 -2.396090
H 3.191531 1.204764 -2.793190
H 4.132240 -0.020430 -1.934901
H 2.291351 3.283178 -1.580038
H 3.989609 3.700691 -1.364398
H 2.915024 3.656630 0.040498
C 4.371242 -2.289761 -0.098602
H 4.507587 -1.982749 -1.135365
H 3.687473 -3.138191 -0.084872
H 5.340843 -2.601230 0.317766
C 3.531164 -1.658839 2.064957
H 3.158267 -0.834195 2.677532
H 4.446234 -2.060704 2.526898
H 2.773434 -2.443739 2.032346
C 2.081736 -2.292802 -2.495801
C 2.829569 -3.155925 -3.294085
C 2.883198 -4.519490 -2.989379
C 2.175713 -5.010668 -1.891448
C 1.409359 -4.149505 -1.100227
H 2.070246 -1.229085 -2.717630
H 3.379291 -2.764280 -4.145539
H 3.474287 -5.192666 -3.603349
H 2.215240 -6.068316 -1.646897
H 0.871196 -4.550189 -0.248597
C 0.021647 -2.175990 2.350398
C -0.590967 -2.852042 3.405456
C -1.516842 -3.863394 3.143603
C -1.834811 -4.193647 1.823545
C -1.229732 -3.514329 0.767602
H 0.704182 -1.356971 2.551516
H -0.360686 -2.574519 4.430009
H -2.002647 -4.382388 3.964434
H -2.570558 -4.965054 1.617105
H -1.506990 -3.751486 -0.255939
C 1.591691 2.692485 2.639521
C 2.073266 3.693628 3.481691
C 1.810369 5.035478 3.191927
C 1.060659 5.363337 2.061824
C 0.568506 4.360130 1.222781
H 1.815099 1.651274 2.858883
H 2.657094 3.427717 4.358503
H 2.191176 5.818501 3.841060
H 0.858228 6.404071 1.825604
H 0.006795 4.640273 0.339511
C 0.059937 2.100003 -2.331952
C -0.517078 2.644778 -3.478425
C -1.624521 3.488799 -3.369802
C -2.158809 3.779122 -2.112450
C -1.589334 3.226818 -0.965857
H 0.899937 1.416902 -2.410337
H -0.112019 2.399035 -4.455922
H -2.079689 3.907404 -4.262403
H -3.035119 4.414068 -2.025946
H -2.037117 3.422423 0.004477
C -1.004453 -1.269116 -2.765000
C -1.986419 -0.741049 -3.567313
C -3.013201 0.069526 -3.025515
C -4.021408 0.641118 -3.846584
C -5.020950 1.412682 -3.299917
C -5.050238 1.643923 -1.903605
C -4.082976 1.108770 -1.083170
C -3.032740 0.306853 -1.613328
C -1.994059 -0.243796 -0.789729
H -0.244890 -1.897418 -3.212190
H -1.985900 -0.948575 -4.634473
H -3.990053 0.453232 -4.916770
H -5.790086 1.842518 -3.934998
H -5.842179 2.252550 -1.476526
H -4.114892 1.304219 -0.017938
C -1.289045 1.103441 2.725119
C -2.247791 0.457955 3.467084
C -3.146991 -0.453109 2.862269
C -4.131967 -1.140260 3.620279
C -5.000768 -2.018063 3.013942
C -4.913895 -2.245894 1.619675
C -3.966881 -1.597295 0.859646
C -3.054651 -0.677505 1.450959
C -2.047325 0.007056 0.688200
H -0.629383 1.803479 3.221427
H -2.327824 0.648665 4.534439
H -4.187406 -0.956622 4.690248
H -5.753717 -2.536442 3.600485
H -5.599288 -2.942651 1.145624
H -3.909282 -1.791907 -0.204488

5

Rh -1.497277 -0.481373 -0.576649
P 0.235176 -1.740224 0.087922
P -0.247982 1.343137 -0.666650
C -0.618937 -3.222374 0.767464
C 1.471956 -2.374566 -1.097637
C -0.905877 2.523268 -1.919691
C -0.163883 2.274153 0.912823
C 1.197408 -0.960716 1.455478
C 1.529966 1.081127 -1.129844
N -3.573173 0.246271 -0.878044
C -4.244549 -0.833673 -0.062363
C -5.747523 -0.884452 -0.147381
C -6.618186 -0.412157 0.761396
C -8.105685 -0.544115 0.543071
C -6.239852 0.274016 2.050935
H -3.907509 -0.697159 0.967570
H -3.834295 -1.792571 -0.416993
H -6.150046 -1.361744 -1.039036
H -6.634526 1.298654 2.062964
H -6.694919 -0.243328 2.905241
H -5.162718 0.332005 2.224416
H -8.565799 -1.124505 1.353804
H -8.588361 0.442227 0.553731
H -8.344588 -1.033107 -0.405907
C -3.986590 0.161356 -2.299499
H -5.059879 0.362906 -2.413770
H -3.423846 0.897134 -2.874086
H -3.767578 -0.838190 -2.686546
C -3.913006 1.584977 -0.344967
H -4.997299 1.753499 -0.373344
H -3.559445 1.667818 0.684646
H -3.412563 2.343441 -0.947677
C -1.709613 -3.022454 1.638983
C -2.461481 -4.104074 2.096479
C -2.142839 -5.400031 1.681899
C -1.069269 -5.607678 0.813273
C -0.311469 -4.528304 0.354741
H -1.959622 -2.016036 1.966979
H -3.293801 -3.935367 2.773979
H -2.728990 -6.243669 2.034469
H -0.818757 -6.613645 0.489112
H 0.515696 -4.704581 -0.324314
C 1.279107 -2.152650 -2.468183
C 2.226420 -2.600208 -3.389352
C 3.366483 -3.275036 -2.947399
C 3.558272 -3.508041 -1.582838
C 2.617056 -3.057225 -0.659030
H 0.398388 -1.609422 -2.800561
H 2.079779 -2.413670 -4.449355
H 4.108481 -3.614980 -3.663944
H 4.450086 -4.021948 -1.237381
H 2.783505 -3.213352 0.403390
C -1.177999 2.025168 -3.207644
C -1.714917 2.853173 -4.192458
C -2.013603 4.186720 -3.896997
C -1.766185 4.684563 -2.617043
C -1.212279 3.861516 -1.632543
H -0.972877 0.980980 -3.431202
H -1.909015 2.456186 -5.184890
H -2.440795 4.831045 -4.659791
H -2.002081 5.717900 -2.379458
H -1.031464 4.265163 -0.642693
C -0.908350 1.844525 2.020587
C -0.806434 2.514393 3.239610
C 0.034172 3.622981 3.360285
C 0.775478 4.059555 2.259715
C 0.684625 3.385012 1.043013
H -1.531499 0.959329 1.926142
H -1.370957 2.162116 4.098240
H 0.121814 4.138689 4.311910
H 1.444002 4.909640 2.355335
H 1.292991 3.706375 0.202026
C 0.917361 -1.298281 2.806631
C 1.451613 -0.566654 3.839793
C 2.303813 0.537095 3.586895
C 2.852285 1.310595 4.644615
C 3.698209 2.363729 4.382140
C 4.030033 2.687134 3.044387
C 3.507628 1.961988 1.997403
C 2.627653 0.867804 2.230734
C 2.049723 0.104639 1.162661
H 0.279731 -2.146075 3.028953
H 1.230261 -0.836053 4.869524
H 2.595386 1.049647 5.668111
H 4.116882 2.945572 5.198124
H 4.701125 3.517309 2.843233
H 3.764198 2.226328 0.978159
C 1.953127 1.394348 -2.451243
C 3.204375 1.048899 -2.898425
C 4.118443 0.373152 -2.054775
C 5.403288 -0.019564 -2.515094
C 6.281839 -0.669777 -1.679518
C 5.906599 -0.952647 -0.344214
C 4.666887 -0.586926 0.129631
C 3.730409 0.085551 -0.706444
C 2.415988 0.446855 -0.253444
H 1.285678 1.919950 -3.121784
H 3.505728 1.295418 -3.913385
H 5.679591 0.205502 -3.541960
H 7.263100 -0.965191 -2.039322
H 6.603504 -1.465959 0.312140
H 4.394927 -0.820229 1.151935

33

Rh -1.764665 0.890951 -0.150639
P -0.934168 -1.143600 0.542310
P 0.335275 1.686121 -0.276160
C -2.019359 -1.794411 1.883888
C -0.868198 -2.427362 -0.769978
C 0.674871 3.372530 -0.973690
C 1.355820 1.885435 1.244892
C 0.765773 -1.277807 1.288352
C 1.132775 0.457810 -1.404208
N -2.935101 2.640774 -0.849677
C -4.251037 1.937134 -0.806641
C -4.044195 0.472412 -1.097001
C -4.472898 -0.553917 -0.311889
C -4.473154 -1.965943 -0.832267
C -5.136555 -0.389754 1.031591
H -4.948776 2.397292 -1.525069
H -4.673329 2.081560 0.188542
H -3.726098 0.228659 -2.109945
H -4.873235 -1.218343 1.693560
H -4.878090 0.541368 1.539464
H -6.227244 -0.416899 0.898646
H -3.938934 -2.636596 -0.153018
H -5.510573 -2.325588 -0.876605
H -4.033690 -2.053852 -1.827268
C -2.596908 3.021937 -2.242053
H -3.330125 3.743722 -2.632819
H -1.603517 3.471027 -2.263283
H -2.594126 2.135077 -2.878787
C -3.003200 3.855441 -0.007212
H -3.119271 3.565193 1.039513
H -2.085578 4.428182 -0.121739
H -3.852880 4.485642 -0.310009
C -2.393787 -0.894979 2.898457
C -3.188485 -1.314153 3.964592
C -3.642209 -2.635109 4.020686
C -3.288833 -3.532092 3.010723
C -2.474506 -3.118857 1.952559
H -2.066425 0.139596 2.842915
H -3.464082 -0.608431 4.743187
H -4.269187 -2.961921 4.845141
H -3.641049 -4.559040 3.046278
H -2.201946 -3.831628 1.181803
C -1.318590 -2.119890 -2.060600
C -1.212694 -3.057385 -3.088069
C -0.652779 -4.310594 -2.835982
C -0.190598 -4.622144 -1.554378
C -0.288543 -3.683834 -0.528450
H -1.718476 -1.130962 -2.259717
H -1.549842 -2.801955 -4.088767
H -0.559669 -5.036971 -3.637881
H 0.266591 -5.587752 -1.359846
H 0.114978 -3.916440 0.453177
C 0.324981 4.450597 -0.137983
C 0.551562 5.767578 -0.530279
C 1.151520 6.036903 -1.763968
C 1.543434 4.977899 -2.581206
C 1.313633 3.654666 -2.189860
H -0.095518 4.253669 0.844263
H 0.274786 6.582144 0.133090
H 1.331697 7.062490 -2.072348
H 2.042984 5.173627 -3.525806
H 1.666121 2.856640 -2.830521
C 0.768579 1.776877 2.509552
C 1.520960 2.027713 3.659180
C 2.863789 2.390094 3.549557
C 3.452981 2.512618 2.287389
C 2.701807 2.271988 1.140367
H -0.275378 1.488890 2.587105
H 1.057802 1.934362 4.637450
H 3.451241 2.579657 4.443238
H 4.499219 2.788309 2.197501
H 3.163322 2.376625 0.162839
C 0.908794 -1.602125 2.664021
C 2.137397 -1.558992 3.277589
C 3.297504 -1.181181 2.559542
C 4.562271 -1.058902 3.193748
C 5.672929 -0.671300 2.479663
C 5.564527 -0.397504 1.094101
C 4.353234 -0.512293 0.450085
C 3.182542 -0.900884 1.159909
C 1.897767 -0.962210 0.533050
H 0.039262 -1.878038 3.247376
H 2.226378 -1.800525 4.333718
H 4.635209 -1.272164 4.257064
H 6.635299 -0.576391 2.974117
H 6.445919 -0.093928 0.536485
H 4.281162 -0.295108 -0.609840
C 0.822016 0.567175 -2.793143
C 1.249194 -0.374160 -3.693629
C 1.986433 -1.508215 -3.265440
C 2.439546 -2.487587 -4.186244
C 3.107745 -3.609486 -3.749528
C 3.336362 -3.798545 -2.366979
C 2.903341 -2.866948 -1.448743
C 2.230265 -1.684919 -1.865227
C 1.772910 -0.686768 -0.934401
H 0.211085 1.392732 -3.141175
H 1.009616 -0.268745 -4.748803
H 2.246736 -2.336137 -5.245316
H 3.455275 -4.351892 -4.462032
H 3.855521 -4.689270 -2.024925
H 3.081456 -3.033273 -0.393365

36

Rh 1.370234 1.217575 -0.216704
P 1.016111 -1.043244 -0.497795
P -0.905365 1.757124 0.248062
C 2.209214 -1.672242 -1.755782
C 1.259100 -2.138271 0.945667
C -1.075129 3.382060 1.101065
C -2.071532 1.824733 -1.159056
C -0.654792 -1.431899 -1.197980
C -1.564400 0.479533 1.419375
N 2.815349 2.813834 -0.202505
C 3.432013 1.509521 -0.382507
C 4.328811 0.940746 0.631726
C 5.300823 0.028628 0.421329
C 6.039620 -0.578052 1.587543
C 5.707455 -0.507588 -0.926167
H 3.760068 1.364996 -1.411657
H 1.481687 0.923927 1.264739
H 4.124222 1.213508 1.664636
H 6.794892 -0.429538 -1.055209
H 5.455351 -1.572775 -1.005495
H 5.227762 -0.003241 -1.767434
H 5.850901 -1.659960 1.641301
H 7.125192 -0.456373 1.475688
H 5.742758 -0.136078 2.544434
C 3.112479 3.604349 1.005370
H 4.131418 4.009278 0.954521
H 2.390033 4.419701 1.074228
H 3.014389 2.986546 1.896323
C 2.785897 3.668591 -1.405244
H 3.765492 4.137515 -1.571906
H 2.532846 3.068357 -2.284267
H 2.024878 4.442726 -1.278434
C 2.491195 -0.866597 -2.873367
C 3.402756 -1.282425 -3.841919
C 4.056671 -2.510256 -3.700449

C 3.785477 -3.316533 -2.594237
C 2.865587 -2.903279 -1.626608
H 1.984933 0.089687 -2.995123
H 3.605824 -0.650962 -4.702282
H 4.773127 -2.834373 -4.449470
H 4.289449 -4.272072 -2.480431
H 2.668027 -3.539587 -0.770604
C 2.110965 -1.739631 1.987336
C 2.334356 -2.586040 3.073006
C 1.711718 -3.835546 3.128321
C 0.876034 -4.243826 2.087306
C 0.654291 -3.402421 0.997289
H 2.609487 -0.777702 1.941297
H 2.992269 -2.267930 3.876784
H 1.878049 -4.489216 3.979655
H 0.382622 -5.209941 2.128529
H -0.003994 -3.721586 0.195856
C -0.150715 3.689905 2.116642
C -0.187601 4.921514 2.768222
C -1.136880 5.878881 2.398620
C -2.046753 5.591476 1.380848
C -2.021209 4.351398 0.736857
H 0.600660 2.957504 2.397757
H 0.526657 5.136305 3.558448
H -1.162890 6.842616 2.898742
H -2.783698 6.332302 1.084040
H -2.734990 4.151007 -0.054176
C -1.554111 1.801074 -2.462312
C -2.408417 1.823752 -3.564799
C -3.790502 1.873082 -3.372247
C -4.315531 1.902542 -2.077885
C -3.462290 1.874938 -0.976103
H -0.478473 1.742506 -2.616663
H -1.997420 1.792339 -4.569700
H -4.458494 1.880850 -4.228411
H -5.390188 1.922701 -1.926182
H -3.876872 1.866364 0.027826
C -0.743882 -1.815647 -2.565893
C -1.959384 -1.941570 -3.192045
C -3.166460 -1.714946 -2.488271
C -4.430950 -1.832847 -3.124735
C -5.595458 -1.634447 -2.419583
C -5.539502 -1.307741 -1.043142
C -4.328731 -1.175166 -0.401806
C -3.102317 -1.368767 -1.099916
C -1.824052 -1.205831 -0.465167
H 0.159773 -2.013752 -3.128886
H -2.004451 -2.228718 -4.239528
H -4.460275 -2.090788 -4.180240
H -6.557996 -1.732534 -2.913080
H -6.461585 -1.157797 -0.488812
H -4.305143 -0.916032 0.649870
C -1.620772 0.785472 2.807479
C -1.886523 -0.186993 3.740140
C -2.145218 -1.522519 3.345970
C -2.430545 -2.536589 4.299363
C -2.728321 -3.818059 3.897523
C -2.757942 -4.134323 2.517810
C -2.472330 -3.176187 1.571616
C -2.143363 -1.843052 1.950004
C -1.813849 -0.826718 0.988973
H -1.457127 1.802949 3.141207
H -1.918666 0.067083 4.796691
H -2.418980 -2.276457 5.354696
H -2.951247 -4.585286 4.633167
H -3.009526 -5.143281 2.203230
H -2.499939 -3.437814 0.520456

45

Rh 1.777873 0.340255 -0.293204
P 0.102519 1.569669 0.537439
P 0.379660 -1.428678 -0.711862
C 0.783500 2.810777 1.713807
C -0.894403 2.503517 -0.679070
C 1.232902 -2.440204 -1.993325
C 0.055345 -2.566035 0.678402
C -1.120522 0.582438 1.521697
C -1.251798 -0.932248 -1.412498
N 4.563388 -1.005643 0.949092
C 4.196442 -0.257284 -0.242049
C 3.769478 1.162262 0.054355
C 3.347816 2.051950 -0.920324
C 3.080532 3.497738 -0.576500
C 3.487885 1.786188 -2.405685
H 4.965775 -0.299950 -1.041667
H 3.345472 -0.834041 -0.731822
H 3.892005 1.504602 1.077808
H 4.373514 2.320007 -2.781662
H 2.624961 2.177961 -2.954473
H 3.606079 0.730216 -2.659878
H 2.127486 3.835292 -0.998752
H 3.868020 4.124527 -1.016842
H 3.063410 3.674460 0.499852
C 4.527564 -2.447205 0.700234
H 3.517872 -2.746983 0.400821
H 4.779132 -2.979213 1.622142
H 5.236559 -2.765538 -0.087054
C 5.873354 -0.587620 1.452409
H 5.873739 0.485425 1.665577
H 6.689047 -0.797025 0.735001
H 6.088826 -1.117732 2.384475
C 1.749750 2.375694 2.640746
C 2.359116 3.280853 3.507414
C 2.024520 4.637690 3.449581
C 1.072951 5.077923 2.529115
C 0.452207 4.171394 1.665615
H 2.032255 1.326315 2.670672
H 3.099375 2.930151 4.220988
H 2.506402 5.346241 4.116901
H 0.812714 6.131113 2.475997
H -0.272163 4.535493 0.946039
C -0.511152 2.506515 -2.027790
C -1.278896 3.184064 -2.974875
C -2.431018 3.867740 -2.580798
C -2.816230 3.872183 -1.237779
C -2.056688 3.188107 -0.290211
H 0.370525 1.948574 -2.328760
H -0.984720 3.167838 -4.020391
H -3.034074 4.388062 -3.318927
H -3.721595 4.387253 -0.932066
H -2.381511 3.164127 0.746277
C 1.812424 -1.772257 -3.089772
C 2.542520 -2.476982 -4.045499
C 2.718610 -3.857594 -3.911178
C 2.155216 -4.526192 -2.823522
C 1.414748 -3.825043 -1.868207
H 1.687391 -0.697038 -3.191936
H 2.976531 -1.949889 -4.890511
H 3.293754 -4.407238 -4.650523
H 2.290467 -5.598353 -2.714013
H 0.988736 -4.359034 -1.025901
C 0.805939 -2.428518 1.855821
C 0.572097 -3.281696 2.934868
C -0.406805 -4.273641 2.844059
C -1.150872 -4.419030 1.670061
C -0.923253 -3.568135 0.590018
H 1.558145 -1.646400 1.926936
H 1.147862 -3.164943 3.848545
H -0.594747 -4.930444 3.688399
H -1.922152 -5.180018 1.603271
H -1.523270 -3.664365 -0.310485
C -1.011268 0.578525 2.939995
C -1.758835 -0.280022 3.707504
C -2.674703 -1.180397 3.111700
C -3.432454 -2.094332 3.891153
C -4.324775 -2.956096 3.296017
C -4.496123 -2.934710 1.890867
C -3.772599 -2.063111 1.108409
C -2.835899 -1.160879 1.688702
C -2.032684 -0.272284 0.897186
H -0.330239 1.261888 3.430800
H -1.653895 -0.272715 4.789424
H -3.294982 -2.097756 4.969335
H -4.901512 -3.650371 3.900108
H -5.204713 -3.614182 1.425895
H -3.911724 -2.066612 0.033937
C -1.455197 -0.991351 -2.817870
C -2.565648 -0.421678 -3.392066
C -3.545221 0.226396 -2.599442
C -4.691585 0.830962 -3.180744
C -5.644928 1.435103 -2.393452
C -5.488782 1.458552 -0.986462
C -4.384097 0.889695 -0.393944
C -3.375798 0.260002 -1.177031
C -2.199669 -0.316541 -0.593960
H -0.729269 -1.492201 -3.447545
H -2.707308 -0.471168 -4.468723
H -4.804712 0.801341 -4.261396
H -6.520674 1.889986 -2.847034
H -6.247298 1.932273 -0.369850
H -4.274284 0.922120 0.683791

46

Rh 1.828278 0.336645 -0.179025
P 0.088331 1.581620 0.527717
P 0.475995 -1.473474 -0.594621
C 0.688557 2.852592 1.717988
C -0.846397 2.481275 -0.763626
C 1.420134 -2.539743 -1.764799
C -0.011872 -2.560968 0.789919
C -1.200879 0.623700 1.453095
C -1.098197 -0.995884 -1.435746
N 5.013668 -0.780054 0.844869
C 4.294106 -0.102924 -0.195004
C 3.791090 1.280012 0.114253
C 3.293145 2.123459 -0.866337
C 2.977545 3.563645 -0.540728
C 3.420984 1.846944 -2.351427
H 4.893757 -0.111995 -1.111554
H 3.377022 -0.769269 -0.526165
H 3.944531 1.669053 1.116786
H 4.315865 2.363784 -2.729905
H 2.565571 2.254887 -2.897931
H 3.514537 0.788927 -2.605106
H 2.007747 3.860380 -0.954719
H 3.736456 4.209252 -1.003532
H 2.970327 3.757793 0.532769
C 4.259714 -0.975564 2.079187
H 3.889969 -0.022992 2.465033
H 4.917311 -1.412392 2.836023
H 3.395352 -1.649122 1.933034
C 5.577680 -2.042619 0.366597
H 6.212526 -1.860883 -0.505979
H 4.795998 -2.774301 0.086304
H 6.197747 -2.484145 1.151514
C 1.620308 2.443892 2.690327
C 2.176304 3.365289 3.575502
C 1.820465 4.715237 3.491776
C 0.901609 5.130483 2.527410
C 0.335288 4.206440 1.645230
H 1.915177 1.398864 2.741794
H 2.891225 3.033715 4.323281
H 2.260564 5.437641 4.172892
H 0.625380 6.178371 2.454001
H -0.361432 4.553010 0.890710
C -0.411484 2.427534 -2.095128
C -1.136944 3.068345 -3.099235
C -2.300005 3.772058 -2.780046
C -2.739737 3.829679 -1.455169
C -2.022678 3.181047 -0.451090
H 0.473761 1.849646 -2.339069
H -0.802353 3.006147 -4.130786
H -2.870537 4.263868 -3.562206
H -3.655041 4.358259 -1.207361
H -2.392466 3.195312 0.570439
C 2.022127 -1.927669 -2.881715
C 2.835444 -2.661397 -3.743048
C 3.079211 -4.014923 -3.489919
C 2.498065 -4.626760 -2.378935
C 1.670464 -3.897271 -1.520405
H 1.853157 -0.870904 -3.072604
H 3.283233 -2.177074 -4.606345
H 3.720063 -4.586564 -4.154800
H 2.685438 -5.677174 -2.175477
H 1.232854 -4.388994 -0.658872
C 0.492482 -2.314566 2.074149
C 0.104802 -3.118589 3.146021
C -0.786598 -4.173644 2.941732
C -1.291685 -4.425420 1.663201
C -0.911069 -3.620737 0.590897
H 1.157965 -1.471946 2.232594
H 0.486771 -2.911662 4.141552
H -1.096286 -4.793452 3.777929
H -1.997686 -5.234983 1.505591
H -1.328427 -3.801995 -0.395863
C -1.214830 0.685504 2.873761
C -2.035931 -0.131340 3.611791
C -2.899139 -1.058920 2.980087
C -3.735473 -1.926566 3.731703
C -4.572810 -2.817878 3.101288
C -4.605153 -2.876460 1.687287
C -3.803157 -2.051159 0.931732
C -2.924248 -1.116121 1.548934
C -2.055054 -0.261302 0.790038
H -0.573432 1.389688 3.388938
H -2.031068 -0.069370 4.697097
H -3.702607 -1.870568 4.816720
H -5.211081 -3.475595 3.684047
H -5.267834 -3.581835 1.194107
H -3.835233 -2.116079 -0.149253
C -1.206540 -1.111355 -2.847946
C -2.279265 -0.575896 -3.518826
C -3.314544 0.094121 -2.821552
C -4.423971 0.661202 -3.503658
C -5.434018 1.285508 -2.808392
C -5.372850 1.368715 -1.396458
C -4.305984 0.837249 -0.707831
C -3.241826 0.185888 -1.393508
C -2.105683 -0.359659 -0.708002
H -0.440343 -1.634648 -3.407151
H -2.347961 -0.672175 -4.599478
H -4.462864 0.586105 -4.587342
H -6.281217 1.710986 -3.338309
H -6.174648 1.859572 -0.852259
H -4.269853 0.917085 0.372246

47

Rh -1.638255 1.032796 -0.165775
P -0.927685 -1.338120 -0.102523
P 0.486672 1.890342 -0.031093
C -2.289056 -2.302911 0.692079
C -0.331917 -2.358705 -1.503668
C 0.649274 3.609993 -0.677089
C 1.147766 1.969343 1.675322
C 0.475537 -1.468012 1.119025
C 1.697868 0.863551 -0.997950
N -5.358598 0.181029 0.673456
C -4.473446 0.989668 0.093344
C -3.705290 0.681603 -1.037379
C -2.912410 1.663296 -1.756508
C -2.418355 1.197215 -3.114299
C -3.325080 3.129688 -1.771414
H -4.374563 1.966447 0.554721
H -1.975893 2.487646 0.325494
H -3.816030 -0.304226 -1.478478
H -4.075933 3.285063 -2.560391
H -2.470760 3.772819 -1.991755
H -3.752138 3.469423 -0.826241
H -1.487766 1.696892 -3.400273
H -3.164747 1.441609 -3.885204
H -2.252935 0.118385 -3.143070
C -5.739971 -1.093592 0.059740
H -4.870290 -1.737824 -0.076950
H -6.216520 -0.913098 -0.910519
H -6.443783 -1.605300 0.715512
C -6.039118 0.556913 1.909912
H -5.698420 1.540915 2.238178
H -5.816770 -0.174443 2.694271
H -7.122944 0.588666 1.753061
C -2.867923 -1.757166 1.854922
C -3.875207 -2.435830 2.539301
C -4.357873 -3.653388 2.049032
C -3.829765 -4.177522 0.868856
C -2.799146 -3.511788 0.196269
H -2.511735 -0.802760 2.236318
H -4.281139 -2.016557 3.456158
H -5.142707 -4.184210 2.580140
H -4.208639 -5.114793 0.471342
H -2.394473 -3.944153 -0.711957
C -0.174629 -1.765671 -2.762439
C 0.348836 -2.499390 -3.828057
C 0.716189 -3.832499 -3.644339
C 0.577247 -4.427788 -2.387127
C 0.069214 -3.692783 -1.318014
H -0.409009 -0.715972 -2.892951
H 0.485899 -2.022941 -4.794285
H 1.129605 -4.401943 -4.471473
H 0.885546 -5.458178 -2.235201
H -0.000017 -4.149583 -0.334555
C 0.141657 3.901149 -1.954882
C 0.203175 5.192714 -2.473955
C 0.756105 6.225337 -1.711589
C 1.245959 5.952163 -0.434850
C 1.197192 4.654104 0.081252
H -0.306384 3.110130 -2.545451
H -0.187584 5.393902 -3.467547
H 0.798896 7.234988 -2.109521
H 1.670262 6.749709 0.168360
H 1.578906 4.468704 1.078288
C 0.287539 1.733827 2.757572
C 0.774753 1.767323 4.064385
C 2.122566 2.045707 4.300201
C 2.982680 2.294062 3.227874
C 2.500597 2.251042 1.921059
H -0.762067 1.519106 2.570310
H 0.104158 1.572192 4.896384
H 2.504425 2.065002 5.316704
H 4.034007 2.495773 3.407153
H 3.180151 2.415786 1.089887
C 0.262247 -2.008531 2.417097
C 1.252103 -1.992134 3.368911
C 2.532108 -1.463071 3.079043
C 3.563634 -1.434538 4.055124
C 4.813960 -0.951909 3.744493
C 5.080563 -0.477349 2.437809
C 4.096729 -0.480576 1.474779
C 2.788475 -0.965282 1.761500
C 1.730478 -0.950947 0.788996
H -0.693090 -2.449285 2.669839
H 1.062127 -2.403428 4.357227
H 3.347312 -1.810401 5.051904
H 5.598853 -0.939597 4.495136
H 6.071161 -0.104328 2.193505
H 4.318643 -0.103588 0.483746
C 2.140768 1.335181 -2.266222
C 2.905917 0.550717 -3.093764
C 3.283489 -0.759294 -2.711451
C 4.076079 -1.581243 -3.555681
C 4.445807 -2.845779 -3.159563
C 4.030596 -3.339132 -1.899618
C 3.260481 -2.565915 -1.060812
C 2.862590 -1.250756 -1.433773
C 2.063290 -0.418373 -0.576442
H 1.896616 2.341170 -2.581722
H 3.242917 0.939695 -4.051488
H 4.388273 -1.189272 -4.520358
H 5.056075 -3.466818 -3.808888
H 4.319441 -4.340527 -1.593542
H 2.944241 -2.963470 -0.103979

53

Rh 1.476996 1.341066 -0.122893
P -0.751900 1.781810 0.072223
P 1.067793 -1.021985 -0.249479
C -1.062154 3.452055 0.795526
C -1.763837 1.746524 -1.453072
C 2.356856 -1.841259 -1.287887
C 0.987822 -1.969119 1.318493
C -1.563481 0.579005 1.243326
C -0.498770 -1.454872 -1.147411
N 5.059527 -0.403134 0.082476
C 4.428769 0.717247 -0.249150
C 3.601237 1.481304 0.586107
C 3.061397 2.775967 0.205502
C 2.870834 3.756951 1.347402
C 3.583178 3.467929 -1.052984
H 4.617125 1.057959 -1.261624
H 1.209443 2.683920 -0.893876
H 3.563946 1.212685 1.639418
H 4.607882 3.831934 -0.888011
H 2.956681 4.332823 -1.289076
H 3.583018 2.822352 -1.936186
H 2.121125 4.512618 1.100799
H 3.821100 4.276461 1.545142
H 2.565242 3.259446 2.272574
C 6.008005 -1.046414 -0.825383
H 6.008206 -0.530889 -1.786364
H 5.718410 -2.086512 -0.989350
H 7.016427 -1.010306 -0.397689
C 4.872308 -1.016479 1.396300
H 3.806762 -1.167084 1.593420
H 5.299433 -0.388322 2.186730
H 5.370517 -1.986378 1.408436
C -0.363778 3.820112 1.958381
C -0.531862 5.081463 2.526104
C -1.386576 6.009222 1.925182

C -2.067023 5.663437 0.757718
C -1.910476 4.393943 0.195087
H 0.325573 3.116882 2.413532
H 0.013455 5.343147 3.428593
H -1.514088 6.996048 2.360601
H -2.725262 6.381681 0.277258
H -2.445368 4.151906 -0.715502
C -1.137722 1.636687 -2.702041
C -1.901445 1.608750 -3.869718
C -3.292896 1.695807 -3.798802
C -3.923038 1.811960 -2.557127
C -3.164154 1.831698 -1.389002
H -0.055699 1.557416 -2.749852
H -1.409329 1.511729 -4.833242
H -3.887242 1.667106 -4.707359
H -5.005631 1.863153 -2.496801
H -3.660718 1.895291 -0.424897
C 2.775921 -1.155739 -2.443255
C 3.656639 -1.751166 -3.346042
C 4.146754 -3.037866 -3.100052
C 3.765908 -3.711465 -1.938029
C 2.877932 -3.117257 -1.035160
H 2.387193 -0.158676 -2.640591
H 3.953763 -1.216144 -4.243980
H 4.825481 -3.507546 -3.806160
H 4.150306 -4.707177 -1.734561
H 2.588265 -3.659341 -0.141800
C 1.286480 -1.298381 2.515207
C 1.190797 -1.960396 3.739569
C 0.794602 -3.298791 3.779800
C 0.488475 -3.971920 2.594401
C 0.575263 -3.309843 1.370566
H 1.557420 -0.246182 2.483772
H 1.406707 -1.426983 4.660947
H 0.707673 -3.812292 4.732722
H 0.154245 -5.004441 2.625587
H 0.286637 -3.824111 0.458243
C -1.740473 0.958439 2.603702
C -2.159868 0.056539 3.550493
C -2.452406 -1.283789 3.201640
C -2.885067 -2.228632 4.169927
C -3.196388 -3.518177 3.804987
C -3.083105 -3.914025 2.450710
C -2.655455 -3.023218 1.492210
C -2.324008 -1.680338 1.832130
C -1.859311 -0.731774 0.857659
H -1.561676 1.981332 2.906055
H -2.286932 0.373565 4.582653
H -2.973719 -1.909508 5.205265
H -3.533582 -4.232262 4.550717
H -3.333379 -4.932042 2.165857
H -2.565780 -3.347137 0.462317
C -0.438905 -1.915723 -2.491600
C -1.580816 -2.081621 -3.236217
C -2.856906 -1.816160 -2.683866
C -4.044950 -1.968412 -3.447185
C -5.277760 -1.722810 -2.887734
C -5.369690 -1.313050 -1.535792
C -4.235153 -1.148191 -0.773514
C -2.942688 -1.389467 -1.319968
C -1.738413 -1.193834 -0.563813
H 0.517359 -2.140258 -2.946076
H -1.512820 -2.427053 -4.264747
H -3.961001 -2.287752 -4.482823
H -6.181286 -1.845533 -3.477846
H -6.345276 -1.123828 -1.097063
H -4.324106 -0.824206 0.256619
54
Rh 1.649085 0.883929 -0.030938
P -0.433292 1.789779 0.082063
P 0.829305 -1.189621 -0.247489
C -0.270856 3.474322 0.821873
C -1.419382 2.033785 -1.440338
C 1.957111 -2.253841 -1.253067
C 0.546160 -2.085518 1.328316
C -1.516397 0.826619 1.239905
C -0.789295 -1.331811 -1.153345
N 4.876428 -1.141760 -0.040321
C 4.454614 0.092110 -0.342293
C 3.799460 0.966103 0.519291
C 3.555221 2.424314 0.189534
C 3.945937 3.332651 1.367579
C 4.155347 2.931355 -1.130486
H 4.674470 0.402531 -1.358701
H 2.421817 2.622493 0.073246
H 3.806550 0.722515 1.580290
H 5.249738 2.881995 -1.097145
H 3.867931 3.974656 -1.294856
H 3.801074 2.353265 -1.989896
H 3.604807 4.359290 1.203098
H 5.036226 3.339603 1.478504
H 3.508789 2.976669 2.305672
C 5.705997 -1.914500 -0.958491
H 5.757224 -1.409759 -1.924551
H 5.274579 -2.906432 -1.111216
H 6.722327 -2.019081 -0.558961
C 4.592918 -1.722809 1.265053
H 3.524700 -1.631804 1.489872
H 5.165848 -1.226210 2.058883
H 4.857051 -2.781122 1.249966
C 0.554662 3.628866 1.951787
C 0.759690 4.884752 2.519741
C 0.156168 6.012721 1.956412
C -0.652520 5.872967 0.828051
C -0.868475 4.613311 0.262844
H 1.041964 2.757981 2.383215
H 1.395719 4.983868 3.394902
H 0.319657 6.993758 2.392911
H -1.119928 6.745924 0.381455
H -1.495975 4.526842 -0.616902
C -0.812982 1.823696 -2.686271
C -1.548101 1.989164 -3.860704
C -2.890367 2.368833 -3.797802
C -3.498498 2.587030 -2.558739
C -2.768665 2.417169 -1.383785
H 0.227046 1.510124 -2.724517
H -1.075962 1.812269 -4.822923
H -3.465010 2.488796 -4.711581
H -4.546058 2.867212 -2.506771
H -3.252792 2.559788 -0.421646
C 2.483418 -1.701538 -2.435406
C 3.238424 -2.479994 -3.311245
C 3.501840 -3.819602 -3.006779
C 3.023212 -4.363803 -1.813946
C 2.255162 -3.586824 -0.940483
H 2.278032 -0.660113 -2.673251
H 3.618599 -2.044338 -4.231242
H 4.084823 -4.429986 -3.690481
H 3.236215 -5.399170 -1.562905
H 1.883615 -4.030514 -0.023598
C 0.951844 -1.495151 2.534898
C 0.703865 -2.133816 3.750051
C 0.052972 -3.369137 3.770746
C -0.354564 -3.962956 2.573787
C -0.118722 -3.321573 1.358681
H 1.427864 -0.518420 2.511444
H 1.004435 -1.659841 4.680168
H -0.149867 -3.862005 4.716944
H -0.882311 -4.911590 2.588157
H -0.482314 -3.765850 0.436401
C -1.646137 1.248463 2.591577
C -2.252582 0.448537 3.529252
C -2.783743 -0.816076 3.175341
C -3.405618 -1.657884 4.135744
C -3.942905 -2.870006 3.767525
C -3.877443 -3.289628 2.417144
C -3.271446 -2.500398 1.465754
C -2.702148 -1.248623 1.810309
C -2.043998 -0.404897 0.847466
H -1.269993 2.218532 2.892092
H -2.340768 0.788142 4.558278
H -3.455735 -1.321384 5.168182
H -4.422180 -3.503964 4.507863
H -4.307371 -4.245105 2.130118
H -3.222293 -2.840507 0.438025
C -0.794057 -1.773193 -2.505700
C -1.934763 -1.720757 -3.268059
C -3.149557 -1.233850 -2.729151
C -4.329525 -1.145732 -3.514666
C -5.503312 -0.676672 -2.971476
C -5.541759 -0.276687 -1.614056
C -4.412837 -0.346265 -0.829211
C -3.179538 -0.823353 -1.357813
C -1.975047 -0.870493 -0.577153
H 0.112896 -2.158633 -2.952691
H -1.913086 -2.056957 -4.301652
H -4.287070 -1.458551 -4.554797
H -6.401533 -0.614315 -3.578913
H -6.471083 0.091415 -1.188751
H -4.459370 -0.026779 0.204925
95
Rh -1.424464 1.411790 0.015785
P 0.839729 1.747811 -0.328738
P -1.142324 -0.977208 0.624224
C 1.273935 3.310705 -1.223166
C 1.930231 1.731983 1.142363
C -2.364242 -1.707672 1.799831
C -1.325588 -1.956723 -0.918823
C 1.425866 0.351392 -1.414671
C 0.515452 -1.442411 1.303739
N -4.737693 0.482662 -1.058429
C -4.016033 1.491971 -0.555828
C -3.498502 1.619542 0.745259
C -2.683577 2.757144 1.128569
C -2.273017 2.838153 2.585166
C -2.921743 4.147295 0.553831
H -1.484646 2.701117 -0.850074
H -3.871326 2.310182 -1.252292
H -3.728000 0.866110 1.491012
H -1.998480 4.732923 0.589329
H -3.674986 4.667098 1.164264
H -3.271481 4.144151 -0.480637
H -3.083385 3.291563 3.176455
H -1.391164 3.476103 2.699379
H -2.054197 1.860143 3.018223
C -5.027816 0.420002 -2.489415
H -6.091932 0.214842 -2.645934
H -4.441076 -0.381096 -2.956731
H -4.776248 1.369556 -2.966086
C -5.044448 -0.738670 -0.320224
H -4.334619 -1.528266 -0.589406
H -6.055986 -1.065831 -0.579083
H -4.995882 -0.573806 0.754649
C 0.474944 3.717538 -2.308052
C 0.760080 4.887142 -3.009554
C 1.835584 5.691525 -2.623437
C 2.618683 5.313605 -1.534057
C 2.345209 4.131355 -0.840441
H -0.384607 3.120979 -2.599363
H 0.134274 5.176761 -3.849070
H 2.052072 6.609882 -3.161430
H 3.447479 5.938760 -1.214272
H 2.964623 3.870300 0.008562
C 1.367173 1.784444 2.423951
C 2.185770 1.784596 3.554080
C 3.573795 1.739972 3.412301
C 4.143277 1.686805 2.137834
C 3.327359 1.675134 1.008494
H 0.288720 1.805429 2.531305
H 1.738746 1.814206 4.543750
H 4.211334 1.736384 4.291525
H 5.220776 1.625341 2.022999
H 3.776217 1.597018 0.022792
C -2.612114 -1.012080 2.996343
C -3.569524 -1.461373 3.905630
C -4.309302 -2.613358 3.626022
C -4.073277 -3.313393 2.441617
C -3.106619 -2.868356 1.536351
H -2.041190 -0.116719 3.221197
H -3.739879 -0.910725 4.826387
H -5.061923 -2.962435 4.326837
H -4.640213 -4.212989 2.218941
H -2.947118 -3.424496 0.619590
C -1.758522 -1.305520 -2.085526
C -1.874479 -2.006326 -3.286387
C -1.564922 -3.367508 -3.332892
C -1.133941 -4.023615 -2.177639
C -1.006352 -3.321958 -0.979319
H -1.971911 -0.238542 -2.054328
H -2.186755 -1.486292 -4.187945
H -1.644461 -3.912690 -4.268694
H -0.868323 -5.075430 -2.215896
H -0.629283 -3.829139 -0.095844
C 1.447552 0.577495 -2.820321
C 1.657297 -0.447883 -3.707997
C 1.883860 -1.769032 -3.251762
C 2.106290 -2.839925 -4.157725
C 2.361191 -4.111386 -3.697357
C 2.400930 -4.360026 -2.304848
C 2.174955 -3.344239 -1.403094
C 1.904639 -2.016243 -1.841627
C 1.644903 -0.939969 -0.922604
H 1.306683 1.580096 -3.204111
H 1.667415 -0.248657 -4.776740
H 2.081761 -2.632227 -5.224476
H 2.538544 -4.922604 -4.397480
H 2.609564 -5.363157 -1.943752
H 2.200924 -3.557586 -0.341382
C 0.617836 -1.812184 2.673490
C 1.839995 -1.977832 3.276042
C 3.039761 -1.805863 2.544506
C 4.311897 -1.957859 3.157479
C 5.468511 -1.810764 2.426747
C 5.395537 -1.507609 1.045909
C 4.176649 -1.345232 0.426318
C 2.959302 -1.479056 1.152810
C 1.674795 -1.272898 0.543664
H -0.280228 -1.969459 3.258109
H 1.896499 -2.252523 4.326335
H 4.353725 -2.198928 4.216594
H 6.436919 -1.932654 2.903156
H 6.310575 -1.400605 0.470279
H 4.140652 -1.105303 -0.629754
96
Rh 1.656636 0.877432 -0.176279
P -0.397593 1.759063 0.286512
P 0.801631 -1.166556 -0.499027
C -0.133106 3.318914 1.237600
C -1.484574 2.219431 -1.110860
C 1.879691 -2.153736 -1.644688
C 0.601769 -2.197550 1.004401
C -1.417274 0.662886 1.376609
C -0.867306 -1.188745 -1.309276
N 4.639501 -1.169194 0.514819
C 4.044742 0.036483 0.598944
C 3.856906 0.950393 -0.441532
C 3.562894 2.418927 -0.210169
C 3.887525 3.249380 -1.461521
C 4.184871 3.035821 1.053577
H 2.425155 2.596704 -0.063432
H 3.758086 0.310215 1.610357
H 4.201124 0.678871 -1.436084
H 3.850210 4.070502 1.178134
H 5.277165 3.029123 0.969733
H 3.905717 2.489113 1.958865
H 4.970182 3.258957 -1.632832
H 3.551416 4.284250 -1.341218
H 3.399658 2.829958 -2.347393
C 4.438811 -2.170274 1.557240
H 5.391660 -2.651181 1.801506
H 3.724607 -2.936204 1.231147
H 4.038163 -1.697080 2.456075
C 5.327369 -1.612323 -0.693660
H 4.644001 -2.045780 -1.426522
H 6.065909 -2.368237 -0.413533
H 5.853984 -0.768806 -1.148084
C 0.834957 3.311963 2.259839
C 1.123321 4.471524 2.977154
C 0.459050 5.663368 2.672877
C -0.493965 5.683782 1.653894
C -0.792159 4.520149 0.939783
H 1.366038 2.390687 2.487944
H 1.869807 4.447276 3.766151
H 0.687116 6.570514 3.224924
H -1.009669 6.607949 1.409138
H -1.530117 4.557849 0.146370
C -0.970555 2.180740 -2.414732
C -1.785573 2.504313 -3.500003
C -3.116319 2.871649 -3.289945
C -3.632639 2.918708 -1.992406
C -2.822929 2.590311 -0.906670
H 0.060361 1.873948 -2.571575
H -1.384790 2.459564 -4.508661
H -3.753401 3.114376 -4.135386
H -4.671357 3.188196 -1.828026
H -3.237493 2.596535 0.097600
C 2.360597 -1.471440 -2.789712
C 3.156712 -2.141049 -3.718149
C 3.506702 -3.478088 -3.503367
C 3.056396 -4.135732 -2.357296
C 2.244547 -3.470665 -1.433657
H 2.106817 -0.425963 -2.946974
H 3.510481 -1.619043 -4.602739
H 4.131714 -4.000215 -4.221966
H 3.332987 -5.171076 -2.179299
H 1.905007 -3.999336 -0.550086
C 1.051359 -1.707603 2.239119
C 0.871136 -2.454736 3.402783
C 0.244127 -3.701252 3.343005
C -0.209410 -4.195493 2.117726
C -0.041267 -3.444666 0.955018
H 1.507589 -0.722610 2.280362
H 1.204124 -2.057091 4.357417
H 0.094076 -4.279428 4.249894
H -0.720415 -5.152191 2.071523
H -0.439809 -3.813741 0.014129
C -1.452612 0.912081 2.775740
C -2.015797 0.006898 3.641995
C -2.589481 -1.197723 3.165832
C -3.162394 -2.149696 4.050766
C -3.733472 -3.305371 3.569153
C -3.753255 -3.555170 2.175923
C -3.198344 -2.655413 1.293894
C -2.596459 -1.450736 1.755901
C -1.986800 -0.504840 0.865107
H -1.033892 1.830654 3.169291
H -2.033457 0.213357 4.709263
H -3.146432 -1.943912 5.118046
H -4.173991 -4.024678 4.253433
H -4.209114 -4.466789 1.800076
H -3.215137 -2.865047 0.230797
C -0.953907 -1.453267 -2.703985
C -2.135266 -1.294082 -3.385299
C -3.309987 -0.872221 -2.717113
C -4.532715 -0.680152 -3.413722
C -5.667644 -0.279836 -2.746935
C -5.622595 -0.055470 -1.349954
C -4.450527 -0.227513 -0.648617
C -3.255491 -0.638201 -1.305273
C -2.008823 -0.790724 -0.608906
H -0.077829 -1.786091 -3.246117
H -2.178624 -1.495348 -4.452709
H -4.554689 -0.858848 -4.485676
H -6.599010 -0.137939 -3.287359
H -6.521564 0.258443 -0.827140
H -4.433130 -0.041822 0.418588
102
Rh 1.852989 0.687109 -0.487715
P 0.807070 -1.557270 -0.344509
P -0.154023 1.761093 -0.313325
C 1.694513 -2.636018 -1.541973
C 0.728220 -2.489663 1.230003
C -0.135629 3.572385 0.045514
C -1.298152 1.706280 -1.749161
C -0.951100 -1.598263 -0.943872
C -0.978021 0.979080 1.147720
N 3.177083 2.319945 2.046084
C 3.466929 1.825581 0.816776
C 3.809178 0.454564 0.554957
C 4.263869 -0.027153 -0.668450
C 4.658640 -1.480223 -0.756525
C 4.758166 0.814546 -1.819282
H 3.821370 2.585433 0.134412
H 2.282066 2.005061 -1.222673
H 3.720855 -0.266569 1.360769
H 4.274319 0.502822 -2.751968
H 4.590629 1.885239 -1.696377
H 5.837096 0.645056 -1.946945
H 4.420121 -1.906303 -1.733957
H 5.748048 -1.557164 -0.627713
H 4.178621 -2.092123 0.010747
C 2.827331 3.731052 2.183859
H 1.740416 3.870689 2.235048
H 3.284004 4.136003 3.092538
H 3.193301 4.291904 1.322494
C 2.829598 1.471301 3.180916
H 3.506058 0.616589 3.247523
H 2.943801 2.055646 4.097324
H 1.795376 1.113949 3.120352
C 1.965793 -2.095105 -2.812889
C 2.688696 -2.822311 -3.756940
C 3.172092 -4.095211 -3.436630

C 2.918192 -4.634339 -2.174842
C 2.178710 -3.913112 -1.232848
H 1.612506 -1.097126 -3.061732
H 2.883332 -2.393890 -4.736187
H 3.744185 -4.660409 -4.166569
H 3.293795 -5.620974 -1.918993
H 1.994385 -4.346539 -0.255710
C 1.322361 -1.936266 2.370748
C 1.224569 -2.581425 3.604135
C 0.533790 -3.789933 3.703769
C -0.062939 -4.349301 2.569905
C 0.025077 -3.700081 1.339779
H 1.827493 -0.981291 2.291486
H 1.674210 -2.135570 4.487006
H 0.448807 -4.290422 4.663825
H -0.612233 -5.282920 2.649076
H -0.467268 -4.121850 0.467753
C 0.849103 4.371580 -0.554091
C 0.840143 5.757314 -0.389614
C -0.150083 6.365404 0.384354
C -1.142715 5.581498 0.976723
C -1.143305 4.197064 0.801068
H 1.625882 3.903759 -1.150865
H 1.608712 6.360589 -0.864843
H -0.152883 7.443000 0.520191
H -1.923016 6.046894 1.572175
H -1.927007 3.606623 1.265168
C -0.851059 1.154730 -2.956502
C -1.688688 1.127669 -4.072256
C -2.977864 1.655488 -3.989126
C -3.425041 2.220455 -2.791592
C -2.588840 2.252144 -1.678407
H 0.152650 0.743198 -3.016068
H -1.334993 0.691265 -5.001982
H -3.633710 1.629157 -4.854439
H -4.429852 2.625364 -2.721176
H -2.949663 2.687327 -0.752190
C -1.277473 -2.261028 -2.158301
C -2.540049 -2.178596 -2.694071
C -3.560163 -1.440466 -2.046856
C -4.859180 -1.311490 -2.606305
C -5.838311 -0.596606 -1.955309
C -5.557292 0.015360 -0.709502
C -4.306722 -0.089148 -0.143362
C -3.267000 -0.810451 -0.794894
C -1.941202 -0.892330 -0.259147
H -0.521226 -2.835045 -2.679206
H -2.767068 -2.682634 -3.630121
H -5.065524 -1.790030 -3.560192
H -6.829026 -0.503874 -2.390634
H -6.336780 0.572283 -0.197341
H -4.104028 0.388548 0.808622
C -0.688502 1.525173 2.432960
C -1.101849 0.903392 3.584605
C -1.830224 -0.311680 3.533187
C -2.278082 -0.961163 4.713061
C -2.984561 -2.140162 4.641224
C -3.265469 -2.718629 3.380811
C -2.839121 -2.114378 2.219283
C -2.113392 -0.890909 2.253994
C -1.660651 -0.233471 1.058447
H -0.140278 2.457196 2.503926
H -0.878954 1.344974 4.553034
H -2.056104 -0.506835 5.675403
H -3.328397 -2.628288 5.548479
H -3.820626 -3.650821 3.329811
H -3.056921 -2.573651 1.262533

107

Rh 1.910844 -0.107332 -0.332099
P 0.202023 -1.629828 -0.384650
P 0.402571 1.611772 -0.202002
C 0.606847 -3.014774 -1.524005
C -0.146183 -2.382309 1.250592
C 1.118955 3.271520 0.163077
C -0.645755 2.045286 -1.645703
C -1.472099 -1.077315 -0.978523
C -0.673556 1.176159 1.234054
N 4.608155 1.190889 1.541010
C 4.224678 0.526738 0.330893
C 3.811501 -0.907349 0.478349
C 3.787286 -1.801676 -0.571069
C 3.609383 -3.271080 -0.270960
C 4.304556 -1.496177 -1.957971
H 5.025111 0.633031 -0.407337
H 3.352099 1.120783 -0.213777
H 3.702125 -1.288025 1.490835
H 3.698525 -2.005125 -2.711893
H 4.327158 -0.431603 -2.202844
H 5.327630 -1.890813 -2.050895
H 3.123920 -3.800722 -1.092241
H 4.603025 -3.721512 -0.136113
H 3.032954 -3.440228 0.643184
C 5.345836 2.433646 1.312879
H 4.714682 3.234903 0.892748
H 5.754700 2.784269 2.265205
H 6.184973 2.249605 0.634924
C 3.540308 1.374031 2.518348
H 3.001541 0.439631 2.689879
H 3.974245 1.696950 3.469474
H 2.812819 2.134190 2.188118
C 1.119160 -2.684974 -2.791191
C 1.461028 -3.684531 -3.700812
C 1.306879 -5.029594 -3.350450
C 0.802737 -5.364976 -2.092958
C 0.450212 -4.364607 -1.183151
H 1.254984 -1.640443 -3.059612
H 1.852778 -3.415587 -4.677776
H 1.580317 -5.810179 -4.054293
H 0.684952 -6.408017 -1.813854
H 0.073502 -4.643644 -0.205614
C 0.683035 -2.096806 2.343910
C 0.400743 -2.630867 3.601401
C -0.712369 -3.454456 3.776866
C -1.545009 -3.742284 2.692122
C -1.269186 -3.205063 1.436184
H 1.524478 -1.426128 2.209167
H 1.040987 -2.390981 4.445562
H -0.939184 -3.861911 4.757564
H -2.422389 -4.366920 2.829237
H -1.942158 -3.401937 0.606415
C 2.254998 3.664515 -0.561840
C 2.782324 4.947743 -0.419007
C 2.181342 5.857567 0.454253
C 1.039346 5.483533 1.164482
C 0.503149 4.203043 1.013740
H 2.723057 2.970506 -1.255006
H 3.660194 5.236386 -0.990092
H 2.594728 6.854849 0.572267
H 0.557650 6.190303 1.834063
H -0.394277 3.936889 1.562542
C -0.400842 1.444152 -2.886195
C -1.151714 1.811394 -4.004209
C -2.149028 2.780625 -3.888075
C -2.388679 3.392699 -2.654293
C -1.636255 3.033621 -1.539044
H 0.369974 0.682764 -2.968341
H -0.960993 1.335347 -4.961813
H -2.738356 3.061528 -4.756037
H -3.167689 4.142909 -2.559338
H -1.829573 3.512263 -0.583821
C -1.949384 -1.537294 -2.236565
C -3.101918 -1.031436 -2.786682
C -3.851784 -0.033447 -2.119540
C -5.011665 0.543902 -2.700878
C -5.719946 1.518979 -2.036794
C -5.298151 1.952635 -0.756151
C -4.179024 1.410341 -0.165859
C -3.417537 0.406362 -0.827986
C -2.215870 -0.133382 -0.266490
H -1.399445 -2.294582 -2.779946
H -3.444302 -1.390318 -3.753908
H -5.327766 0.202450 -3.683114
H -6.604627 1.956752 -2.489848
H -5.863669 2.720886 -0.236656
H -3.864037 1.755247 0.812430
C -0.200335 1.492012 2.541086
C -0.840034 1.020289 3.659302
C -1.994602 0.204237 3.549584
C -2.672660 -0.281674 4.697956
C -3.789686 -1.076364 4.572465
C -4.268149 -1.422452 3.286828
C -3.624921 -0.975105 2.154050
C -2.473723 -0.143811 2.245633
C -1.773410 0.334520 1.085713
H 0.681572 2.109484 2.658167
H -0.464900 1.272634 4.647974
H -2.294756 -0.010174 5.680327
H -4.305732 -1.438494 5.456840
H -5.149538 -2.050326 3.192728
H -4.000264 -1.256821 1.177523

113

Rh 1.837031 0.048570 -0.467967
P 0.268633 -1.584949 -0.466791
P 0.210113 1.643818 -0.284757
C 0.710568 -2.894791 -1.677138
C 0.114534 -2.386208 1.175812
C 0.888572 3.334675 -0.028419
C -0.996197 1.988947 -1.621010
C -1.491559 -1.166206 -0.890852
C -0.675592 1.151672 1.251093
N 4.180698 1.280648 1.753817
C 4.056409 0.846695 0.371380
C 3.799173 -0.635215 0.258246
C 3.857110 -1.360023 -0.914356
C 3.840618 -2.868187 -0.844134
C 4.293279 -0.790866 -2.243915
H 4.908103 1.166799 -0.262796
H 3.196036 1.445759 -0.079995
H 3.705563 -1.169011 1.200447
H 3.658110 -1.176445 -3.048018
H 4.274744 0.301271 -2.285464
H 5.320161 -1.121157 -2.460941
H 3.379522 -3.316086 -1.726128
H 4.880142 -3.223751 -0.805086
H 3.322854 -3.235361 0.046181
C 5.444364 0.813070 2.331279
H 6.327022 1.245637 1.823596
H 5.482500 1.093908 3.387541
H 5.514905 -0.276585 2.269212
C 4.059188 2.733177 1.881786
H 3.075338 3.059568 1.537357
H 4.161675 3.008667 2.935323
H 4.829357 3.281424 1.307044
C 1.088067 -2.480137 -2.967313
C 1.466206 -3.413943 -3.930242
C 1.487356 -4.775751 -3.611323
C 1.123178 -5.193815 -2.330692
C 0.733190 -4.260350 -1.366513
H 1.096299 -1.420643 -3.209091
H 1.752633 -3.080739 -4.923763
H 1.790679 -5.504678 -4.357089
H 1.145081 -6.249347 -2.075626
H 0.468339 -4.601977 -0.372310
C 0.936987 -1.987202 2.238785
C 0.774387 -2.554908 3.503107
C -0.208359 -3.522270 3.716250
C -1.037748 -3.917817 2.663114
C -0.885118 -3.347790 1.400879
H 1.665498 -1.198465 2.085103
H 1.403526 -2.224972 4.324769
H -0.341250 -3.955667 4.703050
H -1.818105 -4.654100 2.830571
H -1.560663 -3.629689 0.597993
C 1.921212 3.742661 -0.890094
C 2.445909 5.031509 -0.808050
C 1.943770 5.930697 0.136331
C 0.901298 5.541764 0.979720
C 0.367174 4.254070 0.893611
H 2.311353 3.051335 -1.634124
H 3.245219 5.332855 -1.478870
H 2.356762 6.932541 0.207814
H 0.497166 6.241981 1.705125
H -0.449684 3.971258 1.549634
C -0.822327 1.414610 -2.885314
C -1.700779 1.728462 -3.923733
C -2.754251 2.616847 -3.702868
C -2.923774 3.202507 -2.444772
C -2.044585 2.897435 -1.409059
H -0.007490 0.714753 -3.047214
H -1.565318 1.273362 -4.900707
H -3.442300 2.855285 -4.508645
H -3.745785 3.889483 -2.268643
H -2.183118 3.353162 -0.433052
C -2.046727 -1.661993 -2.102028
C -3.287578 -1.258288 -2.530881
C -4.051688 -0.331925 -1.781584
C -5.307491 0.144908 -2.242185
C -6.028088 1.055024 -1.503416
C -5.522260 1.521014 -0.265299
C -4.309491 1.074846 0.209201
C -3.533088 0.140563 -0.532974
C -2.240165 -0.293238 -0.095537
H -1.486102 -2.364637 -2.705319
H -3.690692 -1.641714 -3.464695
H -5.687158 -0.220516 -3.192769
H -6.986345 1.416591 -1.864763
H -6.097689 2.237446 0.313914
H -3.932071 1.443387 1.156209
C -0.085390 1.506459 2.498995
C -0.574234 0.998792 3.676276
C -1.670877 0.098934 3.683882
C -2.193269 -0.423166 4.895892
C -3.243836 -1.312723 4.884943
C -3.808442 -1.722981 3.654281
C -3.318134 -1.240281 2.461193
C -2.243328 -0.308425 2.435085
C -1.708707 0.217899 1.210350
H 0.772377 2.169578 2.520176
H -0.116851 1.280094 4.621437
H -1.747970 -0.105108 5.835118
H -3.640392 -1.702404 5.817941
H -4.634344 -2.428503 3.650823
H -3.756917 -1.570223 1.527025

114

Rh 1.774591 0.265378 -0.803054
P 0.460303 -1.520029 -0.539030
P 0.014765 1.678001 -0.387628
C 1.009415 -2.840897 -1.690084
C 0.474015 -2.230683 1.148512
C 0.710681 3.356712 -0.114650
C -1.321249 1.983129 -1.600316
C -1.353314 -1.305268 -0.899710
C -0.737318 1.126885 1.199354
N 3.472297 0.978854 2.148585
C 4.059041 0.940179 0.809947
C 3.741584 -0.332415 0.041841
C 3.933452 -0.488483 -1.325468
C 4.156394 -1.861260 -1.913414
C 4.382762 0.642905 -2.231789
H 5.163621 1.070131 0.842819
H 3.688369 1.819387 0.265425
H 3.674589 -1.240020 0.637818
H 3.910697 0.561534 -3.217693
H 4.183861 1.644561 -1.836793
H 5.469631 0.579697 -2.390357
H 3.701762 -1.963342 -2.901735
H 5.237669 -2.021147 -2.032355
H 3.765530 -2.655185 -1.274035
C 4.183791 0.112046 3.086540
H 5.239024 0.412414 3.227654
H 3.684572 0.145787 4.060200
H 4.166236 -0.925883 2.742143
C 3.410996 2.348859 2.655408
H 2.833219 2.975668 1.968441
H 2.908386 2.353450 3.628131
H 4.409673 2.806688 2.785466
C 1.106775 -2.522327 -3.057494
C 1.539117 -3.474234 -3.978054
C 1.912326 -4.749193 -3.541191
C 1.844335 -5.064809 -2.183822
C 1.390857 -4.119334 -1.259938
H 0.852525 -1.521015 -3.395908
H 1.599644 -3.218046 -5.031942
H 2.260170 -5.489089 -4.256024
H 2.141905 -6.050446 -1.837923
H 1.345502 -4.381521 -0.208905
C 1.215192 -1.624189 2.171458
C 1.166825 -2.146544 3.465104
C 0.382115 -3.264986 3.746768
C -0.368815 -3.863555 2.730842
C -0.331135 -3.346072 1.438005
H 1.779896 -0.718109 1.975319
H 1.729445 -1.661196 4.257310
H 0.339125 -3.660940 4.757096
H -0.997660 -4.721337 2.949758
H -0.941280 -3.796276 0.659668
C 1.835599 3.717883 -0.875530
C 2.408329 4.983821 -0.745758
C 1.855890 5.908398 0.142836
C 0.720981 5.568888 0.884397
C 0.146811 4.303802 0.754723
H 2.253217 3.016647 -1.596391
H 3.277649 5.248173 -1.340895
H 2.300216 6.893560 0.249238
H 0.278952 6.291781 1.563944
H -0.733424 4.054794 1.338995
C -1.215753 1.436945 -2.886075
C -2.212184 1.678123 -3.832569
C -3.315561 2.465740 -3.499315
C -3.418326 3.022595 -2.221308
C -2.423763 2.786965 -1.275038
H -0.362630 0.810744 -3.133531
H -2.130538 1.244144 -4.824956
H -4.095863 2.646123 -4.232826
H -4.279339 3.629569 -1.958606
H -2.517768 3.209106 -0.278752
C -1.903568 -1.873119 -2.081949
C -3.197707 -1.612312 -2.461307
C -4.029938 -0.770310 -1.686016
C -5.353856 -0.452694 -2.089692
C -6.146204 0.366430 -1.318788
C -5.644312 0.899116 -0.106425
C -4.365039 0.609442 0.310829
C -3.514801 -0.229618 -0.464161
C -2.162581 -0.510167 -0.080574
H -1.302721 -2.528243 -2.698487
H -3.594779 -2.053585 -3.371924
H -5.729067 -0.869288 -3.020787
H -7.157548 0.604667 -1.634959
H -6.275644 1.543622 0.498461
H -3.993368 1.029892 1.237918
C -0.187370 1.591894 2.426868
C -0.597278 1.063258 3.625325
C -1.563245 0.026985 3.675600
C -1.997262 -0.525348 4.909073
C -2.916507 -1.549387 4.937401
C -3.433464 -2.068405 3.726591
C -3.028625 -1.556669 2.514090
C -2.090800 -0.488378 2.447103
C -1.649806 0.073073 1.202208
H 0.573510 2.361060 2.420112
H -0.173608 1.433746 4.555496
H -1.588742 -0.123284 5.832710
H -3.245829 -1.962678 5.886270
H -4.153978 -2.880724 3.754704
H -3.428501 -1.969665 1.595583

TS_{1-4}

Rh -1.725443 0.468762 -0.136353
P -0.552449 -1.443821 0.155068
P 0.151269 1.652192 -0.379195
C -1.689456 -2.646396 0.973149
C 0.103459 -2.325004 -1.307881
C -0.170481 3.139358 -1.420341
C 0.869119 2.281333 1.186220
C 0.881882 -1.233042 1.306963
C 1.558642 0.782050 -1.219362
N -3.316617 2.040191 -0.036018
C -4.283829 1.226911 0.765024
C -4.382252 -0.162245 0.195695
C -5.448333 -0.970312 0.109636
C -5.317986 -2.369163 -0.437575
C -6.831469 -0.593642 0.577326
H -3.898586 1.193150 1.792402
H -5.259043 1.733756 0.801212
H -3.445250 -0.615512 -0.185393
H -7.565089 -0.780024 -0.216826
H -7.123345 -1.224014 1.427869
H -6.923298 0.449602 0.888979
H -5.603385 -3.103578 0.325839
H -5.991581 -2.515876 -1.291664
H -4.295919 -2.599599 -0.752133
C -2.993771 3.291036 0.684925
H -2.518373 3.052471 1.638485
H -2.299825 3.881892 0.086191
H -3.903159 3.882765 0.867896
C -3.918547 2.370007 -1.350040
H -4.165429 1.450012 -1.883422
H -4.836885 2.961750 -1.213738
H -3.204916 2.946507 -1.936904
C -2.469669 -2.197802 2.055660
C -3.378954 -3.049688 2.679840
C -3.534775 -4.361293 2.222401

C -2.777975 -4.811621 1.139791
C -1.860637 -3.962042 0.516642
H -2.366322 -1.173416 2.405393
H -3.969335 -2.688283 3.517060
H -4.244783 -5.026198 2.705867
H -2.899032 -5.827609 0.775185
H -1.285170 -4.328115 -0.326053
C -0.271736 -1.893172 -2.587732
C 0.227176 -2.538870 -3.719633
C 1.098973 -3.620827 -3.580046
C 1.470364 -4.060743 -2.306750
C 0.978493 -3.414396 -1.174252
H -0.936147 -1.038544 -2.687162
H -0.056979 -2.191559 -4.708895
H 1.494180 -4.117725 -4.461228
H 2.160178 -4.891692 -2.196162
H 1.293392 -3.739933 -0.186667
C -0.814664 2.949358 -2.657074
C -1.105290 4.034578 -3.482987
C -0.782874 5.331373 -3.071976
C -0.164402 5.532351 -1.837304
C 0.145814 4.444776 -1.016076
H -1.095042 1.945205 -2.965925
H -1.592535 3.869271 -4.439798
H -1.016886 6.178892 -3.709497
H 0.082722 6.537806 -1.508918
H 0.626298 4.621188 -0.060317
C 0.203731 2.052838 2.399173
C 0.763382 2.486956 3.600674
C 1.988525 3.157152 3.599724
C 2.655261 3.391417 2.394624
C 2.103427 2.950230 1.192965
H -0.731568 1.499498 2.393369
H 0.250713 2.288944 4.537630
H 2.429542 3.486283 4.535897
H 3.616827 3.895426 2.392586
H 2.644240 3.102742 0.262993
C 0.733285 -1.568652 2.680178
C 1.692321 -1.222390 3.600701
C 2.863496 -0.528923 3.207839
C 3.855521 -0.149837 4.151092
C 4.996546 0.505431 3.748381
C 5.190099 0.809680 2.379446
C 4.242970 0.462147 1.442775
C 3.049929 -0.215282 1.822690
C 2.031934 -0.570798 0.875575
H -0.144019 -2.111115 3.010832
H 1.562432 -1.486921 4.647148
H 3.697860 -0.392939 5.198777
H 5.751846 0.786916 4.476351
H 6.093675 1.324984 2.066335
H 4.402327 0.710105 0.399965
C 1.833915 1.060539 -2.586893
C 2.737777 0.310377 -3.297441
C 3.434412 -0.760757 -2.688066
C 4.346125 -1.566743 -3.420151
C 5.022164 -2.597391 -2.808782
C 4.812274 -2.860930 -1.433826
C 3.931719 -2.099173 -0.699168
C 3.210820 -1.027556 -1.299126
C 2.252920 -0.242824 -0.571826
H 1.332211 1.880088 -3.084192
H 2.928664 0.537266 -4.343282
H 4.500996 -1.351536 -4.474259
H 5.718834 -3.207896 -3.375835
H 5.349996 -3.674123 -0.954705
H 3.777684 -2.321808 0.349822

TS$_{1-33}$

Rh -1.830984 0.727665 -0.268019
P -0.762043 -1.199227 0.301180
P 0.160527 1.788162 -0.162071
C -1.871425 -2.122641 1.452595
C -0.392874 -2.307287 -1.113783
C 0.395854 3.483881 -0.864473
C 0.815493 2.018506 1.545300
C 0.838513 -1.198492 1.250692
C 1.349852 0.815477 -1.205104
N -3.575972 2.212865 -0.529118
C -4.549882 1.119956 -0.229072
C -4.132030 -0.122738 -0.982545
C -4.422884 -1.398156 -0.627211
C -4.076405 -2.550436 -1.528870
C -5.198675 -1.768095 0.605087
H -5.571194 1.446705 -0.491027
H -4.539768 0.943946 0.849939
H -3.728098 0.031744 -1.983167
H -4.783359 -2.664008 1.073464
H -5.237945 -0.975535 1.354117
H -6.231431 -2.006422 0.313510
H -3.441470 -3.269461 -0.998933
H -4.990208 -3.087389 -1.816292
H -3.553969 -2.236313 -2.434990
C -3.932917 2.790992 -1.850523
H -4.978474 3.133074 -1.846260
H -3.299069 3.643807 -2.085223
H -3.805260 2.045938 -2.636712
C -3.656864 3.263750 0.511166
H -3.414745 2.827538 1.482578
H -2.931034 4.049946 0.297086
H -4.664182 3.705273 0.550650
C -2.460474 -1.393116 2.501407
C -3.216494 -2.037921 3.479639
C -3.409417 -3.421007 3.416148

C -2.847566 -4.150482 2.366564
C -2.079229 -3.507491 1.391161
H -2.303083 -0.318821 2.556301
H -3.654953 -1.462535 4.290213
H -3.997457 -3.925303 4.177352
H -2.999499 -5.224424 2.306662
H -1.640994 -4.091953 0.589903
C -0.754878 -1.900012 -2.405984
C -0.430410 -2.688119 -3.509950
C 0.254724 -3.891644 -3.332649
C 0.625012 -4.301006 -2.049143
C 0.313970 -3.508615 -0.945055
H -1.259038 -0.946628 -2.538664
H -0.698646 -2.355022 -4.508523
H 0.515681 -4.501284 -4.192657
H 1.179197 -5.224522 -1.910546
H 0.651056 -3.807418 0.043618
C -0.687503 4.178154 -1.401862
C -0.534746 5.459293 -1.937646
C 0.723797 6.058008 -1.947058
C 1.822931 5.365922 -1.427353
C 1.663930 4.089643 -0.891978
H -1.648765 3.694069 -1.410419
H -1.394196 5.982000 -2.348911
H 0.853645 7.053373 -2.361677
H 2.808791 5.821746 -1.442323
H 2.533075 3.567562 -0.509857
C -0.111229 2.067789 2.597080
C 0.310137 2.320498 3.903242
C 1.665359 2.518058 4.173785
C 2.595650 2.465446 3.133298
C 2.173156 2.224621 1.827073
H -1.163040 1.895394 2.383223
H -0.418641 2.356943 4.708355
H 1.996636 2.705755 5.191095
H 3.654717 2.592138 3.337052
H 2.914246 2.153217 1.039612
C 0.855968 -1.594921 2.616615
C 2.010030 -1.531726 3.357666
C 3.217132 -1.058795 2.788931
C 4.403892 -0.936761 3.559849
C 5.556459 -0.432407 3.003121
C 5.568417 -0.034466 1.643479
C 4.437503 -0.154638 0.867366
C 3.225644 -0.670088 1.411226
C 2.014386 -0.754503 0.647184
H -0.047681 -1.956815 3.088716
H 2.002518 -1.837611 4.400685
H 4.380458 -1.239980 4.603386
H 6.457765 -0.336662 3.601489
H 6.479846 0.366363 1.209326
H 4.464085 0.154931 -0.171043
C 1.352299 1.168393 -2.587298
C 2.013119 0.404728 -3.514932
C 2.715388 -0.765150 -3.128746
C 3.395064 -1.569863 -4.080226
C 4.064656 -2.707713 -3.690782
C 4.072619 -3.088176 -2.328384
C 3.418914 -2.328947 -1.383337
C 2.726747 -1.139763 -1.746854
C 2.025849 -0.328950 -0.786465
H 0.824260 2.058114 -2.912568
H 2.004875 0.692953 -4.563153
H 3.377275 -1.268586 -5.124533
H 4.585309 -3.315281 -4.425260
H 4.595463 -3.990842 -2.025340
H 3.426749 -2.642661 -0.346562

TS$_{4-45}$

Rh -1.759919 0.366247 0.352344
P -0.362426 -1.375169 0.813438
P -0.125360 1.526533 -0.596942
C -1.263568 -2.262284 2.148351
C -0.098068 -2.604436 -0.507933
C -0.831641 2.678521 -1.828633
C 0.905937 2.522884 0.540952
C 1.285951 -0.877640 1.459706
C 1.078831 0.483155 -1.547779
N -4.250886 -1.007912 -0.972880
C -4.370211 -0.142415 0.204373
C -3.751687 1.225696 -0.019545
C -3.303665 2.060178 0.992354
C -2.949994 3.499442 0.693058
C -3.505205 1.777162 2.468376
H -5.419036 -0.051646 0.562735
H -3.848490 -0.658656 1.035146
H -3.832936 1.637194 -1.020993
H -4.368512 2.356950 2.827137
H -2.637145 2.105925 3.050457
H -3.697551 0.727473 2.702488
H -2.009242 3.786817 1.175771
H -3.733526 4.156439 1.094977
H -2.861039 3.690145 -0.377393
C -4.435115 -2.413642 -0.611523
H -3.694454 -2.704023 0.140940
H -4.278947 -3.039300 -1.495545
H -5.444082 -2.625489 -0.211233
C -5.170373 -0.615987 -2.039522
H -4.978434 0.413905 -2.354234
H -6.230727 -0.690409 -1.732801
H -5.016867 -1.264565 -2.907220
C -1.773854 -1.510222 3.225534
C -2.567666 -2.115363 4.199121
C -2.875013 -3.475840 4.103067

C -2.377833 -4.227397 3.036473
C -1.576629 -3.627620 2.062098
H -1.545620 -0.449928 3.298472
H -2.947864 -1.525992 5.028749
H -3.498777 -3.947018 4.857010
H -2.612865 -5.285054 2.959783
H -1.201327 -4.221925 1.235868
C -0.905384 -2.544840 -1.654350
C -0.719232 -3.470044 -2.682178
C 0.265710 -4.454288 -2.570517
C 1.062525 -4.522648 -1.424475
C 0.882813 -3.601123 -0.394282
H -1.670989 -1.776316 -1.735113
H -1.337640 -3.416727 -3.573825
H 0.415644 -5.166702 -3.376591
H 1.836159 -5.279685 -1.341555
H 1.521729 -3.638765 0.483474
C -1.819334 2.209838 -2.705457
C -2.440830 3.061245 -3.616812
C -2.096926 4.416364 -3.654428
C -1.125033 4.909228 -2.782991
C -0.492314 4.056638 -1.874415
H -2.107526 1.162545 -2.659602
H -3.197453 2.670545 -4.291491
H -2.587745 5.083331 -4.357131
H -0.858006 5.961838 -2.803779
H 0.247940 4.461262 -1.193668
C 0.564018 2.602634 1.898399
C 1.364540 3.325012 2.782987
C 2.507486 3.978622 2.317281
C 2.850601 3.907793 0.964910
C 2.059122 3.177313 0.079921
H -0.313012 2.069804 2.254202
H 1.102409 3.368233 3.836237
H 3.135648 4.534231 3.007170
H 3.748230 4.399934 0.603624
H 2.352604 3.093904 -0.962850
C 1.523666 -0.878920 2.860458
C 2.649858 -0.288439 3.380910
C 3.609172 0.323330 2.535854
C 4.771791 0.948115 3.060940
C 5.706637 1.513407 2.224130
C 5.515318 1.476046 0.821708
C 4.394184 0.886781 0.282600
C 3.403983 0.296739 1.117711
C 2.213028 -0.301102 0.590301
H 0.811918 -1.350873 3.527992
H 2.820263 -0.293036 4.454511
H 4.912637 0.964865 4.138606
H 6.595042 1.983815 2.635260
H 6.259789 1.918914 0.166279
H 4.256746 0.871755 -0.792412
C 0.933978 0.395549 -2.960362
C 1.662951 -0.506062 -3.695381
C 2.593561 -1.371329 -3.071382
C 3.330164 -2.330925 -3.815371
C 4.234592 -3.160628 -3.193697
C 4.439936 -3.059304 -1.796612
C 3.738745 -2.140704 -1.048326
C 2.790191 -1.269344 -1.656312
C 2.008416 -0.333906 -0.897295
H 0.239733 1.048888 -3.472923
H 1.530716 -0.561568 -4.772899
H 3.166055 -2.395725 -4.887866
H 4.794605 -3.890748 -3.770635
H 5.157628 -3.713949 -1.310473
H 3.905100 -2.083237 0.020642

TS$_{33-114}$

Rh 1.847733 -0.030886 -0.458467
P 0.254689 -1.597595 -0.537484
P 0.293707 1.617205 -0.314617
C 0.684023 -2.867682 -1.792754
C -0.005581 -2.464176 1.057058
C 1.228453 3.175932 -0.067944
C -0.896322 2.102770 -1.616312
C -1.477977 -1.080389 -0.976440
C -0.622734 1.196448 1.219956
N 3.401838 1.065553 2.054733
C 4.284066 0.464022 1.044325
C 3.742820 -0.828282 0.454623
C 3.902321 -1.234738 -0.859927
C 3.859655 -2.709926 -1.186696
C 4.515212 -0.383315 -1.952893
H 5.295172 0.255995 1.456955
H 4.435870 1.213544 0.260295
H 3.519691 -1.611401 1.176811
H 4.009447 -0.570428 -2.906350
H 4.481293 0.692477 -1.756325
H 5.572352 -0.656360 -2.087752
H 3.427945 -2.906635 -2.170033
H 4.891744 -3.088785 -1.200867
H 3.302033 -3.282207 -0.441400
C 3.380961 0.297497 3.298666
H 4.356871 0.316247 3.819058
H 2.620950 0.711482 3.968856
H 3.122024 -0.745941 3.110209
C 3.793765 2.447497 2.333595
H 3.749768 3.043819 1.419861
H 3.096211 2.884725 3.055217
H 4.812721 2.521734 2.757521
C 0.997032 -2.417617 -3.089018
C 1.371865 -3.320167 -4.081677
C 1.464849 -4.684452 -3.786077

C 1.175218 -5.136012 -2.498307
C 0.782429 -4.234950 -1.504723
H 0.959476 -1.354672 -3.311530
H 1.603976 -2.960201 -5.079953
H 1.768026 -5.388526 -4.555388
H 1.255407 -6.192766 -2.260588
H 0.571709 -4.603336 -0.507092
C 0.698618 -2.050235 2.193297
C 0.445541 -2.638330 3.432698
C -0.512716 -3.646159 3.543971
C -1.229381 -4.055681 2.415317
C -0.987909 -3.461652 1.178048
H 1.397471 -1.228128 2.104166
H 0.981468 -2.292854 4.312104
H -0.717725 -4.098764 4.509541
H -1.991003 -4.824833 2.503688
H -1.576141 -3.757727 0.313809
C 2.353777 3.363395 -0.890445
C 3.094576 4.543993 -0.821191
C 2.717704 5.550572 0.071074
C 1.589887 5.379251 0.878089
C 0.843121 4.202282 0.806648
H 2.639662 2.591980 -1.604474
H 3.960555 4.677809 -1.463100
H 3.293581 6.469392 0.129746
H 1.285210 6.167345 1.560654
H -0.038121 4.089730 1.430177
C -0.766797 1.580225 -2.908361
C -1.640433 1.987496 -3.917585
C -2.643493 2.917715 -3.639681
C -2.767447 3.451242 -2.353514
C -1.894260 3.050849 -1.345135
H 0.007836 0.847091 -3.114296
H -1.542203 1.572079 -4.916411
H -3.328713 3.228087 -4.423029
H -3.551125 4.169798 -2.133906
H -2.003181 3.457837 -0.343884
C -2.042921 -1.524081 -2.203343
C -3.264641 -1.064592 -2.630813
C -4.002297 -0.133017 -1.862396
C -5.239607 0.394612 -2.317671
C -5.937714 1.305182 -1.558427
C -5.425945 1.720799 -0.305092
C -4.230284 1.225512 0.163980
C -3.476644 0.288261 -0.598334
C -2.201200 -0.200293 -0.163510
H -1.506342 -2.233759 -2.819613
H -3.673633 -1.410114 -3.576816
H -5.623476 0.066536 -3.280117
H -6.882345 1.705235 -1.915021
H -5.983198 2.437859 0.290880
H -3.850031 1.557603 1.122963
C -0.065461 1.560158 2.478638
C -0.600125 1.075830 3.646432
C -1.701244 0.181689 3.631063
C -2.263350 -0.324563 4.831993
C -3.311312 -1.216444 4.797743
C -3.835202 -1.644218 3.554658
C -3.310608 -1.172571 2.372187
C -2.236629 -0.239134 2.369489
C -1.670302 0.278157 1.157069
H 0.806646 2.200543 2.512548
H -0.174465 1.369625 4.602891
H -1.849433 0.005746 5.781363
H -3.737323 -1.595268 5.722207
H -4.657326 -2.353754 3.534012
H -3.718267 -1.513248 1.427724

TS$_{45-46}$

Rh 1.810591 0.309100 -0.166560
P 0.100517 1.555332 0.569640
P 0.430095 -1.468792 -0.603992
C 0.724835 2.780267 1.793023
C -0.805226 2.501123 -0.709614
C 1.385997 -2.518454 -1.777889
C -0.095869 -2.579047 0.748651
C -1.217200 0.607416 1.462935
C -1.125558 -0.953102 -1.455109
N 4.927575 -0.846349 0.945709
C 4.300162 -0.174352 -0.186992
C 3.786206 1.212367 0.138173
C 3.319282 2.098294 -0.816764
C 3.017719 3.531856 -0.452242
C 3.453857 1.857081 -2.307240
H 4.970251 -0.148824 -1.058481
H 3.454833 -0.830623 -0.585141
H 3.932009 1.539874 1.164466
H 4.353438 2.377071 -2.669774
H 2.604058 2.281749 -2.850322
H 3.545006 0.803970 -2.582421
H 2.054482 3.852692 -0.864010
H 3.787324 4.181594 -0.891192
H 3.006167 3.693951 0.626435
C 4.175780 -2.000847 1.426693
H 3.159493 -1.694668 1.688491
H 4.652867 -2.396992 2.328032
H 4.108118 -2.817100 0.681280
C 6.329068 -1.172274 0.688210
H 6.875404 -0.267790 0.403434
H 6.459398 -1.928684 -0.108945
H 6.783279 -1.561274 1.604506
C 1.653684 2.329048 2.749302
C 2.218238 3.215834 3.664118
C 1.873722 4.570681 3.626865

C 0.958436 5.027352 2.677749
C 0.383744 4.139129 1.765121
H 1.944216 1.281512 2.763220
H 2.932519 2.852743 4.397525
H 2.320832 5.265631 4.331616
H 0.692142 6.079691 2.639925
H -0.309403 4.517465 1.022724
C -0.365411 2.466193 -2.040118
C -1.072981 3.142732 -3.033352
C -2.222724 3.863657 -2.704312
C -2.667483 3.902153 -1.380509
C -1.968824 3.217600 -0.387323
H 0.508419 1.874723 -2.292382
H -0.734941 3.095268 -4.064556
H -2.779305 4.383554 -3.478307
H -3.573012 4.443905 -1.125376
H -2.344219 3.216914 0.632222
C 1.996298 -1.892557 -2.883147
C 2.822140 -2.614675 -3.742741
C 3.068999 -3.969448 -3.500032
C 2.480316 -4.594492 -2.400168
C 1.642138 -3.876895 -1.542436
H 1.823284 -0.835198 -3.067553
H 3.276179 -2.120340 -4.596992
H 3.718493 -4.532066 -4.164191
H 2.671081 -5.645775 -2.204695
H 1.199001 -4.377852 -0.689099
C 0.373190 -2.363438 2.051442
C -0.049907 -3.186998 3.094494
C -0.942627 -4.230710 2.843134
C -1.413817 -4.451282 1.546094
C -0.997171 -3.627204 0.502334
H 1.037519 -1.528394 2.249877
H 0.305218 -3.003370 4.104347
H -1.279703 -4.865638 3.657072
H -2.120414 -5.252252 1.351396
H -1.387318 -3.784770 -0.499568
C -1.241173 0.642403 2.884367
C -2.090609 -0.165779 3.599499
C -2.970642 -1.059549 2.942766
C -3.834915 -1.919874 3.670803
C -4.685195 -2.781236 3.016587
C -4.702811 -2.816299 1.601511
C -3.874752 -1.996627 0.868375
C -2.982581 -1.091326 1.510495
C -2.087870 -0.242341 0.775203
H -0.584318 1.318245 3.417774
H -2.094079 -0.124314 4.685761
H -3.812297 -1.883030 4.756885
H -5.344468 -3.433865 3.581403
H -5.374899 -3.499098 1.089676
H -3.896042 -2.043420 -0.213798
C -1.223057 -1.043751 -2.869661
C -2.279676 -0.476905 -3.540424
C -3.307703 0.201919 -2.840733
C -4.399477 0.802388 -3.522755
C -5.403172 1.435061 -2.825836
C -5.353297 1.493323 -1.412195
C -4.303629 0.928982 -0.723309
C -3.246441 0.268183 -1.410649
C -2.127581 -0.311302 -0.724875
H -0.462096 -1.572951 -3.430535
H -2.341163 -0.554333 -4.623018
H -4.430064 0.746323 -4.607851
H -6.236870 1.886271 -3.355869
H -6.150103 1.990989 -0.866863
H -4.275716 0.989668 0.358268
TS$_{46-47}$
Rh 1.853874 -0.351978 -0.354525
P 0.574414 1.609356 0.100401
P -0.028436 -1.713418 -0.592694
C 1.576021 2.998114 0.800810
C -0.478268 2.392605 -1.179087
C 0.225394 -3.021020 -1.861623
C -0.417899 -2.584232 0.971808
C -0.610909 1.100634 1.445537
C -1.617802 -0.894675 -1.075214
N 5.067633 -0.872213 1.231965
C 4.519519 -0.686932 0.032994
C 3.891352 0.483318 -0.455634
C 3.297334 0.522057 -1.763334
C 3.041725 1.901771 -2.335309
C 3.625472 -0.487091 -2.848947
H 4.657513 -1.519505 -0.644315
H 2.496125 -1.840275 -0.437619
H 3.966635 1.408949 0.104835
H 4.588240 -0.223069 -3.313005
H 2.869139 -0.440833 -3.637698
H 3.672178 -1.520519 -2.503104
H 2.219644 1.896368 -3.054415
H 3.941578 2.230399 -2.876370
H 2.827700 2.647022 -1.566837
C 4.957841 0.103286 2.312979
H 4.980610 1.120488 1.922893
H 5.801710 -0.026940 2.994608
H 4.024321 -0.050290 2.869903
C 5.524214 -2.204047 1.633337
H 5.584908 -2.856748 0.761005
H 4.821405 -2.642301 2.353200
H 6.512503 -2.134599 2.097608
C 2.513882 2.688543 1.803991
C 3.374485 3.659529 2.312870
C 3.335397 4.961728 1.806076
C 2.429587 5.276700 0.793536
C 1.555296 4.306309 0.294433
H 2.570786 1.672462 2.181301
H 4.076681 3.401268 3.101465
H 4.008542 5.720303 2.194445
H 2.397528 6.282990 0.385725
H 0.867631 4.575356 -0.498875
C -0.449122 1.894226 -2.488762
C -1.263560 2.450345 -3.474430
C -2.114668 3.512032 -3.160611
C -2.157163 4.009603 -1.856125
C -1.349575 3.448845 -0.867846
H 0.182869 1.042695 -2.719084
H -1.245816 2.044100 -4.481604
H -2.754951 3.940910 -3.925681
H -2.836511 4.817633 -1.602629
H -1.410611 3.819565 0.151623
C 0.742219 -2.628402 -3.107685
C 0.973024 -3.568228 -4.109733
C 0.706796 -4.920381 -3.872805
C 0.204668 -5.320085 -2.634240
C -0.040536 -4.376814 -1.632067
H 0.961876 -1.580442 -3.287284
H 1.367128 -3.248717 -5.070531
H 0.893472 -5.656599 -4.649274
H 0.000227 -6.369568 -2.442190
H -0.426417 -4.706916 -0.673993
C 0.476165 -2.499649 2.050084
C 0.169984 -3.104953 3.268674
C -1.027365 -3.807775 3.418496
C -1.917413 -3.905376 2.346675
C -1.619717 -3.290412 1.131481
H 1.404086 -1.944669 1.932743
H 0.860368 -3.021277 4.103410
H -1.270926 -4.271696 4.369758
H -2.855690 -4.438724 2.464390
H -2.333759 -3.337419 0.314117
C -0.276250 1.358893 2.804702
C -1.008991 0.828107 3.837702
C -2.144075 0.020957 3.585239
C -2.915689 -0.529501 4.643215
C -4.044311 -1.270611 4.379574
C -4.443687 -1.491840 3.039889
C -3.707503 -0.982961 1.994046
C -2.531601 -0.213622 2.226957
C -1.735842 0.321721 1.155194
H 0.557726 2.006419 3.040817
H -0.732665 1.043775 4.866959
H -2.602117 -0.343727 5.667361
H -4.633268 -1.681145 5.194603
H -5.338307 -2.073055 2.835044
H -4.023779 -1.173361 0.975713
C -2.164209 -1.145751 -2.364064
C -3.253226 -0.445776 -2.822756
C -3.869759 0.546515 -2.023097
C -4.983758 1.293024 -2.491459
C -5.580273 2.242988 -1.694767
C -5.082334 2.484620 -0.391834
C -3.999831 1.781933 0.087131
C -3.355089 0.793352 -0.709511
C -2.210483 0.060127 -0.246599
H -1.719514 -1.902517 -2.998458
H -3.655643 -0.652430 -3.811317
H -5.359021 1.095262 -3.492315
H -6.434370 2.805940 -2.059923
H -5.557842 3.234107 0.234433
H -3.626135 1.986284 1.083481
TS$_{47-48}$
Rh -1.680030 0.849115 -0.090969
P -0.844356 -1.433711 -0.226878
P 0.384507 1.855522 -0.151196
C -2.131308 -2.650595 0.296299
C 0.023395 -2.218442 -1.636883
C 0.358243 3.456705 -1.057685
C 1.024690 2.246301 1.520277
C 0.424294 -1.450200 1.147846
C 1.748050 0.863931 -0.932951
N -5.434430 0.203092 1.066663
C -4.537782 0.878253 0.343617
C -3.813532 0.414277 -0.760385
C -3.055388 1.293182 -1.640431
C -2.693586 0.670731 -2.976702
C -3.432235 2.760921 -1.790690
H -4.380207 1.899439 0.673144
H -2.062421 2.304166 0.414505
H -3.957251 -0.612647 -1.081417
H -4.233354 2.854619 -2.539123
H -2.577051 3.341075 -2.143613
H -3.777369 3.220553 -0.863280
H -1.842568 1.182658 -3.436100
H -3.540774 0.773037 -3.671824
H -2.457791 -0.392615 -2.891372
C -6.008470 -1.074106 0.639201
H -5.285726 -1.666630 0.083634
H -6.895141 -0.892603 0.018622
H -6.302828 -1.647361 1.520322
C -6.092140 0.827251 2.214226
H -5.659627 1.810987 2.405745
H -5.960393 0.204014 3.105545
H -7.165867 0.939650 2.021891
C -2.905466 -2.340971 1.431603
C -3.868045 -3.231342 1.904746
C -4.126647 -4.417920 1.212837
C -3.413659 -4.702734 0.048025
C -2.415645 -3.833173 -0.402562
H -2.747420 -1.399680 1.952551
H -4.426990 -2.991211 2.805385
H -4.882348 -5.109018 1.574816
H -3.619254 -5.613148 -0.507754
H -1.858703 -4.086299 -1.297580
C 0.161133 -1.512247 -2.839237
C 0.868171 -2.066763 -3.905866
C 1.443349 -3.332307 -3.780755
C 1.317245 -4.040311 -2.582725
C 0.618339 -3.484758 -1.512676
H -0.242345 -0.510755 -2.921272
H 0.984866 -1.502269 -4.826386
H 2.002677 -3.760412 -4.607224
H 1.782883 -5.015300 -2.474482
H 0.549869 -4.026785 -0.573493
C -0.042299 3.466760 -2.404991
C -0.131091 4.662944 -3.114346
C 0.157789 5.874244 -2.479164
C 0.539002 5.876271 -1.137451
C 0.643025 4.676153 -0.428621
H -0.283662 2.531859 -2.901137
H -0.433272 4.650331 -4.157830
H 0.082981 6.808695 -3.027579
H 0.760715 6.813657 -0.635352
H 0.940608 4.698943 0.613709
C 0.218604 2.016797 2.644665
C 0.716940 2.250815 3.926405
C 2.018417 2.727409 4.093693
C 2.821466 2.972690 2.976972
C 2.331657 2.726127 1.695799
H -0.794449 1.645173 2.512842
H 0.091655 2.056059 4.792989
H 2.409578 2.902417 5.091590
H 3.836900 3.335332 3.105201
H 2.969577 2.891638 0.831863
C 0.088926 -1.965603 2.431194
C 0.940458 -1.837700 3.501371
C 2.198669 -1.205694 3.360311
C 3.095073 -1.079614 4.454967
C 4.334965 -0.508263 4.286386
C 4.723925 -0.034537 3.010625
C 3.870547 -0.130095 1.935088
C 2.579316 -0.716155 2.069770
C 1.666330 -0.833010 0.964872
H -0.844157 -2.493560 2.574309
H 0.659891 -2.245917 4.469286
H 2.785245 -1.454238 5.427255
H 5.018303 -0.423600 5.126371
H 5.704509 0.414421 2.880499
H 4.182535 0.250123 0.970061
C 2.328549 1.295145 -2.158308
C 3.255025 0.526193 -2.819615
C 3.673669 -0.720626 -2.296783
C 4.631277 -1.525650 -2.969383
C 5.047553 -2.721286 -2.431158
C 4.515157 -3.161449 -1.195714
C 3.578812 -2.407081 -0.525848
C 3.125855 -1.162659 -1.049818
C 2.149974 -0.352173 -0.374060
H 2.056108 2.255521 -2.575820
H 3.688364 0.880651 -3.751601
H 5.033991 -1.174169 -3.915924
H 5.785367 -3.327582 -2.948637
H 4.845982 -4.106534 -0.774565
H 3.174917 -2.765442 0.413347
TS$_{95-96}$
Rh 1.514008 1.348643 -0.255082
P -0.693470 1.754263 0.369484
P 1.080980 -1.011281 -0.593222
C -0.880682 3.236961 1.457877
C -1.853185 2.015813 -1.022079
C 2.258596 -1.830760 -1.762210
C 1.090319 -2.066500 0.907274
C -1.400408 0.370226 1.391921
C -0.584511 -1.275072 -1.376370
N 4.500200 -0.042272 1.252670
C 3.964582 1.043000 0.693612
C 3.669166 1.268247 -0.673376
C 3.158476 2.544140 -1.095743
C 2.926891 2.753664 -2.578602
C 3.581037 3.836679 -0.406931
H 1.410963 2.894411 0.049680
H 3.811517 1.858589 1.390627
H 3.907472 0.514376 -1.414660
H 2.879969 4.641700 -0.644393
H 4.572960 4.130076 -0.779913
H 3.636880 3.761146 0.680963
H 3.823903 3.196398 -3.037160
H 2.101236 3.455261 -2.736738
H 2.699855 1.826679 -3.108329
C 4.680140 -0.128548 2.701158
H 5.734737 -0.310930 2.935396
H 4.080316 -0.953783 3.100494
H 4.365246 0.802819 3.175699
C 4.842000 -1.262741 0.527464
H 4.801049 -1.108967 -0.548053
H 4.139393 -2.057846 0.796175
H 5.855169 -1.571805 0.805217
C 0.138874 3.507241 2.388107
C 0.041607 4.592170 3.257532
C -1.068689 5.439148 3.196863
C -2.077232 5.189102 2.266605
C -1.989359 4.091902 1.405306
H 1.013443 2.862502 2.422041
H 0.835854 4.783661 3.973848
H -1.141284 6.292246 3.865232
H -2.937589 5.849332 2.204475
H -2.779996 3.924951 0.683407
C -1.330029 2.227034 -2.306363
C -2.185381 2.426485 -3.390478
C -3.568734 2.418819 -3.200617
C -4.096914 2.209307 -1.924643
C -3.245062 2.003399 -0.841042
H -0.252855 2.218246 -2.450950
H -1.771698 2.580459 -4.383127
H -4.235054 2.566638 -4.045481
H -5.171765 2.177932 -1.776897
H -3.663879 1.797832 0.139558
C 2.615546 -1.121206 -2.923079
C 3.537125 -1.640915 -3.831758
C 4.131746 -2.881795 -3.588368
C 3.788233 -3.595845 -2.439412
C 2.857270 -3.078373 -1.534831
H 2.159704 -0.154705 -3.115545
H 3.792292 -1.076462 -4.724201
H 4.855596 -3.287688 -4.288885
H 4.243049 -4.562794 -2.243294
H 2.617298 -3.648545 -0.644920
C 1.486990 -1.488948 2.122835
C 1.488666 -2.243425 3.296898
C 1.097689 -3.583913 3.267021
C 0.693730 -4.164786 2.062548
C 0.680216 -3.409191 0.890831
H 1.755066 -0.436438 2.143579
H 1.771955 -1.779084 4.237697
H 1.088883 -4.169586 4.181434
H 0.360219 -5.197574 2.040954
H 0.320892 -3.854772 -0.032348
C -1.421364 0.530762 2.806313
C -1.766292 -0.506560 3.636883
C -2.129002 -1.770547 3.111569
C -2.481782 -2.855184 3.957794
C -2.848153 -4.071628 3.428780
C -2.873551 -4.250533 2.025024
C -2.526923 -3.220251 1.179772
C -2.141905 -1.948097 1.690929
C -1.752001 -0.861651 0.835386
H -1.164777 1.488509 3.241926
H -1.773348 -0.360983 4.714286
H -2.463345 -2.702924 5.033991
H -3.122625 -4.893484 4.083736
H -3.167592 -5.210853 1.610938
H -2.544505 -3.377741 0.107925
C -0.658413 -1.502532 -2.778897
C -1.864079 -1.519366 -3.435443
C -3.079158 -1.339400 -2.731651
C -4.333497 -1.350348 -3.397879
C -5.506382 -1.202734 -2.693894
C -5.468359 -1.037776 -1.288440
C -4.266688 -1.010647 -0.616964
C -3.032562 -1.154109 -1.312981
C -1.761174 -1.094705 -0.646910
H 0.246510 -1.671370 -3.348388
H -1.895651 -1.685444 -4.509326
H -4.348762 -1.484862 -4.476401
H -6.461396 -1.217479 -3.210964
H -6.396742 -0.928486 -0.735002
H -4.257524 -0.873010 0.457703
TS$_{97-101}$
Rh 1.718592 0.673068 -0.101905
P 0.830950 -1.544134 0.068392
P -0.292052 1.716132 0.366008
C 1.817735 -2.990600 -0.518270
C 0.157103 -2.127976 1.671811
C -0.023450 3.137043 1.497459
C -1.118627 2.388215 -1.126494
C -0.544081 -1.435273 -1.164681
C -1.646459 0.715767 1.143382
N 5.458647 -0.386469 -0.356629
C 4.538216 0.519687 0.019462
C 3.766948 1.314087 -0.834779
C 2.935125 2.428786 -0.399460
C 2.504668 3.378517 -1.509909
C 3.329165 3.193212 0.859333
H 4.469254 0.666408 1.092003
H 1.955607 0.726842 1.393218
H 3.865377 1.145943 -1.906634
H 4.225944 3.798466 0.658513
H 2.533895 3.876576 1.161581
H 3.545026 2.548689 1.716136
H 1.617485 3.947956 -1.215443
H 3.303210 4.104870 -1.725643
H 2.272807 2.856830 -2.444977
C 5.799212 -0.576097 -1.763526
H 6.031440 0.388289 -2.226798
H 6.680398 -1.216490 -1.831368
H 4.981690 -1.046802 -2.324851
C 6.031296 -1.318623 0.612723
H 7.118692 -1.360166 0.495180
H 5.801504 -0.980953 1.625811
H 5.616698 -2.325346 0.480203
C 3.133284 -2.759493 -0.934624
C 3.929526 -3.804389 -1.410593
C 3.412228 -5.098312 -1.473942

C 2.098111 -5.340576 -1.061376
C 1.304986 -4.296353 -0.587876
H 3.521661 -1.751563 -0.876138
H 4.948321 -3.608256 -1.734232
H 4.025664 -5.915082 -1.843136
H 1.689938 -6.346021 -1.110351
H 0.288369 -4.505420 -0.275223
C 0.692444 -1.599043 2.855370
C 0.244861 -2.049425 4.098489
C -0.745896 -3.030203 4.171699
C -1.285990 -3.561842 2.998387
C -0.834348 -3.116973 1.757610
H 1.456668 -0.828692 2.802751
H 0.666581 -1.630623 5.007994
H -1.101023 -3.374451 5.138759
H -2.075291 -4.305823 3.045858
H -1.293010 -3.508578 0.856404
C 0.519229 2.880414 2.770192
C 0.847976 3.928181 3.626163
C 0.666288 5.252582 3.214617
C 0.145808 5.517059 1.948176
C -0.202671 4.466891 1.093201
H 0.689852 1.855522 3.089632
H 1.257852 3.713239 4.608907
H 0.933843 6.070607 3.876916
H 0.007279 6.542720 1.618518
H -0.602789 4.693135 0.111210
C -0.602477 2.113824 -2.399073
C -1.271295 2.549606 -3.543007
C -2.458391 3.272696 -3.422537
C -2.982905 3.549211 -2.156773
C -2.324489 3.099674 -1.014029
H 0.306815 1.528958 -2.504603
H -0.872236 2.312997 -4.524751
H -2.982646 3.607917 -4.312488
H -3.914176 4.099848 -2.061070
H -2.750942 3.292294 -0.033365
C -0.216158 -1.815027 -2.501052
C -1.076364 -1.571773 -3.542179
C -2.318440 -0.924844 -3.322884
C -3.217266 -0.665432 -4.390791
C -4.415574 -0.028520 -4.163017
C -4.758007 0.380005 -2.852203
C -3.907360 0.141156 -1.796442
C -2.664132 -0.523969 -1.991336
C -1.754830 -0.786049 -0.907953
H 0.723648 -2.320906 -2.695131
H -0.813666 -1.882526 -4.550413
H -2.940042 -0.981296 -5.393353
H -5.098836 0.163640 -4.985139
H -5.700116 0.891795 -2.678113
H -4.181673 0.470012 -0.801442
C -2.131698 1.060825 2.435404
C -3.088821 0.302042 3.061353
C -3.633753 -0.843414 2.433368
C -4.595848 -1.659519 3.085915
C -5.113255 -2.772621 2.464821
C -4.691614 -3.108243 1.155030
C -3.763456 -2.333515 0.496212
C -3.195470 -1.181780 1.113198
C -2.179345 -0.390153 0.476966
H -1.751012 1.940215 2.938141
H -3.439943 0.578981 4.052130
H -4.913816 -1.387910 4.089200
H -5.847099 -3.392262 2.971860
H -5.107140 -3.984132 0.664979
H -3.451126 -2.608849 -0.504341
TS$_{102-107}$
Rh 1.955806 -0.170669 -0.413756
P 0.135519 -1.708422 -0.370624
P 0.508138 1.612277 -0.247211
C 0.448129 -3.122494 -1.505526
C -0.237932 -2.432627 1.272486
C 1.214681 3.279789 0.120491
C -0.547885 2.040576 -1.689988
C -1.516976 -1.075592 -0.947618
C -0.565938 1.210192 1.203093
N 4.486611 1.224559 1.402172
C 4.279946 0.392784 0.354822
C 3.758736 -0.954271 0.534482
C 3.645280 -1.879213 -0.505332
C 3.388812 -3.322734 -0.133635
C 4.246290 -1.690668 -1.880731
H 4.965466 0.553752 -0.472081
H 3.102952 0.984015 -0.653031
H 3.580959 -1.294508 1.550957
H 3.616413 -2.173645 -2.633273
H 4.379409 -0.646938 -2.174990
H 5.227741 -2.187783 -1.919711
H 2.906200 -3.871015 -0.943903
H 4.353586 -3.813231 0.060929
H 2.771787 -3.418809 0.764619
C 5.216750 2.475723 1.218994
H 4.531215 3.328233 1.145262
H 5.895125 2.632202 2.063862
H 5.814697 2.425426 0.305759
C 3.682001 1.101438 2.614779
H 4.111458 0.370885 3.310918
H 3.639208 2.071200 3.115793
H 2.661272 0.797424 2.358106
C 0.982741 -2.818990 -2.770249
C 1.284963 -3.833527 -3.677460
C 1.070150 -5.169873 -3.326296
C 0.544486 -5.480181 -2.071178
C 0.230010 -4.463522 -1.165210
H 1.168303 -1.781942 -3.039736
H 1.694101 -3.583357 -4.652353
H 1.313662 -5.963092 -4.027050
H 0.379403 -6.516675 -1.791248
H -0.162898 -4.724464 -0.188900
C 0.606131 -2.150862 2.355437
C 0.315294 -2.647998 3.626114
C -0.822607 -3.431244 3.825709
C -1.671495 -3.713745 2.752274
C -1.386272 -3.212405 1.483428
H 1.467063 -1.509363 2.202122
H 0.967599 -2.411109 4.462021
H -1.056332 -3.809976 4.816283
H -2.568237 -4.305795 2.908381
H -2.071507 -3.402289 0.662014
C 2.379195 3.673621 -0.554619
C 2.876863 4.971191 -0.423517
C 2.220590 5.894361 0.393337
C 1.054632 5.516938 1.062429
C 0.548114 4.224130 0.919668
H 2.894638 2.964631 -1.196089
H 3.776325 5.259705 -0.960560
H 2.609778 6.902616 0.500864
H 0.531192 6.231125 1.691699
H -0.367717 3.956083 1.436535
C -0.338122 1.382702 -2.908618
C -1.092728 1.723877 -4.032199
C -2.060289 2.725613 -3.945023
C -2.266281 3.393887 -2.734771
C -1.510063 3.059263 -1.614380
H 0.410562 0.597291 -2.970165
H -0.927716 1.202972 -4.971063
H -2.652838 2.987423 -4.816801
H -3.023447 4.168463 -2.661165
H -1.680174 3.582219 -0.678691
C -2.048169 -1.534063 -2.184219
C -3.180102 -0.972496 -2.723343
C -3.856981 0.080759 -2.063149
C -4.996327 0.709783 -2.631609
C -5.635805 1.735040 -1.973340
C -5.162697 2.169248 -0.711043
C -4.061059 1.578555 -0.134206
C -3.369232 0.522572 -0.791693
C -2.186183 -0.071788 -0.245078
H -1.554076 -2.334145 -2.720546
H -3.563458 -1.330971 -3.675304
H -5.352521 0.366547 -3.599469
H -6.505486 2.211453 -2.416424
H -5.674601 2.976413 -0.194825
H -3.706330 1.924365 0.830139
C -0.070185 1.545030 2.497948
C -0.706201 1.117636 3.635852
C -1.884796 0.332310 3.559653
C -2.565902 -0.101205 4.726929
C -3.712320 -0.857573 4.633166
C -4.217973 -1.216605 3.361726
C -3.571991 -0.820575 2.211530
C -2.390568 -0.029971 2.269890
C -1.692555 0.399508 1.088727
H 0.819745 2.157435 2.586722
H -0.315177 1.387723 4.613788
H -2.167475 0.180781 5.698260
H -4.230882 -1.178972 5.531734
H -5.122528 -1.814035 3.292420
H -3.967834 -1.111695 1.245934
TS$_{107-113}$
Rh 1.870973 -0.031775 -0.341092
P 0.238866 -1.610389 -0.358351
P 0.293118 1.623816 -0.246575
C 0.685744 -2.999456 -1.474955
C -0.028140 -2.327688 1.307286
C 0.989865 3.307439 0.024386
C -0.790963 1.966477 -1.687440
C -1.474549 -1.152293 -0.917396
C -0.747552 1.215431 1.220727
N 4.506336 1.172737 1.684536
C 4.161202 0.691756 0.350036
C 3.848333 -0.784214 0.336290
C 3.859425 -1.588378 -0.781123
C 3.802932 -3.086140 -0.602817
C 4.275049 -1.126185 -2.157281
H 4.937063 0.945958 -0.386473
H 3.277507 1.307605 -0.042165
H 3.781964 -1.241069 1.321326
H 3.620201 -1.560367 -2.918626
H 4.270534 -0.040706 -2.281224
H 5.291574 -1.491395 -2.366979
H 3.324298 -3.584832 -1.447021
H 4.833712 -3.464026 -0.544890
H 3.284850 -3.373891 0.315898
C 5.909403 1.567795 1.776722
H 6.148121 2.466387 1.176650
H 6.156172 1.779969 2.821439
H 6.550542 0.745827 1.442761
C 3.603867 2.183942 2.222138
H 2.581893 1.797087 2.206570
H 3.872548 2.384730 3.263823
H 3.625577 3.137799 1.665660
C 1.110177 -2.676735 -2.776453
C 1.477832 -3.679436 -3.671847
C 1.440710 -5.019578 -3.272406
C 1.028455 -5.346794 -1.979979
C 0.648809 -4.343749 -1.083798
H 1.155662 -1.635008 -3.082625
H 1.799684 -3.417040 -4.675709
H 1.734824 -5.802266 -3.965493
H 1.003515 -6.385287 -1.662857
H 0.341892 -4.614841 -0.079884
C 0.796451 -1.949899 2.376772
C 0.562510 -2.455003 3.656177
C -0.493743 -3.340378 3.876894
C -1.319621 -3.720418 2.815568
C -1.094435 -3.212514 1.537610
H 1.593740 -1.233023 2.208194
H 1.196854 -2.144547 4.481437
H -0.682108 -3.725215 4.874761
H -2.152921 -4.394877 2.987544
H -1.763066 -3.482414 0.724932
C 2.112637 3.670629 -0.737227
C 2.646092 4.955994 -0.609944
C 2.061555 5.898503 0.199689
C 0.928989 5.555184 0.940166
C 0.387819 4.271265 0.846971
H 2.564104 2.951278 -1.416365
H 3.514970 5.221239 -1.245405
H 2.479552 6.897986 0.275018
H 0.459080 6.288592 1.589132
H -0.501864 4.028278 1.418697
C -0.535808 1.345862 -2.915782
C -1.316743 1.652209 -4.031493
C -2.353643 2.580213 -3.924713
C -2.603649 3.212006 -2.702948
C -1.821877 2.913440 -1.589985
H 0.266387 0.616859 -2.989050
H -1.118315 1.161092 -4.979889
H -2.965979 2.813584 -4.790822
H -3.413340 3.929910 -2.615233
H -2.023712 3.405346 -0.643166
C -1.963957 -1.668550 -2.148973
C -3.151755 -1.230555 -2.681254
C -3.927573 -0.247415 -2.022015
C -5.124946 0.263257 -2.589362
C -5.858449 1.225747 -1.934412
C -5.425144 1.712123 -0.676836
C -4.270250 1.233972 -0.100031
C -3.481509 0.245623 -0.753945
C -2.242897 -0.225010 -0.209167
H -1.395874 -2.416600 -2.686042
H -3.502595 -1.632080 -3.628474
H -5.449334 -0.118889 -3.553737
H -6.771645 1.612947 -2.376795
H -6.010521 2.469890 -0.163999
H -3.948226 1.619132 0.860666
C -0.284206 1.616783 2.507321
C -0.881406 1.151367 3.651753
C -1.979930 0.257130 3.589391
C -2.612100 -0.225747 4.765066
C -3.677355 -1.093885 4.685644
C -4.148340 -1.518903 3.420933
C -3.548832 -1.075820 2.262934
C -2.451159 -0.171423 2.306534
C -1.796834 0.304574 1.119535
H 0.552532 2.299536 2.589747
H -0.514844 1.469573 4.624415
H -2.240871 0.107273 5.730881
H -4.158727 -1.453512 5.590351
H -4.989031 -2.204360 3.363495
H -3.917219 -1.417618 1.303039
TS$_{113-114}$
Rh 1.801434 0.207740 -0.696725
P 0.439002 -1.554557 -0.472867
P 0.045341 1.659283 -0.419368
C 0.957313 -2.901157 -1.609026
C 0.451377 -2.258450 1.232325
C 0.667547 3.379311 -0.229047
C -1.264197 1.880022 -1.680094
C -1.373812 -1.333528 -0.830244
C -0.723832 1.167174 1.179508
N 3.626259 1.257345 1.984775
C 3.957902 1.067504 0.578535
C 3.767383 -0.358867 0.104534
C 3.940965 -0.765446 -1.208308
C 4.136844 -2.229712 -1.515624
C 4.361600 0.169757 -2.322584
H 4.981135 1.424543 0.326985
H 3.299245 1.754943 -0.004184
H 3.715391 -1.125182 0.872441
H 3.829108 -0.070434 -3.249694
H 4.202393 1.230006 -2.101209
H 5.435231 0.040372 -2.525628
H 3.675773 -2.519403 -2.462353
H 5.215043 -2.425413 -1.603466
H 3.739345 -2.873894 -0.728318
C 4.599428 0.616076 2.870412
H 5.617855 1.032349 2.755007
H 4.286526 0.757871 3.909211
H 4.646403 -0.459755 2.681336
C 3.503714 2.680430 2.302216
H 2.750036 3.144869 1.659753
H 3.185644 2.793526 3.343287
H 4.454208 3.231571 2.172576
C 1.100541 -2.578733 -2.971411
C 1.506114 -3.544640 -3.889946
C 1.803885 -4.840202 -3.454517
C 1.687637 -5.161052 -2.101727
C 1.261590 -4.199674 -1.180951
H 0.901497 -1.564030 -3.306637
H 1.603302 -3.284952 -4.940251
H 2.130713 -5.591977 -4.166898
H 1.927347 -6.162731 -1.756954
H 1.181537 -4.465090 -0.132707
C 1.212367 -1.617084 2.238587
C 1.160112 -2.104073 3.545459
C 0.351309 -3.196807 3.859240
C -0.419257 -3.802294 2.862707
C -0.377694 -3.317973 1.556892
H 1.798527 -0.730509 2.017885
H 1.737457 -1.610930 4.322314
H 0.304856 -3.565855 4.879528
H -1.067521 -4.638619 3.106645
H -1.006929 -3.770369 0.795231
C 1.737124 3.773510 -1.051032
C 2.231980 5.076852 -0.998126
C 1.659415 6.004508 -0.125032
C 0.579431 5.629257 0.677698
C 0.079522 4.327238 0.622740
H 2.175333 3.064050 -1.750790
H 3.059310 5.367223 -1.639193
H 2.046200 7.018147 -0.077507
H 0.121418 6.351915 1.346863
H -0.761311 4.051954 1.251447
C -1.123189 1.274705 -2.934906
C -2.093298 1.469762 -3.918950
C -3.205498 2.270529 -3.653920
C -3.343447 2.886743 -2.406771
C -2.374693 2.698199 -1.424155
H -0.261780 0.641594 -3.129398
H -1.983429 0.990469 -4.887500
H -3.964756 2.415869 -4.416741
H -4.211097 3.504509 -2.196427
H -2.493267 3.170958 -0.453676
C -1.934084 -1.938635 -1.989019
C -3.225338 -1.675126 -2.375745
C -4.043248 -0.791064 -1.632635
C -5.360587 -0.464022 -2.049943
C -6.137102 0.400478 -1.313138
C -5.626060 0.969661 -0.121336
C -4.353248 0.670702 0.309295
C -3.518717 -0.212982 -0.432170
C -2.170316 -0.498683 -0.039744
H -1.340867 -2.620404 -2.583935
H -3.629990 -2.143415 -3.269365
H -5.743212 -0.908987 -2.964762
H -7.143090 0.646505 -1.640265
H -6.245302 1.649184 0.457200
H -3.974225 1.118361 1.220678
C -0.159389 1.661811 2.389682
C -0.566163 1.173827 3.606095
C -1.545012 0.151866 3.693576
C -1.977690 -0.357266 4.945903
C -2.910656 -1.367198 5.011122
C -3.442952 -1.914907 3.819878
C -3.039184 -1.445653 2.589827
C -2.087327 -0.393115 2.484609
C -1.646713 0.123655 1.219829
H 0.611099 2.421463 2.354788
H -0.130745 1.565899 4.521799
H -1.557439 0.066806 5.854309
H -3.239017 -1.747217 5.974129
H -4.174656 -2.715640 3.876981
H -3.451172 -1.880302 1.686800

3.5 Associative mechanism (B3LYP+D3/BS2 level)

291
Rh 1.220129 0.953980 -0.593123
P 0.322059 -1.073981 -0.975492
P -0.554110 1.578613 0.600331
C 1.238468 -1.731798 -2.436002
C 0.356570 -2.417866 0.268229
C 0.008000 2.776109 1.886711
C -1.942658 2.374912 -0.296577
C -1.449429 -0.946590 -1.500633
C -1.387003 0.229866 1.568715
N 5.179355 -0.696613 -0.813003
C 3.899484 -0.579792 -0.146232
C 3.970558 -0.188972 1.311551
C 4.328659 -1.012189 2.309271
C 4.305204 -0.550883 3.745033
C 4.746329 -2.445085 2.094127
H 3.228406 0.177906 -0.674732
H 3.377001 -1.533937 -0.264747
H 3.690289 0.832430 1.555521
H 4.940891 -2.671429 1.044217
H 3.973239 -3.130757 2.466795
H 5.662024 -2.662997 2.657880
H 5.301084 -0.628474 4.200494
H 3.639530 -1.187065 4.345219
H 3.964113 0.485779 3.842051
C 6.032405 0.477388 -0.709892
H 5.647727 1.341625 -1.287321
H 7.028241 0.238830 -1.096490
H 6.133338 0.773763 0.337506
C 5.044710 -1.156480 -2.187445
H 4.536912 -0.416737 -2.836324
H 4.459853 -2.079346 -2.218965
H 6.034880 -1.356824 -2.608847
C 1.508640 -0.846804 -3.497758
C 2.225948 -1.271315 -4.615254
C 2.695294 -2.585649 -4.685300
C 2.443997 -3.467947 -3.632829
C 1.723945 -3.045880 -2.512460
H 1.144752 0.177213 -3.447684
H 2.420201 -0.577539 -5.428399
H 3.255429 -2.918717 -5.554140
H 2.810261 -4.489550 -3.679665
H 1.546134 -3.742574 -1.701009
C 1.045645 -2.218902 1.471547
C 1.049378 -3.215254 2.448707
C 0.372856 -4.416560 2.228839
C -0.311378 -4.622557 1.027721
C -0.326545 -3.626070 0.053487
H 1.563794 -1.282379 1.646208
H 1.568883 -3.044507 3.387384
H 0.368628 -5.187547 2.993728
H -0.853239 -5.548387 0.860599
H -0.891054 -3.776016 -0.862886
C 1.148157 2.427979 2.636093
C 1.656100 3.294537 3.602727
C 1.046846 4.534331 3.819776
C -0.074331 4.894800 3.071021
C -0.596070 4.021200 2.112405
H 1.637888 1.474882 2.452913
H 2.530913 3.006084 4.179291
H 1.446329 5.215055 4.565805
H -0.549207 5.858513 3.231153
H -1.463934 4.321572 1.535724
C -1.864259 2.533371 -1.687201
C -2.938968 3.067873 -2.397113
C -4.099850 3.452242 -1.723111
C -4.187393 3.293360 -0.337862
C -3.118939 2.748218 0.372868
H -0.974734 2.190288 -2.208234
H -2.877051 3.166711 -3.477032
H -4.941083 3.858874 -2.276411
H -5.097045 3.571339 0.185929
H -3.210147 2.588047 1.443798
C -1.776113 -0.923974 -2.883696
C -3.050879 -0.630076 -3.303717
C -4.078511 -0.343563 -2.371209
C -5.397580 -0.026712 -2.791829
C -6.386274 0.235517 -1.871382
C -6.091791 0.194185 -0.487389
C -4.821319 -0.103630 -0.048995
C -3.774323 -0.383960 -0.971793
C -2.437240 -0.688973 -0.549182
H -1.015181 -1.149298 -3.621300
H -3.285494 -0.622277 -4.365274
H -5.612258 0.000426 -3.857121
H -7.393338 0.471959 -2.202459
H -6.875683 0.403347 0.234932
H -4.609335 -0.122593 1.013538
C -1.150983 0.123428 2.967197
C -1.620230 -0.947045 3.687815
C -2.359192 -1.979621 3.061913
C -2.822749 -3.107981 3.789059
C -3.543080 -4.100255 3.164903
C -3.828499 -4.001044 1.781981
C -3.389538 -2.920846 1.050042
C -2.641255 -1.874994 1.661725
C -2.139700 -0.752266 0.920197
H -0.597638 0.897481 3.482747
H -1.426567 -1.009529 4.755744
H -2.598020 -3.171435 4.850674
H -3.895462 -4.958773 3.729204
H -4.397287 -4.786958 1.293299
H -3.609678 -2.866961 -0.009320
N 2.225263 2.959517 -0.970840
C 2.797973 2.756941 -2.326564
H 1.989779 2.581216 -3.041203
H 3.468526 1.894400 -2.327097
H 3.367936 3.642690 -2.646394
C 1.322619 4.135555 -1.021069
H 0.920712 4.328805 -0.027288
H 0.496494 3.938969 -1.704733
H 1.870744 5.026836 -1.361680
C 3.321805 3.250904 -0.016562
H 4.030419 2.423130 -0.005272
H 2.903686 3.379281 0.982654
H 3.855946 4.169156 -0.305754

359
Rh -0.465716 1.334247 1.419309
P -0.141145 -0.874696 1.578767
P 0.583825 1.512143 -0.707017
C -0.487662 -1.559853 3.274426
C -0.933604 -2.112733 0.457805
C -0.426737 2.676276 -1.729914
C 2.323159 2.099677 -0.858267
C 1.675778 -1.161271 1.285656
C 0.670447 -0.032978 -1.734346
N -4.322906 0.109081 1.133975
C -4.806314 -0.513964 0.082764
C -4.978161 0.048392 -1.216766
C -5.484809 -0.622129 -2.291736
C -5.585402 0.070381 -3.620804
C -5.979336 -2.041130 -2.277856
H -1.277944 1.178487 2.799952
H -5.070362 -1.550924 0.251538
H -4.665361 1.075187 -1.371432
H -5.779883 -2.583800 -1.352500
H -5.525891 -2.604612 -3.102639
H -7.063212 -2.053247 -2.454961
H -6.621475 0.057418 -3.984681
H -4.988534 -0.464128 -4.372289
H -5.242039 1.107825 -3.583335
C -3.782629 1.454752 1.126623
H -2.653672 1.380709 1.063821
H -4.017312 1.931274 2.080052
H -4.173358 2.052093 0.306116
C -4.070738 -0.614337 2.389274
H -3.014512 -0.491709 2.649476
H -4.302179 -1.672966 2.269280
H -4.685395 -0.182804 3.184950
C -0.088963 -0.788331 4.382529
C -0.342242 -1.215615 5.683990
C -1.033628 -2.409876 5.909394
C -1.460975 -3.169338 4.821748
C -1.185018 -2.752726 3.515032
H 0.401066 0.166060 4.215967
H -0.013790 -0.608072 6.522819
H -1.242969 -2.738179 6.923550
H -2.008310 -4.094263 4.982979
H -1.532131 -3.364383 2.690876
C -1.887925 -1.687034 -0.472579
C -2.461073 -2.588379 -1.370377
C -2.096169 -3.935697 -1.339048
C -1.142107 -4.372543 -0.417474
C -0.554876 -3.465313 0.464047
H -2.131750 -0.631699 -0.521912
H -3.164209 -2.228386 -2.114569
H -2.530428 -4.637120 -2.045622
H -0.828152 -5.411747 -0.409280
H 0.222301 -3.805129 1.142568
C -1.790940 2.367422 -1.902230
C -2.658677 3.266241 -2.516998
C -2.189055 4.510238 -2.953025
C -0.843271 4.831899 -2.781984
C 0.033301 3.920788 -2.182061
H -2.167809 1.413682 -1.544780
H -3.706180 3.005346 -2.653163
H -2.866203 5.217855 -3.422442
H -0.466669 5.793791 -3.118369
H 1.077088 4.191315 -2.062448
C 3.104327 2.167810 0.302997
C 4.456511 2.504577 0.227379
C 5.038249 2.782614 -1.010129
C 4.270192 2.706924 -2.175736
C 2.923603 2.353606 -2.102444
H 2.656559 1.912442 1.259619
H 5.057742 2.530435 1.131471
H 6.091709 3.040452 -1.070051
H 4.725158 2.905780 -3.142133
H 2.339752 2.263139 -3.014858
C 2.550051 -1.229920 2.406618
C 3.914007 -1.201747 2.252243
C 4.497901 -1.083429 0.966661
C 5.905383 -1.011573 0.792358
C 6.453497 -0.881299 -0.462853
C 5.608992 -0.818799 -1.597002
C 4.241585 -0.897552 -1.460592
C 3.638537 -1.033208 -0.178146
C 2.212749 -1.092728 -0.002898
H 2.135639 -1.319521 3.403362
H 4.562317 -1.268012 3.122723
H 6.541913 -1.057477 1.672588
H 7.531088 -0.823962 -0.587122
H 6.044715 -0.702796 -2.585261
H 3.610214 -0.835410 -2.338722
C -0.017785 -0.127631 -2.974638
C -0.053042 -1.303107 -3.683908
C 0.607387 -2.459952 -3.204300
C 0.552376 -3.693743 -3.905707
C 1.213917 -4.803976 -3.433083
C 1.963721 -4.720044 -2.235467
C 2.032274 -3.538384 -1.531850
C 1.353057 -2.371975 -1.984867
C 1.371845 -1.137603 -1.249453
H -0.523438 0.742423 -3.375676
H -0.587073 -1.354580 -4.630036
H -0.019660 -3.742418 -4.829167
H 1.169169 -5.742316 -3.978575
H 2.489826 -5.596785 -1.868337
H 2.605077 -3.493567 -0.613206
N -0.557365 3.589155 1.902683
C -0.694393 3.741269 3.374077
H 0.136282 3.234199 3.868339
H -1.623347 3.276891 3.704815
H -0.691795 4.807253 3.654724
C 0.679735 4.294451 1.493474
H 0.798875 4.240819 0.412253
H 1.541948 3.823804 1.966019
H 0.641317 5.354481 1.791574
C -1.708285 4.253090 1.250466
H -2.635103 3.791648 1.594523
H -1.634617 4.146901 0.168261
H -1.738672 5.324897 1.505462

368
Rh 1.580530 0.358792 -0.881314
P 0.291765 -1.525098 -0.717398
P -0.159201 1.709859 0.310026
C 0.978467 -2.882616 -1.780228
C -0.026905 -2.362061 0.889813
C 0.514148 3.124141 1.284013
C -1.344358 2.467008 -0.878171
C -1.421850 -1.149183 -1.326902
C -1.309329 0.800979 1.447675
N 4.484414 -0.958405 -0.476221
C 3.296112 -0.713846 0.202671
C 3.415113 0.087283 1.454905
C 3.602759 -0.430656 2.685165
C 3.733541 0.466020 3.891666
C 3.743004 -1.902696 2.975434
H 2.335663 -0.423665 -1.999882
H 2.765882 -1.655743 0.330294
H 3.396892 1.166844 1.357872
H 3.523172 -2.538415 2.115600
H 3.083531 -2.204620 3.797524
H 4.770213 -2.117706 3.300003
H 4.643222 0.227830 4.457458
H 2.890827 0.315892 4.581669
H 3.770855 1.524570 3.618944
C 5.695527 -0.173030 -0.307642
H 5.858597 0.557478 -1.112966
H 6.559659 -0.847357 -0.296954
H 5.665829 0.354079 0.646894
C 4.463469 -1.887936 -1.588233
H 4.128000 -1.399615 -2.516696
H 3.766798 -2.705595 -1.385266
H 5.464063 -2.301613 -1.743165
C 1.299408 -2.604588 -3.122687
C 1.906675 -3.563587 -3.928790
C 2.241004 -4.815119 -3.401641
C 1.956301 -5.094485 -2.066647
C 1.326016 -4.139094 -1.261742
H 1.104500 -1.618135 -3.531065
H 2.135379 -3.328326 -4.964468
H 2.726713 -5.559309 -4.026105
H 2.223289 -6.057372 -1.640434
H 1.122622 -4.383096 -0.226246
C 0.502502 -1.856337 2.081819
C 0.200162 -2.463966 3.300770
C -0.623944 -3.589250 3.340402
C -1.156648 -4.101031 2.154751
C -0.869896 -3.485937 0.937940
H 1.119582 -0.967364 2.061184
H 0.594112 -2.043899 4.221694
H -0.865510 -4.056227 4.290472
H -1.817938 -4.961621 2.180394
H -1.315438 -3.869931 0.024549
C 1.381655 2.835547 2.354794
C 2.025122 3.857908 3.049817
C 1.840810 5.190416 2.668940
C 0.999290 5.488292 1.597437
C 0.335379 4.466091 0.912927
H 1.559079 1.803764 2.639292
H 2.674612 3.615625 3.886277
H 2.349471 5.987058 3.203757
H 0.849628 6.519981 1.291936
H -0.319707 4.723811 0.088158
C -1.281320 2.131672 -2.237745
C -2.225197 2.628328 -3.135511
C -3.243846 3.469027 -2.683704
C -3.321675 3.802161 -1.329285
C -2.384662 3.296443 -0.429276
H -0.520903 1.438506 -2.585773
H -2.177346 2.340747 -4.181705
H -3.986017 3.847878 -3.379957
H -4.124827 4.438877 -0.970057
H -2.469704 3.535110 0.627387
C -1.750381 -1.439233 -2.680789
C -2.944671 -1.042017 -3.228386
C -3.890440 -0.318883 -2.461729
C -5.124315 0.108888 -3.019134
C -6.036691 0.803537 -2.258931
C -5.743506 1.102426 -0.907421
C -4.554326 0.702261 -0.340918
C -3.588896 -0.026312 -1.092613
C -2.338957 -0.461563 -0.524862
H -1.062526 -2.010902 -3.289800
H -3.179517 -1.290927 -4.260344
H -5.334706 -0.126125 -4.059435
H -6.980184 1.123451 -2.691618
H -6.462128 1.658255 -0.311977
H -4.343836 0.952030 0.691664
C -1.276645 1.051329 2.846543
C -2.012845 0.295602 3.725201
C -2.835249 -0.759492 3.263356
C -3.583380 -1.565176 4.162546
C -4.396839 -2.572047 3.696436
C -4.491784 -2.811964 2.304989
C -3.772809 -2.051731 1.410351
C -2.918627 -1.002406 1.854652
C -2.139670 -0.204804 0.945298
H -0.676507 1.864137 3.234723
H -1.975440 0.509152 4.790631
H -3.504138 -1.366166 5.228334
H -4.969102 -3.180318 4.390875
H -5.135347 -3.607151 1.939542
H -3.850341 -2.260172 0.350226
N 2.782033 2.177121 -1.688423
C 3.672226 1.706832 -2.780629
H 3.066342 1.277160 -3.579994
H 4.339087 0.936011 -2.404461
H 4.266708 2.541913 -3.182870
C 1.870782 3.190710 -2.274539
H 1.220945 3.600990 -1.503374
H 1.258316 2.732289 -3.050477
H 2.449343 4.014166 -2.720649
C 3.604884 2.855402 -0.659575
H 4.346037 2.161836 -0.267250
H 2.965500 3.199710 0.153840
H 4.131079 3.720618 -1.091432

TS$_{291-359}$
Rh 1.172935 1.258722 -0.299040
P 0.735353 -0.869553 -0.750909
P -0.997601 1.620419 0.555468
C 1.876384 -1.586947 -2.038319
C 0.749951 -2.107454 0.619057
C -1.019880 2.917498 1.876875
C -2.328776 2.103319 -0.618929
C -0.948400 -1.097853 -1.534897
C -1.738704 0.150996 1.437823
N 5.431323 0.469329 -0.828855
C 4.731826 -0.423835 -0.163908
C 4.696305 -0.536872 1.261009
C 4.448720 -1.702692 1.922402
C 4.487463 -1.728337 3.422838
C 4.201984 -3.033421 1.273532
H 2.723355 0.903462 -0.701359
H 4.236382 -1.169151 -0.776092
H 4.916510 0.350289 1.846016
H 4.193763 -3.008032 0.183924
H 3.245033 -3.441265 1.617969
H 4.981445 -3.739195 1.590843
H 5.315679 -2.361915 3.768201
H 3.567488 -2.180160 3.810098
H 4.605581 -0.733142 3.860194
C 6.131128 1.568470 -0.157805
H 5.412018 2.331142 0.160580
H 6.840290 2.015264 -0.855243
H 6.677444 1.195078 0.710270
C 5.306722 0.550921 -2.286132
H 4.526177 1.275068 -2.542284
H 5.017259 -0.419729 -2.687062
H 6.262875 0.857635 -2.715428
C 2.178979 -0.773652 -3.146140
C 2.997417 -1.234841 -4.176273
C 3.581019 -2.504247 -4.095441
C 3.320995 -3.307124 -2.984686
C 2.461529 -2.859593 -1.973489
H 1.770439 0.232729 -3.184276
H 3.194595 -0.597827 -5.034776
H 4.229649 -2.861403 -4.890350
H 3.769779 -4.293845 -2.907749
H 2.257077 -3.513046 -1.134028
C 1.068489 -1.654909 1.907819
C 0.993991 -2.521115 2.999370
C 0.641207 -3.860291 2.811203
C 0.336560 -4.323594 1.528922
C 0.369145 -3.447169 0.443769
H 1.333137 -0.608870 2.040698
H 1.187626 -2.145712 4.000700
H 0.576985 -4.533139 3.661290
H 0.027871 -5.353911 1.381239
H 0.056673 -3.798348 -0.535659
C -0.000359 2.852571 2.845059
C 0.092448 3.810515 3.854383
C -0.820995 4.867876 3.899842
C -1.826535 4.951702 2.936042
C -1.930489 3.981832 1.934384
H 0.724184 2.042143 2.796419
H 0.880455 3.737332 4.599366
H -0.746296 5.621187 4.678845
H -2.537254 5.773119 2.961137
H -2.714676 4.069702 1.190601
C -1.978352 2.309917 -1.961163

C -2.956174 2.614905 -2.908460
C -4.293992 2.719025 -2.522526
C -4.652619 2.512613 -1.188019
C -3.677350 2.198512 -0.242368
H -0.939974 2.185683 -2.258651
H -2.676438 2.754156 -3.949104
H -5.058080 2.945315 -3.260563
H -5.695158 2.570703 -0.889343
H -3.967729 1.998148 0.785546
C -1.050522 -1.154481 -2.953686
C -2.266421 -1.114337 -3.589702
C -3.471695 -1.025427 -2.852451
C -4.736947 -0.978159 -3.495640
C -5.898455 -0.912163 -2.761092
C -5.836024 -0.885759 -1.347628
C -4.623085 -0.921044 -0.697287
C -3.400204 -0.994193 -1.423392
C -2.117150 -1.023062 -0.773829
H -0.155785 -1.239150 -3.556390
H -2.313811 -1.160039 -4.675122
H -4.769341 -0.999930 -4.582138
H -6.862325 -0.881329 -3.261054
H -6.754542 -0.830980 -0.770036
H -4.595677 -0.886423 0.385035
C -1.814149 0.135146 2.858287
C -2.196984 -0.991459 3.544341
C -2.537607 -2.181159 2.857293
C -2.908239 -3.364188 3.550564
C -3.249370 -4.506677 2.863474
C -3.233966 -4.508066 1.447994
C -2.873547 -3.377669 0.749180
C -2.509264 -2.179060 1.426187
C -2.099855 -0.993616 0.725678
H -1.571467 1.028059 3.419835
H -2.246266 -0.976618 4.630609
H -2.921865 -3.347482 4.637641
H -3.535941 -5.405367 3.402288
H -3.508360 -5.410274 0.908409
H -2.860159 -3.398115 -0.334161
N 1.865441 3.457954 -0.422774
C 2.722275 3.558855 -1.626321
H 2.159187 3.231818 -2.503358
H 3.587331 2.908699 -1.507863
H 3.062564 4.596995 -1.778560
C 0.775502 4.452018 -0.573418
H 0.157713 4.463812 0.321912
H 0.152686 4.181166 -1.427835
H 1.187112 5.461650 -0.731380
C 2.669434 3.807456 0.768070
H 3.440621 3.047935 0.914334
H 2.026295 3.823921 1.648243
H 3.141974 4.797640 0.653058
